# Carboranyl-1,8-naphthalimide intercalators induce lysosomal membrane permeabilization and ferroptosis in cancer cell lines

**DOI:** 10.1080/14756366.2023.2171028

**Published:** 2023-01-30

**Authors:** Sebastian Rykowski, Dorota Gurda-Woźna, Agnieszka Fedoruk-Wyszomirska, Marta Orlicka-Płocka, Aleksandra Kowalczyk, Paweł Stączek, Marta Denel-Bobrowska, Katarzyna Biniek-Antosiak, Wojciech Rypniewski, Eliza Wyszko, Agnieszka B. Olejniczak

**Affiliations:** a Institute of Medical Biology, Polish Academy of Sciences, Łódź, Poland; b Institute of Bioorganic Chemistry, Polish Academy of Sciences, Poznan, Poland; c Department of Molecular Microbiology, Faculty of Biology and Environmental Protection, University of Lodz, Łódź, Poland

**Keywords:** Carborane, 1,8-naphthalimides, intercalation, anticancer activity

## Abstract

The synthesis of carborane-1,8-naphthalimide conjugates and evaluation of their DNA-binding ability and anticancer activity were performed. A series of 4-carboranyl-3-nitro-1,8-naphthalimide derivatives, mitonafide and pinafide analogs, were synthesised via amidation and reductive amination reactions, and their calf thymus DNA (ct-DNA)-binding properties were investigated using circular dichroism, UV–vis spectroscopy, and thermal denaturation. Results showed that conjugates **34**–**37** interacted very strongly with ct-DNA (Δ*T*_m_ = 10.00–13.00 °C), indicating their ability to intercalate with DNA, but did not inhibit the activity of topoisomerase II. The conjugates inhibited the cell growth of the HepG2 cancer cell line *in vitro*. The same compounds caused the G2M phase arrest. Cell lines treated with these conjugates showed an increase in reactive oxygen species, glutathione, and Fe^2+^ levels, lipid peroxidation, and mitochondrial membrane potential relative to controls, indicating the involvement of ferroptosis. Furthermore, these conjugates caused lysosomal membrane permeabilization in HepG2 cells but not in MRC-5 cells.

## Introduction

1,8-Naphthalimides are a class of polycyclic imides consisting of π-deficient flat aromatic or heteroaromatic ring systems. The majority of the 1,8-naphthalimides exhibit a wide range of biological properties such as anticancer, antibacterial, and antiviral activities^1^. This group of compounds and their derivatives are primarily considered as DNA intercalators, and their high antitumor activity and ability to suppress tumour growth and metastasis are usually attributed to their potential to interact with DNA^2^^,^^3^. However, 1,8-naphthalimide derivatives demonstrate anticancer activities in several other ways such as induction of reactive oxygen species (ROS), malfunction of lysosome and mitochondria, and so on^4–6^. They have also been reported to inhibit topoisomerases (Topo I and Topo II)^7^^,^^8^. The structure–activity relationships of 1,8-naphthalimides with different modulations at various positions of the ring were earlier summarised by Tomczyk et al^9^. and, more recently, by Tandon et al^10^.

**Figure 1.**
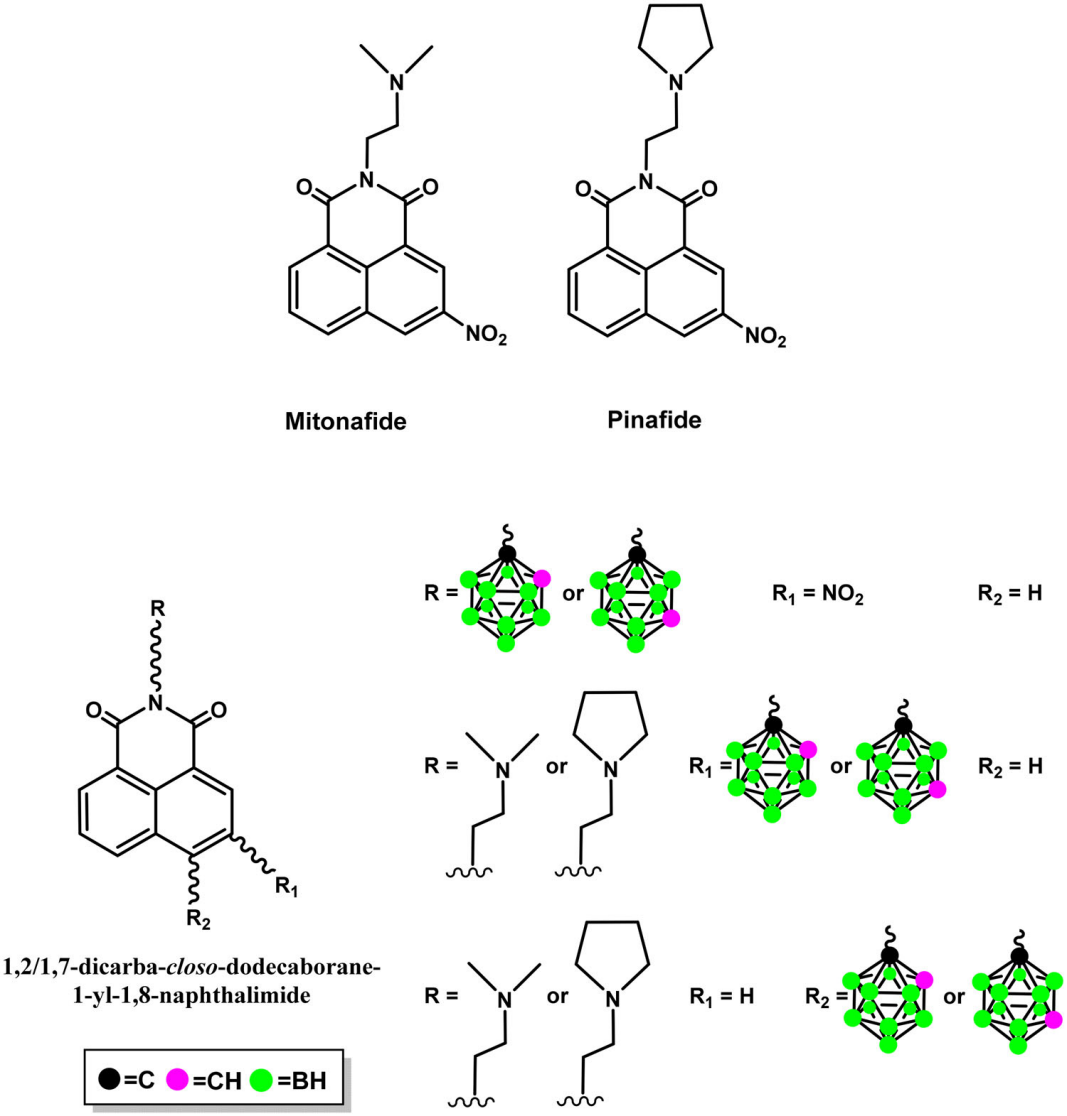
Structures of mitonafide, pinafide, and 1,8-naphthalimides modified with 1,2-dicarba-*closo*-dodecaborane or 1,7-dicarba-*closo*-dodecaborane^14–16^.

In our previous studies, the methods used to synthesise 1,8-naphthalimides modified with a carborane group or mitonafide and pinafide analogs, through modification of imide^11^, bearing the carborane group at position 3^12^ or 4 of the ring^13^, have been discussed (Figure 1). It was observed that the attachment site of carborane to the 1,8-naphthalimide moiety and the structure of the linker between the carborane cluster and the heteroaromatic ring system are the factors influencing various physicochemical (interaction with DNA) and biological (cytotoxicity, cell death, cell cycle, ROS production) properties of a HepG2 cancer cell line. Surprisingly, conjugates that interact most strongly with DNA were not as effective as Topo II inhibitors.

Several studies have reviewed the application of carboranes (isomers of dicarba-*closo*-dodecaborane) in medicinal chemistry, describing their derivatives as carriers for boron neutron capture therapy and as unique three-dimensional pharmacophores or modulators, thus indicating that carboranes are still an emerging class of compounds. Due to the distinct physical and chemical properties of carboranes, their derivatives with better or new anticancer, antibacterial, antiviral, and nematicidal activities, among others, have been designed^14–23^. The properties that are critical for the application of carboranes in medicinal chemistry are as follows: their ability to form unique noncovalent interactions with biological targets; spherical or ellipsoidal geometry and rigid 3 D arrangement to build 3 D constructions; lipophilicity, amphiphilicity, or hydrophilicity (depending on the type of the boron cluster used); adjustable pharmacokinetics, bioavailability, bio-orthogonality, and stability in biological environments; and a decreased susceptibility to metabolism^14^^,^^15^.

In this paper, based on the properties of carborane clusters useful in medicinal chemistry and the physicochemical and biological properties of carborane-1,8-naphthalimide conjugates we have described so far, a method for synthesising 1,8-naphthalimide derivatives, analogs of mitonafide and pinafide, modified with *ortho*-carborane (1,2-dicarba-*closo*-dodecaborane) or *meta*-carborane (1,7-dicarba-*closo*-dodecaborane) at position 4 and a nitro group at position 3 of the heteroaromatic skeleton is described, and their interaction with calf-thymus DNA (ct-DNA) and anticancer activity is discussed.

## Results and discussion

### Chemistry

#### Synthesis of mitonafide and pinafide analogs containing carborane clusters

**Scheme 1.**
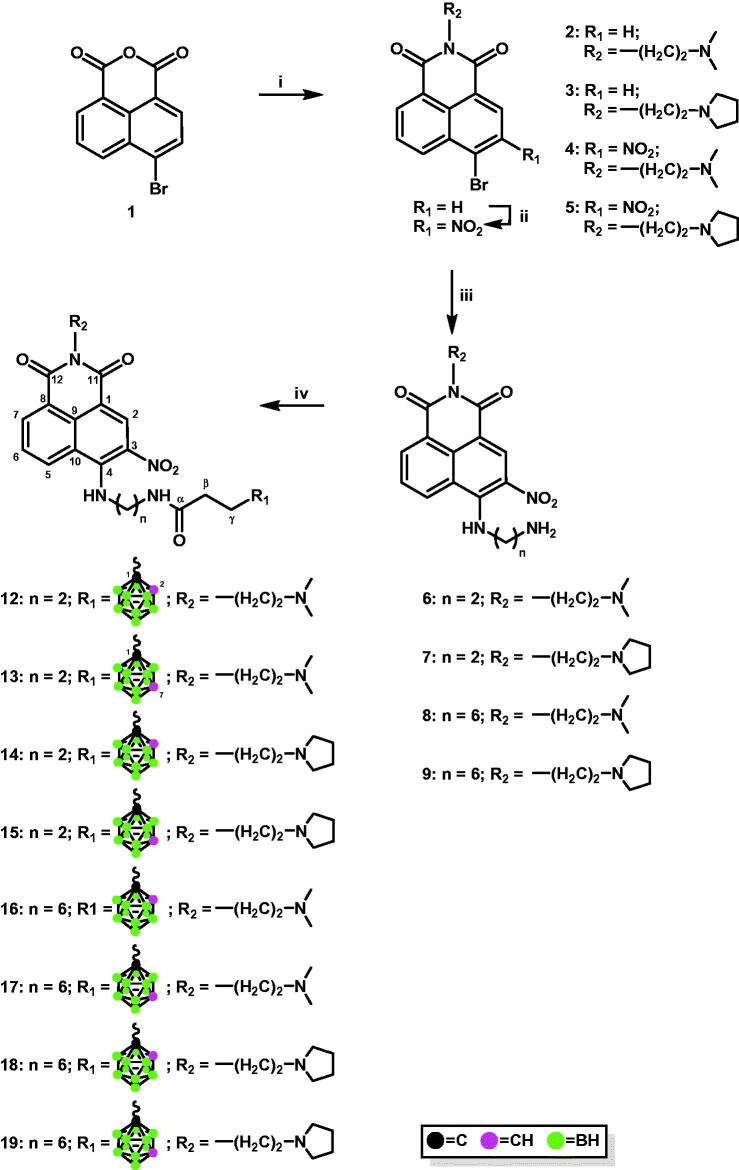
Modification of 4-carboranyl-3-nitro-1,8-naphthalimide derivatives with *ortho*-/*meta*-carborane via the formation of the amide bond. i. *N*,*N*-dimethylethylenediamine, EtOH_abs_, 2 h, 45 °C (for **2**); *N*-(2-aminoethyl)pyrrolidine, EtOH_abs_, 2 h, 45 °C (for **3**); ii. NaNO_3_, H_2_SO_4conc_, 1 h at 0 °C, 3 h at rt; iii. ethane-1,2-diamine, EtOH_abs_, 2 h, reflux (for **6** and **7**); hexane-1,6-diamine, EtOH_abs_, 2 h, reflux (for **8** and **9**); iv. 3–(1,2-dicarba-*closo*-dodecaboran-1-yl)propionic acid (**10**), PyBOP, TEA_anh_, CH_2_Cl_2anh_, 2–5 h, rt (for **12**, **14**, **16**, and **18**); 3–(1,7-dicarba-*closo*-dodecaboran-1-yl)propionic acid (**11**), PyBOP, TEA_anh_, CH_2_Cl_2anh_, 2–5 h, rt (for **13**, **15**, **17**, and **19**).

Appropriate carborane cluster acceptors **6**–**9** were obtained by the reaction of 4-bromo-*N*-[2-(dimethylamino)ethyl]-3-nitro-1,8-naphthalimide (**4**) or 4-bromo-3-nitro-*N*-[2-(*N*-pyrrolidinyl)ethyl]-1,8-naphthalimide (**5**) with ethane-1,2-diamine or hexane-1,6-diamine in absolute EtOH (Scheme 1). The products obtained were purified using column chromatography, and their structure was confirmed using ^1^H-NMR (Figures S1–S5, Electronic Supporting Information (ESI)).

DNA intercalators and Topo II poisons share three common essential structural features. The first one is a planar polyaromatic system (chromophore) which is sandwiched between DNA base-pairs. The second feature is a cationic species, interacting with the negatively charged phosphate group of DNA. The cationic centre may be an amino or nitrogen containing heterocyclic group, which can be protonated at physiological pH. The third feature is a groove binding side chain moiety, occupying the minor groove of DNA^24–26^.

Our previously synthesised 4-carboranyl-1,8-naphthalimides, mitonafide and pinafide analogs, with an amine linker between the carborane group and the naphthalimide moiety, were found to interact strongly with DNA, which indicated an intercalative binding mode. The same compounds were the most cytotoxic against HepG2 cells among all the modified conjugated tested.^13^ In the current work, these compounds were selected to be lead structures in the synthesis of new derivatives. Furthermore, studies revealed that specific substituent types on the naphthalic ring also might increased the cytotoxicity; e.g., 3-amino-, 3-nitro-, 3-methoxy groups gave the best results^9^.

**Scheme 2.**
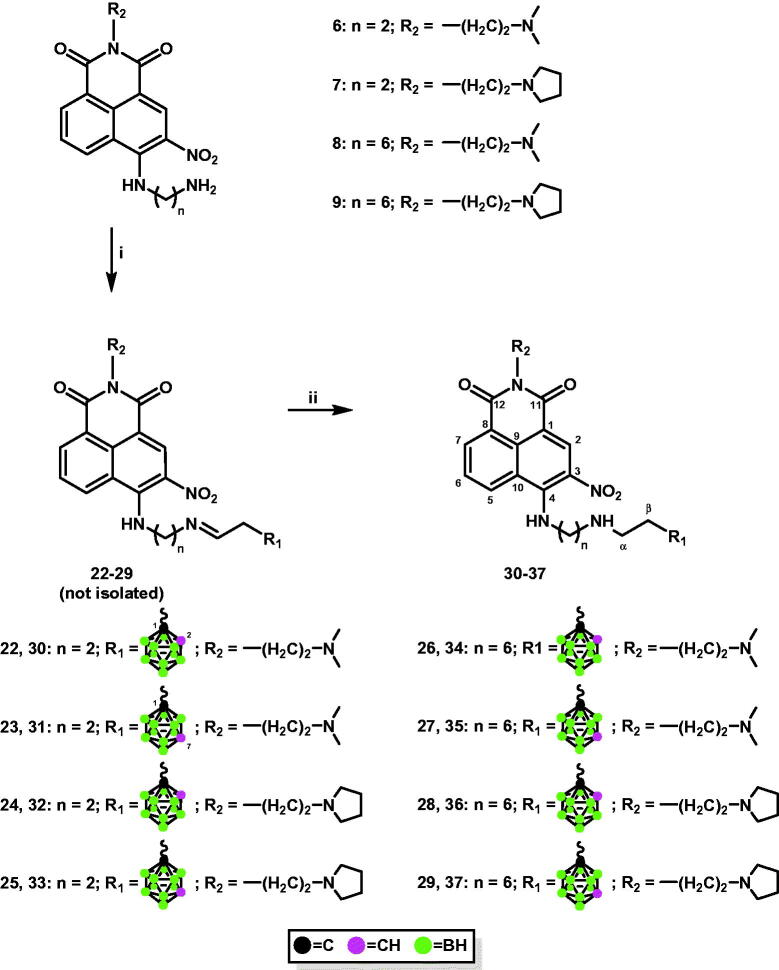
Synthesis of conjugates **30**–**37** via reductive amination. i. 2–(1,2-dicarba-*closo*-dodecaboran-1-yl)ethanal (**20**), MeOH_anh_, 24 h, 70 °C (for **22**, **24**, **26**, and **28**); 2–(1,7-dicarba-*closo*-dodecaboran-1-yl)ethanal (**21**), MeOH_anh_, 24 h, 70 °C (for **23**, **25**, **27**, and **29**); ii. NaBH_3_CN, 24 h, rt.

1,8-Naphthalimide derivatives, mitonafide and pinafide analogs, containing a carborane cluster at position 4 and a nitro group at position 3 of the heteroaromatic skeleton described in this study (compounds **12**–**19** and **30**–**37**, Schemes 1 and 2) were synthesised via (1) amidation reaction (Scheme 1 and 2) reductive amination (Scheme 2).

The formation of amide bonds is an immanent feature in organic and synthetic medicinal chemistry. Amides are the central building blocks in a number of interesting pharmaceuticals, proteins, peptides, polymers, natural products, functional materials, and biologically relevant carbocyclic or heterocyclic molecules and are also used in many industries^27^. In the present study, methods for synthesising 4-carboranyl-3-nitro-1,8-naphthalimide derivatives bearing *ortho*-/*meta*-carborane clusters **12**–**19** were developed based on the formation of an amide bond between naphthalimide and carborane using appropriate acids containing a carborane cluster as carborane cluster donors (Scheme 1). Briefly, 4-[(2-aminoethyl)amino]-*N*-[2-(dimethylamino)ethyl]-3-nitro-1,8-naphthalimide (**6**) or 4-[(2-aminoethyl)amino]-3-nitro-*N*-[2-(*N*-pyrrolidinyl)ethyl]-1,8-naphthalimide (**7**) or 4-[(6-aminohexyl)amino]-*N*-[2-(dimethylamino)ethyl]-3-nitro-1,8-naphthalimide (**8**), and 4-[(6-aminohexyl)amino]-3-nitro-*N*-[2-(*N*-pyrrolidinyl)ethyl]-1,8-naphthalimide (**9**) with 3–(1,2-dicarba-*closo*-dodecaboran-1-yl)propionic acid (**10**)^28^ or 3–(1,7-dicarba-*closo*-dodecaboran-1-yl)propionic acid (**11**)^29^ were dissolved in anhydrous CH_2_Cl_2_, and then anhydrous benzotriazol-1-yl-oxytripyrrolidinophosphonium hexafluorophosphate (PyBOP) and triethylamine (TEA) were added to the solution. Upon completion of the reaction, the crude products were purified using column silica gel chromatography (twice for compounds **16**, **18**, and **19**), following which conjugates **12**–**19** were obtained as orange solid in a moderate or good yield (32–73%). The structure, purity, and homogeneity of these compounds were confirmed using ^1^H-NMR, ^13^C-NMR, ^11^B-NMR, FT-IR, MS, RP-HPLC (Figures S6–S61, ESI), and TLC. 3-Carboranyl-1,8-naphthalimide derivatives were synthesised with good yields using the same methodology^12^, whereas 4-carboranyl-1,8-naphthalimide derivatives^13^ were synthesised using appropriate active esters bearing carborane clusters^16^. The methodology using active esters was unsuccessful for the synthesis of **12**–**19**. After 24 h, the desired product was not obtained.

Reductive amination is one of the key approaches to C–N bond construction because of its operational easiness and a wide-ranging toolbox of protocols. Recent studies have shown that at least one-fourth of C–N-bond-forming reactions in the pharmaceutical industry are performed via reductive amination^30^.

Treatment of 4-[(2-aminoethyl)amino]-*N*-[2-(dimethylamino)ethyl]-3-nitro-1,8-naphthalimide (**6**) or 4-[(2-aminoethyl)amino]-3-nitro-*N*-[2-(*N*-pyrrolidinyl)ethyl]-1,8-naphthalimide (**7**) or 4-[(6-aminohexyl)amino]-*N*-[2-(dimethylamino)ethyl]-3-nitro-1,8-naphthalimide (**8**), and 4-[(6-aminohexyl)amino]-3-nitro-*N*-[2-(*N*-pyrrolidinyl)ethyl]-1,8-naphthalimide (**9**) with aldehydes containing *ortho*-carborane (**20**) or *meta*-carborane (**21**)^31^ in MeOH at 70 °C for 24 h under an inert (Ar) atmosphere yielded the corresponding Schiff bases **22**–**29**. However, due to their instability, the resulting compounds could not be isolated (Scheme 2).

Conjugates **30**–**37** were synthesised by treating Schiff bases **22**–**29** with NaBH_3_CN followed by column chromatography. The structure, purity, and homogeneity of these compounds were confirmed using ^1^H-NMR, ^13^C-NMR, ^11^B-NMR, FT-IR, MS, RP-HPLC (Figures S62–S121, ESI), and TLC.

#### X-Ray structural analysis

**Figure 2.**
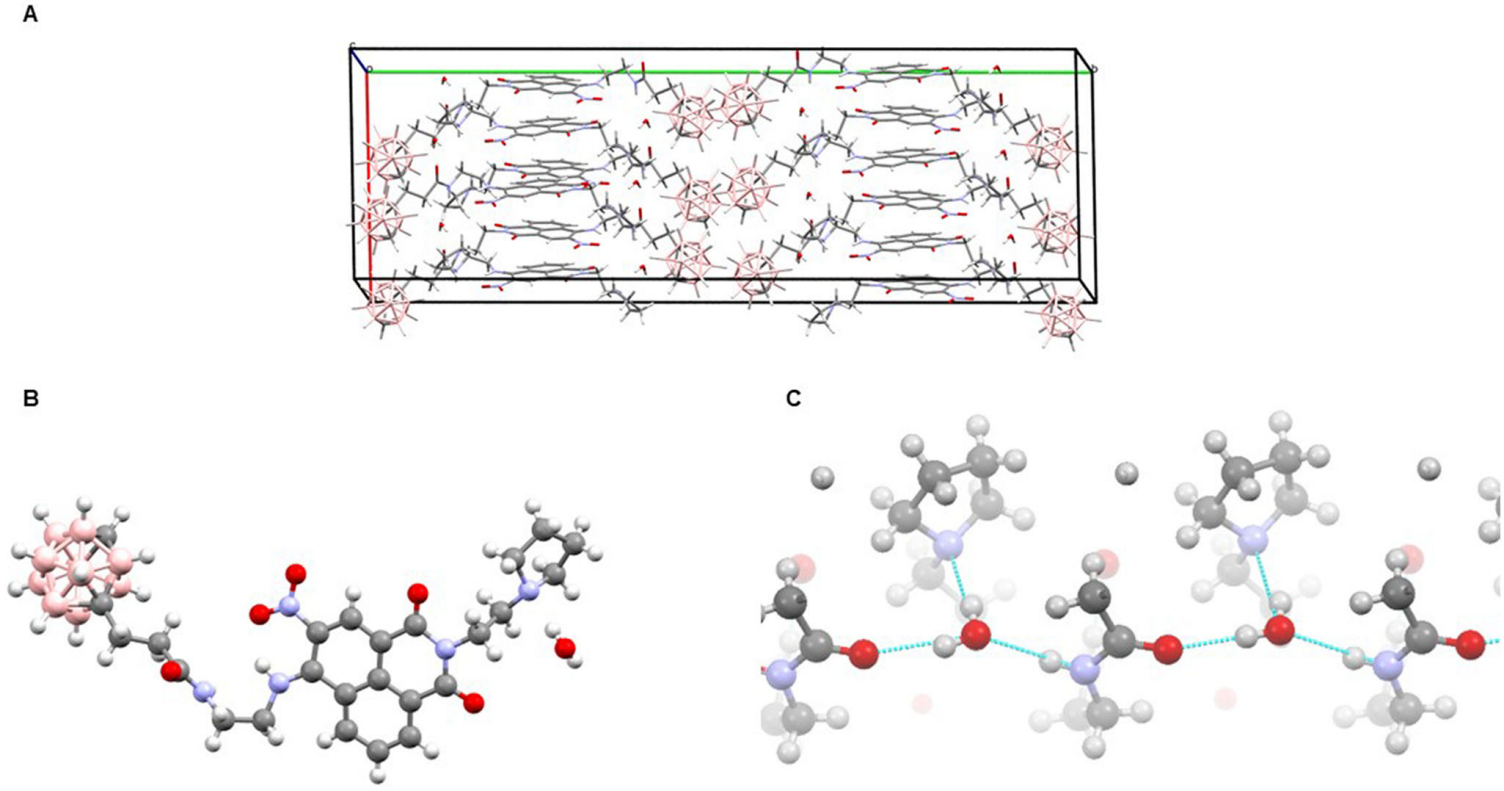
Crystallographic structure of **15**: (A) crystal packing within the unit cell, showing the distinct zones of rings stacking and carborane clustering. (B) A view of a single **15** molecule with an associated water molecule. (C) Some polar groups of **15** together with water molecules form an intermolecular hydrogen bond network; each water molecule links three neighbouring **15** molecules. Oxygen atoms are drawn red, nitrogen blue, carbon grey, boron pale pink, hydrogen white.

The crystal structure showed three molecules of **15** and three water molecules in the asymmetric unit, with similar structures and zipper-like arrangement. The 1,8-naphthalimide ring systems stacked extensively, in the antiparallel fashion, whereas the carborane clusters formed separate zones (Figure 2(A,B)). Intramolecular hydrogen bonding between the nitro group and the amine group adjacent to the naphthalene rings was observed. The bond stabilised the two groups to be nearly coplanar with the ring system. Clearly defined water molecules formed a network of hydrogen bonds, each water molecule linking three neighbouring **15** molecules via (1) the carbonyl oxygen atom of the amide group of one molecule, (2) the amine group of the amide of another molecule, and (3) the nitrogen atom of the pyrrole group of a third molecule (Figure 2(C)). The arrays of amide groups were oriented in the same direction, resulting in a clear electrostatic polarity for the crystal lattice.

### Physicochemical investigation with DNA

#### Melting temperature measurements

Small molecules can disturb the natural structure and dynamics of nucleic acids, and thus, they have potential applications in cancer therapeutics.

Melting temperature (*T*_m_) of DNA is defined as the temperature where half of the DNA population is melted—one half of the DNA is in the double-helical state and the other half is in a random coiled state^32^. An increase in the temperature transforms the double-stranded DNA into single strands by disrupting the hydrogen bonds between base pairs^33^. *T*_m_ depends on the length and sequence of the DNA. The majority of the DNA-binding small molecules known so far stabilise duplex DNA against heat denaturation. 1,8-Naphthalimides are valuable anticancer agents that stack between the DNA bases and exhibit stabilising effects depending on the substituent attached to the naphthalimide, the position of the substituent, and the sequence of DNA. In some cases, the 4-substituents have exhibited different trends than the 3-substituents^34^.

In the present study, *T*_m_ was measured to estimate the impact of the obtained compounds **12**–**19** and **30**–**37** on DNA stabilisation (Table 1, and Figures S122–S125, ESI).

**Table 1.** Δ*T*_m_ and binding constant (K*_b_*) based on UV–vis of ct-DNA in the presence of 1,8-naphthalimide–carborane conjugates.

Compound	Δ*T*_m_ [°C]	K*_b_* [M^−1^]
	ct-DNA	
**12**	3.33	2.21 ± 0.33 × 10^5^
**13**	3.67	1.58 ± 0.63 × 10^5^
**14**	3.33	2.22 ± 0.90 × 10^5^
**15**	5.00	2.26 ± 0.92 × 10^5^
**16**	0.00	4.02 ± 0.83 × 10^5^
**17**	0.00	2.24 ± 1.45 × 10^5^
**18**	−0.67	4.10 ± 1.15 × 10^5^
**19**	−5.00	1.06 ± 0.31 × 10^5^
**30**	3.33	3.25 ± 0.53 × 10^5^
**31**	1.67	2.26 ± 0.44 × 10^5^
**32**	0.33	4.26 ± 0.51 × 10^5^
**33**	0.33	nd
**34**	11.67	1.42 ± 0.40 × 10^5^
**35**	13.00	4.50 ± 1.68 × 10^5^
**36**	10.00	2.91 ± 0.49 × 10^5^
**37**	13.00	1.69 ± 1.13 × 10^5^

nd: not deteremined.

Thermal melting experiments indicated that the studied conjugates obtained via the amidation reaction (**12**–**19**) showed weaker DNA stabilisation compared with the conjugates synthesised via reductive amination (**30**–**37**). In the first group of compounds, **18** and **19** destabilised ct-DNA, whereas **12**–**15** stabilised ct-DNA with Δ*T*_m_ values = 3.33–5.00 °C. Conjugates **16** and **17** showed no effect on the tested DNA. In the second group of compounds, all conjugates stabilised ct-DNA, although derivatives **30**–**33** with a shorter linker between carborane modification and the 1,8-naphthalimide residue stabilised ct-DNA more weakly than compounds **34**–**37** with a (CH_2_)_6_ linker. The thermal denaturation experiment conducted for ct-DNA in the absence of the modified 1,8-naphthalimide revealed a *T*_m_ value of 74 °C. In contrast, in the presence of **34**–**37**, the *T*_m_ of ct-DNA significantly increased within the range of values 84–87 °C (Δ*T*_m_ = 10.00–13.00 °C, Table 1) with derivatives **35** and **37** bearing *meta*-carborane showing the highest value. In comparison with unmodified mitonafide and pinafide (Δ*T*_m_ = 5.17 and 6.50 °C, respectively)^12^, this may indicate an intercalative interaction with DNA as the dominant binding mode. In addition, conjugates **35** and **37** stabilised ct-DNA most strongly among all 1,8-naphthalimides containing carborane clusters obtained in our laboratory^12^^,^^13^: the Δ*T*_m_ values of selected 4-carboranyl-1,8-naphthalimides increased within the range of 81.67–86.33 °C (Δ*T*_m_ = 7.67–12.33 °C), also suggesting their intercalation with DNA^13^.

#### Circular dichroism (CD) spectra measurements

For an in-depth understanding of the interactions of the modified 1,8-naphthalimides **12**–**19** and **30**–**37** with DNA, CD measurements were conducted. The characteristic right-handed B-form CD spectra of the DNA in the 200–300 nm region can provide information relevant to the structural change of the DNA on interaction with ligands. The CD spectra of free DNA exhibited a negative peak around 245 nm due to helicity and a positive peak around 270 nm due to base stacking^35^. As described earlier, treatment of the DNA with mitonafide and pinafide caused a decrease in the negative peak and an increase in the positive peak^12^. As illustrated in Figures S126, S128-S131 (ESI), an increase in the concentration of conjugates bearing a carborane cluster **12**, **13**, **16**–**19**, **31** and **33** did not cause any appreciable change in the CD spectra of ct-DNA. In the presence of conjugates **14**, **15**, **30**, **32**, and **36**, the positive and negative bands of ct-DNA were disturbed (Figures S127, S130, S131, and S133, ESI). Simple groove binding and electrostatic interaction of ligands caused less or no disturbance to the base-stacking and helicity bands, whereas the intensities of both positive and negative bands were affected in the intercalative mode of binding^36^. In contrast, CD spectra of ct-DNA were remarkably disturbed in the presence of conjugates **34**, **35**, and **37** (Figures S132 and S133, ESI), resulting in the increase and decrease in the positive and negative bands, respectively, without any shift in the band positions. Both effects are consistent with the intercalative properties of the compounds. No conformational change from the B-form structure of DNA was observed. The obtained data for those compounds are in good agreement with their melting curve results.

#### UV–vis spectra measurement

Similar to CD spectroscopy and thermal denaturation studies, UV–vis absorption spectroscopy is also one of the simplest and most commonly used techniques for studying the stability of DNA and its interactions with small ligand molecules. Drug–DNA interactions can be studied using UV–vis absorption spectroscopy by monitoring the changes in the absorption properties of the drug or the DNA. Whether there is any interaction between the DNA and the drug can therefore be easily examined by the shifting of the position of the maximum of this band when the ligand is free in the solution and when the ligand is bound with DNA^37^. Compounds binding with DNA through intercalation usually exhibit hypochromism and bathochromism. In the case of an electrostatic attraction between the compound and the DNA, a hyperchromic effect is observed, which reflects the corresponding changes of the DNA in its conformation and structure after the complex–DNA interaction has occurred^37^.

The spectral changes observed in the electronic absorption of **12**–**19** and **30**–**37** in the absence and the presence of ct-DNA are illustrated in Figures S134–S148 (ESI). The progressive addition of ct-DNA at the concentration of 1.25–15 µM to a fixed amount of 1,8-naphthalimide (10 µM concentration) decreased the absorbance for almost all the tested modified conjugates, except for conjugate **33**, for which changes in the absorbance were not observed. For conjugates **12**–**19**, **30**, and **32**, a slight bathochromic effect (2–4 nm) was observed. In contrast, the bathochromic effect was observed for conjugates **31** (6 nm), **34** (9 nm), **35** (10 nm), **36** (7 nm), and **37** (8 nm). Hypochromicity was observed for **12**–**19**, **30**–**32**, and **34**–**37** (10–53%), with compound **13** showing the highest value.

To compare the DNA-binding strength of the tested conjugates, the binding constant K*_b_* was calculated (Table 1) as described in the Materials and Methods section. The K*_b_* values of compounds **12**, **14**, **15**, **17**, **31**, and **36** were similar to that of mitonafide (2.54 × 10^5^)^12^. Derivatives **16**, **18**, **32**, and **35** showed approximately 1.5-fold higher K*_b_* values compared to that of mitonafide. The K*_b_* value could not be determined for conjugate **33** due to the lack of noticeable changes in the UV spectra.

### Biological Investigation

#### *In vitro* cytotoxic activity

The *in vitro* cytotoxic activity of the obtained carborane-1,8-naphthalimide conjugates was investigated by examining their cytotoxic effects using the MTT (3–(4,5-dimethylthiazol-2-yl)-2,5-diphenyltetrazolium bromide) dye assay^38^^,^^39^ against the human cancer cell line HepG2 established from hepatocellular carcinoma (for compounds **12**–**19** and **30**–**37**) and against human lung fibroblasts (MRC-5) (for compounds **34**–**37**). The IC_50_ values—the drug concentration (µM) required to inhibit cell growth by 50%—determined for the synthesised compounds are summarised in Table 2.

**Table 2.** Cellular cytotoxic activity of compounds **12**–**19** and **30**–**37**.

Compound	IC_50_^a^[µM]	
	HepG2	MRC-5
**12**	21.71 ± 0.95	nd
**13**	9.20 ± 0.68	nd
**14**	8.97 ± 1.13	nd
**15**	9.23 ± 0.35	nd
**16**	5.12 ± 0.87	nd
**17**	8.03 ± 1.25	nd
**18**	4.79 ± 0.80	nd
**19**	5.86 ± 0.56	nd
**30**	4.51 ± 0.66	nd
**31**	11.59 ± 0.62	nd
**32**	8.12 ± 0.88	nd
**33**	20.60 ± 3.83	nd
**34**	4.78 ± 0.79	2.13 ± 0.23
**35**	4.32 ± 0.71	2.14 ± 0.36
**36**	3.63 ± 0.30	2.33 ± 0.02
**37**	4.23 ± 0.82	2.26 ± 0.05

^a^Concentration of the compound required to inhibit cell growth by 50%.

Mitonafide IC_50_ < 1 µM, pinafide IC_50_ = 1.23 ± 0.15^11^.

nd: not determined.

Generally, mitonafide derivatives were less cytotoxic than the modified pinafide derivatives, and conjugates modified with *meta*-carboranes were less active than the corresponding conjugates modified with *ortho*-carborane. A comparison of the activity of the 1,8-naphthalimides in the series showed that the conjugates obtained via amidation reaction **12**–**19** were slightly less cytotoxic than the modified 1,8-naphthalimides obtained using the reductive amination reaction **30**–**37**. Recently, we have reported that 4-carboranyl-1,8-naphthalimides synthesised using the reductive amination reaction were more cytotoxic against the HepG2 cell line than the conjugates obtained via the “click reaction,” Mitsunobu reaction, or amidation reaction^13^. Among compounds **12**–**19** and **30**–**37**, the conjugates containing a shorter linker (CH_2_)_2_ between the heteroaromatic system and carborane modification (**12**–**15** and **30**–**33**) were less cytotoxic than those containing a longer linker (CH_2_)_6_ between the heteroaromatic system and carborane (**16**–**19** and **34**–**37**). The highest cytotoxicity to the tested cell line was shown by the pinafide analog **36** containing *ortho*-carborane, at a concentration as low as 3.63 µM, whereas the pinafide analog **37** containing *meta*-carborane was less cytotoxic, with an IC_50_ value of 4.23 µM. The mitonafide analogs bearing *ortho*-carborane **34** (IC_50_ = 4.78 µM) or *meta*-carborane **35** (IC_50_ = 4.32 µM) were slightly less cytotoxic than their pinafide analogs. Furthermore, conjugates **34**–**37** were more active than the mitonafide and pinafide analogs also obtained through reductive amination but containing a carborane cluster at position 3 of the heteroaromatic system (IC_50_ = 4.77–53.09 µM)^12^ and less active than 4-carboranyl-1,8-naphthalimide derivatives^13^. The conjugates **34**–**37** were slightly more cytotoxic against the MRC-5 cell line (IC_50_ = 2.13–2.33 µM) than against HepG2.

Considering the physicochemical and cytotoxic properties of conjugates **34**–**37**, they were used for further biological studies.

#### Apoptosis/necrosis assay by flow cytometry

Anticancer agents induce cancer cell death primarily through apoptosis or necrosis. As anticancer agents cause apoptosis and necrosis at low and high concentrations, respectively, cancer cells may merely be injured by an anticancer agent in apoptosis and their death may result from the activation of the internal constituents to induce apoptosis^38^.

**Figure 3.**
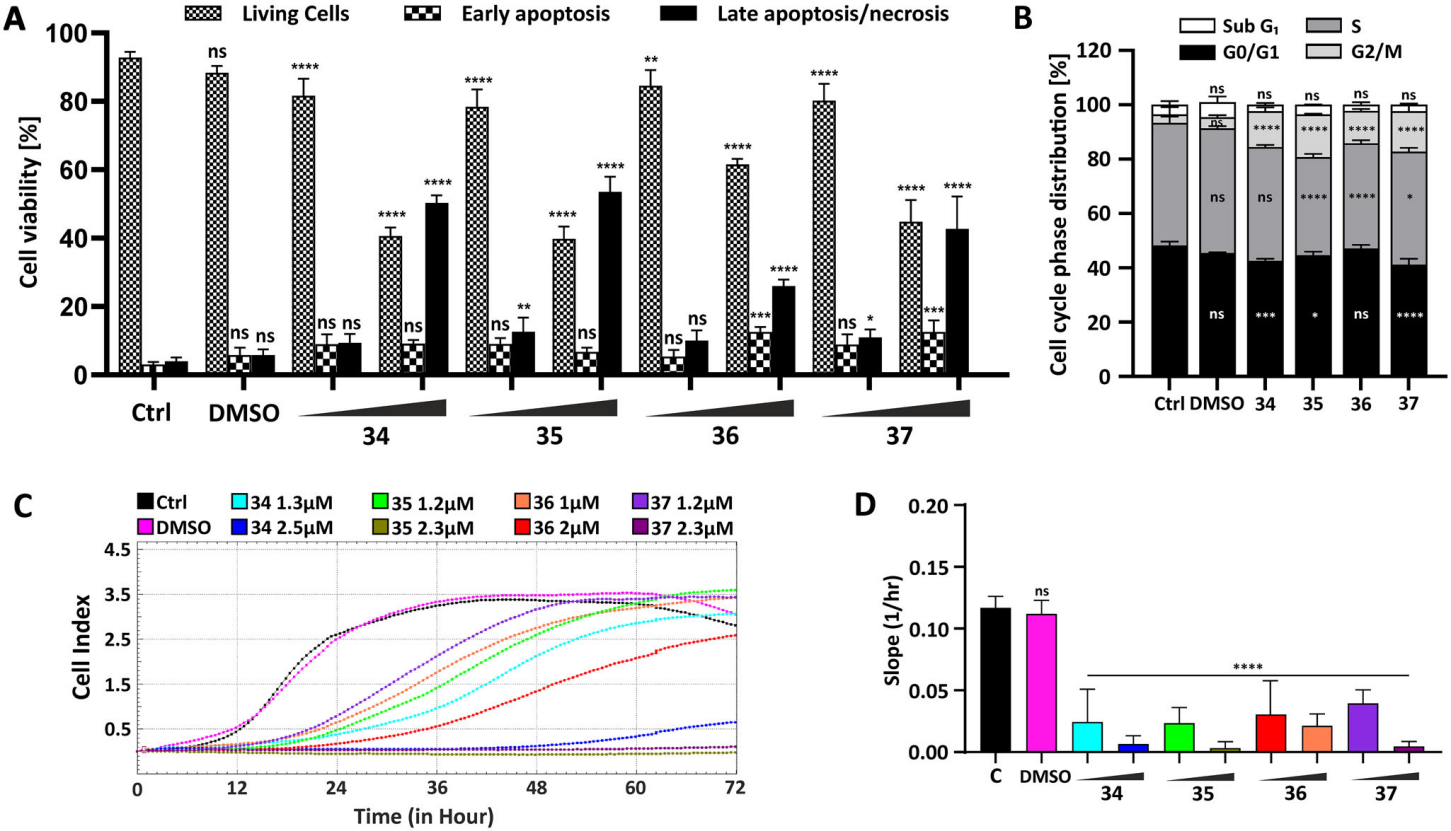
Influence of compounds **34**–**37** on cellular viability, migration, and cell cycle progression. (A) Flow cytometry analysis of apoptosis induction by the analysed compounds in HepG2 cells after 24-h treatment with compounds **34**–**37** in a concentration corresponding to the half (**34**–2.5 µM, **35**–2.3 µM, **36**–2 µM, **37**–2.3 µM) and the total (**34**–5 µM, **35**–4.5 µM, **36**–4 µM, **37**–4.5 µM) IC_50_ value using YO-PRO 1/PI dual staining. The number of live and apoptotic and necrotic cells was evaluated for each compound after 24 h and presented as a bar graph. The data are presented as a mean percentage ± SD from three independent experiments. (B) Cell cycle phase distribution in HepG2 cells treated for 24 h with the compounds in a final concentration: **34**–5 µM, **35**–4.5 µM, **36**–4 µM, **37**–4.5 µM. Percentage cell distribution in each of the cell cycle phases was calculated and presented as a mean ± SD from three independent experiments. (C) Real-time migration potential in HepG2 cells treated with compounds **34**–**37**. The inhibitory potential of the compounds was analysed using the xCELLigence system in a concentration corresponding to the half (**34**–2.5 µM, **35**–2.3 µM, **36**–2 µM, **37**–2.3 µM) and one-fourth (**34**–1.3 µM, **35**–1.2 µM, **36**–1 µM, **37**–1.2 µM of their IC_50_ value. The rate of cellular migration was monitored every 30 min for 72 h. (D) Impedance-based signals were recorded, and the slope parameter representing the rate of changes in CI values was calculated for HepG2 cells within the first 24 h. Statistical significance is indicated with asterisks: (ns) *p* > 0.05, *p < 0.05, **p < 0.01, ***p < 0.001, ****p < 0.0001.

To investigate whether conjugates **34**–**37** induced apoptosis in HepG2 cells, the cells were incubated with the conjugates for 24 h, and a flow cytometry analysis was carried out. The concentration corresponding to the half and total IC_50_ values was chosen for each compound (**34**–2.5; 5 µM, **35**–2.3; 4.5 µM, **36**–2; 4 µM; **37**–2.3; 4.5 µM). After incubation, the cells were stained by YO-PRO1/PI dual staining. All the studied compounds induced early apoptosis in HepG2 after 24-h treatment; however, the rate of this type of cell death was not the prominent one (Figure 3(A), Figure S149, ESI). The highest percentage of early apoptotic cells was observed in HepG2 cells treated with compounds **36** and **37** (12.55 and 12.56%, respectively). Compounds **34** and **35** caused apoptotic changes only in 9.12 and 6.7% of cells. This suggests that apoptosis is not the dominant cell death process, while the most significant fluorescent signal was observed from necrotic cells (PI positive). The highest percentage of necrosis was found in HepG2 cells treated with compound **35** (55.75%).

In studies of 1,8-naphthalimide derivatives containing boron clusters, the majority of the conjugates promoted the apoptosis mode of cell death. Some derivatives triggered autophagy or ferroptosis^11^^,^^13^.

#### Cell cycle analysis by flow cytometry

Cell cycle involves coordinated events that result in the formation of two cells from one mother cell. It comprises four major phases: G1 (growth phase 1), S (DNA synthesis phase), G2 (growth phase 2), and M (mitosis phase). These phases function to integrate environment-sensing signalling pathways with cell growth and proliferation^39^. Cancer cells often deregulate the cell cycle and undergo unscheduled cell divisions; therefore, inhibition of the cell cycle presents an opportunity for therapeutic intervention in treating proliferative diseases like cancer^40^. Most of the anticancer drugs disturb the proliferation cycle of tumour cells by inhibiting/damaging cell cycle events, which activate checkpoints, arrest cells, and induce apoptosis^41^.

To understand the mechanism underlying the inhibitory effect of the carborane-1,8-naphthalimide conjugates on cellular viability, cell cycle regulation was studied. For this purpose, HepG2 cells were exposed to compounds **34**–**37** (**34** (4.78 µM), **35** (4.32 µM), **36** (3.63 µM), and **37** (4.23 µM)). The concentration chosen for each of these compounds corresponded to their total IC_50_ value. After exposure, the HepG2 cells were examined using flow cytometry, and their DNA content was measured via PI staining. Based on the DNA content, it was observed that the tested compounds affected the cell cycle progression, resulting in G2M phase arrest. Compounds **35** and **37** showed the greatest impact, in which the observed accumulation of the cells in this phase was the most prominent (15 vs 5% in untreated cells) (Figure 3(B), Figure S150, ESI).

In comparison, unmodified mitonafide and pinafide induced cell cycle arrest at the S and G2M phases, respectively^11^. The studied 1,8-naphthalimide derivatives containing carborane group at the *N*-imide position caused cell cycle arrest at the G0/G1 phase^11^, 3-carboranyl-1,8-naphthalimides halted the cell cycle at the G2M phase^12^, and 4-carboranyl-1,8-naphthalimides affected the cell cycle at the G0/G1 or G2M phases^13^.

#### Cell migration inhibition assay

Cell migration is fundamental to establishing and maintaining the proper organisation of multicellular organisms. In an adult organism, cell migration is essential for proper immune response, wound repair, and tissue homeostasis, and aberrant cell migration results in various pathologies^42^.

Using the xCELLigence platform, the migration potential of HepG2 cells after the administration of compounds was evaluated. The cells were incubated with compounds **34**–**37** at the concentration corresponding to one-fourth and half of their IC_50_ value for 24 h (**34**–1.3; 2.5 µM, **35**–1.2; 2.3 µM, **36**–1; 2 µM, **37**–1.2; 2.3 µM) before the analysis and were then seeded on a cell invasion and migration (CIM) plate. Impedance-based cell index (CI) of the HepG2 cells was measured every 30 min for 72 h. Control cells were grown only in a culture medium. To eliminate potentially misleading solvent effects, the cells were incubated only with DMSO (Figure 3(C,D)). The rate of migration was determined by calculating the slope parameter representing the changes in the CI within the first 24 h of the analysis (Figure 3(D)). The obtained data clearly showed that all of the analysed compounds affected the migration of HepG2 cells compared with control. Conjugates **34**, **35**, and **37** showed the highest influence on the migration activity. However, mitonafide analog **35** containing a *meta*-carborane cluster showed the highest impairment (Figure 3(D)).

#### Oxidative stress measurement in HepG2 cells by flow cytometry

ROS, a group of ions and molecules, include hydroxyl radicals, alkoxyl radicals, superoxide anion, singlet oxygen, and hydrogen peroxide. Endogenous ROS are mainly formed in the mitochondrial respiratory chain. At low levels, ROS play important roles in the regulation of biological functions in mammalian cells, whereas excess production of ROS can induce cell death due to their oxidative damaging effects on intracellular biomacromolecules. The types of cancer cell death induced by ROS are apoptotic, autophagic, ferroptotic, and necrotic cell death. Some anticancer drugs currently used in clinics, such as molecular targeted drugs and chemotherapeutic agents, effectively kill cancer cells by inducing ROS generation^43^. A previous study showed that 1,8-naphthalimide derivatives could trigger apoptosis via activating the ROS-p38 MAPK pathway^44^ and induce apoptosis signal transduction through the ROS/lysosomal/mitochondrial pathway^4^.

**Figure 4.**
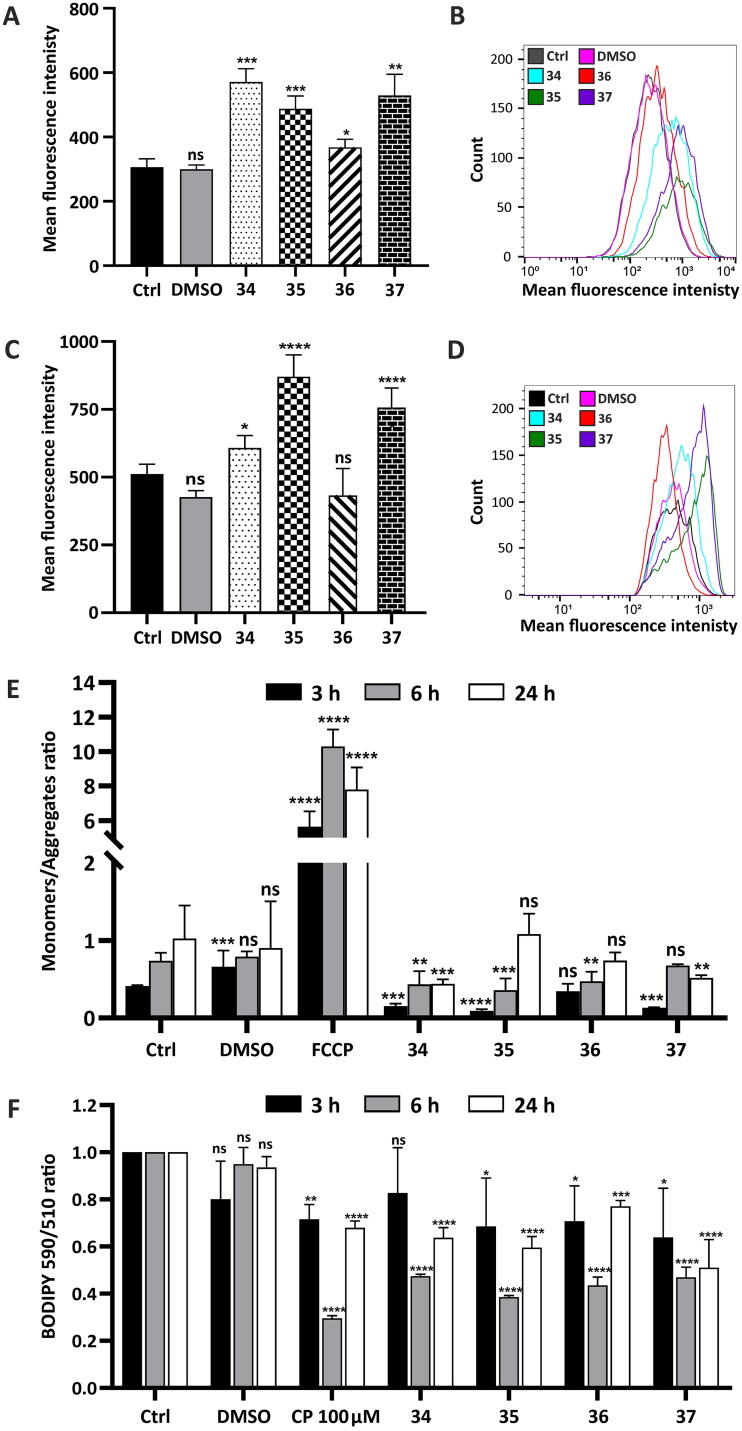
Bioenergetic and metabolic impairment in HepG2 cells treated with compounds **34**–**37**. (A–E) Analysis of oxidative stress parameters in HepG2 cells treated with the analysed compounds for 24 h in a following concentration: **34**–5 µM, **35**–4.5 µM, **36**–4 µM, **37**–4.5 µM. ROS production (A and B) was investigated using a FACSCalibur flow cytometer with H_2_DCFDA/PI double staining in a final concentration 0.5 µM and 10 µM/mL respectively. Antioxidant defense system (C and D) was investigated using Thiolite Green sensor. (A) Green fluorescence intensity was analysed (∼ 510/525 ex/em), and the data are shown as a bar graph of the three independent experiments (mean ± SD) or as representative histograms (B). (C) Flow cytometry analysis of GSH using Thiolite Green dye (50 µM). The GSH level was estimated, and the fluorescence intensity changes are presented as a bar graph (mean ± SD) from three replicates or as representative histograms (D). (E) Flow cytometry mitochondrial membrane potential (MMP) analysis using JC-1 fluorescent dye (final concentration 2.5 µM). Green fluorescence emitted by monomers of the dye and red fluorescence corresponding to its aggregates were counted and presented as a monomers/aggregates ratio on a bar graph from three replicates after 3, 6, and 24 h of incubation. FCCP (3 µM) emitting a strong green fluorescent signal was used as a negative control. (F) Detection of lipid peroxidation estimated via fluorescent BODIPY 581/591 C11 reagent (10 µM). Upon oxidation, a fluorescence shift from red to green was measured after 3, 6, and 24 h of incubation with the analysed compounds in a concentration that corresponded to their IC_50_ value and presented as a 590/510 ratio on bar graphs. As a positive control, 100 µM cumene hydroxyperoxide (CP) for 2 h was used. Statistical significance is indicated with asterisks: (ns) *p* > 0.05, *p < 0.05, **p < 0.01, ***p < 0.001, ****p < 0.0001.

To determine the effect of compounds **34**–**37** on the HepG2 cells, the oxidative status of the cells was analysed after 3, 6, and 24 h of treatment as the pathological level of ROS affects the proper functioning of biomacromolecules. The compounds were added to the growth medium at a concentration that corresponds to their total IC_50_ value (**34** (4.78 µM), **35** (4.32 µM), **36** (3.63 µM), and **37** (4.23 µM)). The ability to generate ROS in the HepG2 cells was investigated using a FACSCalibur flow cytometer, via H_2_DCFDA staining. Negligible ROS production after 3 and 6 h of treatment with conjugates **34**–**37** was observed (Figure S151, ESI). After 24 h, flow cytometry analysis was performed which indicated that the most potent inductors of ROS were compounds **34** and **37** as the green fluorescence intensity in HepG2-treated cells increased approximately twofold. The weakest ROS inductor was compound **36**. Compound **35** showed a moderate effect on ROS generation after 24-h treatment (Figure 4(A,B)).

In the series of 1,8-naphthalimides bearing boron clusters we have reported so far, the most potent inducers of intracellular ROS production were *N*-imide derivatives. The most promising ones were *N*-[3–(1,2-dicarba-*closo*-dodecaborane-1-yl)propyl]-1,8-naphthalimide, *N*-[2–(3,3′-commo-bis(1,2-dicarba-3-cobalta(III)-*closo*-dodecaborate-1-yl)ethyl)]-1,8-naphthalimide, and *N*-{[2–(3,3′-commo-bis(1,2-dicarba-3-cobalta(III)-*closo*-dodecaborate-1-yl)ethyl]-1′-aminoethyl)}-1,8-naphthalimide] with an approximately twofold increase in fluorescence intensity^11^.

#### Reduced glutathione (GSH) level measurement

GSH is a tripeptide compound composed of glutamate, cysteine, and glycine. It plays a role in many cell life activities involved in the occurrence and development of various human diseases, including cardiovascular diseases, ageing diseases, and diabetes. GSH is one of the major physiological free radical scavengers and can effectively remove excess ROS as well as endogenous and exogenous electrophilic species to protect cells^45^.

To confirm the ability of conjugates **34**–**37** to generate ROS, GSH level in HepG2 cells was determined by flow cytometry using Thiolite Green dye, whose fluorescence is enhanced upon reaction with thiol-containing compounds. The cells were incubated with compounds **34**–**37** in a final concentration corresponding to their total IC_50_ value (**34** −5 µM, **35**–4.5 µM, **36**–4 µM, **37**–4.5 µM). After 24 h, an increase in the intensity of green fluorescence was observed. The highest change was observed in the cells treated with compound **35** (70% higher compared to control), whereas the slightest change was observed in the cells treated with compound **36** (not statistically significant) (Figure 4(C,D)). These changes were in correlation with the oxidative stress induced by the compounds, and this forced natural antioxidant defense system activation.

#### MMP measurement

A strong positive correlation between MMP (ΔΨ) and ROS production has been repeatedly demonstrated in different experimental models^46^.

In this study, mitochondrial functions in the HepG2 cells treated with the analysed compounds **34**–**37** were analysed. Final concentrations of the compounds were as follows: **34**–5 µM, **35**–4.5 µM, **36**–4 µM, **37**–4.5 µM. To follow the changes in the cells over time and find out whether they correlated with our previous observations, the same experimental conditions as above were chosen (3, 6, and 24 h of treatment). MMP was analysed by flow cytometry using JC-1 fluorescent dye. A high MMP allows the dye to aggregate, emitting red fluorescence. In the case of a low MMP, the dye is present in mitochondria as monomers, emitting green fluorescence. To create a strong green signal, 3 µM FCCP was used for 15 min. Results revealed that the analysed compounds affected the MMP, leading to its hyperpolarization, which is shown as a monomers/aggregates ratio (Figure 4(E)). The highest changes were observed in the HepG2 cells after 3-h treatment with compound **35** (90.8% aggregates vs 69.9% in control cells, Figure S152, ESI). Further incubation did not decrease the potential, after neither 6 h nor 24 h.

#### Lipid peroxidation measurement

Cell membranes are sensitive to free radical damage as they contain polyunsaturated fatty acids. Another main effect of ROS is lipid peroxidation, which occurs when membrane phospholipids come into contact with an ROS oxidising agent. In this reaction, the free radical oxidises an unsaturated lipid chain, leading to the formation of a hydroperoxidized lipid and an alkyl radical. This process of lipoperoxidation results in alterations of the membrane structure, thus affecting its fluidity and damaging its integrity^47^. Lipid peroxidation is also the central biochemical and metabolic event leading to plasma membrane damage during ferroptosis^48^.

The induction of lipid peroxidation in HepG2 cells after 3, 6 and 24 h of treatment with the tested compounds was studied. The cells were treated with compounds **34**–**37** in a concentration that corresponded to their total IC_50_ value (**34** −5 µM, **35**–4.5 µM, **36**–4 µM, **37**–4.5 µM). Peroxidation of lipids was estimated by flow cytometry using the BODIPY 581/591 C11 reagent, which displays a shift in emitted fluorescence. As a positive control, 100 µM CP was used for the last 2 h of the experiment. Results revealed that all of the analysed compounds caused peroxidation of lipids, which started within the first 3 h of incubation and increased over time. The tested compounds affected the HepG2 cells similarly, causing extensive fluorescence shift expressed as a 590/510 ratio (Figure 4(F)).

#### Intracellular Fe^2+^ level determination

Lipid peroxidation observed in HepG2 upon incubation with compounds **34**–**37** prompted us to analyse the iron level of the compounds taking into account their ability to induce ferroptosis. Ferroptosis is a new type of cell death discovered recently and is usually accompanied by a large amount of iron accumulation and lipid peroxidation during the cell death process^49^. Activating or blocking the ferroptosis pathway to alleviate the progression of the disease has proved to be a promising therapeutic strategy for many diseases. As a new type of programmed cell death, ferroptosis has been increasingly used in the treatment of many cancers with clinical drugs and experimental compounds^50^.

**Figure 5.**
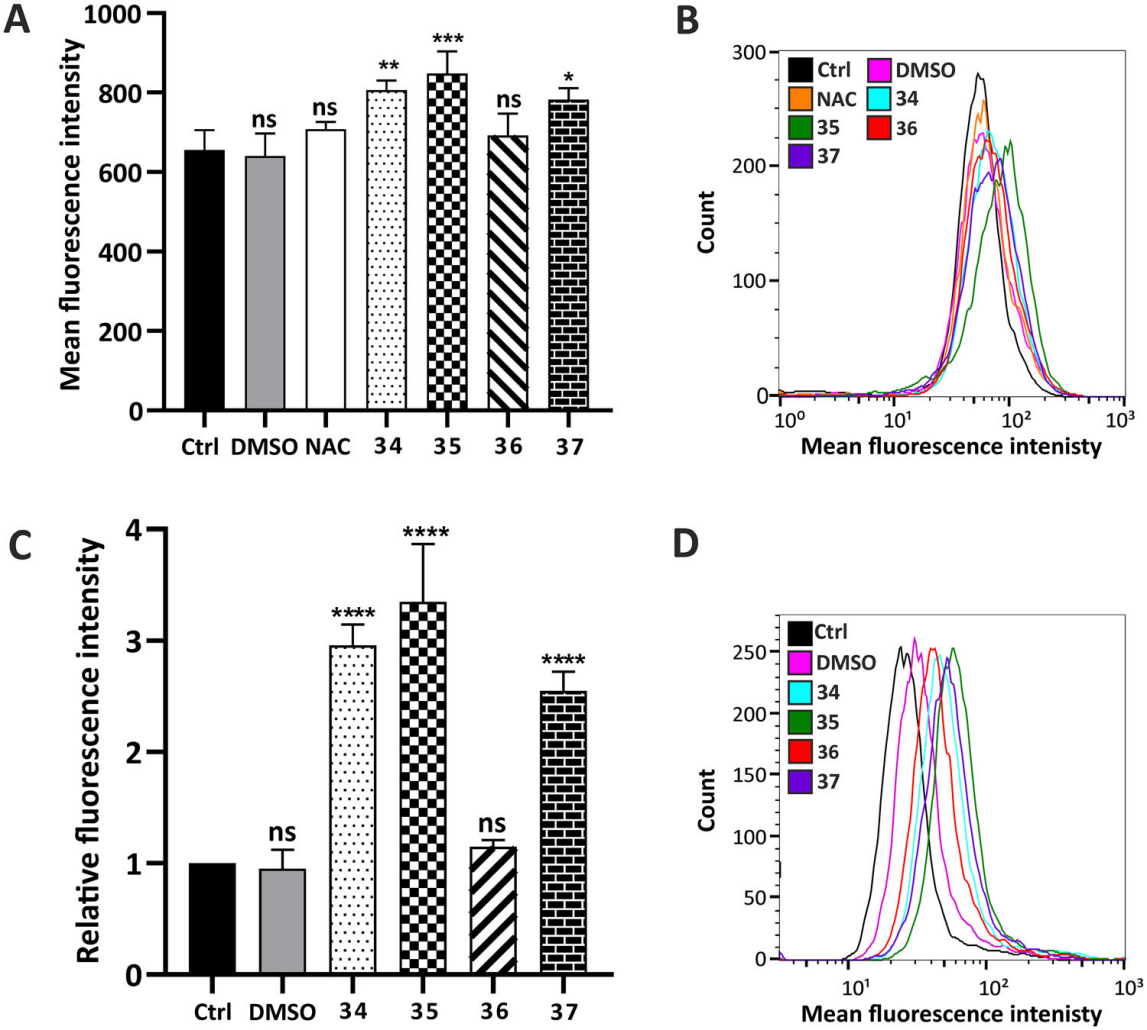
Hallmarks of ferroptosis in HepG2 cells induced by compounds **34**–**37**—intracellular Fe^2+^ level determination and Ca^2+^ homeostasis determination. (A and B) Fe^2+^ content was determined using a FeRhoNox-1 fluorescent probe (5 µM) after 24-h incubation with the analysed compounds (final concentration: **34**–5 µM, **35**–4.5 µM, **36**–4 µM, **37**–4.5 µM). Red fluorescence emitted by the dye upon reaction with Fe^2+^ was analysed using a FACSCalibur flow cytometer and presented as a bar graph from two independent experiments (six replicates) or representative histograms (B). 3 mM N-acetyl-L-cysteine (NAC) for 2 h was used as a negative control. (C and D) Intracellular calcium level was defined in 24 h-treated HepG2 cells using flow cytometry. Fluorescent signals correlated with Ca^2+^ accumulation were counted and presented as a bar graph from two independent experiments (six replicates, C) or as representative histograms (D). Statistical significance is indicated with asterisks: (ns) *p* > 0.05, *p < 0.05, **p < 0.01, ***p < 0.001, ****p < 0.0001.

To detect the intracellular level of Fe^2+^ ions, a FerHo-Nox-1 fluorescent probe was used, which emits a red fluorescent signal specifically after reaction with free Fe^2+^ ions. Flow cytometry analysis was performed after 24-h treatment with the compounds in a concentration that corresponds to their total IC_50_ value (**34** −5 µM, **35**–4.5 µM, **36**–4 µM, **37**–4.5 µM). N-Acetyl-L-cysteine (NAC) (3 mM) was used as the inhibitor of ferrous accumulation. Quantitative analysis of fluorescence intensity demonstrated that the analysed compounds induced ferrous accumulation. The highest ferrous level was observed in the cells treated with compound **35**, whereas the lowest was observed in the cells treated with compound **36** (Figure 5(A,B)).

#### Ca^2+^ homeostasis determination

The impact of the analysed compounds on Ca^2+^ homeostasis in HepG2 cells after 24-h incubation was also investigated, taking into consideration the calcium–iron connection in ferroptosis^51^. Flow cytometry analysis revealed that all of the analysed compounds, except for compound **36**, significantly affected calcium homeostasis, leading to its high accumulation within the cells. The strongest effect on Ca^2+^ homeostasis was observed in the HepG2 cells treated with compound **35** (more than threefold compared to control), and the slightest effect was observed in the cells treated with compound **36** (Figure 5(C,D)).

Results of the analysis of ROS production, lipid peroxidation, accumulation of iron, and disturbance of Ca^2+^ homeostasis confirmed that conjugates **34**–**37** were the strongest inducers of ferroptosis in HepG2 cells. Interestingly, ferroptosis was also induced by 3-{[1–(3-(1,2-dicarba-*closo*-dodecaborane-1-yl)propyl)-1*H*-1,2,3-triazol-4-yl]methoxy}-1,8-naphthalic anhydride, 3-{[1–(3–(1,7-dicarba-*closo*-dodecaborane-1-yl)propyl)-1*H*-1,2,3-triazol-4-yl]methoxy}-1,8-naphthalic anhydride, and 3-[(1,7-dicarba-*closo*-dodecaborane-1-yl)ethylamino]-1,8-naphthalic anhydride^12^. The rest of the studied conjugates primarily induced apoptosis^11^^,^^13^. To the best of our knowledge, these are the first examples of 1,8-naphthalimide derivatives and boron cluster derivatives inducting ferroptosis.

#### Status of lysosomes in cancer and normal cell lines after treatment with compounds 34–37

Our previous studies showed that 1,8-naphthalimides modified with the carborane group at positions 3 and 4 targeted the lysosomes of living cells, with good cell membrane permeability, which also enabled the localisation of the carborane cluster in the cells^12^^,^^13^.

Cell death can be induced by lysosomes, and this type of cell death is called lysosomal membrane permeabilization (LMP)^52^. In LMP, impaired lysosomal membranes allow the release of specific lysosomal enzymes into the cytosol, resulting in the hydrolysis of various organelles. Lipophilic or amphiphilic compounds with a basic moiety get protonated and trapped within lysosomes, and such a lysosomotropic behaviour is found in many pharmacological drugs^53^. LMP and cathepsin release activates effectors, such as ROS, and iron that result in other types of cell death such as apoptosis, pyroptosis, and ferroptosis^54^. A recent study clearly demonstrated that ferroptosis is a lysosomal cell death process^55^^,^^56^.

Lysosome-targeting anticancer agents based on the 1,8-naphthalimide derivatives were limited. A previous study has demonstrated that 1,8-naphthalimide derivatives containing polyamines and long hydrocarbon chains could induce LPM^7^.

Differences in hydrophobicity between boron cluster compounds affect modes of interaction of their derivatives with lipid membranes. The mercaptododecaborate dianion ([B_12_H_11_SH]^2−^, BSH)—one of the most important boron carriers in BNCT—interacts with liposomes containing cationic or zwitterionic lipids through positively charged headgroups of these lipids^57^. Interestingly, globular dodecaborate clusters, and prominently B_12_Br_12_^2−^, can function as anionic inorganic membrane carriers, including liposomal membranes, for a wide range of hydrophilic cargo molecules (with a molecular mass of 146–4500 Da)^58^. Moreover, metallacarboranes like [COSAN]^−^ have the ability to incorporate into or cross lipid membranes in liposomes without damaging the membranes^59^^,^^60^. Carboranes and their derivatives interact with model lipid membranes and exhibit a proton-carrying activity in planar bilayers and liposomes in a concentration- and pH-dependent manner^61^.

**Figure 6.**
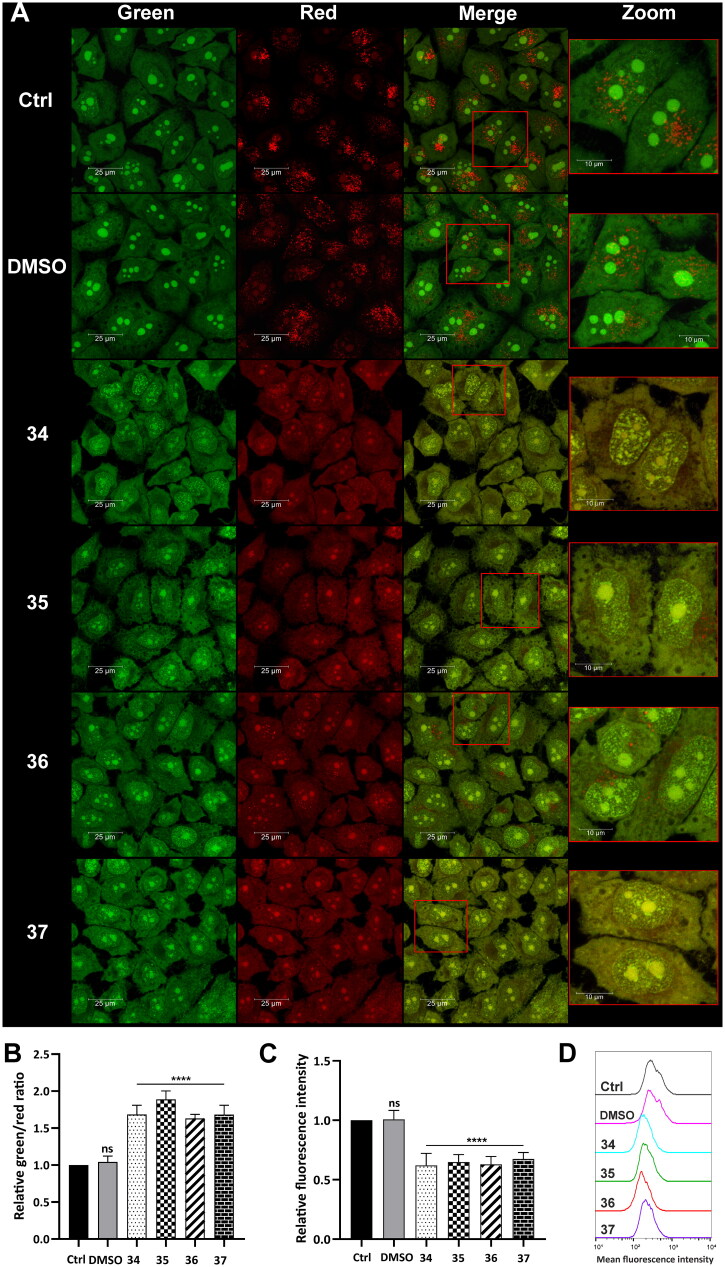
Lysosome status in HepG2 cells after treatment with compounds **34**–**37**. Confocal microscopy (A) and flow cytometry (B) analysis of lysosome integrity using AO staining in HepG2 cells treated with compounds **34**–**37** for 3 h at the final concentration corresponding to the total IC_50_ value (**34**–5 µM, **35**–4.5 µM, **36**–4 µM, **37**–4.5 µM). Untreated cells and cells treated with DMSO were used as controls. (A) AO fluorescence was visualised at two spectral settings at an ex/em wavelength of 488/505–550 nm for green fluorescence and 488/600–650 nm for red fluorescence. Merged images and magnifications of their fragments are shown on the right panels. (B) Intensity of the red and green fluorescence of the stained cells was quantified and expressed as a green/red ratio. (C and D) Flow cytometry analysis of lysosomes in HepG2 cells after 3 h of incubation with the analysed compounds using Lysotracker Deep Red staining. Intensity of the red fluorescence was measured and quantified (C). The observed fluorescence intensity shift was presented as representative histograms (D). Statistical significance is indicated with asterisks: (ns) not significant, (****) *p* < 0.0001.

Lysosomal integrity was analysed in HepG2 cells after 3-h treatment with compounds **34**–**37** in a final concentration corresponding to the total IC_50_ value (5, 4.5, 4, and 4.5 µM, respectively). After the incubation, the cells were stained with acridine orange (AO) and Lysotracker Deep Red. AO is a lysomotropic dye that accumulates in acidic lysosomes. Upon excitation with blue light, it emits red fluorescence. During lysosome membrane permeabilization, it dissipates throughout the membrane and emits green fluorescence. Confocal microscopy visualisation clearly showed the diffuse fluorescence pattern in the HepG2 cells after treatment with the analysed compounds (Figure 6(A)). Simultaneously, flow cytometry analysis was carried out, which confirmed this observation. Green and red fluorescence intensity was quantified using a FACSCalibur flow cytometer, and the green/red ratio was calculated to measure LMP^62^. Results showed that the treatment of the HepG2 cells with the modified 1,8-naphthalimides markedly increased the ratio. The highest change was observed in the cells treated with compounds **34** and **35** (approximately twofold, Figure 6(B)). Moreover, the HepG2 cells were stained with Lysotracker Deep Red, which selectively stains acidic organelles like lysosomes. Red fluorescence intensity was analysed using a flow cytometer. Results showed that only 3-h treatment with the analysed compounds significantly affected the lysosomes, which was manifested by a decrease in fluorescence intensity compared to control (Figure 6(C,D)).

1,8-Naphthalimide–boron cluster conjugates are lipophilic compounds. The *R*_M0_ values of the conjugates were in the range of 2.62–4.48, and the highest lipophilicity was found for conjugates bearing carborane clusters. A parallel artificial membrane permeability assay revealed that conjugates bearing carborane clusters exhibited a high degree of permeation^22^. Our results suggested that compounds **34**–**37** intercalate into the cell membrane of lysosomes within 3 h and induce LPM. This is consistent with the findings of Stark et al., who reported that compounds containing a lipophilic polyaromatic ring fragment undergo marked sequestration and concentration within lysosomal membranes, inducing alterations in membrane fluidity and integrity. The lysosomal membrane concentration of lysosomotropic drugs provides the first molecular basis for the disruption of the lysosomal central signalling functions and cue sensing^63^. In the present study, it was considered that ferroptosis induced by tested compounds **34**–**37** is supported by LPM. It was reported that redox-active iron from ferritin or transferrin is presumably involved in local ROS generation within endosomes/lysosomes. It has been shown labile iron was consistently observed in endosomes or lysosomes in primary hepatocytes or hepatocellular carcinoma using iron-specific fluorescent probes. Lysosomal ROS or some ferrous ions (Fe^2+^) may relocate to the cytoplasm, which in turn could induce an ROS burst. In addition, lysosomal ROS and iron may contribute to lipid ROS production because oxidising membranes were observed in the perinuclear compartments during the early phase of ferroptosis^55^.

**Figure 7.**
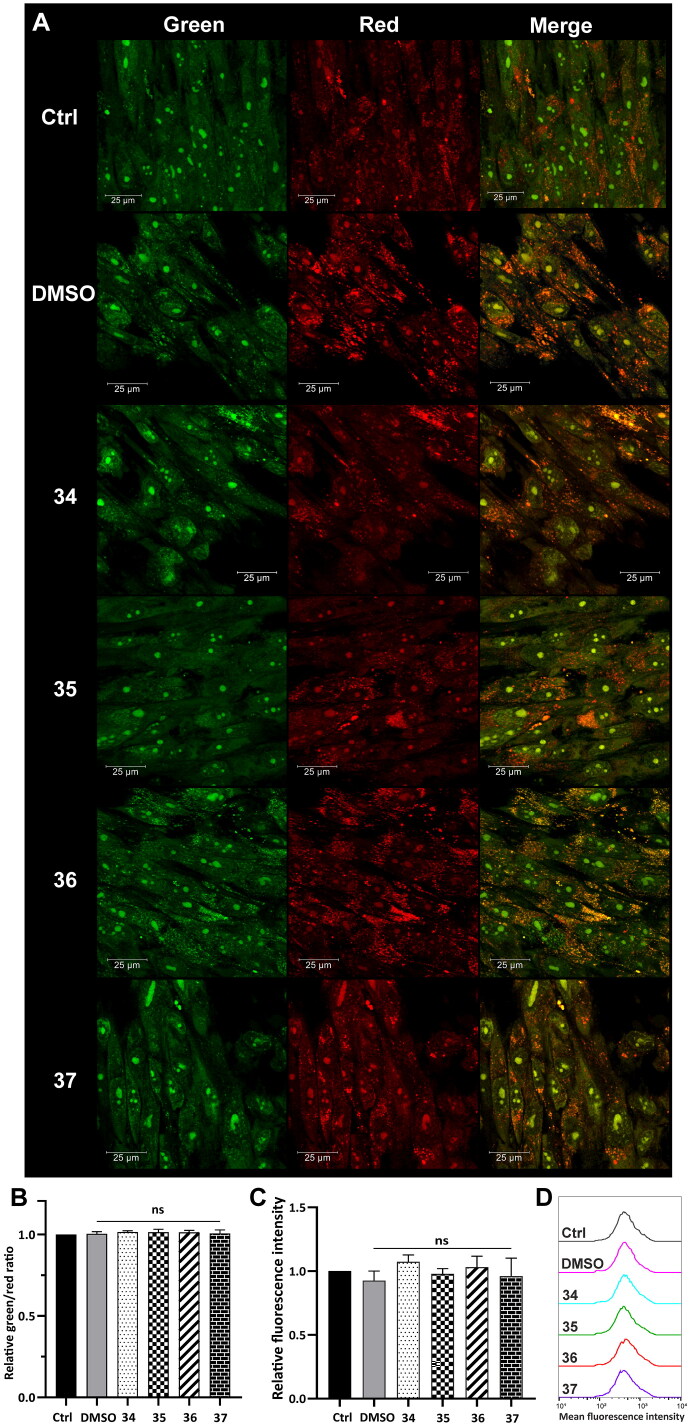
Lysosome status in MRC-5 cells after treatment with compounds **34**–**37**. Confocal microscopy (A) and flow cytometry (B) analysis of lysosome integrity using AO staining in MRC-5 cells treated with compounds **34**–**37** for 3 h at the final concentration corresponding to the total IC_50_ value (**34**–2.1 µM, **35**–2.1 µM, **36**–2.3 µM, **37** -2.3 µM). Untreated cells and cells treated with DMSO were used as controls. (A) AO fluorescence was visualised at two spectral settings at an ex/em wavelength of 488/505–550 nm for green fluorescence and 488/600–650 nm for red fluorescence. Merged images and magnifications of their fragments are shown on the right panels. (B) Intensity of the red and green fluorescence of the stained cells was quantified and expressed as a green/red ratio. (C and D) Flow cytometry analysis of lysosomes in MRC-5 cells after 3 h of incubation with the analysed compounds using Lysotracker Deep Red staining. Intensity of the red fluorescence was measured and quantified (C). The fluorescence intensity distribution was presented as representative histograms (D). Statistical significance is indicated with asterisks: (ns) not significant.

Tumour cell lysosomes are more fragile than lysosomes of normal cells and thus are more susceptible to LMP^64^^,^^65^. To verify lysosome status, the same analysis was conducted in normal human fibroblasts (MRC-5). The cells were incubated with compounds **34**–**37** in a concentration corresponding to the total IC_50_ value (Table 1). Using confocal microscopy, a strong punctate red fluorescence signal was observed in the treated MRC-5 derived from unaffected lysosomes (Figure 7(A)). Red and green fluorescence emitted from AO staining was also measured using a flow cytometer, and no changes in the green/red ratio were observed (Figure 7(B)). Lysosomal status was also analysed using Lysotracker Deep Red staining. The decrease in fluorescence intensity observed in HepG2 cells treated with the compounds was not observed for MRC-5, which indicates that the lysosomal membrane stayed intact (Figure 7(C,D)). This confirms the selectivity of the modified 1,8-naphthalimides.

#### Human topoisomerase IIα relaxation assay

DNA topoisomerases I and II (Topo I and II, respectively) play a role in solving the topological problems associated with DNA replication, transcription, recombination, and chromatin remodelling by introducing temporary single- or double-strand breaks in the DNA before changing the linking number of a helix and then resealing phosphodiester bonds. In addition, these enzymes fine-tune the steady-state level of DNA supercoiling to facilitate protein interactions with the DNA as well as prevent excessive supercoiling^66^. 1,8-Naphthalimides can exert their antitumor activities also through topoisomerase I/II inhibition^7^^,^^8^.

Our earlier study showed that the presence of the carboranyl cluster at position 3 of 1,8-naphthalimide moieties did not promote them as effective Topo II inhibitors^12^. The 4-carboranyl-1,8-naphthalimides studied showed anti-Topo II activity with IC_50_ = 0.58–61.59 µM. Two compounds—*N*-[2-(dimethylamino)ethyl]-4-{1-[3-(1,2-dicarba-*closo*-dodecaborane-1-yl)propyl]-1*H*-1,2,3-triazol-4-yl}-1,8-naphthalimide (IC_50_ = 0.58 µM) and *N*-[2-(*N*-pyrrolidinyl)ethyl]-4-[(2–(3–(1,2-dicarba-*closo*-dodecaborane-1-yl)propanamido)ethyl)amino]-1,8-naphthalimide (IC_50_ = 2.81 µM)—were found to be more active than mitonafide (IC_50_ = 5.13 µM).

Based on these findings, conjugates **34**–**37** were tested in the screening assay for human topoisomerase IIα inhibitory activity at the concentration of 100 µM (Figure S153, ESI). However, they inhibited DNA migration in the gel, probably due to intercalation, and it was impossible to determine their effect on human topoisomerase IIα. Therefore, these compounds were subjected to further detailed analyses of inhibitory potential at concentrations of 1, 5, and 10 µM (Figure S154, ESI). None of the tested compounds inhibited Topo II activity. However, a difference in DNA migration in the gel was observed with an increase in the concentration of compounds, indicating intercalation into the DNA strands.

#### DNA unwinding assay

Compounds that are able to intercalate into DNA or bind in the groove are involved in the local unwinding of the DNA, leading to a decrease in the twist number and an increase in the writhe number of the previously relaxed plasmid DNA used in the assay, resulting in a positively supercoiled DNA. If this DNA is then nicked by a topoisomerase, the enzyme will remove supercoils and relax the DNA, followed by religation. Upon removal of the compound and the enzyme by butanol extraction, the twist will increase, so the writhe will decrease, resulting in more negatively supercoiled DNA, which runs faster on agarose gel. The occurrence of a band representing supercoiled plasmid DNA on agarose gel indicates that the compound is an intercalator or groove binder. If the compound does did not interact with the DNA, then it will be a relaxed plasmid will be observed. This assay should be verified by performing a second reaction using a negatively supercoiled plasmid as a substrate to confirm that the compound is not simply acting as an inhibitor of the Topo I.

**Figure 8.**
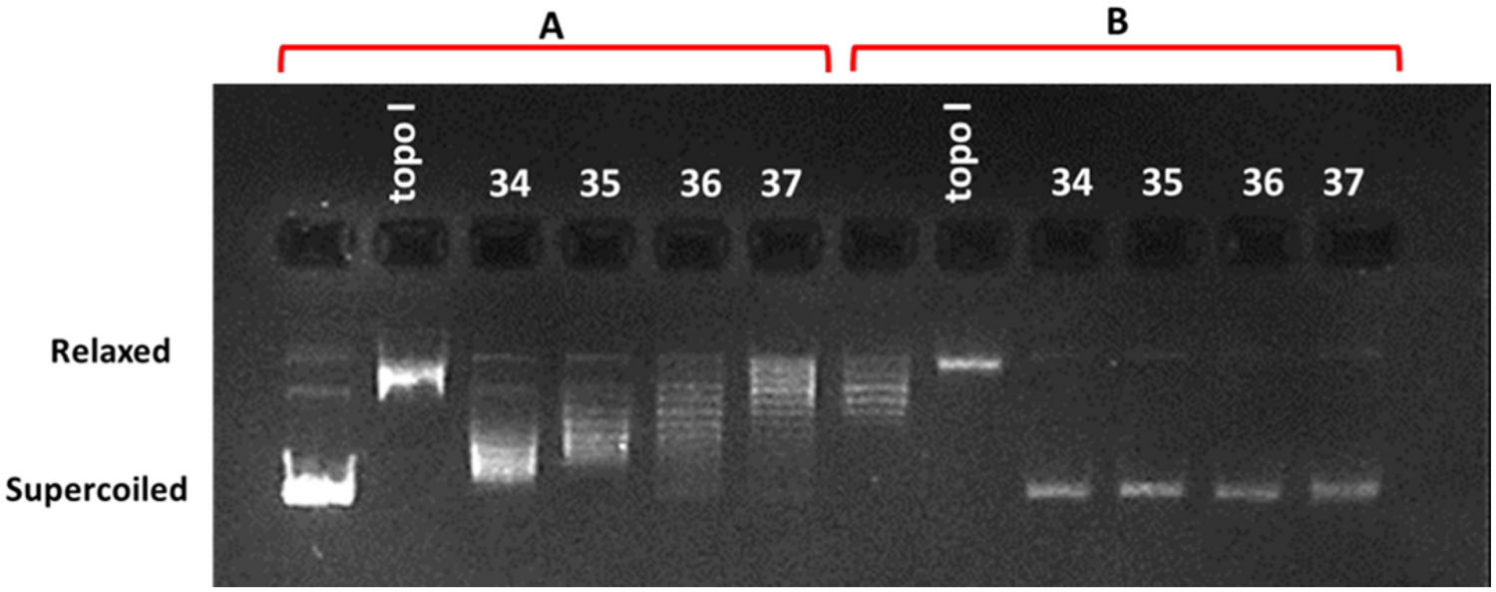
DNA unwinding assay in the presence of compounds **34**–**37** at a concentration of 10 μM. Panel **A** indicates the reaction with a supercoiled pBR 322 as a substrate for wheat germ topoisomerase I (Topo I), and panel **B** indicates the reaction with a relaxed plasmid as a substrate. Topo I indicates control reactions without any drug. Supercoiled and relaxed fractions of the plasmid DNA are marked.

Incubation of the relaxed plasmid with the tested compounds **34**–**37** in the presence of Topo I showed the appearance of a supercoiled DNA fraction in each case, which proves the intercalating effect of these compounds (Figure 8(B)). This was further confirmed in the second reaction using a supercoiled plasmid as a substrate. The absence of an intensive supercoiled DNA on agarose gel after incubation of the tested compounds with Topo I indicates that they are not strong inhibitors of this enzyme (Figure 8(A)). Depending on the degree of supercoiling, DNA runs as a ladder of topoisomers on the gel when poor intercalators were tested but also when strong intercalators were used at low concentrations, or at the same position as the substrate. Among the studied compounds, the most relaxed topoisomers were observed in the presence of conjugate **37**, which indicates that it was the strongest DNA intercalator in this pool what is consistent with physicochemical studies.

## Conclusions

In this study, convenient protocols for the synthesis of novel 1,8-naphthalimide derivatives containing *ortho*- or *meta*-carborane cluster at position 4 and nitro group at position 3 of the heteroaromatic skeleton as analogs of mitonafide and pinafide were tested. The X-ray structure of the pinafide analog bearing *meta*-carborane **15** was established. The DNA-binding properties of the synthesised compounds were investigated using thermal denaturation experiments, CD spectroscopy, and UV–vis spectroscopy. Conjugates **34**–**37**, which were obtained via reductive amination, with an amine linker -NH-(CH_2_)_6_-NH-(CH_2_)_2_- between the carborane group and the 1,8-naphthalimide moiety, were found to interact most strongly with ct-DNA among all the 1,8-naphthalimide–carborane conjugates synthesised in our laboratory, containing boron clusters attached to imide, at position 3 or 4 of the ring systems, and also mitonafide. Among this group of derivatives, the conjugates containing *meta*-carborane showed the strongest effect. This indicated an intercalative binding mode, which was additionally confirmed using the DNA unwinding assay. A similar compound with a shorter linker -NH-(CH_2_)_2_-NH-(CH_2_)_2_- showed weak DNA stabilisation. Compounds **34**–**37** were cytotoxic against HepG2 cells with IC_50_ values in the range of 3.62–4.78 µM. *In vitro* cytotoxic activity decreased in the order **36 **>** 37 **>** 35 **>** 34**. This study also showed that these compounds could effectively induce cell cycle arrest at the G2M phase and inhibit cell migration but did not have inhibitory activity against Topo II. This study further demonstrated that compounds **34**–**37** induced ferroptosis as a dominant cell death process in the tested cancer cells (HepG2), which was confirmed by intracellular iron accumulation and subsequent lipid peroxidation measurement. The same compounds also induced LMP. Lysosomes contribute to ferroptosis by modulating iron equilibria and ROS expression. In comparison with the normal cell line (MRC-5), LMP process was not confirmed, which ensures the selectivity of the modified compounds. The results showed mitonafide analog **35** bearing *meta*-carborane as the most promising among the tested compounds. It interacted most strongly with DNA, was most effective in inhibiting cell migration, and had the greatest impact on GSH and Fe^2+^ levels, MMP changes, and Ca^2+^ homeostasis. These properties play an important role in anticancer activity through ferroptosis induction.

This study showed that selected 4-carboranyl-3-nitro-1,8-naphthalimide derivatives have the structural features of DNA intercalators, ferroptosis inducers, and lysosomotropic detergents. These encouraging results suggest that such inorganic–organic hybrids can be considered novel multitarget compounds with anticancer activity. Further studies on these compounds are under way in our laboratory.

## Materials and methods

### Chemistry

Most of the chemicals used in this study were obtained from the Acros Organics (Geel, Belgium) and used without further purification unless otherwise stated. 4-Bromonaphthalic anhydride (**1**) was obtained from the TCI (Tokyo, Japan) and used without further purification. Boron clusters were purchased from KATCHEM spol. s.r.o. (Řež/Prague, Czech Republic). All experiments involving water-sensitive compounds were conducted under thorough dry conditions and an argon atmosphere. Flash column chromatography was performed on silica gel 60 (230–400 mesh, Sigma-Aldrich). *R*_f_ refers to analytical TLC performed using precoated silica gel 60 F254 plates purchased from Sigma-Aldrich (Steinheim, Germany) and developed in the solvent system indicated. The compounds were visualised using UV light (254 nm) or a 0.5% acidic solution of PdCl_2_ in HCl/methanol by heating with a heat gun for boron-containing derivatives. The yields were not optimised.

^1^H-NMR, ^13^C-NMR, and ^11^B-NMR spectra were recorded on a Bruker Avance III 600 MHz spectrometer equipped with a direct ATM probe. The spectra for ^1^H, ^13^C, and ^11^B nuclei were recorded at 600.26, 150.94, and 192.59 MHz, respectively. Deuterated solvents were used as standards. The following abbreviations are used to denote the multiplicities: s = singlet, d = doublet, dd = doublet of doublets, ddd = doublet of doublets of doublets, t = triplet, dt = doublet of triplets, q = quartette, quin = quintet, br s = broad singlet, and m = multiplet. *J* values were expressed in Hz.

Mass spectra were recorded on a CombiFlash PurIon Model Eurus35 (Teledyne ISCO, Lincoln, USA). Ionisation was achieved by atmospheric pressure chemical ionisation (APCI) ionisation in the positive ion mode (APCI+) and the negative ion mode (APCI−). The entire flow was directed to the APCI ion source operating in the positive ion mode. Total ion chromatograms were recorded in the *m*/*z* range of 100–700. Vaporisation and capillary temperature were set at 400 and 300 °C, respectively. Capillary voltage was 150 V, and current discharge was 10 µA. High-resolution mass spectrometry (HRMS) measurements were performed using Synapt G2-Si mass spectrometer (Waters) equipped with an ESI/APCI source and quadrupole-Time-of-flight mass analyser. To ensure accurate mass measurements, data were collected in centroid mode and mass was corrected during acquisition using leucine enkephalin solution as an external reference (Lock-SprayTM). The results of the measurements were processed using the MassLynx 4.1 software (Waters) incorporated with the instrument. Data were presented for the most abundant mass in the boron distribution plot of the base peak (100%) and for the peak corresponding to the highest *m*/*z* value with its relative abundance (%).

The theoretical molecular mass peaks of the compounds were calculated using the “Show Analysis Window” option in the ChemDraw Ultra 12.0 program. The calculated *m*/*z* corresponded to the average mass of the compounds consisting of natural isotopes.

Infrared spectra (IR) were recorded using a Nicolet 6700 Fourier-transform infrared spectrometer (Thermo Scientific) equipped with an ETC EverGlo* source for the IR range, a Geon-KBr beam splitter, and a DLaTGS/KBr detector with a smart orbit sampling compartment and a diamond window. The samples were placed directly on the diamond crystal, and pressure was applied to make the surface of the sample conform to that of the diamond crystal.

UV measurements were performed using a GBC Cintra10 UV–VIS spectrometer (Dandenong, Australia). Before the UV experiment, the samples were dissolved in 99.8% C_2_H_5_OH. The measurement was performed at ambient temperature.

RP-HPLC analysis was performed on a Hewlett–Packard 1050 system equipped with a UV detector and a Hypersil Gold C18 column (4.6 × 250 mm, 5 µm particle size, Thermo Scientific, Runcorn, UK). UV detection was carried out at 380 nm. The flow rate was set at 1 ml min^−1^. All analyses were run at ambient temperature. The gradient elution was as follows: 10 min from 0 to 25% A, 10 min from 25 to 60% A, and 10 min from 60 to 0% A. Buffer A contained 0.1% HCOOH in CH_3_CN, and buffer B contained 0.1% HCOOH in H_2_O.

Crystals of **15** were obtained by slow evaporation from the mixture of MeOH and CH_2_Cl_2_ (3:2, v/v). X-ray diffraction measurements were carried out under cryogenic conditions on an XtaLab Synergy four-circle diffractometer (Oxford Diffraction) equipped with a Cu rotating anode PhotonJet X-ray source and a HyPis-6000HE CCD detector. The data were processed using the CRYSALISPRO software (Rigaku Oxford Diffraction), and the structure was solved with SHELXT and refined with SHELXL programs^67^^,^^68^. The refinement of atomic positions was unrestrained except for hydrogen atoms that were maintained at riding positions. Table S1 (ESI) summarises the crystallographic data.

4-Bromo-*N*-[2-(dimethylamino)ethyl]-1,8-naphthalimide (**2**) and 4-bromo-*N*-[2-(*N*-pyrrolidinyl)ethyl]-1,8-naphthalimide (**3**) were obtained via the reaction of 4-bromonaphthalic anhydride (**1**) with appropriate amine *N*,*N*-dimethylethylenediamine and *N*-(2-aminoethyl)pyrrolidine, respectively^69^. 3–(1,2-Dicarba-*closo*-dodecaboran-1-yl)propionic acid (**10**) was synthesised as described in the literature^28^. 3-(1,7-Dicarba-*closo*-dodecaboran-1-yl)propionic acid (**11**) was also synthesised as described earlier^29^. 2–(1,2-Dicarba-*closo*-dodecaboran-1-yl)ethanal (**20**) and 2–(1,7-dicarba-*closo*-dodecaboran-1-yl)ethanal (**51**) were synthesised using a procedure described in the literature^31^.

#### Synthesis of 4-bromo-3-nitro-N-[2-(N-pyrrolidinyl)ethyl]-1,8-naphthalimide (5)

4-Bromo-*N*-[2-(*N*-pyrrolidinyl)ethyl]-1,8-naphthalimide (**3**) (42 mg, 112.9 µmol) was dissolved in concentrated H_2_SO_4_ (0.6 ml) at 0 °C, and NaNO_3_ (14.5 mg, 170.6 µmol) was added in several portions. The reaction mixture was stirred for 1 h at 0 °C and for 3 h at rt, poured on ice, and then neutralised with saturated NaHCO_3_. The crude product was extracted to CH_2_Cl_2_ (4 × 5 ml), the organic layer was dried over MgSO_4_. Then, the drying agent was filtered off and washed with CH_2_Cl_2_. Filtrate and washings were combined and evaporated to dryness under a vacuum. The crude product was purified by column chromatography on silica gel (230–400 mesh) using a gradient of MeOH (0–7%) in CH_2_Cl_2_ as an eluting solvent system, and a brown solid was obtained. Yield: 35.1 mg (75%). TLC (MeOH/CH_2_Cl_2_, 1:9, *v/v*): *R*_f_ = 0.52; ^1^H-NMR (CDCl_3_, 600.26 MHz): *δ* (ppm) = 8.82–8.79 (m, 3H, 3H_arom_), 8.02 (t, *J* = 7.9 Hz, 1H, H_arom_), 4.41 (t, *J* = 6.9 Hz, 2H, CH_2_-N(CO)_2_), 2.90 (br s, 2H, CH_2_-pyrrolidine), 2.73 (br s, 4H, 2 × N-C*H*_2pyrrolidine_-CH_2_), 1.83 (br s, 4H, 2 × CH_2_-C*H*_2pyrrolidine_-CH_2_).

#### Synthesis of 1,8-naphthalimide derivatives 6–9

4-Bromo-*N*-[2-(dimethylamino)ethyl]-3-nitro-1,8-naphthalimide (**4**) or 4-bromo-3-nitro-*N*-[2-(*N*-pyrrolidinyl)ethyl]-1,8-naphthalimide (**5**) was suspended in absolute EtOH (30 ml per 1 mmol). Then, ethane-1,2-diamine or hexane-1,6-diamine (9.5 equiv.) was added, and the mixture was refluxed under dry argon for 2 h. The mixture was subsequently cooled down to rt, and the solvent was evaporated. The crude product was purified by column chromatography on silica gel (230–400 mesh) using MeOH (5–20%) in CH_2_Cl_2_ with the addition of 1% of TEA as an eluting solvent system.

##### 4-[(2-Aminoethyl)amino]-N-[2-(dimethylamino)ethyl]-3-nitro-1,8-naphthalimide (6)

orange solid, yield: 58.1 mg (79%). TLC (2 × TEA/MeOH/CH_2_Cl_2_, 1:20:80, *v/v*): *R*_f_ = 0.58; ^1^H-NMR (acetone-d_6_, 600.26 MHz): *δ* (ppm) = 9.06 (s, 1H, H_arom_), 8.95 (d, *J* = 8.5 Hz, 1H, H_arom_), 8.61 (d, *J* = 7.3 Hz, 1H, H_arom_), 7.82 (t, *J* = 7.9 Hz, 1H, H_arom_), 4.26 (t, *J* = 5.6 Hz, 2H, C*H*_2_-NH), 4.21 (t, *J* = 6.9 Hz, 2H, CH_2_-N(CO)_2_), 3.55 (t, *J* = 5.5 Hz, 2H, C*H*_2_-NH_2_), 2.56 (t, *J* = 6.9 Hz, 2H, C*H*_2_-N(CH_3_)_2_), 2.25 (s, 6H, N(CH_3_)_2_).

##### 4-[(2-Aminoethyl)amino]-3-nitro-N-[2-(N-pyrrolidinyl)ethyl]-1,8-naphthalimide (7)

orange solid, yield: 17.5 mg (77%). TLC (2 × TEA/MeOH/CH_2_Cl_2_, 1:20:80, *v/v*): *R*_f_ = 0.58; ^1^H-NMR (acetone-d_6_, 600.26 MHz): *δ* (ppm) = 9.05 (s, 1H, H_arom_), 8.94 (d, *J* = 8.5 Hz, 1H, H_arom_), 8.60 (d, *J* = 7.3 Hz, 1H, H_arom_), 7.81 (t, *J* = 7.9 Hz, 1H, H_arom_), 4.31 (t, *J* = 6.8 Hz, 2H, CH_2_-N(CO)_2_), 4.25 (t, *J* = 5.6 Hz, 2H, C*H*_2_-NH), 3.55 (t, *J* = 5.6 Hz, 2H, C*H*_2_-NH_2_), 3.06 (br s, 2H, CH_2_-pyrrolidine), 2.95 (br s, 4H, 2 × N-C*H*_2pyrrolidine_-CH_2_ overlapped with H_2_O), 1.80 (br s, 4H, 2 × CH_2_-C*H*_2pyrrolidine_-CH_2_).

##### 4-[(6-Aminohexyl)amino]-N-[2-(dimethylamino)ethyl]-3-nitro-1,8-naphthalimide (8)

orange solid, yield: 17.4 mg (70%). TLC (2 × TEA/MeOH/CH_2_Cl_2_, 1:20:80, *v/v*): *R*_f_ = 0.43; ^1^H-NMR (MeOH-d_4_, 600.26 MHz): *δ* (ppm) = 8.94 (s, 1H, H_arom_), 8.78 (dd, *J* = 8.5, 1.1 Hz, 1H, H_arom_), 8.56 (dd, *J* = 7.4, 1.0 Hz, 1H, H_arom_), 7.74 (dd, *J* = 8.5, 7.4 Hz, 1H, H_arom_), 4.22 (t, *J* = 7.0 Hz, 2H, CH_2_-N(CO)_2_), 3.94 (t, *J* = 6.9 Hz, 2H, C*H*_2_-NH), 2.83–2.78 (m, 2H, C*H*_2_-N(CH_3_)_2_ overlapped with TEA), 2.65 (t, *J* = 7.0 Hz, 2H, C*H*_2_-NH_2_), 2.35 (s, 6H, N(CH_3_)_2_), 1.93–1.84 (m, 2H, CH_2_-C*H*_2_-CH_2_), 1.65–1.57 (m, 4H, 2 × CH_2_-C*H*_2_-CH_2_), 1.56–1.49 (m, 2H, CH_2_-C*H*_2_-CH_2_).

##### 4-[(6-Aminohexyl)amino]-3-nitro-N-[2-(N-pyrrolidinyl)ethyl]-1,8-naphthalimide (9)

orange solid, yield: 26.6 mg (70%). TLC (2 × TEA/MeOH/CH_2_Cl_2_, 1:20:80, *v/v*): *R*_f_ = 0.43; ^1^H-NMR (MeOH-d_4_, 600.26 MHz): *δ* (ppm) = 8.88 (s, 1H, H_arom_), 8.78 (dd, *J* = 8.5, 1.1 Hz, 1H, H_arom_), 8.54 (d, *J* = 7.4, 1.0 Hz, 1H, H_arom_), 7.73 (dd, *J* = 8.6, 7.3 Hz, 1H, H_arom_), 4.23 (t, *J* = 6.9 Hz, 2H, CH_2_-N(CO)_2_), 3.94 (t, *J* = 6.9 Hz, 2H, C*H*_2_-NH), 2.96–2.91 (m, 2H, C*H*_2_-NH_2_), 2.87 (t, *J* = 6.9 Hz, 2H, CH_2_-pyrrolidine), 2.79–2.77 (m, 4H, 2 × N-C*H*_2pyrrolidine_-CH_2_), 1.94–1.84 (m, 6H, 2 × CH_2_-C*H*_2pyrrolidine_-CH_2_ overlapped with CH_2_-C*H*_2_-CH_2_), 1.72–1.69 (m, 2H, CH_2_-C*H*_2_-CH_2_), 1.61–1.48 (m, 4H, 2 × CH_2_-C*H*_2_-CH_2_).

#### Synthesis of 1,8-naphthalimide derivatives 12–19 modified with carborane cluster via amidation reaction

4-[(2-Aminoethyl)amino]-*N*-[2-(dimethylamino)ethyl]-3-nitro-1,8-naphthalimide (**6**), 4-[(2-aminoethyl)amino]-3-nitro-*N*-[2-(*N*-pyrrolidinyl)ethyl]-1,8-naphthalimide (**7**), 4-[(6-aminohexyl)amino]-*N*-[2-(dimethylamino)ethyl]-3-nitro-1,8-naphthalimide (**8**), or 4-[(6-aminohexyl)amino]-3-nitro-*N*-[2-(*N*-pyrrolidinyl)ethyl]-1,8-naphthalimide (**9**) and 3–(1,2-dicarba-*closo*-dodecaboran-1-yl)propionic acid (**10**) (1.05 equiv.) or 3–(1,7-dicarba-*closo*-dodecaboran-1-yl)propionic acid (**11**) (1.05 equiv.) were dissolved in anhydrous CH_2_Cl_2_ (22 ml per 1 mmol), and then anhydrous TEA (2.1 equiv.) and PyBOP (1.1 equiv.) were added. The reaction mixture was stirred for 2–5 h at rt under an inert (Ar) atmosphere. Then, it was diluted with CH_2_Cl_2_ (33 ml per 1 mmol) and washed with the same volume of H_2_SO_4_ (2.5%) (compound **17** was diluted with CH_2_Cl_2_ and incubated with 2 M HCl (2 × 1 h)), NaHCO_3_ (2.5%), and finally twice with water. The organic phase was separated, dried over MgSO_4_, filtered, and evaporated to dryness. Then, the product was purified by column chromatography on silica gel (230–400 mesh) using a gradient of MeOH (3–10%) in CH_2_Cl_2_ as an eluting solvent system. Compounds **16**, **18**, and **19** were purified again by column chromatography under the same conditions as described above.

##### 4-[(2-[3–(1,2-Dicarba-closo-dodecaborane-1-yl)propanamido]ethyl)amino]-N-[2-(dimethylamino)ethyl]-3-nitro-1,8-naphthalimide (12)

orange solid, yield: 11.2 mg (73%). TLC (MeOH/CH_2_Cl_2_, 3:7, *v/v*): *R*_f_ = 0.51; ^1^H-NMR (MeOH-d_4_, 600.26 MHz): *δ* (ppm) = 8.84 (s, 1H, H_arom_), 8.67 (dd, *J* = 8.5, 0.9 Hz, 1H, H_arom_), 8.53 (dd, *J* = 7.3, 0.9 Hz, 1H, H_arom_), 7.73 (dd, *J* = 8.5, 7.5 Hz, 1H, H_arom_), 4.49 (br s, 1H, CH_carborane_), 4.27 (t, *J* = 6.8 Hz, 2H, CH_2_-N(CO)_2_), 3.90 (t, *J* = 5.7 Hz, 2H, C*H*_2_-NH), 3.55 (t, *J* = 5.7 Hz, 2H, C*H*_2_-NH), 2.91 (t, *J* = 6.8 Hz, 2H, C*H*_2_-N(CH_3_)_2_), 2.57 (s, 6H, N(CH_3_)_2_), 2.50 (dd, *J* = 9.0, 6.6 Hz, 2H, CH_2_-carborane), 2.36 (dd, *J* = 9.0, 6.6 Hz, 2H, NH-C(O)-C*H*_2_), 3.0–1.5 (m, 10H, B_10_H_10_); ^13^C-NMR (MeOH-d_4_, 150.95 MHz): *δ* (ppm) = 173.89 (1 C, C(O)-NH), 165.15, 164.14 (2 C, C11, C12), 151.19–111.50 (10 C, 10 C_arom_), 76.21 (1 C, C_carborane_), 63.93 (1 C, CH_carborane_), 57.56 (1 C, *C*H_2_-N(CH_3_)_2_), 50.58 (1 C, CH_2_-NH), 45.27 (2 C, 2 × CH_3_), 40.92 (1 C, CH_2_-NH), 38.03 (1 C, *C*H_2_-N(CO)_2_), 35.74–33.97 (2 C, CH_2_-carborane and NH-C(O)-*C*H_2_); ^11^B-NMR {^1^H BB} (MeOH-d_4_, 192.59 MHz): *δ* (ppm) = −2.79 (s, 1B, B9), −5.99 (s, 1B, B12), −9.70 (s, 2B, B8,10), −11.92–−13.07 (m, 6B, B3,4,5,6,7,11); UV (99.8% CH_3_CH_2_OH): λ_max_ = 246, 393 nm, λ_min_ = 226, 329 nm, λ_sh_ = 311 nm; FT-IR: *ν*_max_ (cm–^1^) = 2930 (C-H_aliphat_), 2577 (B-H), 1697 (C = O), 1647 (C = O), 723 (B-B); RP-HPLC: *t*_R_ = 20.52 min; APCI-MS: *m/z*: 570 [M + H]^+^, calcd for C_23_H_35_B_10_N_5_O_5_ = 569.

##### 4-[(2-[3–(1,7-Dicarba-closo-dodecaborane-1-yl)propanamido]ethyl)amino]-N-[2-(dimethylamino)ethyl]-3-nitro-1,8-naphthalimide (13)

orange solid, yield: 9.8 mg (65%). TLC (MeOH/CH_2_Cl_2_, 3:7, *v/v*): *R*_f_ = 0.52; ^1^H-NMR (MeOH-d_4_, 600.26 MHz): *δ* (ppm) = 8.86 (s, 1H, H_arom_), 8.67 (dd, *J* = 8.5, 0.8 Hz, 1H, H_arom_), 8.54 (dd, *J* = 7.4, 0.8 Hz, 1H, H_arom_), 7.74 (dd, *J* = 8.5, 7.5 Hz, 1H, H_arom_), 4.26 (t, *J* = 6.9 Hz, 2H, CH_2_-N(CO)_2_), 3.88 (t, *J* = 5.7 Hz, 2H, C*H*_2_-NH), 3.52 (t, *J* = 5.6 Hz, 2H, C*H*_2_-NH), 3.48 (br s, 1H, CH_carborane_), 2.83 (t, *J* = 6.8 Hz, 2H, C*H*_2_-N(CH_3_)_2_), 2.50 (s, 6H, N(CH_3_)_2_), 2.20–2.17 (m, 2H, CH_2_-carborane), 2.14–2.11 (m, 2H, NH-C(O)-C*H*_2_), 3.0–1.5 (m, 10H, B_10_H_10_); ^13^C-NMR (MeOH-d_4_, 150.95 MHz): *δ* (ppm) = 174.34 (1 C, C(O)-NH), 165.14, 164.13 (2 C, C11, C12), 151.17–111.58 (10 C, 10 C_arom_), 76.52 (1 C, C_carborane_), 57.61 (1 C, *C*H_2_-N(CH_3_)_2_), 57.04 (1 C, CH_carborane_), 50.56 (1 C, CH_2_-NH), 45.44 (2 C, 2 × CH_3_), 40.94 (1 C, CH_2_-NH), 38.27 (1 C, *C*H_2_-N(CO)_2_), 36.59–33.29 (2 C, CH_2_-carborane and NH-C(O)-*C*H); ^11^B-NMR {^1^H BB} (MeOH-d_4_, 192.59 MHz): *δ* (ppm) = −4.55 (s, 1B, B5), −9.87–−11.03 (m, 5B, B4,6,9,10,12), −13.56 (s, 2B, B8,11), −15.24 (s, 2B, B2,3); UV (99.8% CH_3_CH_2_OH): λ_max_ = 247, 393 nm, λ_min_ = 226, 329 nm, λ_sh_ = 309 nm; FT-IR: *ν*_max_ (cm–^1^) = 2931 (C-H_aliphat_), 2595 (B-H), 1697 (C = O), 1647 (C = O), 729 (B-B); RP-HPLC: *t*_R_ = 20.40 min; APCI-MS: *m/z*: 570 [M + H]^+^, calcd for C_23_H_35_B_10_N_5_O_5_ = 569.

##### 4-[(2-[3–(1,2-Dicarba-closo-dodecaborane-1-yl)propanamido]ethyl)amino]-3-nitro-N-[2-(N-pyrrolidinyl)ethyl]-1,8-naphthalimide (14)

orange solid, yield: 13.5 mg (52%). TLC (MeOH/CH_2_Cl_2_, 3:7, *v/v*): *R*_f_ = 0.50; ^1^H-NMR (MeOH-d_4_, 600.26 MHz): *δ* (ppm) = 8.80 (s, 1H, H_arom_), 8.66 (d, *J* = 7.9 Hz, 1H, H_arom_), 8.52 (d, *J* = 6.8 Hz, 1H, H_arom_), 7.72 (dd, *J* = 8.3, 7.5 Hz, 1H, H_arom_), 4.50 (br s, 1H, CH_carborane_), 4.31 (t, *J* = 6.5 Hz, 2H, CH_2_-N(CO)_2_), 3.89 (t, *J* = 5.6 Hz, 2H, C*H*_2_-NH), 3.56 (t, *J* = 5.6 Hz, 2H, C*H*_2_-NH), 3.18 (t, *J* = 6.6 Hz, 2H, CH_2_-pyrrolidine), 3.12 (br s, 4H, 2 × N-C*H*_2pyrrolidine_-CH_2_), 2.50 (dd, *J* = 9.1, 6.5 Hz, 2H, CH_2_-carborane), 2.37 (dd, *J* = 9.1, 6.5 Hz, 2H, NH-C(O)-C*H*_2_), 2.01–1.93 (m, 4H, 2 × CH_2_-C*H*_2pyrrolidine_-CH_2_), 3.0–1.5 (m, 10H, B_10_H_10_); ^13^C-NMR (MeOH-d_4_, 150.95 MHz): *δ* (ppm) = 173.92 (1 C, C(O)-NH), 165.14, 164.11 (2 C, C11, C12), 151.14–111.27 (10 C, 10 C_arom_), 76.23 (1 C, C_carborane_), 63.94 (1 C, CH_carborane_), 55.63 (2 C, 2 × N-*C*H_2pyrrolidine_-CH_2_), 54.54 (1 C, CH_2_-pyrrolidine), 50.57 (1 C, CH_2_-NH), 40.86 (1 C, CH_2_-NH), 38.67 (1 C, *C*H_2_-N(CO)_2_), 35.75–33.97 (2 C, CH_2_-carborane and NH-C(O)-*C*H_2_), 24.14 (2 C, 2 × CH_2_-*C*H_2pyrrolidine_-CH_2_); ^11^B-NMR {^1^H BB} (MeOH-d_4_, 192.59 MHz): *δ* (ppm) = −2.79 (s, 1B, B9), −6.01 (s, 1B, B12), −9.70 (s, 2B, B8,10), −11.92–−13.00 (m, 6B, B3,4,5,6,7,11); UV (99.8% CH_3_CH_2_OH): λ_max_ = 246, 394 nm, λ_min_ = 227, 329 nm, λ_sh_ = 309 nm; FT-IR: *ν*_max_ (cm–^1^) = 2930 (C-H_aliphat_), 2577 (B-H), 1697 (C = O), 1647 (C = O), 722 (B-B); RP-HPLC: *t*_R_ = 20.74 min; APCI-MS: *m/z*: 596 [M + H]^+^, calcd for C_25_H_37_B_10_N_5_O_5_ = 595.

##### 4-[(2-[3–(1,7-Dicarba-closo-dodecaborane-1-yl)propanamido]ethyl)amino]-3-nitro-N-[2-(N-pyrrolidinyl)ethyl]-1,8-naphthalimide (15)

orange solid, yield: 15.6 mg (60%). TLC (MeOH/CH_2_Cl_2_, 3:7, *v/v*): *R*_f_ = 0.50; ^1^H-NMR (MeOH-d_4_, 600.26 MHz): *δ* (ppm) = 8.82 (s, 1H, H_arom_), 8.67 (d, *J* = 8.5 Hz, 1H, H_arom_), 8.53 (dd, *J* = 7.3, 0.8 Hz, 1H, H_arom_), 7.73 (dd, *J* = 8.4, 7.4 Hz, 1H, H_arom_), 4.32 (d, *J* = 6.6 Hz, 2H, CH_2_-N(CO)_2_), 3.87 (t, *J* = 5.6 Hz, 2H, C*H*_2_-NH), 3.53 (t, *J* = 5.6 Hz, 2H, C*H*_2_-NH), 3.49 (br s, 1H, CH_carborane_), 3.19 (t, *J* = 6.6 Hz, 2H, CH_2_-pyrrolidine), 3.13 (br s, 4H, 2 × N-C*H*_2pyrrolidine_-CH_2_), 2.21 (dt, *J* = 6.8, 4.0 Hz, 2H, CH_2_-carborane), 2.15 (dd, *J* = 7.8, 4.1 Hz, 2H, NH-C(O)-C*H*_2_), 1.99–1.95 (m, 4H, 2 × CH_2_-C*H*_2pyrrolidine_-CH_2_), 3.0–1.5 (m, 10H, B_10_H_10_); ^13^C-NMR (MeOH-d_4_, 150.95 MHz): *δ* (ppm) = 174.39 (1 C, C(O)-NH), 165.18, 164.14 (2 C, C11, C12), 151.13–111.28 (10 C, 10 C_arom_), 76.53 (1 C, C_carborane_), 57.05 (1 C, CH_carborane_), 55.63 (2 C, 2 × N-*C*H_2pyrrolidine_-CH_2_), 54.56 (1 C, CH_2_-pyrrolidine), 50.53 (1 C, CH_2_-NH), 40.84 (1 C, CH_2_-NH), 38.69 (1 C, *C*H_2_-N(CO)_2_), 36.60–33.29 (2 C, CH_2_-carborane and NH-C(O)-*C*H_2_), 24.13 (2 C, 2 × CH_2_-*C*H_2pyrrolidine_-CH_2_); ^11^B-NMR {^1^H BB} (MeOH-d_4_, 192.59 MHz): *δ* (ppm) = −4.53 (s, 1B, B5), −9.89–−11.04 (m, 5B, B4,6,9,10,12), −13.57 (s, 2B, B8,11), −15.23 (s, 2B, B2,3); UV (99.8% CH_3_CH_2_OH): λ_max_ = 247, 394 nm, λ_min_ = 226, 329 nm, λ_sh_ = 309 nm; FT-IR: *ν*_max_ (cm–^1^) = 2928 (C-H_aliphat_), 2595 (B-H), 1697 (C = O), 1650 (C = O), 730 (B-B); RP-HPLC: *t*_R_ = 20.61 min; APCI-MS: *m/z*: 596 [M + H]^+^, calcd for C_25_H_37_B_10_N_5_O_5_ = 595.

##### 4-[(6-[3–(1,2-Dicarba-closo-dodecaborane-1-yl)propanamido]hexyl)amino]-N-[2-(dimethylamino)ethyl]-3-nitro-1,8-naphthalimide (16)

orange solid, yield: 8.6 mg (32%). TLC (MeOH/CH_2_Cl_2_, 3:7, *v/v*): *R*_f_ = 0.40; ^1^H-NMR (MeOH-d_4_, 600.26 MHz): *δ* (ppm) = 8.83 (s, 1H, H_arom_), 8.73 (d, *J* = 7.7 Hz, 1H, H_arom_), 8.51 (dd, *J* = 7.3, 0.9 Hz, 1H, H_arom_), 7.70 (dd, *J* = 8.5, 7.3 Hz, 1H, H_arom_), 4.53 (br s, 1H, CH_carborane_), 4.20 (t, *J* = 6.9 Hz, 2H, CH_2_-N(CO)_2_), 3.90 (t, *J* = 7.0 Hz, 2H, C*H*_2_-NH), 3.17 (t, *J* = 7.0 Hz, 2H, C*H*_2_-NH), 2.78 (t, *J* = 6.9 Hz, 2H, C*H*_2_-N(CH_3_)_2_), 2.58 (dd, *J* = 8.8, 6.9 Hz, 2H, CH_2_-carborane), 2.47 (s, 6H, N(CH_3_)_2_), 2.40 (dd, *J* = 8.7, 6.8 Hz, 2H, NH-C(O)-C*H*_2_), 1.90–1.81 (m, 2H, CH_2_-C*H*_2_-CH_2_), 1.56–1.50 (m, 4H, 2 × CH_2_-C*H*_2_-CH_2_), 1.48–1.40 (m, 2H, CH_2_-C*H*_2_-CH_2_), 3.0–1.5 (m, 10H, B_10_H_10_); ^13^C-NMR (MeOH-d_4_, 150.95 MHz): *δ* (ppm) = 172.73 (1 C, C(O)-NH), 165.05, 164.00 (2 C, C11, C12), 151.45–111.08 (10 C, 10 C_arom_), 76.44 (1 C, C_carborane_), 63.95 (1 C, CH_carborane_), 57.55 (1 C, *C*H_2_-N(CH_3_)_2_), 51.14 (1 C, CH_2_-NH), 45.45 (2 C, 2 × CH_3_), 40.26 (1 C, CH_2_-NH), 38.24 (1 C. *C*H_2_-N(CO)_2_), 35.94–34.20 (2 C, CH_2_-carborane and NH-C(O)-*C*H_2_), 31.76 (CH_2_-*C*H_2_-CH_2_), 30.14 (1 C, CH_2_-*C*H_2_-CH_2_), 27.50 (1 C, CH_2_-*C*H_2_-CH_2_), 27.43 (1 C, CH_2_-*C*H_2_-CH_2_); ^11^B-NMR {^1^H BB} (MeOH-d_4_, 192.59 MHz): *δ* (ppm) = −2.76 (s, 1B, B9), −6.06 (s, 1B, B12), −9.70 (s, 2B, B8,10), −11.90–−13.07 (m, 6B, B3,4,5,6,7,11); UV (99.8% CH_3_CH_2_OH): λ_max_ = 246, 395 nm, λ_min_ = 226, 328 nm, λ_sh_ = 307 nm; FT-IR: *ν*_max_ (cm–^1^) = 2931 (C-H_aliphat_), 2577 (B-H), 1697 (C = O), 1648 (C = O), 723 (B-B); RP-HPLC: *t*_R_ = 21.80 min; APCI-MS: *m/z*: 626 [M + H]^+^, calcd for C_27_H_43_B_10_N_5_O_5_ = 625.

##### 4-[(6-[3–(1,7-Dicarba-closo-dodecaborane-1-yl)propanamido]hexyl)amino]-N-[2-(dimethylamino)ethyl]-3-nitro-1,8-naphthalimide (17)

orange solid, yield: 12 mg (41%). TLC (MeOH/CH_2_Cl_2_, 3:7, *v/v*): *R*_f_ = 0.40; ^1^H-NMR (MeOH-d_4_, 600.26 MHz): *δ* (ppm) = 8.82 (s, 1H, H_arom_), 8.73 (d, *J* = 8.5 Hz, 1H, H_arom_), 8.51 (d, *J* = 7.2 Hz, 1H, H_arom_), 7.70 (t, *J* = 7.9 Hz, 1H, H_arom_), 4.16 (t, *J* = 7.0 Hz, 2H, CH_2_-N(CO)_2_), 3.91 (t, *J* = 7.0 Hz, 2H, C*H*_2_-NH), 3.51 (br s, 1H, CH_carborane_), 3.16 (t, *J* = 7.0 Hz, 2H, C*H*_2_-NH), 2.65 (t, *J* = 7.1 Hz, 2H, C*H*_2_-N(CH_3_)_2_), 2.37 (s, 6H, N(CH_3_)_2_), 2.30–2.25 (m, 4H, NH-C(O)-C*H*_2_ and CH_2_-carborane), 1.87 (dd, *J* = 14.4, 7.2 Hz, 2H, CH_2_-C*H*_2_-CH_2_), 1.57–1.50 (m, 4H, 2 × CH_2_-C*H*_2_-CH_2_), 1.47–1.40 (m, 2H, CH_2_-C*H*_2_-CH_2_), 3.0–1.5 (m, 10H, B_10_H_10_); ^13^C-NMR (MeOH-d_4_, 150.95 MHz): *δ* (ppm) = 173.21 (1 C, C(O)-NH), 165.06, 164.04 (2 C, C11, C12), 151.51–111.27 (10 C, 10 C_arom_), 57.62 (1 C, *C*H_2_-N(CH_3_)_2_), 57.05 (1 C, CH_carborane_), 51.15 (1 C, CH_2_-NH), 45.69 (2 C, 2 × CH_3_), 40.23 (1 C, CH_2_-NH), 38.59 (1 C, *C*H_2_-N(CO)_2_), 36.76–33.51 (2 C, CH_2_-carborane and NH-C(O)-*C*H_2_), 31.77 (1 C, CH_2_-*C*H_2_-CH_2_), 30.14 (1 C, CH_2_-*C*H_2_-CH_2_), 27.50 (1 C, CH_2_-*C*H_2_-CH_2_), 27.42 (1 C, CH_2_-*C*H_2_-CH_2_); ^11^B-NMR {^1^H BB} (MeOH-d_4_, 192.59 MHz): *δ* (ppm) = −4.49 (s, 1B, B5), −9.88–−11.01 (m, 5B, B4,6,9,10,12), −13.55 (s, 2B, B8,11), −15.17 (s, 2B, B2,3); UV (99.8% CH_3_CH_2_OH): λ_max_ = 246, 395 nm, λ_min_ = 225, 328 nm, λ_sh_ = 306 nm; FT-IR: *ν*_max_ (cm–^1^) = 2933 (C-H_aliphat_), 2593 (B-H), 1697 (C = O), 1648 (C = O), 729 (B-B); RP-HPLC: *t*_R_ = 21.70 min; APCI-MS: *m/z*: 626 [M + H]^+^, calcd for C_27_H_43_B_10_N_5_O_5_ = 625.

##### 4-[(6-[3–(1,2-Dicarba-closo-dodecaborane-1-yl)propanamido]hexyl)amino]-3-nitro-N-[2-(N-pyrrolidinyl)ethyl]-1,8-naphthalimide (18)

orange solid, yield: 16.7 mg (47%). TLC (MeOH/CH_2_Cl_2_, 3:7, *v/v*): *R*_f_ = 0.38; ^1^H-NMR (MeOH-d_4_, 600.26 MHz): *δ* (ppm) = 8.83 (s, 1H, H_arom_), 8.75 (d, *J* = 7.9 Hz, 1H, H_arom_), 8.53 (dd, *J* = 7.4, 0.8 Hz, 1H, H_arom_), 7.72 (dd, *J* = 8.4, 7.4 Hz, 1H, H_arom_), 4.53 (br s, 1H, CH_carborane_), 4.31 (t, *J* = 6.3 Hz, 2H, CH_2_-N(CO)_2_), 3.90 (t, *J* = 6.9 Hz, 2H, C*H*_2_-NH), 3.27 (t, *J* = 6.3 Hz, 2H, CH_2_-pyrrolidine), 3.21 (br s, 4H, 2 × N-C*H*_2pyrrolidine_-CH_2_), 3.17 (t, *J* = 7.0 Hz, 2H, C*H*_2_-NH), 2.58 (dd, *J* = 8.8, 6.8 Hz, 2H, CH_2_-carborane), 2.40 (dd, *J* = 8.8, 6.8 Hz, 2H, NH-C(O)-C*H*_2_), 2.03–1.98 (m, 4H, 2 × CH_2_-C*H*_2pyrrolidine_-CH_2_), 1.89–1.83 (m, 2H, CH_2_-C*H*_2_-CH_2_), 1.57–1.50 (m, 4H, 2 × CH_2_-C*H*_2_-CH_2_), 1.46–1.41 (m, 2H, CH_2_-C*H*_2_-CH_2_), 3.0–1.5 (m, 10H, B_10_H_10_); ^13^C-NMR (MeOH-d_4_, 150.95 MHz): *δ* (ppm) = 172.73 (1 C, C(O)-NH), 165.19, 164.12 (2 C, C11, C12), 151.49–110.82 (10 C, 10 C_arom_), 76.44 (1 C, C_carborane_), 63.94 (1 C, CH_carborane_), 55.62 (2 C, 2 × N-*C*H_2pyrrolidine_-CH_2_), 54.40 (1 C, CH_2_-pyrrolidine), 51.12 (1 C, CH_2_-NH), 40.26 (1 C, CH_2_-NH), 38.39 (1 C, *C*H_2_-N(CO)_2_), 35.94–34.20 (2 C, CH_2_-carborane and NH-C(O)-*C*H_2_), 31.72 (1 C, CH_2_-*C*H_2_-CH_2_), 30.12 (1 C, CH_2_-*C*H_2_-CH_2_), 27.48 (1 C, CH_2_-*C*H_2_-CH_2_), 27.41 (1 C, CH_2_-*C*H_2_-CH_2_), 24.11 (2 C, 2 × CH_2_-*C*H_2pyrrolidine_-CH_2_); ^11^B-NMR {^1^H BB} (MeOH-d_4_, 192.59 MHz): *δ* (ppm) = −2.75 (s, 1B, B9), −5.97 (s, 1B, B12), −9.68 (s, 2B, B8,10), −11.77–−13.00 (m, 6B, B3,4,5,6,7,11); UV (99.8% CH_3_CH_2_OH): λ_max_ = 246, 395 nm, λ_min_ = 226, 329 nm, λ_sh_ = 309 nm; FT-IR: *ν*_max_ (cm–^1^) = 2931 (C-H_aliphat_), 2576 (B-H), 1697 (C = O), 1648 (C = O), 722 (B-B); RP-HPLC: *t*_R_ = 22.05 min; APCI-MS: *m/z*: 652 [M + H]^+^, calcd for C_29_H_45_B_10_N_5_O_5_ = 651.

##### 4-[(6-[3–(1,7-Dicarba-closo-dodecaborane-1-yl)propanamido]hexyl)amino]-3-nitro-N-[2-(N-pyrrolidinyl)ethyl]-1,8-naphthalimide (19)

orange solid, yield: 14.1 mg (39%). TLC (MeOH/CH_2_Cl_2_, 3:7, *v/v*): *R*_f_ = 0.40; ^1^H-NMR (MeOH-d_4_, 600.26 MHz): *δ* (ppm) = 8.82 (s, 1H, H_arom_), 8.75 (d, *J* = 8.5 Hz, 1H, H_arom_), 8.52 (d, *J* = 7.3 Hz, 1H, H_arom_), 7.72 (dd, *J* = 8.4, 7.4 Hz, 1H, H_arom_), 4.29 (t, *J* = 6.3 Hz, 2H, CH_2_-N(CO)_2_), 3.90 (t, *J* = 7.0 Hz, 2H, C*H*_2_-NH), 3.50 (br s, 1H, CH_carborane_), 3.22 (t, *J* = 6.4 Hz, 2H, CH_2_-pyrrolidine), 3.17–3.14 (m, 6H, C*H*_2_-NH and 2 × N-C*H*_2pyrrolidine_-CH_2_), 2.30–2.22 (m, 4H, NH-C(O)-C*H*_2_ and CH_2_-carborane), 2.00–1.96 (m, 4H, 2 × CH_2_-C*H*_2pyrrolidine_-CH_2_), 1.90–1.84 (m, 2H, CH_2_-C*H*_2_-CH_2_), 1.57–1.51 (m, 4H, 2 × CH_2_-C*H*_2_-CH_2_), 1.46–1.42 (m, 2H, CH_2_-C*H*_2_-CH_2_), 3.0–1.5 (m, 10H, B_10_H_10_); ^13^C-NMR (MeOH-d_4_, 150.95 MHz): *δ* (ppm) = 173.21 (1 C, C(O)-NH), 165.14, 164.07 (2 C, C11, C12), 151.45–110.85 (10 C, 10 C_arom_), 76.78 (1 C, C_carborane_), 57.06 (1 C, CH_carborane_), 55.56 (2 C, 2 × N-*C*H_2pyrrolidine_-CH_2_), 54.44 (1 C, CH_2_-pyrrolidine), 51.11 (1 C, CH_2_-NH), 40.23 (1 C, CH_2_-NH), 38.57 (1 C, *C*H_2_-N(CO)_2_), 36.77–33.50 (2 C, CH_2_-carborane and NH-C(O)-*C*H_2_), 31.71 (1 C, CH_2_-*C*H_2_-CH_2_), 30.13 (1 C, CH_2_-*C*H_2_-CH_2_), 27.49 (1 C, CH_2_-*C*H_2_-CH_2_), 27.41 (1 C, CH_2_-*C*H_2_-CH_2_), 24.13 (2 C, 2 × CH_2_-*C*H_2pyrrolidine_-CH_2_); ^11^B-NMR {^1^H BB} (MeOH-d_4_, 192.59 MHz): *δ* (ppm) = −4.45 (s, 1B, B5), −9.83–−10.99 (m, 5B, B4,6,9,10,12), −13.54 (s, 2B, B8,11), −15.15 (s, 2B, B2,3); UV (99.8% CH_3_CH_2_OH): λ_max_ = 246, 395 nm, λ_min_ = 226, 328 nm, λ_sh_ = 307 nm; FT-IR: *ν*_max_ (cm–^1^) = 2933 (C-H_aliphat_), 2593 (B-H), 1698 (C = O), 1650 (C = O), 729 (B-B); RP-HPLC: *t*_R_ = 21.91 min; APCI-MS: *m/z*: 652 [M + H]^+^, calcd for C_29_H_45_B_10_N_5_O_5_ = 651.

#### Synthesis of 1,8-naphthalimide derivatives 30–37 modified with carborane cluster via reductive amination

4-[(2-Aminoethyl)amino]-*N*-[2-(dimethylamino)ethyl]-3-nitro-1,8-naphthalimide (**6**), 4-[(2-aminoethyl)amino]-3-nitro-*N*-[2-(*N*-pyrrolidinyl)ethyl]-1,8-naphthalimide (**7**), 4-[(6-aminohexyl)amino]-*N*-[2-(dimethylamino)ethyl]-3-nitro-1,8-naphthalimide (**8**), or 4-[(6-aminohexyl)amino]-3-nitro-*N*-[2-(*N*-pyrrolidinyl)ethyl]-1,8-naphthalimide (**9**) was dissolved in anhydrous MeOH (3 ml per 0.1 mmol), and 2–(1,2-dicarba-*closo*-dodecaborane-1-yl)ethanal (**20**) (1.35 equiv.) or 2–(1,7-dicarba-*closo*-dodecaborane-1-yl)ethanal (**21**) (1.35 equiv.) was added. The reaction mixture was stirred for 24 h at 70 °C under an inert (Ar) atmosphere. Then, NaBH_3_CN (3.5 equiv.) was added to the Schiff base **22**–**29**, and the reaction mixture was stirred for 24 h at rt under an inert (Ar) atmosphere. Subsequently, the solvent was evaporated to dryness under vacuum, and the crude product was purified by column chromatography on silica gel (230–400 mesh) with a gradient of MeOH (3–15%) in CH_2_Cl_2_ as the eluent. Additionally, the purified products **34**–**37** were dissolved in MeOH/CH_2_Cl_2_ (1:39, *v*/*v*) and washed with the same volume of NaHCO_3_ (2.5%) and thrice with water. The organic phase was separated, dried over MgSO_4_, filtered, and evaporated to dryness. Then, the product was purified again by column chromatography under the same conditions as described above.

##### 4-[(2-[(1,2-Dicarba-closo-dodecaborane-1-yl)ethylamino]ethyl)amino]-N-[2-(dimethylamino)ethyl]-3-nitro-1,8-naphthalimide (30)

orange solid, yield: 7.5 mg (40%). TLC (MeOH/CH_2_Cl_2_, 3:7, *v/v*): *R*_f_ = 0.35; ^1^H-NMR (CDCl_3_, 600.26 MHz): *δ* (ppm) = 10.18 (br s, 1H, NH), 9.20 (s, 1H, H_arom_), 8.65 (d, *J* = 7.2 Hz, 1H, H_arom_), 8.59 (d, *J* = 8.3 Hz, 1H, H_arom_), 7.67 (t, *J* = 7.9 Hz, 1H, H_arom_), 4.28 (t, *J* = 7.0 Hz, 2H, CH_2_-N(CO)_2_), 3.99 (dd, *J* = 10.6, 4.8 Hz, 2H, C*H*_2_-NH), 3.88 (br s, 1H, CH_carborane_), 2.97 (t, *J* = 5.5 Hz, 2H, C*H*_2_-NH), 2.88 (t, *J* = 7.3 Hz, 2H, C*H*_2_-CH_2_-carborane), 2.65 (t, *J* = 6.8 Hz, 2H, C*H*_2_-N(CH_3_)_2_), 2.48 (t, *J* = 7.2 Hz, 2H, CH_2_-carborane), 2.36 (s, 6H, N(CH_3_)_2_), 3.0–1.5 (m, 10H, B_10_H_10_); ^13^C-NMR (CDCl_3_, 150.95 MHz): *δ* (ppm) = 163.89, 162.72 (2 C, C11, C12), 150.64–111.23 (10 C, 10 C_arom_), 73.27 (1 C, C_carborane_), 61.57 (1 C, CH_carborane_), 57.07 (1 C, *C*H_2_-N(CH_3_)_2_), 49.77 (1 C, CH_2_-NH), 49.28 (1 C, CH_2_-NH), 48.25 (1 C *C*H_2_-CH_2_-carborane), 45.86 (2 C, 2 × CH_3_), 38.26 (1 C, *C*H_2_-N(CO)_2_), 38.23 (1 C, CH_2_-carborane); ^11^B-NMR {^1^H BB} (CDCl_3_, 192.59 MHz): *δ* (ppm) = −2.20 (s, 1B, B9), −5.49 (s, 1B, B12), −9.34 (s, 2B, B8,10), −11.53–−12.91 (m, 6B, B3,4,5,6,7,11); UV (99.8% CH_3_CH_2_OH): λ_max_ = 247, 397 nm, λ_min_ = 226, 329 nm, λ_sh_ = 305 nm; FT-IR: *ν*_max_ (cm–^1^) = 2922 (C-H_aliphat_), 2579 (B-H), 1695 (C = O), 1649 (C = O), 723 (B-B); RP-HPLC: *t*_R_ = 17.56 min; APCI-MS: *m/z*: 542 [M + H]^+^, calcd for C_22_H_35_B_10_N_5_O_4_ = 541.

##### 4-[(2-[(1,7-Dicarba-closo-dodecaborane-1-yl)ethylamino]ethyl)amino]-N-[2-(dimethylamino)ethyl]-3-nitro-1,8-naphthalimide (31)

orange solid, yield: 8.5 mg (42%). TLC (MeOH/CH_2_Cl_2_, 3:7, *v/v*): *R*_f_ = 0.34; ^1^H-NMR (CDCl_3_, 600.26 MHz): *δ* (ppm) = 10.18 (br 1H, NH), 9.23 (s, 1H, H_arom_), 8.64 (d, *J* = 7.3 Hz, 1H, H_arom_), 8.60 (d, *J* = 8.5 Hz, 1H, H_arom_), 7.66 (t, *J* = 7.9 Hz, 1H, H_arom_), 4.28 (t, *J* = 6.9 Hz, 2H, CH_2_-N(CO)_2_), 3.95 (dd, *J* = 10.7, 4.9 Hz, 2H, C*H*_2_-NH), 2.96–2.92 (m, 3H, CH_carborane_ overlapped with C*H*_2_-NH), 2.73 (t, *J* = 7.8 Hz, 2H, C*H*_2_-CH_2_-carborane), 2.66 (t, *J* = 6.8 Hz, 2H, C*H*_2_-N(CH_3_)_2_), 2.35 (s, 6H, N(CH_3_)_2_), 2.19 (t, *J* = 7.7 Hz, 2H, CH_2_-carborane), 3.0–1.5 (m, 10H, B_10_H_10_); ^13^C-NMR (CDCl_3_, 150.95 MHz): *δ* (ppm) = 163.88, 162.77 (2 C, C11, C12), 150.64–110.99 (10 C, 10 C_arom_), 73.93 (1 C, C_carborane_), 57.06 (1 C, *C*H_2_-N(CH_3_)_2_), 55.21 (1 C, CH_carborane_), 49.79 (1 C, CH_2_-NH), 49.01 (1 C, CH_2_-NH), 48.81 (1 C, *C*H_2_-CH_2_-carborane), 45.86 (2 C, 2CH_3_), 38.26 (1 C, *C*H_2_-N(CO)_2_), 36.92 (1 C, CH_2_-carborane); ^11^B-NMR {^1^H BB} (CDCl_3_, 192.59 MHz): *δ* (ppm) = −4.20 (s, 1B, B5), −9.65–−10.73 (m, 5B, B4,6,9,10,12), −13.51 (s, 2B, B8,11), −15.22 (s, 2B, B2,3); UV (99.8% CH_3_CH_2_OH): λ_max_ = 246, 397 nm, λ_min_ = 225, 328 nm, λ_sh_ = 306 nm; FT-IR: *ν*_max_ (cm–^1^) = 2926 (C-H_aliphat_), 2592 (B-H), 1693 (C = O), 1648 (C = O), 729 (B-B); RP-HPLC: *t*_R_ = 16.17 min; APCI-MS: *m/z*: 542 [M + H]^+^, calcd for C_22_H_35_B_10_N_5_O_4_ = 541.

##### 4-[(2-[(1,2-Dicarba-closo-dodecaborane-1-yl)ethylamino]ethyl)amino]-3-nitro-N-[2-(N-pyrrolidinyl)ethyl]-1,8-naphthalimide (32)

orange solid, yield: 10 mg (43%). TLC (MeOH/CH_2_Cl_2_, 3:7, *v/v*): *R*_f_ = 0.34; ^1^H-NMR (DMSO-d_6_, 600.26 MHz): *δ* (ppm) = 8.86 (d, *J* = 8.1 Hz, 1H, H_arom_), 8.83 (s, 1H, H_arom_), 8.53 (dd, *J* = 7.4, 0.8 Hz, 1H, H_arom_), 7.78 (dd, *J* = 8.3, 7.5 Hz, 1H, H_arom_), 5.17 (br s, 1H, CH_carborane_), 4.15 (t, *J* = 6.8 Hz, 2H, CH_2_-N(CO)_2_), 3.85 (t, *J* = 5.4 Hz, 2H, C*H*_2_-NH), 2.84–2.78 (m, 4H, C*H*_2_-NH overlapped with CH_2_-pyrrolidine), 2.70–2.63 (m, 6H, C*H*_2_-CH_2_-carborane overlapped with 2 × N-C*H*_2pyrrolidine_-CH_2_), 2.41 (t, *J* = 7.5 Hz, 2H, CH_2_-carborane), 1.71 (br s, 4H, 2 × CH_2_-C*H*_2pyrrolidine_-CH_2_), 3.0–1.5 (m, 10H, B_10_H_10_); ^13^C-NMR (DMSO-d_6_, 150.95 MHz): *δ* (ppm) = 163.12, 162.02 (2 C, C11, C12), 149.99–109.16 (10 C, 10 C_arom_), 74.78 (1 C, C_carborane_), 62.78 (1 C, CH_carborane_), 53.78 (2 C, 2 × N-*C*H_2pyrrolidine_-CH_2_), 52.79 (1 C, CH_2_-pyrrolidine), 48.85–47.61 (3 C, 2 × CH_2_-NH and *C*H_2_-CH_2_-carborane), 38.15 (1 C, *C*H_2_-N(CO)_2_), 36.60 (1 C, CH_2_-carborane), 23.11 (2 C, 2 × CH_2_-*C*H_2pyrrolidine_-CH_2_); ^11^B-NMR {^1^H BB} (DMSO-d_6_, 192.59 MHz): *δ* (ppm) = −3.25 (s, 1B, B9), −6.16 (s, 1B, B12), −9.94–−12.93 (m, 8B, B3,4,5,6,7,8,10,11); UV (99.8% CH_3_CH_2_OH): λ_max_ = 246, 397 nm, λ_min_ = 226, 328 nm, λ_sh_ = 307 nm; FT-IR: *ν*_max_ (cm–^1^) = 2958 (C-H_aliphat_), 2579 (B-H), 1694 (C = O), 1649 (C = O), 728 (B-B); RP-HPLC: *t*_R_ = 17.42 min; APCI-MS: *m/z*: 568 [M + H]^+^, calcd for C_24_H_37_B_10_N_5_O_4_ = 567.

##### 4-[(2-[(1,7-Dicarba-closo-dodecaborane-1-yl)ethylamino]ethyl)amino]-3-nitro-N-[2-(N-pyrrolidinyl)ethyl]-1,8-naphthalimide (33)

orange solid, yield: 5.1 mg (40%). TLC (MeOH/CH_2_Cl_2_, 3:7, *v/v*): *R*_f_ = 0.34; ^1^H-NMR (CDCl_3_, 600.26 MHz): *δ* (ppm) = 10.18 (br s, 1H, NH), 9.23 (s, 1H, H_arom_), 8.64 (d, *J* = 7.3 Hz, 1H, H_arom_), 8.59 (d, *J* = 8.5 Hz, 1H, H_arom_), 7.66 (t, *J* = 7.9 Hz, 1H, H_arom_), 4.35 (t, *J* = 7.1 Hz, 2H, CH_2_-N(CO)_2_), 3.95 (dd *J* = 10.7, 4.9 Hz, 2H, C*H*_2_-NH), 2.99–2.92 (m, 3H, CH_carborane_ overlapped with C*H*_2_-NH), 2.88 (br s, 2H, CH_2_-pyrrolidine), 2.80–2.69 (m, 6H, C*H*_2_-CH_2_-carborane overlapped with 2 × N-C*H*_2pyrrolidine_-CH_2_), 2.19 (t, *J* = 7.8 Hz, 2H, CH_2_-carborane), 1.83 (br s, 4H, 2 × CH_2_-C*H*_2pyrrolidine_-CH_2_), 3.0–1.5 (m, 10H, B_10_H_10_); ^13^C-NMR (CDCl_3_, 150.95 MHz): *δ* (ppm) = 163.89, 162.77 (2 C, C11, C12), 150.69–111.0 (10 C, 10 C_arom_), 73.92 (1 C, C_carborane_), 55.21 (1 C, CH_carborane_), 54.55 (2 C, 2 × N-*C*H_2pyrrolidine_-CH_2_), 53.76 (1 C, CH_2_-pyrrolidine), 49.81–48.82 (3 C, 2 × CH_2_-NH and *C*H_2_-CH_2_-carborane), 39.05 (1 C, *C*H_2_-N(CO)_2_), 36.93 (1 C, CH_2_-carborane), 23.72 (2 C, 2 × CH_2_-*C*H_2pyrrolidine_-CH_2_); ^11^B-NMR {^1^H BB} (CDCl_3_, 192.59 MHz): *δ* (ppm) = −4.22 (s, 1B, B5), −9.72–−10.73 (m, 5B, B4,6,9,10,12), −13.52 (s, 2B, B8,11), −15.23 (s, 2B, B2,3); UV (99.8% CH_3_CH_2_OH): λ_max_ = 246, 397 nm, λ_min_ = 226, 328 nm, λ_sh_ = 310 nm; FT-IR: *ν*_max_ (cm–^1^) = 2920 (C-H_aliphat_), 2589 (B-H), 1692 (C = O), 1649 (C = O), 730 (B-B); RP-HPLC: *t*_R_ = 17.48 min; APCI-MS: *m/z*: 568 [M + H]^+^, calcd for C_24_H_37_B_10_N_5_O_4_ = 567.

##### 4-[(6-[(1,2-Dicarba-closo-dodecaborane-1-yl)ethylamino]hexyl)amino]-N-[2-(dimethylamino)ethyl]-3-nitro-1,8-naphthalimide (34)

orange solid, yield: 11.1 mg (28%). TLC (MeOH/CH_2_Cl_2_, 3:7, *v/v*): *R*_f_ = 0.32; ^1^H-NMR (acetone-d_6_, 600.26 MHz): *δ* (ppm) = 9.77 (br s, 1H, NH), 9.02 (s, 1H, H_arom_), 8.92 (dd, *J* = 8.5, 1.0 Hz, 1H, H_arom_), 8.60 (dd, *J* = 7.3, 0.9 Hz, 1H, H_arom_), 7.81 (dd, *J* = 8.5, 7.5 Hz, 1H, H_arom_), 4.93 (br s, 1H, CH_carborane_), 4.20 (t, *J* = 6.9 Hz, 2H, CH_2_-N(CO)_2_), 4.05 (t, *J* = 6.9 Hz, 2H, C*H*_2_-NH), 2.77 (t, *J* = 7.1 Hz, 2H, C*H*_2_-NH), 2.61–2.54 (m, 4H, C*H*_2_-CH_2_-carborane overlapped with C*H*_2_-N(CH_3_)_2_), 2.50 (t, *J* = 7.1, 2H, CH_2_-carborane), 2.27 (s, 6H, N(CH_3_)_2_), 1.93–1.87 (m, 2H, CH_2_-C*H*_2_-CH_2_), 1.55–1.45 (m, 4H, 2 × CH_2_-C*H*_2_-CH_2_), 1.45–1.39 (m, 2H, CH_2_-C*H*_2_-CH_2_), 3.0–1.5 (m, 10H, B_10_H_10_); ^13^C-NMR (acetone-d_6_, 150.95 MHz): *δ* (ppm) = 163.23, 162.19 (2 C, C11, C12), 150.50–110.63 (10 C, 10 C_arom_), 75.05 (1 C, C_carborane_), 61.89 (1 C, CH_carborane_), 56.69 (1 C, *C*H_2_-N(CH_3_)_2_), 50.11–48.20 (3 C, 2 × CH_2_-NH and *C*H_2_-CH_2_-carborane), 45.05 (2 C, 2 × CH_3_), 37.74 (1 C, CH_2_-carborane), 36.68 (1 C, *C*H_2_-N(CO)_2_), 30.64 (1 C, CH_2_-*C*H_2_-CH_2_), 29.46 (1 C, signal of CH_2_-*C*H_2_-CH_2_ overlapped with acetone), 26.58 (1 C, CH_2_-*C*H_2_-CH_2_), 26.31 (1 C, CH_2_-*C*H_2_-CH_2_); ^11^B-NMR {^1^H BB} (acetone-d_6_, 192.59 MHz): *δ* (ppm) = −3.03 (s, 1B, B9), −6.03 (s, 1B, B12), −9.95–−13.21 (m, 8B, B3,4,5,6,7,8,10,11); UV (99.8% CH_3_CH_2_OH): λ_max_ = 246, 395 nm, λ_min_ = 228, 326 nm, λ_sh_ = 307 nm; FT-IR: *ν*_max_ (cm–^1^) = 2934 (C-H_aliphat_), 2572 (B-H), 1697 (C = O), 1650 (C = O), 724 (B-B); RP-HPLC: *t*_R_ = 17.51 min; APCI-MS: *m/z*: 598 [M + H]^+^, calcd for C_26_H_43_B_10_N_5_O_4_ = 597; HRMS (ESI+) 598.4420 [M + H]+, calcd for C_26_H_43_B_10_N_5_O_4_ = 597.4318.

##### 4-[(6-[(1,7-Dicarba-closo-dodecaborane-1-yl)ethylamino]hexyl)amino]-N-[2-(dimethylamino)ethyl]-3-nitro-1,8-naphthalimide (35)

orange solid, yield: 12.3 mg (31%). TLC (MeOH/CH_2_Cl_2_, 3:7, *v/v*): *R*_f_ = 0.31; ^1^H-NMR (MeOH-d_4_, 600.26 MHz): *δ* (ppm) = 8.93 (s, 1H, H_arom_), 8.78 (d, *J* = 8.5 Hz, 1H, H_arom_), 8.56 (d, *J* = 7.3 Hz, 1H, H_arom_), 7.73 (dd, *J* = 8.3, 7.5 Hz, 1H, H_arom_), 4.22 (t, *J* = 7.0 Hz, 2H, CH_2_-N(CO)_2_), 3.93 (t, *J* = 6.9 Hz, 2H, C*H*_2_-NH), 3.54 (br s, 1H, CH_carborane_), 2.71–2.60 (m, 6H, C*H*_2_-NH and C*H*_2_-CH_2_-carborane overlapped with C*H*_2_-N(CH_3_)_2_), 2.38 (s, 6H, N(CH_3_)_2_), 2.22–2.17 (m, 2H, CH_2_-carborane), 1.90–1.84 (m, 2H, CH_2_-C*H*_2_-CH_2_), 1.57–1.50 (m, 4H, 2 × CH_2_-C*H*_2_-CH_2_), 1.46–1.41 (m, 2H, CH_2_-C*H*_2_-CH_2_), 3.0–1.5 (m, 10H, B_10_H_10_); ^13^C-NMR (MeOH-d_4_, 150.95 MHz): *δ* (ppm) = 165.13, 164.13 (2 C, C11, C12), 151.61–111.39 (10 C, 10 C_arom_), 74.93 (1 C, C_carborane_), 57.66 (1 C, *C*H_2_-N(CH_3_)_2_), 57.10 (1 C, CH_carborane_), 51.18–49.85 (3 C, 2 × CH_2_-NH and *C*H_2_-CH_2_-carborane), 45.70 (2 C, 2 × CH_3_), 38.59 (1 C, *C*H_2_-N(CO)_2_), 36.29 (1 C, CH_2_-carborane), 31.70 (1 C, CH_2_-*C*H_2_-CH_2_), 29.69 (1 C, CH_2_-*C*H_2_-CH_2_), 27.67 (1 C, CH_2_-*C*H_2_-CH_2_), 27.50 (1 C, CH_2_-*C*H_2_-CH_2_); ^11^B-NMR {^1^H BB} (MeOH-d_4_, 192.59 MHz): *δ* (ppm) = −4.47 (s, 1B, B5), −9.76–−11.00 (m, 5B, B4,6,9,10,12), −13.52 (s, 2B, B8,11), −15.16 (s, 2B, B2,3); UV (99.8% CH_3_CH_2_OH): λ_max_ = 246, 394 nm, λ_min_ = 227, 328 nm, λ_sh_ = 309 nm; FT-IR: *ν*_max_ (cm–^1^) = 2928 (C-H_aliphat_), 2593 (B-H), 1697 (C = O), 1651 (C = O), 730 (B-B); RP-HPLC: *t*_R_ = 18.24 min; APCI-MS: *m/z*: 598 [M + H]^+^, calcd for C_26_H_43_B_10_N_5_O_4_ = 597; HRMS (ESI+) 598.4412 [M + H]^+^, calcd for C_26_H_43_B_10_N_5_O_4_ = 597.4318.

##### 4-[(6-[(1,2-Dicarba-closo-dodecaborane-1-yl)ethylamino]hexyl)amino]-3-nitro-N-[2-(N-pyrrolidinyl)ethyl]-1,8-naphthalimide (36)

orange solid, yield: 8 mg (31%). TLC (MeOH/CH_2_Cl_2_, 3:7, *v/v*): *R*_f_ = 0.30; ^1^H-NMR (acetone-d_6_, 600.26 MHz): *δ* (ppm) = 9.77 (br s, 1H, NH), 8.99 (s, 1H, H_arom_), 8.90 (d, *J* = 8.5 Hz, 1H, H_arom_), 8.58 (d, *J* = 7.3 Hz, 1H, H_arom_), 7.79 (dd, *J* = 8.3, 7.5 Hz, 1H, H_arom_), 4.93 (br s, 1H, CH_carborane_), 4.25 (t, *J* = 6.9 Hz, 2H, CH_2_-N(CO)_2_), 4.04 (t, *J* = 6.9 Hz, 2H, C*H*_2_-NH), 2.83 (t, *J* = 6.9 Hz, 2H, CH_2_-pyrrolidine), 2.77 (t, *J* = 7.1 Hz, 2H, C*H*_2_-NH), 2.69 (br s, 4H, N-C*H*_2pyrrolidine_-CH_2_), 2.56 (t, *J* = 6.9 Hz, 2H, CH_2_-carborane), 2.51 (t, *J* = 7.1 Hz, 2H, C*H*_2_-CH_2_-carborane), 1.93–1.88 (m, 2H, CH_2_-C*H*_2_-CH_2_), 1.78–1.73 (m, 4H, 2 × CH_2_-C*H*_2pyrrolidine_-CH_2_), 1.54–1.47 (m, 4H, 2 × CH_2_-C*H*_2_-CH_2_), 1.45–1.40 (m, 2H, CH_2_-C*H*_2_-CH_2_), 3.0–1.5 (m, 10H, B_10_H_10_); ^13^C-NMR (acetone-d_6_, 150.95 MHz): *δ* (ppm) = 164.13, 163.09 (2 C, C11, C12), 151.33–111.50 (10 C, 10 C_arom_), 75.90 (1 C, C_carborane_), 62.79 (1 C, CH_carborane_), 54.85 (2 C, 2 × N-*C*H_2pyrrolidine_-CH_2_), 54.07 (1 C, CH_2_-pyrrolidine), 50.95–49.04 (3 C, 2 × CH_2_-NH and *C*H_2_-CH_2_-carborane), 39.42 (1 C, *C*H_2_-N(CO)_2_), 37.51 (1 C, CH_2_-carborane), 31.50 (1 C, CH_2_-*C*H_2_-CH_2_), 30.35 (signal of CH_2_-*C*H_2_-CH_2_ overlapped with acetone), 27.44 (1 C, CH_2_-*C*H_2_-CH_2_), 27.18 (1 C, CH_2_-*C*H_2_-CH_2_), 24.24 (2 C, 2 × CH_2_-*C*H_2pyrrolidine_-CH_2_), ^11^B-NMR {^1^H BB} (acetone-d_6_, 192.59 MHz): *δ* (ppm) = −3.01 (s, 1B, B9), −6.05 (s, 1B, B12), −9.93–−13.17 (m, 8B, B3,4,5,6,7,8,10,11); UV (99.8% CH_3_CH_2_OH): λ_max_ = 246, 394 nm, λ_min_ = 227, 326 nm, λ_sh_ = 307 nm; FT-IR: *ν*_max_ (cm–^1^) = 2926 (C-H_aliphat_), 2580 (B-H), 1697 (C = O), 1651 (C = O), 722 (B-B); RP-HPLC: *t*_R_ = 18.32 min; APCI-MS: *m/z*: 624 [M + H]^+^, calcd for C_28_H_45_B_10_N_5_O_4_ = 623; HRMS (ESI+) 624.4568 [M + H]^+^, calcd for C_28_H_45_B_10_N_5_O_4_ = 623.4475.

##### 4-[(6-[(1,7-Dicarba-closo-dodecaborane-1-yl)ethylamino]hexyl)amino]-3-nitro-N-[2-(N-pyrrolidinyl)ethyl]-1,8-naphthalimide (37)

orange solid, yield: 8.5 mg (33%). TLC (MeOH/CH_2_Cl_2_, 3:7, *v/v*): *R*_f_ = 0.32; ^1^H-NMR (MeOH-d_4_, 600.26 MHz): *δ* (ppm) = 8.79 (s, 1H, H_arom_), 8.72 (d, *J* = 8.1 Hz, 1H, H_arom_), 8.49 (d, *J* = 7.3 Hz, 1H, H_arom_), 7.68 (dd, *J* = 8.4, 7.6 Hz, 1H, H_arom_), 4.17 (t, *J* = 7.1 Hz, 2H, CH_2_-N(CO)_2_), 3.90 (t, *J* = 6.9 Hz, 2H, C*H*_2_-NH), 3.53 (br s, 1H, CH_carborane_), 2.86 (t, *J* = 7.1 Hz, 2H, CH_2_-pyrrolidine), 2.78 (br s, 4H, 2 × N-C*H*_2pyrrolidine_-CH_2_), 2.68–2.60 (m, 4H, CH_2_-carborane overlapped with C*H*_2_-NH), 2.22–2.16 (m, 2H, C*H*_2_-CH_2_-carborane), 1.88–1.82 (m, 6H, 2 × CH_2_-C*H*_2pyrrolidine_-CH_2_ overlapped with CH_2_-C*H*_2_-CH_2_), 1.58–1.50 (m, 4H, 2 × CH_2_-C*H*_2_-CH_2_), 1.47–1.40 (m, 2H, CH_2_-C*H*_2_-CH_2_), 3.0–1.5 (m, 10H, B_10_H_10_); ^13^C-NMR (MeOH-d_4_, 150.95 MHz): *δ* (ppm) = 163.56, 162.51 (2 C, C11, C12), 150.06–109.76 (10 C, 10 C_arom_), 75.55 (1 C, C_carborane_), 55.72 (1 C, CH_carborane_), 53.94 (2 C, 2 × N-*C*H_2pyrrolidine_-CH_2_), 53.14 (1 C, CH_2_-pyrrolidine), 49.75–48.45 (3 C, 2 × CH_2_-NH and *C*H_2_-CH_2_-carborane), 38.06 (1 C, *C*H_2_-N(CO)_2_), 34.87 (1 C, CH_2_-carborane), 30.29 (1 C, CH_2_-*C*H_2_-CH_2_), 28.28 (1 C CH_2_-*C*H_2_-CH_2_), 26.29 (1 C, CH_2_-*C*H_2_-CH_2_), 26.11 (1 C, CH_2_-*C*H_2_-CH_2_), 22.83 (2 C, 2 × CH_2_-*C*H_2pyrrolidine_-CH_2_); ^11^B-NMR {^1^H BB} (MeOH-d_4_, 192.59 MHz): *δ* (ppm) = −4.46 (s, 1B, B5), −9.65–−10.93 (m, 5B, B4,6,9,10,12), −13.49 (s, 2B, B8,11), −15.13 (s, 2B, B2,3); UV (99.8% CH_3_CH_2_OH): λ_max_ = 246, 395 nm, λ_min_ = 225, 326 nm, λ_sh_ = 309 nm; FT-IR: *ν*_max_ (cm–^1^) = 2930 (C-H_aliphat_), 2592 (B-H), 1697 (C = O), 1634 (C = O), 729 (B-B); RP-HPLC: *t*_R_ = 18.08 min; APCI-MS: *m/z*: 624 [M + H]^+^, calcd for C_28_H_45_B_10_N_5_O_4_ = 623; HRMS (ESI+) 624.4566 [M + H]^+^, calcd for C_28_H_45_B_10_N_5_O_4_ = 623.4475.

### Physicochemical investigation with DNA

#### Materials

ct-DNA was purchased from Sigma (USA) and used without purification. Sodium cacodylate (used for the preparation of cacodylate buffer) was purchased from Acros Organics (Geel, Belgium). Water was obtained from a Milli-Q purification system. All experiments were performed with freshly prepared solutions.

#### Preparation of ct-DNA

The ct-DNA was dissolved in H_2_O and reconstituted overnight at 4 °C to dissolve all the material. The molar concentration of ct-DNA was determined from UV–vis spectra based on the molar absorption coefficient (ε) of 6600 M^–1 ^cm^−1^ at 260 nm. The purity of ct-DNA was confirmed using UV–vis spectroscopy by measuring the ratio of absorbance at 260 to 280 nm, and the value was found to be ≥1.8, indicating that DNA was sufficiently free of proteins.

#### T_m_ measurements

The measurements were carried out by adding aliquots of DMSO stock solution of the tested compounds to the buffer solution (pH 7.0, 20 mM, cacodylate buffer, DMSO content of the final solution = 0.33–0.39%). *T*_m_ curves were collected at r = 0.3 (r = [compound]/[ct-DNA]) to assure the dominant binding mode. Thermal melting curves were plotted considering the absorption change at 260 nm as a function of temperature using a GBC Cintra10 UV–VIS spectrometer (Dandenong, Australia) equipped with a GBC Thermocell Peltier Power Supply (Dandenong, Australia) with the use of a 1-cm-path-length cell. The absorbance of the samples was monitored at 260 nm from 35 to 95 °C with a heating rate of 1 °C/min. *T*_m_ values are the midpoints or the transition curves determined from the maximum of the first derivative. Δ*T*_m_ values were calculated by subtracting the *T*_m_ of the free nucleic acid from the *T*_m_ of the sample. All Δ*T*_m_ values reported in the study were measured in triplicate. The error in Δ*T*_m_ was ±0.5 °C.

#### CD Measurements

The measurements were performed by adding aliquots of DMSO stock solution of the tested compounds to the buffer solution (pH 7.0, 20 mM, cacodylate buffer, DMSO content of the final solutions = 0.22–1.30%). Changes in the CD spectrum of ct-DNA upon the addition of compound were measured at different molar ratios r = [compound]/[ct-DNA]. CD spectra were recorded on a JASCO J-815 CD spectrometer with a JASCO PFD-425S Peltier thermostated cell holder (JASCO, Tokyo, Japan) by using a square quartz cuvette of path length 0.5 cm (0.5 ml) in the 230–400 nm region. The CD profiles reported were an average of three successive scans with a scan time of 200 nm per minute and an appropriately corrected baseline. Temperature was maintained at 20 °C during the experiment.

#### UV–vis spectra titration

The measurements were performed by adding aliquots of DMSO stock solution of the tested compounds to the buffer solution (pH 7.4, 20 mM, 50 mM NaCl, Tris–HCl buffer, DMSO content of the final solutions = 0.54–0.65%) to the final concentration 10 µM. The tested compounds were incubated for 5 min, with increasing concentrations ranging from 0 to 15 µM of ct-DNA at 37 °C. Then, the UV–vis absorption spectra between 300 and 500 nm were recorded using a GBC Cintra10 UV–VIS spectrometer equipped with a GBC Thermocell Peltier Power Supply with the use of a 1-cm-path-length cell. The binding constant was calculated according to the following equation^70^:

$$\frac{A_{0}}{A-A_{0}}=\;\frac{\varepsilon_{G}}{\varepsilon_{H-G}-\varepsilon_{G}}+\;\frac{\varepsilon_{G}}{\varepsilon_{H-G}-\varepsilon_{G}}\;\times \;\frac{1}{K_{b}[\mathrm{DNA}]}$$

where K*_b_* is the binding constant; A_0_ and a are absorbances of the free tested compound and the apparent one; ε_G_ and ε_H–G_ are their coefficient, respectively; [DNA] is the concentration of DNA in base pairs. The slope to intercept ratio of the plot between A_0_/A − A_0_ and 1/[DNA] yielded the binding constant.

### Biological investigation

#### Cytotoxicity assay

The cytotoxic properties of the synthesised compounds were evaluated using the human hepatocellular carcinoma cell line (HepG2) and human lung fibroblasts (MRC-5). The HepG2 and MRC-5 cell lines were purchased from American Type Culture Collection (ATCC, Manassas, VA, USA). The HepG2 cells were propagated in Eagle’s minimal essential medium (EMEM, ATCC, Manassas, VA, USA), supplemented with 10% inactivated foetal bovine serum (FBS, Life Technologies, Warsaw, Poland) and 100 units/ml penicillin G with 100 mg/ml streptomycin (Life Technologies, Warsaw, Poland). The cells were incubated at 37 °C in a humidified atmosphere containing 5% CO_2_. Upon reaching 80–90% confluency, the cells were harvested with 0.25% trypsin in 1 mM EDTA (Life Technologies, Warsaw, Poland) and transferred to 96-well microplates at 2 × 10^4^ cells/well. After overnight incubation of cells at 37 °C in a humidified atmosphere containing 5% CO_2_, the culture medium was removed and replaced with a freshly prepared solution of compounds in the culture medium or medium itself as the control group. The stock solutions of each compound were prepared in DMSO at a concentration of 100 mM and were diluted with the growth medium supplemented with 10% FBS to ensure drug dissolution to obtain concentrations ranging from 0.1 to 1000 µM. Cytotoxicity was evaluated using the MTT assay. After incubation with the compounds, the cells were treated with MTT dye solution (5 mg/ml) for 2 h and lysed with a solvent solution containing DMF (45 ml), sodium dodecylsulfate (13.5 g), and distilled water (55 ml). After overnight incubation at 37 °C, optical density at 550 nm, with a reference wavelength of 670 nm, was measured in an ELISA reader (VarioskanLux, Thermo Scientific, Waltham, MA, USA). All determinations were carried out in triplicate. Results were calculated as the percentage of control group viability. The IC_50_ values of the compounds were calculated in an Excel add-in ED50V10.

#### Apoptosis/necrosis assay by flow cytometry

Apoptosis/necrosis assay was carried out by double staining of cells with YO-PRO-1 (Thermo Fisher Scientific) and propidium iodide (PI, Sigma-Aldrich) fluorescent dyes. Briefly, HepG2 cells (2 × 10^5^) were seeded onto 6-well plates. The next day, the cells were treated for 24 h with the analysed compounds at a concentration corresponding to half of their IC_50_ (**34**–2.5 µM, **35**–2.3 µM, **36**–2 µM, **37**–2.3 µM)and total IC_50_ values (**34**–5 µM, **35**–4.5 µM, **36**–4 µM, **37**–4.5 µM). Subsequently, the cells were detached with trypsin (Thermo Fisher Scientific), washed twice with DPBS (1 ml) (Thermo Fisher Scientific), and stained with YO-PRO-1 (0.5 µM) and PI (10 µg/ml) according to the manufacturer’s protocol for 30 min at 37 °C in the dark. Immediately after staining, the cells were analysed with 488 nm excitation using a FACSCalibur flow cytometer (Becton Dickinson), and data were analysed using the FlowJo software (Becton Dickinson).

#### Cell cycle analysis

The HepG2 (5 × 10^5^) cells were seeded onto 6-well cell culture plates and incubated for 24 h in a fully supplemented EMEM (Corning®) with the addition of the analysed compounds at a concentration corresponding to the total IC_50_ values (**34**–5 µM, **35**–4.5 µM, **36**–4 µM, **37**–4.5 µM). The DNA content was determined using flow cytometry with PI (Sigma-Aldrich, Steinheim, Germany) staining. After incubation, the cells were trypsinized and washed twice with 1 ml of PBS (Capricorn). In the next step, the cells were fixed with an ice-cold 80% ethanol. After incubation for 1 h at 4 °C, the fixed cells were washed twice with PBS and suspended in the staining PI/RNAse A/PBS buffer (PI 50 µg/ml, RNAse A 100 µg/ml) for 30 min at 37 °C in the dark. PI (Sigma) fluorescence was measured using a FACSCalibur (Becton Dickinson), and data were analysed using the FlowJo software (Becton Dickinson).

#### Real-time migration potential analysis using the xCELLigence system

Transwell cell migration experiments were performed using an xCELLigence RTCA Analyser (Roche, Switzerland) in CIM plates (ACEA Biosciences, San Diego, CA, USA). Each well consisted of an upper and a lower chamber separated by a microporous polyethylene terephthalate membrane containing randomly distributed 8-µm pores. Prior to the migration assay, the HepG2 cells (2 × 10^4^) were seeded onto 48-well plates containing the growth medium (EMEM) and incubated until 70–80% confluency at 37 °C in a humidified atmosphere containing 5% CO_2_. Subsequently, the cells were treated for 24 h with the tested compounds at final concentrations corresponding to their one-fourth (**34**–1.3 µM, **35**–1.2 µM, **36**–1 µM, **37**–1.2 µM) and half (**34**–2.5 µM, **35**–2.3 µM, **36**–2 µM, **37**–2.3 µM ) of their IC_50_ value. Then, 160 µl of complete growth medium (supplemented with 10% FBS) was added to the lower chamber, and 50 µl of serum-free growth medium was added to the upper chambers of the CIM plate. The plates were incubated at 37 °C under 5% CO_2_ saturation for 1 h prior to insertion into the xCELLigence platform. To initiate a transwell migration experiment, the cells were detached with trypsin (Corning®), resuspended in the serum-free growth medium, and seeded in the upper chamber at a density of 1 × 10^4^ cells in 100 µl per well that was previously treated with the compounds. The impedance of the microelectrode array was monitored and converted to CI. Normalisation values were recorded using the RTCA Software 1.2.1. Readouts were performed every 30 min for 72 h.

#### Oxidative stress measurement in HepG2 cells by flow cytometry

The HepG2 cells were seeded (2 × 10^5^ cells/well) onto 12-well plates and cultured in supplemented EMEM at 37 °C under 5% CO_2_ saturation until 70–80% confluency was reached. Subsequently, the cells were exposed for 24 h to the tested compounds at the final concentration equal to the total IC_50_ values (**34**–5 µM, **35**–4.5 µM, **36**–4 µM, **37**–4.5 µM). The intracellular ROS level was analysed by staining with H_2_DCFDA (ex/em: ∼492–495/517–527 nm) in accordance with the manufacturer’s protocol (Thermo Fisher Scientific, Waltham, MA, USA), wherein ROS trigger the fluorescence. The cells were separated by trypsin (Corning®) and washed twice with DPBS (1 ml, Capricorn). The pelleted cells were suspended in 0.5 ml of DPBS containing H_2_DCFDA at the final concentration of 0.5 µM. In order to eliminate dead cells from analysis, propidium iodide (10 µg/ml) was also added to the staining mix. The cells were then incubated at 37 °C for 30 min in the dark and then analysed with excitation at 488 nm using a FACSCalibur flow cytometer (Becton Dickinson, Franklin Lakes, NJ, USA), and the data were analysed using the FlowJo software (Becton Dickinson).

#### Analysis of GSH level

The HepG2 cells were seeded at a density of 2 × 10^5^ cells/well onto 12-well plates and under the same environmental conditions as the above experiments. The cells were then treated with the tested compounds at the final concentration equal to the total IC_50_ values for 24 h. The cellular GSH level was measured by staining with the nonfluorescent Thiolite Green dye, which emits strong fluorescence after reaction with thiols (ex/em: ∼540/590 nm) according to the manufacturer’s protocol (AAT Bioquest, Sunnyvale, CA, USA). After incubation with the compounds (**34**–5 µM, **35**–4.5 µM, **36**–4 µM, **37**–4.5 µM) the cells were detached with trypsin (Corning®) and washed twice with 1 ml of DPBS (Capricorn). Subsequently, the cells were suspended in a staining solution (50 µM) and incubated at 37 °C in a 5% CO_2_ environment for 30 min. After staining, the fluorescence intensity was analysed using a FACSCalibur flow cytometer with excitation at 488 nm, and the data were analysed using the FlowJo software (Becton Dickinson).

#### MMP measurement

The HepG2 cells were seeded at the density of 2 × 10^5^ cells/well onto 12-well plates and cultured with EMEM at 37 °C under 5% CO_2_ saturation until 70–80% confluency was reached. Subsequently, the cells were treated for 3, 6, and 24 h with the tested compounds at the final concentration corresponding to the total IC_50_ values (**34**–5 µM, **35**–4.5 µM, **36**–4 µM, **37**–4.5 µM). After incubation, the cells were detached with trypsin (Corning®) and washed twice with 1 ml of DPBS (Capricorn), and alterations in ΔΨm were analysed using the MMP-sensitive cationic dye 5,5′,6,6′-tetrachloro-1,1′,3,3′-tetraethylbenzimidazolylcarbocyanine iodide (JC-1; Thermo Fisher Scientific), which can selectively enter mitochondria and change colour from red to green as the membrane potential decreases. The cells were stained with 2.5 µM JC-1 for 30 min at 37 °C in the dark. The maximum emission wavelengths of JC-1 monomers and “J-aggregates” were ∼ 525 (FL-1 channel) and ∼ 590 (FL-2 channel) nm, respectively. To create a strong, single positive green fluorescence signal, 3 µM FCCP was used. The mean fluorescence intensity representing the intracellular MMP level was analysed after incubation with the dye, with excitation at 488 nm using a FACSCalibur flow cytometer (Becton Dickinson), and the data were analysed using the FlowJo software (Becton Dickinson).

#### Lipid peroxidation measurement

The HepG2 cells were seeded at a density of 2 × 10^5^ cells/well onto 12-well plates and cultured under the same environmental conditions as the above experiments. Then, the cells were treated for 24 h with the tested compounds at the final concentration corresponding to the total IC_50_ values. The cells were then detached with trypsin (Corning®) and washed twice with 1 ml of DPBS (Capricorn), and intracellular oxidation of lipids was analysed by staining with the BODIPY® 581/591 C11 reagent according to the manufacturer’s protocol (Thermo Fisher Scientific, Waltham, MA, USA). Upon oxidation in living cells, the reagent shifts the fluorescence emission peak from 590 (red) to 510 nm (green). The cells were analysed after 30 min incubation in a 10 µM staining solution at 488 nm excitation using a FACSCalibur flow cytometer (Becton Dickinson, Franklin Lakes, NJ, USA), and the data were analysed using the FlowJo software (Becton Dickinson). The ratios of the signal from the 590 to 510 channels were used to determine lipid peroxidation in cells.

#### Intracellular Fe^2+^ level determination

The HepG2 cells were seeded at the density of 2 × 10^5^ cells/well onto 12-well plates and cultured with supplemented EMEM at 37 °C under 5% CO_2_ saturation until 70–80% confluency was reached. Then, the cells were treated with compounds **34**–**37** at the final concentration corresponding to the total IC_50_ values (**34**–5 µM, **35**–4.5 µM, **36**–4 µM, **37**–4.5 µM) for 24 h. Subsequently, the cells were detached with trypsin (Corning®), washed with 1 ml DPBS (Capricorn), and then resuspended in the growth medium with 5 µM FeRhoNox™-1 (Goryo Chemical) for 1 h. The dye specifically detects labile Fe^2+^ ions via orange (red) fluorescence, irreversibly fluoresces upon reaction with Fe^2+^ ions, but does not react with other ions. The cells were analysed after incubation with the dye with excitation at 488 nm using a FACSCalibur flow cytometer (Becton Dickinson), and the data were analysed using the FlowJo software (Becton Dickinson). To create a strong, single, positive red fluorescence signal, 3 mM *N*-acetylcysteine (2 h incubation; Sigma-Aldrich) was used.

#### Ca^2+^ homeostasis determination

The HepG2 cells were incubated in the presence of the tested compounds at the final concentration corresponding to the total IC_50_ values (**34**–5 µM, **35**–4.5 µM, **36**–4 µM, **37**–4.5 µM) for 24 h. Ca^2+^ released was determined by the selective dye Calcium Rhod-2 AM (Thermo Fisher Scientific), which binds specifically to Ca^2+^ ions that are related to an increase in dihydrorhod-2 AM concentration, and thus, an enhancement in fluorescence intensity occurred. After treatment, the cells were detached with trypsin (Corning®), washed with 1 ml of DPBS (Capricorn), and then resuspended in EMEM with 2.5 µM Rhod-2 AM. All the samples were incubated for 15 min at 37 °C in the dark, and fluorescence intensity was then analysed at an excitation/emission wavelength of 552/581 nm. The cells were analysed after incubation with the dye with excitation at 488 nm using a FACSCalibur flow cytometer (Becton Dickinson), and the data were analysed using the FlowJo software (Becton Dickinson).

#### Lysotracker red and acridine orange staining

To analyse the condition of the lysosomes, the cells (2 × 10^5^) were stained with the 100 nM LysoTracker™ Deep Red (Thermo Fisher Scientific) fluorescent dye for 30 min under growth conditions. The HepG2 and MRC-5 cells were treated with the tested compounds for 3 h.Final concentration of the compounds for HepG2 cells were as follows: **34**–5 µM, **35**–4.5 µM, **36**–4 µM, **37**–4.5 µM, whereas for MRC-5 were: **34**–2.1 µM, **35**–2 µM, **36**–2.3 µM, **37**–2.3 µM. Subsequently, the cells were trypsinized, rinsed with 1 ml of DPBS (Capricorn), and then incubated with the dye. Then, cytometric analysis of the stained cells was performed at an excitation/emission wavelength of 647/668 nm.

Lysosomal permeabilization was tested using the acridine orange (AO) dye. After treatment with the compounds, the cell pellets were suspended in 500 µl of DBPS containing AO (Sigma-Aldrich) at the final concentration of 0.5 µg/ml. After 15 min of staining at 37 °C in the dark, the cells were washed with DPBS to remove the unbound dye and resuspended in 500 µl of DPBS. Fluorescence intensity was analysed at an excitation wavelength of 488 nm, and two emission spectra were acquired at 505–550 and 600–650 nm for green and red fluorescence, respectively. The cells were analysed after incubation with the dye, with excitation at 488 nm, using a FACSCalibur flow cytometer (Becton Dickinson), and the data were analysed using the FlowJo software (Becton Dickinson).

#### Confocal microscopy analysis of lysosome integrity and nuclei morphology using AO staining

The HepG2 or MCR-5 cells were seeded onto a 10-well CELLview Slide (34 mm^2^, Greiner Bio-One GmbH, Austria) at the density of 1.5 × 10^4^ cells/well and grown in 200 µl of supplemented EMEM at 37 °C in a 5% CO_2_ atmosphere. After 24 h, the cells were treated with compounds **34**–**37** at the final concentration: **34**–5 µM, **35**–4.5 µM, **36**–4 µM, **37**–4.5 µM for 3 h. The untreated cells and cells incubated with DMSO were used as controls for the experiments. The final amount of DMSO was 5 and 2.3 µl for HepG2 and MRC-5, respectively, which correlated with the highest volume of the compound with the lowest IC_50_ value. After incubation, the cells were stained with AO at the final concentration of 1 µg/ml for 15 min under growth conditions. The medium was then removed, and the cells were rinsed with PBS, placed in FluoroBrite DMEM (Thermo Fisher Scientific, USA), and immediately examined under a Leica TCS SP5 II confocal microscope at 37 °C using an environmental cell culture chamber and an HC PL APO 63×/1.2 water-immersion objective. Sequentially scanned Z-stack images were collected at an excitation wavelength of 488 nm, and two emission spectra were acquired at 505–550 and 600–650 nm for green and red fluorescence, respectively. Image acquisition was carried out using the Leica LAS AF 2.7.3 software. Image processing and fluorescence intensity analysis were performed using the Leica LAS X 3.3.3 software with 2D or 3D deconvolution modules.

#### Human Topoisomerase IIα relaxation assay

The Human Topoisomerase IIα Relaxation Assay Kit (Cat. No. HTR 201) was purchased from Inspiralis (Norwich, UK). The Topo IIα inhibition assay was performed according to the manufacturer’s instructions. Briefly, the reaction mixture (30 µl) containing the tested compound (100 µM) dissolved in DMSO (whose final concentration of 2% did not influence the Topo IIα activity), supercoiled pBR322 (0.5 µg) in 1X Assay Buffer, and ATP (1 mM) was incubated at 37 °C for 15 min. Then, Topo IIα (1 U) was added, and the reaction mixture was incubated at 37 °C for an additional 45 min. The reaction was terminated by the addition of the STEB buffer (40% (*w/v*) sucrose, Tris–HCl (100 mM, pH 8), EDTA (1 mM), bromophenol blue (0.5 mg/ml)). The products were analysed by electrophoresis using agarose gel (1%) in TAE buffer at 70 V for 2 h, followed by ethidium bromide staining. For the most active compounds **34**–**37**, the reaction was repeated at concentrations of 1, 5, and 10 µM. The percentage of Topo IIα activity inhibition was calculated by densitometric quantification (Quantity One software, Bio-Rad). The occurrence of a band representing the supercoiled DNA on an agarose gel indicated the inhibition of enzyme activity.

#### DNA unwinding assay

The DNA Unwinding Assay Kit (Cat no. DUKSR001) was purchased from Inspiralis (Norwich, UK). This assay is based on two reactions with different DNA substrates (relaxed or supercoiled pBR 322) and is used to investigate whether a compound intercalates into the DNA double-helix or binds in the minor groove, leading to unwinding of the DNA. In the first reaction, 0.5 µg of relaxed plasmid was incubated for 15 min with compounds **34**, **35**, **36**, or **37** at a concentration of 10 µM in 2X assay buffer. After incubation, 2.5 U of wheat germ topoisomerase I (Topo I) was added, and the samples were incubated for 45 min at 37 °C. Then, 20 µl of water and 50 µl of butanol were added, vortexed briefly, and centrifuged. After extraction, the aqueous layer was mixed with an equal volume of STEB buffer (40% (*w/v*) sucrose, Tris–HCl (100 mM, pH 8), EDTA (1 mM), bromophenol blue (0.5 mg/ml)), extracted with chloroform/isoamyl alcohol (24:1), and analysed on 1% agarose gel in TAE buffer at 70 V for 2 h, followed by ethidium bromide staining. The second reaction was performed as described in the case of relaxed DNA but using a supercoiled plasmid as a substrate for Topo I.

## Supplementary Material

Supplemental Material
