# Supplementary material for: Carboranyl-1,8-naphthalimide intercalators induce lysosomal membrane permeabilization and ferroptosis in cancer cell lines
Source: J Enzyme Inhib Med Chem. 2023 Jan 30;38(1):2171028. doi: 10.1080/14756366.2023.2171028 (PMC9888480; doi:10.1080/14756366.2023.2171028)
Supplement: Supplemental Material [file IENZ_A_2171028_SM4507.pdf]

Electronic Supporting Information

Carboranyl-1,8-naphthalimide intercalators induce lysosomal membrane permeabilization and ferroptosis in cancer cell lines

Sebastian Rykowski[a,#], Dorota Gurda-Woźna[b,#], Agnieszka Fedoruk-Wyszomirska[b], Marta Orlicka-Płocka[b], Aleksandra Kowalczyk[c], Paweł Stączek[c], Marta Denel-Bobrowska[a], Katarzyna Biniek-Antosiak[b], Wojciech Rypniewski[b], Eliza Wyszko[b,*], Agnieszka B. Olejniczak[a,*]

[a]Institute of Medical Biology, Polish Academy of Sciences, 106 Lodowa St., Łódź 93-232, Poland

[b]Institute of Bioorganic Chemistry, Polish Academy of Sciences, 12/14 Z. Noskowskiego St., Poznan 61-704, Poland

[c]Department of Molecular Microbiology, Faculty of Biology and Environmental Protection, University of Lodz, 12/16 Banacha St., Łódź 90-237, Poland

Table of contents

Figure S1. ^{1}H-NMR spectrum of **5**. **7**
Figure S2. ^{1}H-NMR spectrum of **6**. **8**
Figure S3. ^{1}H-NMR spectrum of **7**. **9**
Figure S4. ^{1}H-NMR spectrum of **8**. **10**
Figure S5. ^{1}H-NMR spectrum of **9**. **11**
Figure S6. ^{1}H-NMR spectrum of **12**. **12**
Figure S7. ^{13}C-NMR spectrum of **12**. **13**
Figure S8. ^{11}B-NMR {^{1}H BB} spectrum of **12**. **14**
Figure S9. UV spectrum of **12**. **15**
Figure S10. IR spectrum of **12**. **16**
Figure S11. HPLC analysis of **12**. **17**
Figure S12. MS spectrum of **12**. **18**
Figure S13. ^{1}H-NMR spectrum of **13**. **19**
Figure S14. ^{13}C-NMR spectrum of **13**. **20**
Figure S15. ^{11}B-NMR {^{1}H BB} spectrum of **13**. **21**
Figure S16. UV spectrum of **13**. **22**
Figure S17. IR spectrum of **13**. **23**
Figure S18. HPLC analysis of **13**. **24**
Figure S19. MS spectrum of **13**. **25**
Figure S20. ^{1}H-NMR spectrum of **14**. **26**
Figure S21. ^{13}C-NMR spectrum of **14**. **27**
Figure S22. ^{11}B-NMR {^{1}H BB} spectrum of **14**. **28**
Figure S23. UV spectrum of **14**. **29**
Figure S24. IR spectrum of **14**. **30**
Figure S25. HPLC analysis of **14**. **31**
Figure S26. MS spectrum of **14**. **32**
Figure S27. ^{1}H-NMR spectrum of **15**. **33**
Figure S28. ^{13}C-NMR spectrum of **15**. **34**
Figure S29. ^{11}B-NMR {^{1}H BB} spectrum of **15**. **35**
Figure S30. UV spectrum of **15**. **36**
Figure S31. IR spectrum of **15**. **37**
Figure S32. HPLC analysis of **15**. **38**
Figure S33. MS spectrum of **15**. **39**
Figure S34. ^{1}H-NMR spectrum of **16**. **40**
Figure S35. ^{13}C-NMR spectrum of **16**. **41**
Figure S36. ^{11}B-NMR {^{1}H BB} spectrum of **16**. **42**
Figure S37. UV spectrum of **16**. **43**
Figure S38. IR spectrum of **16**. **44**
Figure S39. HPLC analysis of **16**. **45**
Figure S40. MS spectrum of **16**. **46**
Figure S41. ^{1}H-NMR spectrum of **17**. **47**
Figure S42. ^{13}C-NMR spectrum of **17**. **48**
Figure S43. ^{11}B-NMR {^{1}H BB} spectrum of **17**. **49**
Figure S44. UV spectrum of **17**. **50**
Figure S45. IR spectrum of **17**. **51**
Figure S46. HPLC analysis of **17**. **52**
Figure S47. MS spectrum of **17**. **53**
Figure S48. ^{1}H-NMR spectrum of **18**. **54**

Figure S49. ^{13}C-NMR spectrum of **18**. **55**
Figure S50. ^{11}B-NMR {^{1}H BB} spectrum of **18**. **56**
Figure S51. UV spectrum of **18**. **57**
Figure S52. IR spectrum of **18**. **58**
Figure S53. HPLC analysis of **18**. **59**
Figure S54. MS spectrum of **18**. **60**
Figure S55. ^{1}H-NMR spectrum of **19**. **61**
Figure S56. ^{13}C-NMR spectrum of **19**. **62**
Figure S57. ^{11}B-NMR {^{1}H BB} spectrum of **19**. **63**
Figure S58. UV spectrum of **19**. **64**
Figure S59. IR spectrum of **19**. **65**
Figure S60. HPLC analysis of **19**. **66**
Figure S61. MS spectrum of **19**. **67**
Figure S62. ^{1}H-NMR spectrum of **30**. **68**
Figure S63. ^{13}C-NMR spectrum of **30**. **69**
Figure S64. ^{11}B-NMR {^{1}H BB} spectrum of **30**. **70**
Figure S65. UV spectrum of **30**. **71**
Figure S66. IR spectrum of **30**. **72**
Figure S67. HPLC analysis of **30**. **73**
Figure S68. MS spectrum of **30**. **74**
Figure S69. ^{1}H-NMR spectrum of **31**. **75**
Figure S70. ^{13}C-NMR spectrum of **31**. **76**
Figure S71. ^{11}B-NMR {^{1}H BB} spectrum of **31**. **77**
Figure S72. UV spectrum of **31**. **78**
Figure S73. IR spectrum of **31**. **79**
Figure S74. HPLC analysis of **31**. **80**
Figure S75. MS spectrum of **31**. **81**
Figure S76. ^{1}H-NMR spectrum of **32**. **82**
Figure S77. ^{13}C-NMR spectrum of **32**. **83**
Figure S78. ^{11}B-NMR {^{1}H BB} spectrum of **32**. **84**
Figure S79. UV spectrum of **32**. **85**
Figure S80. IR spectrum of **32**. **86**
Figure S81. HPLC analysis of **32**. **87**
Figure S82. MS spectrum of **32**. **88**
Figure S83. ^{1}H-NMR spectrum of **33**. **89**
Figure S84. ^{13}C-NMR spectrum of **33**. **90**
Figure S85. ^{11}B-NMR {^{1}H BB} spectrum of **33**. **91**
Figure S86. UV spectrum of **33**. **92**
Figure S87. IR spectrum of **33**. **93**
Figure S88. HPLC analysis of **33**. **94**
Figure S89. MS spectrum of **33**. **95**
Figure S90. ^{1}H-NMR spectrum of **34**. **96**
Figure S91. ^{13}C-NMR spectrum of **34**. **97**
Figure S92. ^{11}B-NMR {^{1}H BB} spectrum of **34**. **98**
Figure S93. UV spectrum of **34**. **99**
Figure S94. IR spectrum of **34**. **100**
Figure S95. HPLC analysis of **34**. **101**
Figure S96. MS spectrum of **34**. **102**
Figure S97. HRMS spectrum of **34**. **103**
Figure S98. ^{1}H-NMR spectrum of **35**. **104**

Figure S99. ^{13}C-NMR spectrum of **35**. **105**
Figure S100. ^{11}B-NMR {^{1}H BB} spectrum of **35**. **106**
Figure S101. UV spectrum of **35**. **107**
Figure S102. IR spectrum of **35**. **108**
Figure S103. HPLC analysis of **35**. **109**
Figure S104. MS spectrum of **35**. **110**
Figure S105. HRMS spectrum of **35**. **111**
Figure S106. ^{1}H-NMR spectrum of **36**. **112**
Figure S107. ^{13}C-NMR spectrum of **36**. **113**
Figure S108. ^{11}B-NMR {^{1}H BB} spectrum of **36**. **114**
Figure S109. UV spectrum of **36**. **115**
Figure S110. IR spectrum of **36**. **116**
Figure S111. HPLC analysis of **36**. **117**
Figure S112. MS spectrum of **36**. **118**
Figure S113. HRMS spectrum of **36**. **119**
Figure S114. ^{1}H-NMR spectrum of **37**. **120**
Figure S115. ^{13}C-NMR spectrum of **37**. **121**
Figure S116. ^{11}B-NMR {^{1}H BB} spectrum of **37**. **122**
Figure S117. UV spectrum of **37**. **123**
Figure S118. IR spectrum of **37**. **124**
Figure S119. HPLC analysis of **37**. **125**
Figure S120. MS spectrum of **37**. **126**
Figure S121. HRMS spectrum of **37**. **127**
Figure S122. Melting curves of ct-DNA upon addition of **12-15** (c (ct-DNA) = 2 × 10^{-5} mol dm^{-3}) at molar ratio r = 0.3 (r = [compound]/[ct-DNA]), sodium cacodylate buffer (pH 7.0, 20 mM). **128**
Figure S123. Melting curves of ct-DNA upon addition of **16-19** (c (ct-DNA) = 2 × 10^{-5} mol dm^{-3}) at molar ratio r = 0.3 (r = [compound]/[ct-DNA]), sodium cacodylate buffer (pH 7.0, 20 mM). **129**
Figure S124. Melting curves of ct-DNA upon addition of **30-33** (c (ct-DNA) = 2 × 10^{-5} mol dm^{-3}) at molar ratio r = 0.3 (r = [compound]/[ct-DNA]), sodium cacodylate buffer (pH 7.0, 20 mM). **130**
Figure S125. Melting curves of ct-DNA upon addition of **34-37** (c (ct-DNA) = 2 × 10^{-5} mol dm^{-3}) at molar ratio r = 0.3 (r = [compound]/[ct-DNA]), sodium cacodylate buffer (pH 7.0, 20 mM). **131**
Figure S126. Changes in the CD spectrum of ct-DNA upon addition of **12** (c (ct-DNA) = 4 × 10^{-5} mol dm^{-3}) (left) and **13** (c (ct-DNA) = 4 × 10^{-5} mol dm^{-3}) (right) at different molar ratios r = [compound]/[ct-DNA], sodium cacodylate buffer (pH 7.0, 20 mM). **132**
Figure S127. Changes in the CD spectrum of ct-DNA upon addition of **14** (c (ct-DNA) = 4 × 10^{-5} mol dm^{-3}) (left) and **15** (c (ct-DNA) = 4 × 10^{-5} mol dm^{-3}) (right) at different molar ratios r = [compound]/[ct-DNA], sodium cacodylate buffer (pH 7.0, 20 mM). **133**
Figure S128. Changes in the CD spectrum of ct-DNA upon addition of **16** (c (ct-DNA) = 4 × 10^{-5} mol dm^{-3}) (left) and **17** (c (ct-DNA) = 4 × 10^{-5} mol dm^{-3}) (right) at different molar ratios r = [compound]/[ct-DNA], sodium cacodylate buffer (pH 7.0, 20 mM). **134**
Figure S129. Changes in the CD spectrum of ct-DNA upon addition of **18** (c (ct-DNA) = 4 × 10^{-5} mol dm^{-3}) (left) and **19** (c (ct-DNA) = 4 × 10^{-5} mol dm^{-3}) (right) at different molar ratios r = [compound]/[ct-DNA], sodium cacodylate buffer (pH 7.0, 20 mM). **135**
Figure S130. Changes in the CD spectrum of ct-DNA upon addition of **30** (c (ct-DNA) = 4 × 10^{-5} mol dm^{-3}) (left) and **31** (c (ct-DNA) = 4 × 10^{-5} mol dm^{-3}) (right) at different molar ratios r = [compound]/[ct-DNA], sodium cacodylate buffer (pH 7.0, 20 mM). **136**

Figure S131. Changes in the CD spectrum of ct-DNA upon addition of **32** (c (ct-DNA) = 4 × 10^{-5} mol dm^{-3}) (left) and **33** (c (ct-DNA) = 4 × 10^{-5} mol dm^{-3}) (right) at different molar ratios r = [compound]/[ct-DNA], sodium cacodylate buffer (pH 7.0, 20 mM). **137**
Figure S132. Changes in the CD spectrum of ct-DNA upon addition of **34** (c (ct-DNA) = 4 × 10^{-5} mol dm^{-3}) (left) and **35** (c (ct-DNA) = 4 × 10^{-5} mol dm^{-3}) (right) at different molar ratios r = [compound]/[ct-DNA], sodium cacodylate buffer (pH 7.0, 20 mM). **138**
Figure S133. Changes in the CD spectrum of ct-DNA upon addition of **36** (c (ct-DNA) = 4 × 10^{-5} mol dm^{-3}) (left) and **37** (c (ct-DNA) = 4 × 10^{-5} mol dm^{-3}) (right) at different molar ratios r = [compound]/[ct-DNA], sodium cacodylate buffer (pH 7.0, 20 mM). **139**
Figure S134. UV-vis absorption spectra of compound **12** (10 μM) in the presence of increasing amount of cf-DNA (0-15 μM) (left). The plot of $A_0/A-A_0$ versus 1/[DNA] yielded the binding constant (right). **140**
Figure S135. UV-vis absorption spectra of compound **13** (10 μM) in the presence of increasing amount of ct-DNA (0-15 μM) (left). The plot of $A_0/A-A_0$ versus 1/[DNA] yielded the binding constant (right). **141**
Figure S136. UV-vis absorption spectra of compound **14** (10 μM) in the presence of increasing amount of ct-DNA (0-15 μM) (left). The plot of $A_0/A-A_0$ versus 1/[DNA] yielded the binding constant (right). **142**
Figure S137. UV-vis absorption spectra of compound **15** (10 μM) in the presence of increasing amount of ct-DNA (0-15 μM) (left). The plot of $A_0/A-A_0$ versus 1/[DNA] yielded the binding constant (right). **143**
Figure S138. UV-vis absorption spectra of compound **16** (10 μM) in the presence of increasing amount of ct-DNA (0-15 μM) (left). The plot of $A_0/A-A_0$ versus 1/[DNA] yielded the binding constant (right). **144**
Figure S139. UV-vis absorption spectra of compound **17** (10 μM) in the presence of increasing amount of ct-DNA (0-15 μM) (left). The plot of $A_0/A-A_0$ versus 1/[DNA] yielded the binding constant (right). **145**
Figure S140. UV-vis absorption spectra of compound **18** (10 μM) in the presence of increasing amount of ct-DNA (0-15 μM) (left). The plot of $A_0/A-A_0$ versus 1/[DNA] yielded the binding constant (right). **146**
Figure S141. UV-vis absorption spectra of compound **19** (10 μM) in the presence of increasing amount of ct-DNA (0-15 μM) (left). The plot of $A_0/A-A_0$ versus 1/[DNA] yielded the binding constant (right). **147**
Figure S142. UV-vis absorption spectra of compound **30** (10 μM) in the presence of increasing amount of ct-DNA (0-15 μM) (left). The plot of $A_0/A-A_0$ versus 1/[DNA] yielded the binding constant (right). **148**
Figure S143. UV-vis absorption spectra of compound **31** (10 μM) in the presence of increasing amount of ct-DNA (0-15 μM) (left). The plot of $A_0/A-A_0$ versus 1/[DNA] yielded the binding constant (right). **149**
Figure S144. UV-vis absorption spectra of compound **32** (10 μM) in the presence of increasing amount of ct-DNA (0-15 μM) (left). The plot of $A_0/A-A_0$ versus 1/[DNA] yielded the binding constant (right). **150**
Figure S145. UV-vis absorption spectra of compound **34** (10 μM) in the presence of increasing amount of ct-DNA (0-15 μM) (left). The plot of $A_0/A-A_0$ versus 1/[DNA] yielded the binding constant (right). **151**
Figure S146. UV-vis absorption spectra of compound **35** (10 μM) in the presence of increasing amount of ct-DNA (0-15 μM) (left). The plot of $A_0/A-A_0$ versus 1/[DNA] yielded the binding constant (right). **152**

Figure S147. UV-vis absorption spectra of compound **36** (10 μM) in the presence of increasing amount of ct-DNA (0-15 μM) (left). The plot of $A_0/A-A_0$ versus 1/[DNA] yielded the binding constant (right). **153**
Figure S148. UV-vis absorption spectra of compound **37** (10 μM) in the presence of increasing amount of ct-DNA (0-15 μM) (left). The plot of $A_0/A-A_0$ versus 1/[DNA] yielded the binding constant (right). **154**
Figure S149. Apoptosis/necrosis analysis in HepG2 cells after 24 h treatment with compounds **34-37**. **155**
Figure S150. Cell cycle analysis using flow cytometry in HepG2 cells. **156**
Figure S151. Analysis of reactive oxygen species (ROS) production in HepG2 cells after 3 and 6 h of treatment with the compounds **34-37**. **157**
Figure S152. Mitochondrial membrane potential (MMP) analysis in HepG2 cells treated with compounds **34-37**. **158**
Figure S153. Human Topoisomerase IIα relaxation assay in the presence of conjugates modified with carborane-1,8-naphthalimides **34-37** at the concentration of 100 µM. **159**
Figure S154. Inhibition of the relaxation activity of human Topoisomerase IIα in the presence of compounds **34-37** at the concentrations of 1, 5, and 10 µM. **160**
Table S1. Crystallographic data. **161**

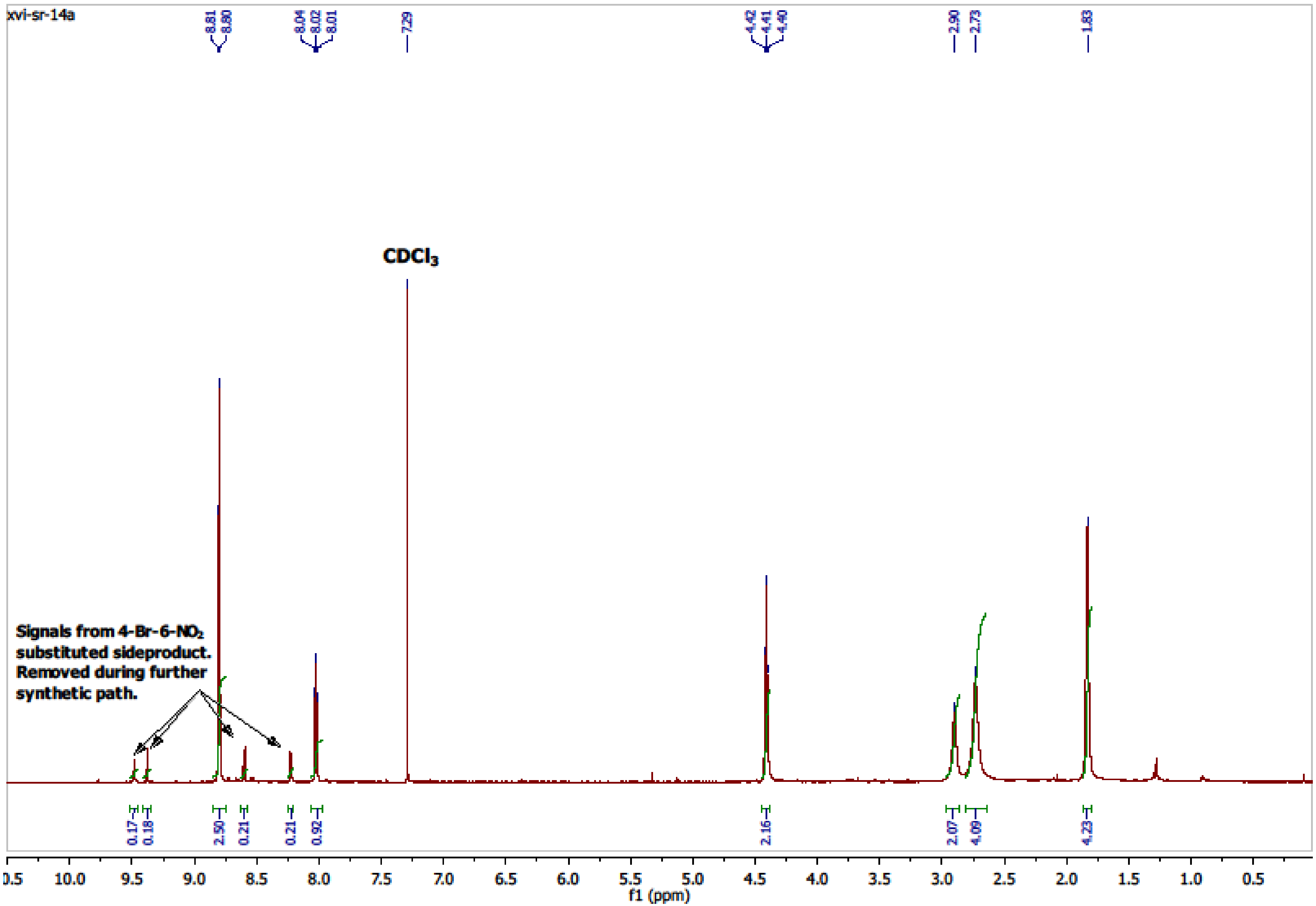

Figure S1. ^{1}H-NMR spectrum of **5**.

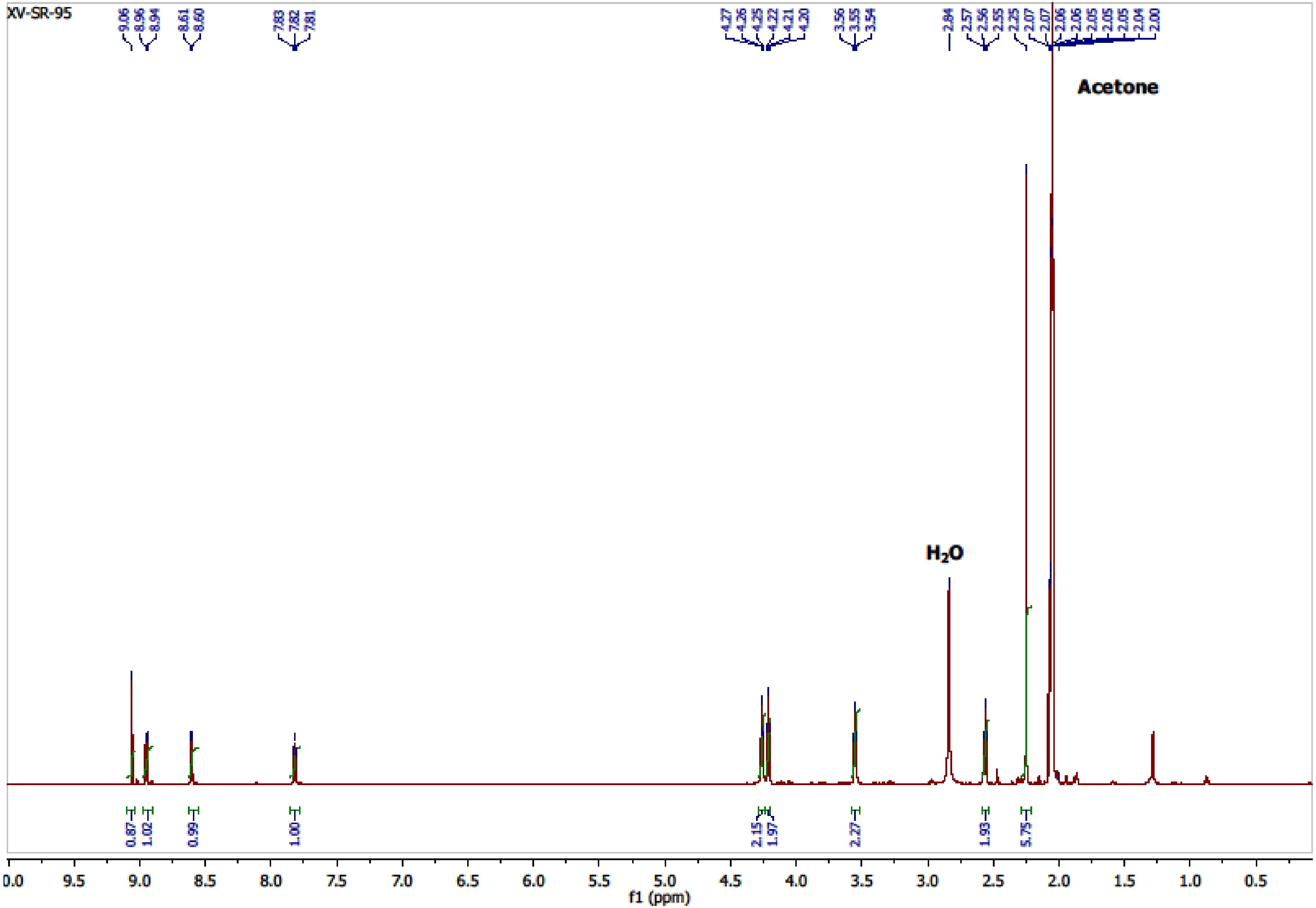

Figure S2. ^{1}H-NMR spectrum of **6**.

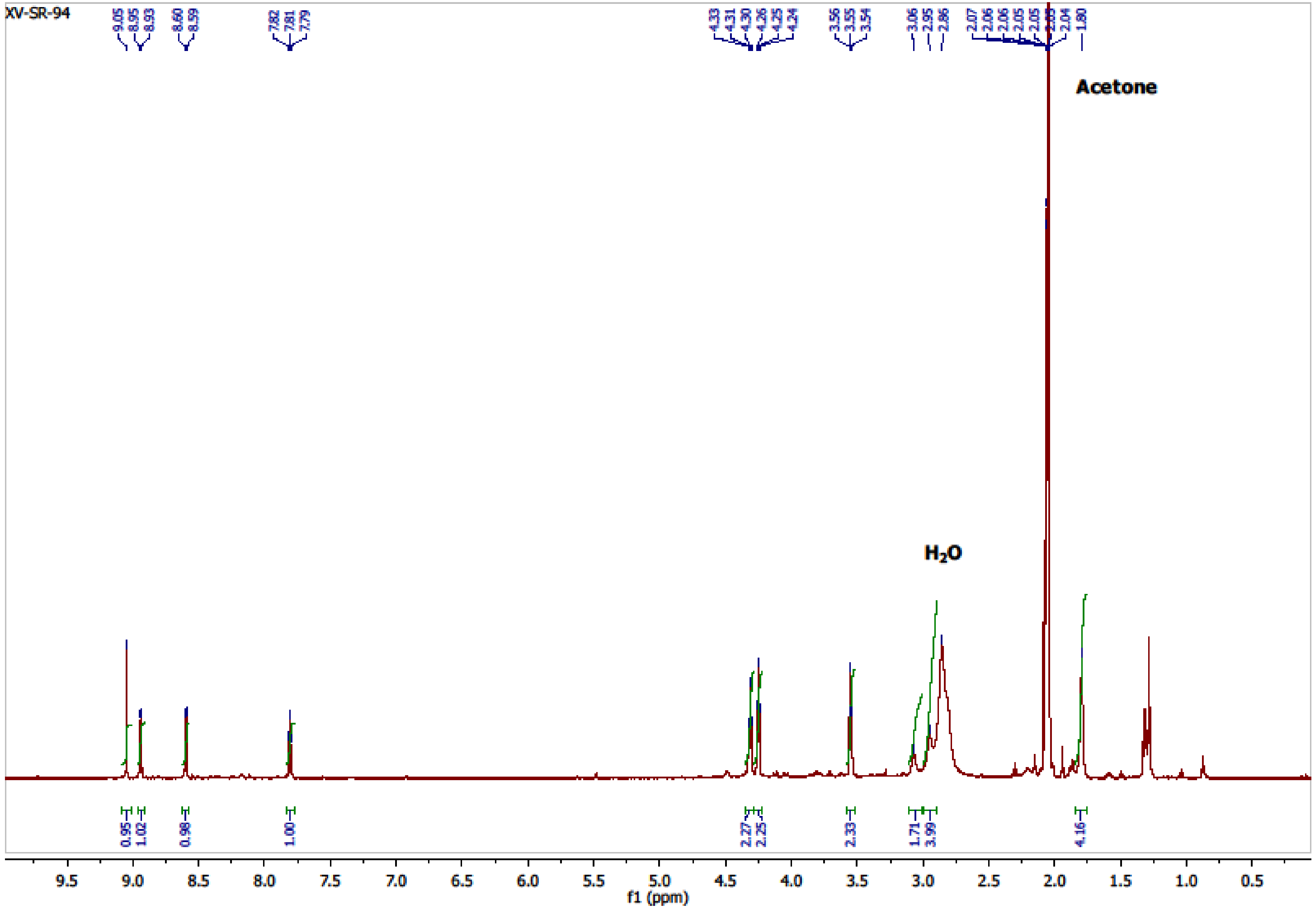

Figure S3. ^{1}H-NMR spectrum of **7**.

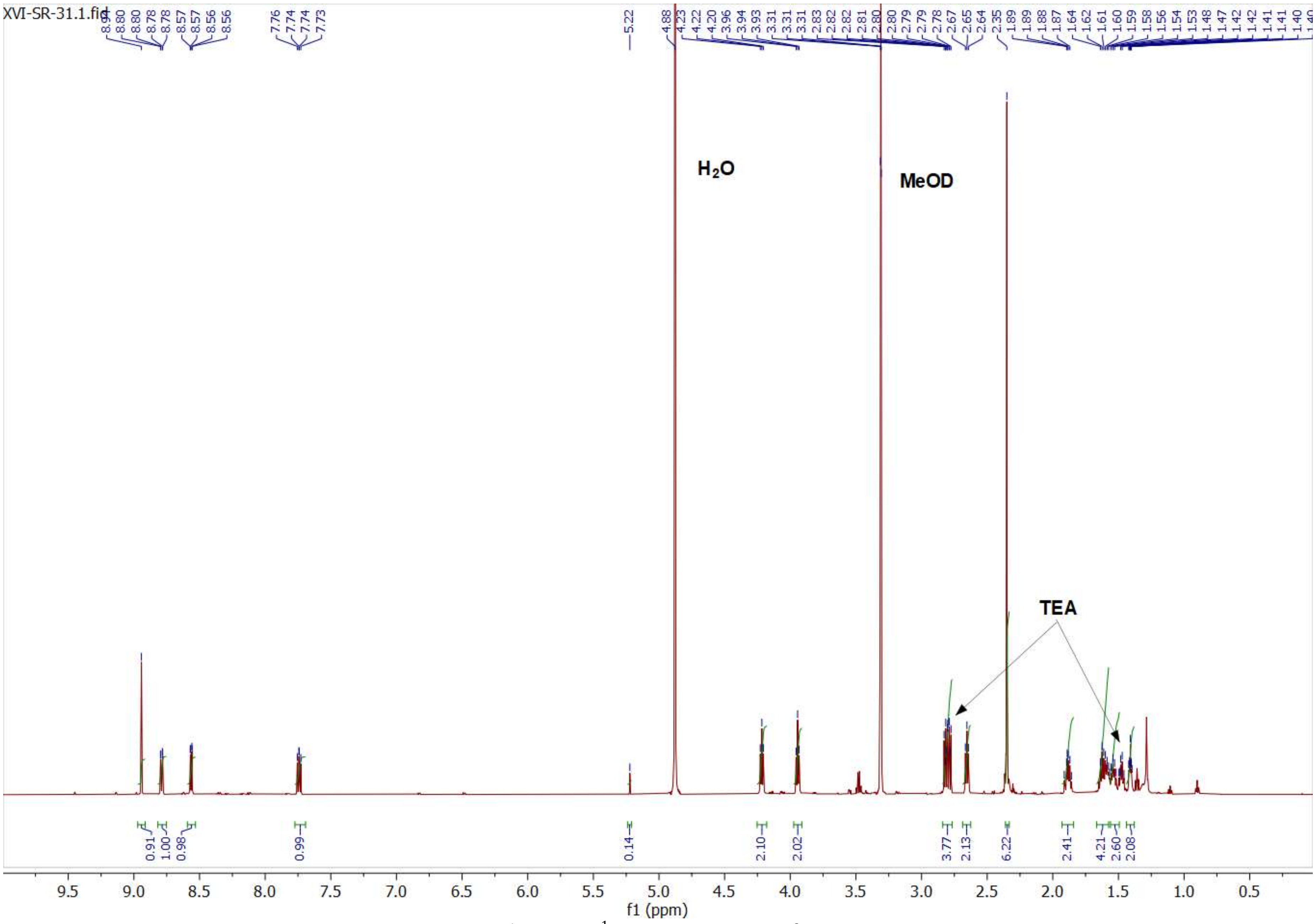

Figure S4. ^{1}H-NMR spectrum of **8**.

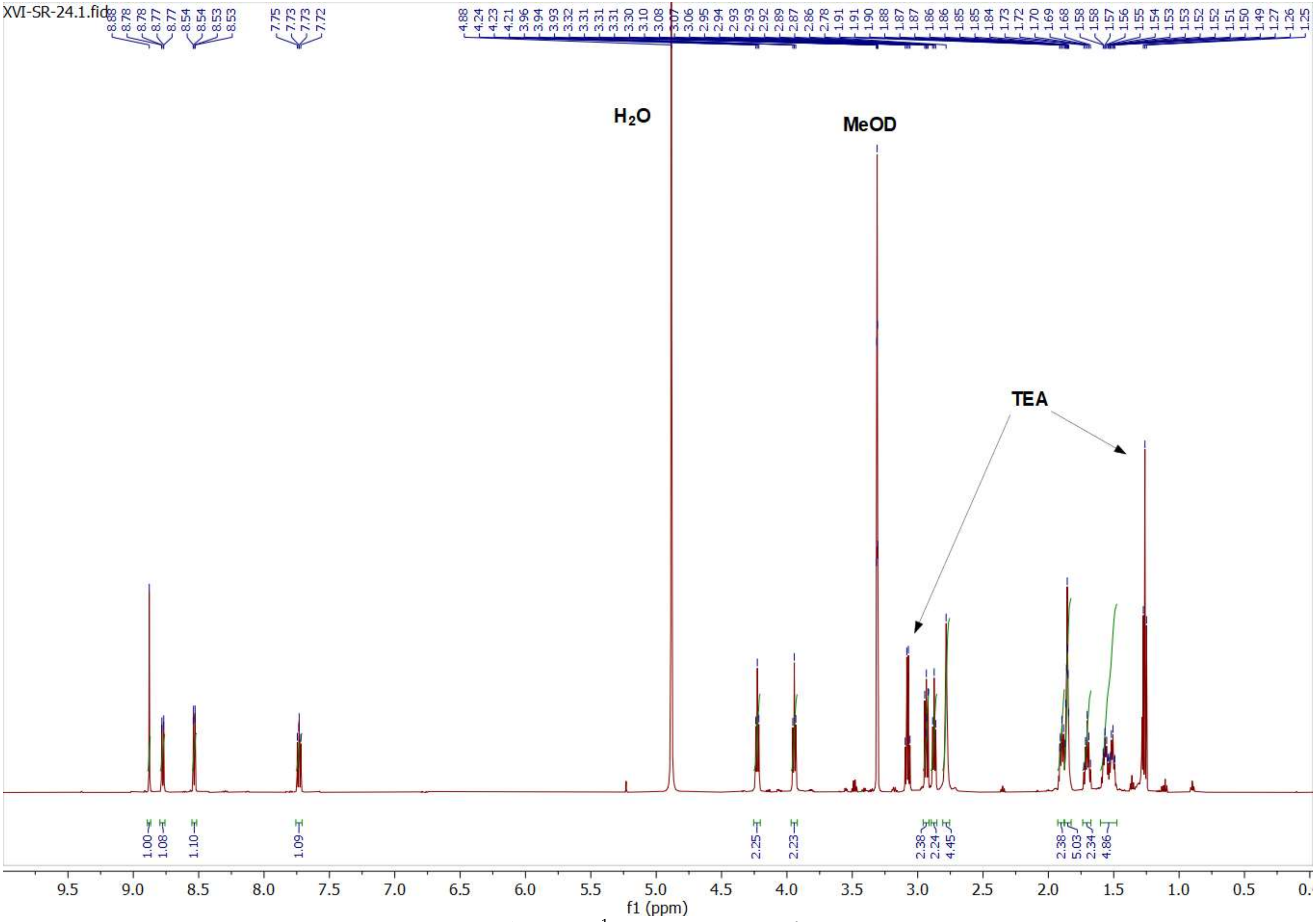

Figure S5. ^{1}H-NMR spectrum of **9**.

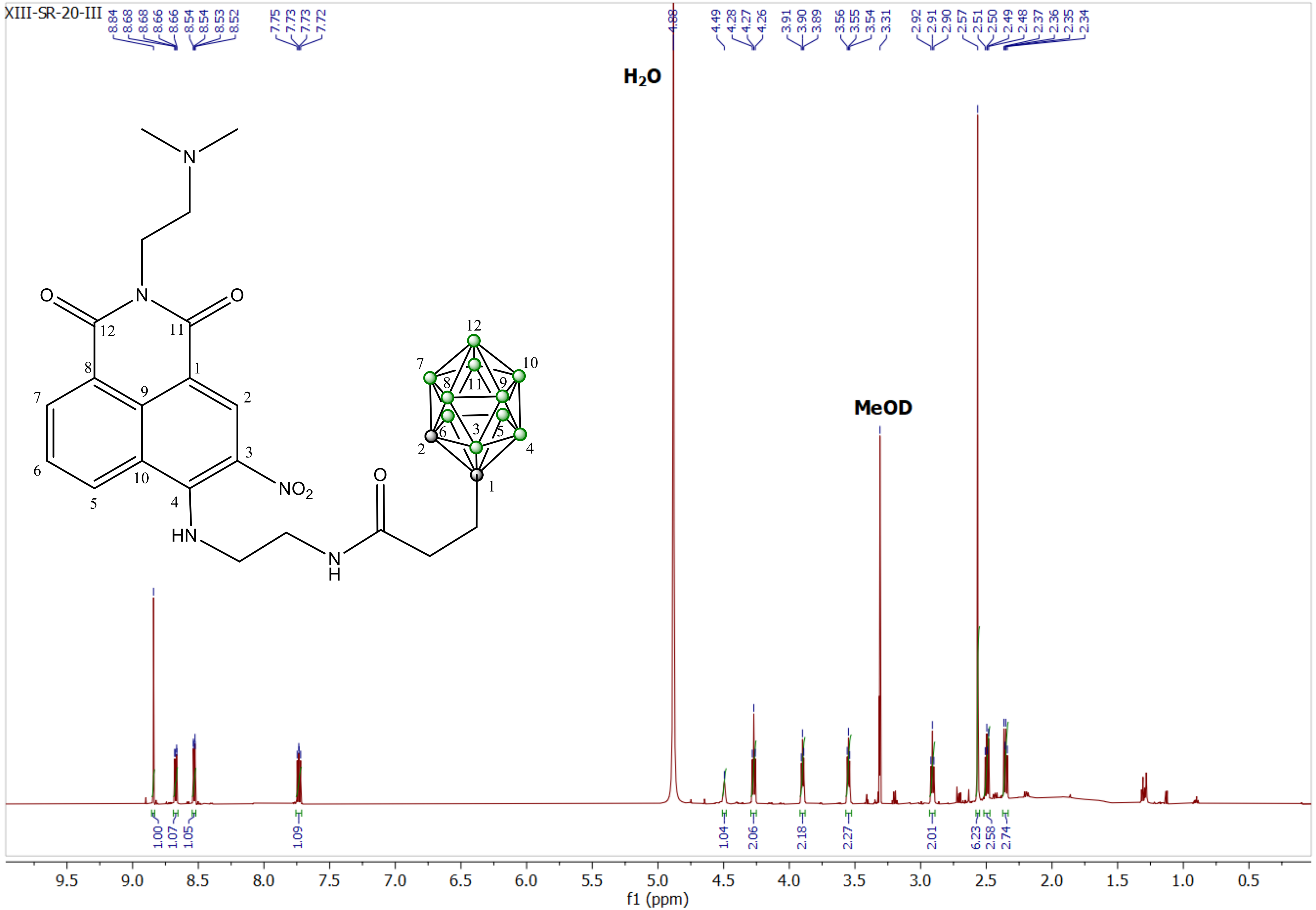

Figure S6. ^{1}H-NMR spectrum of **12**.

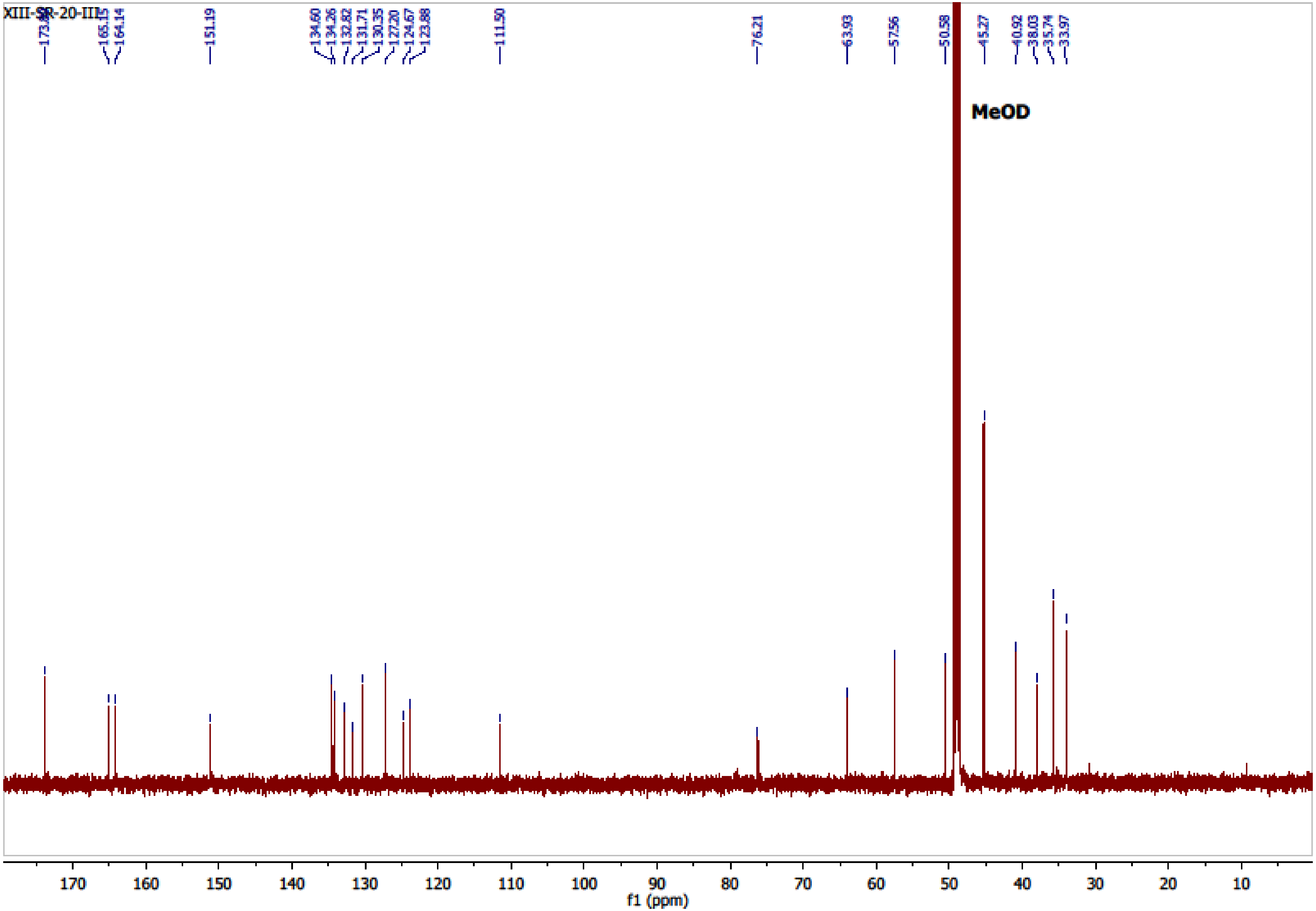

Figure S7. ^{13}C-NMR spectrum of **12**.

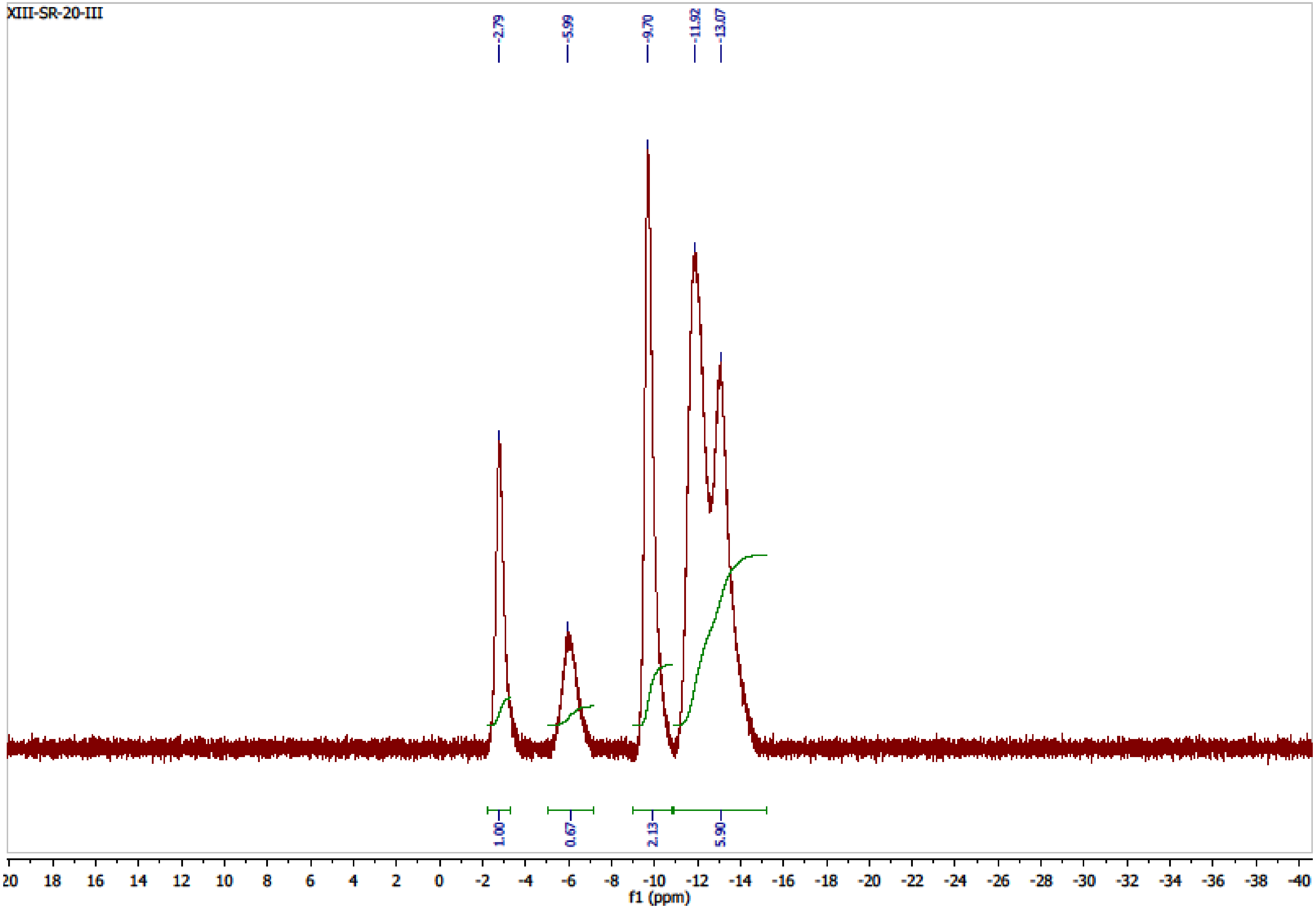

Figure S8. ^{11}B-NMR {^{1}H BB} spectrum of **12**.

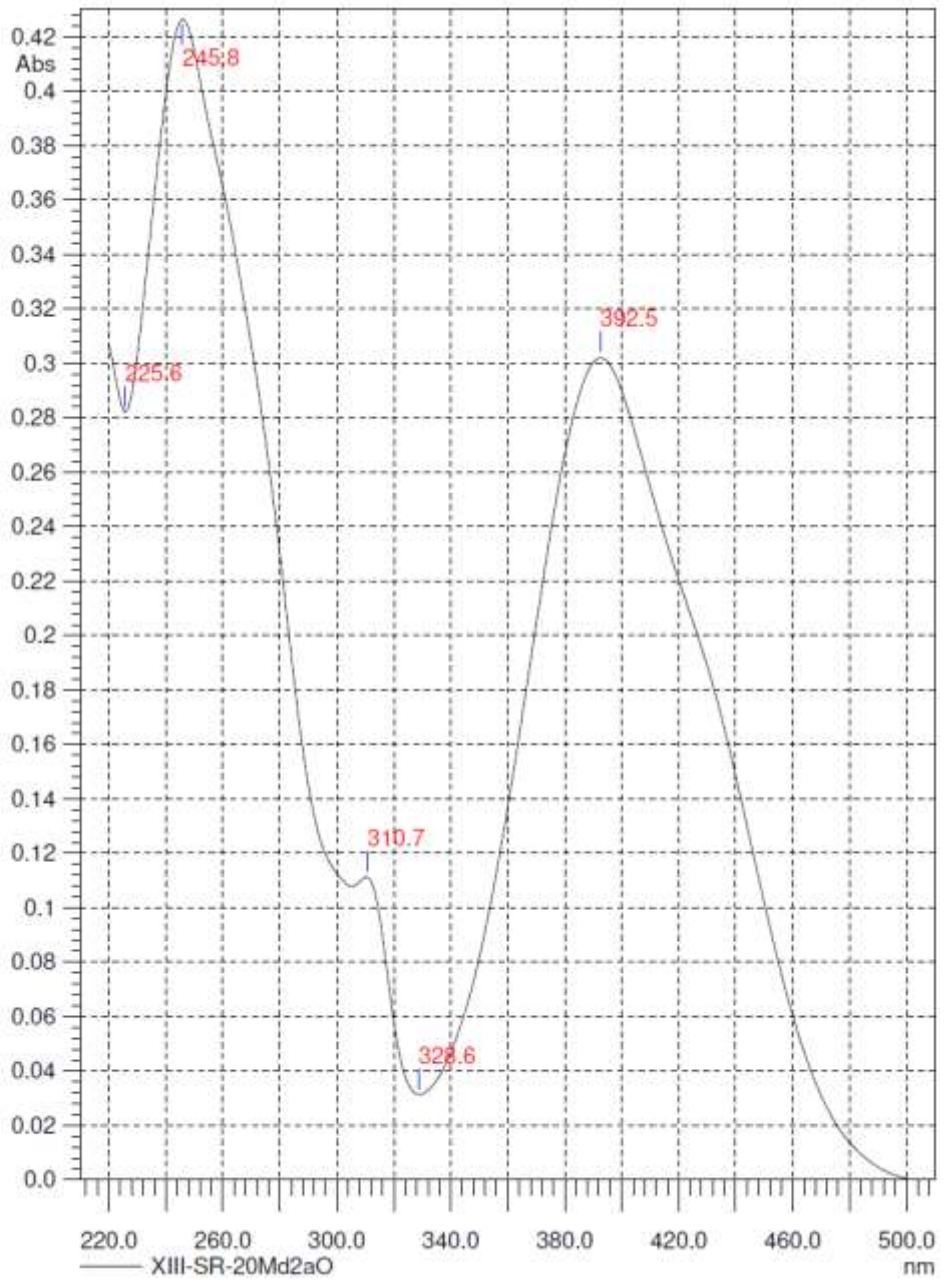

Figure S9. UV spectrum of **12**.

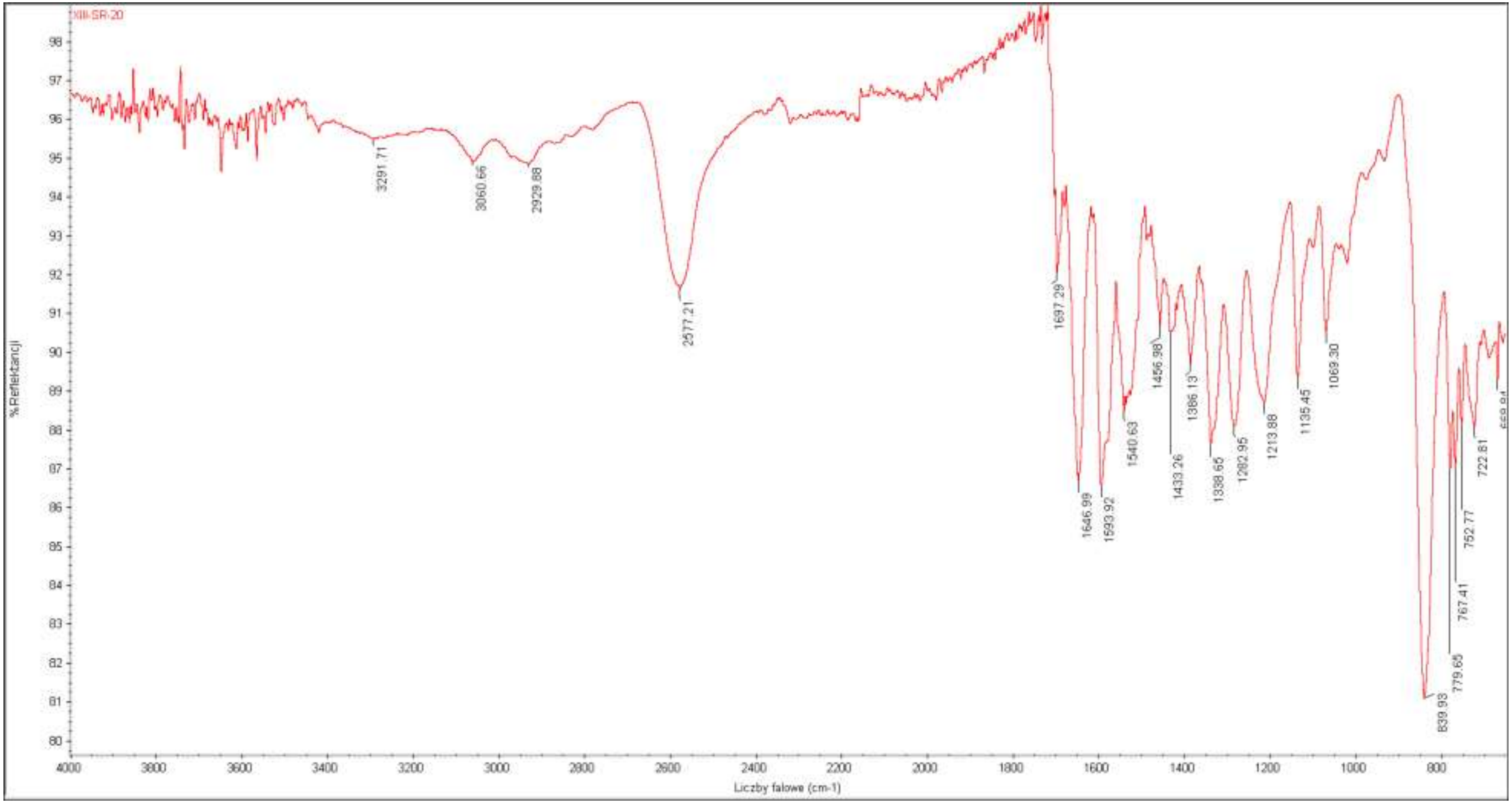

Figure S10. IR spectrum of **12**.

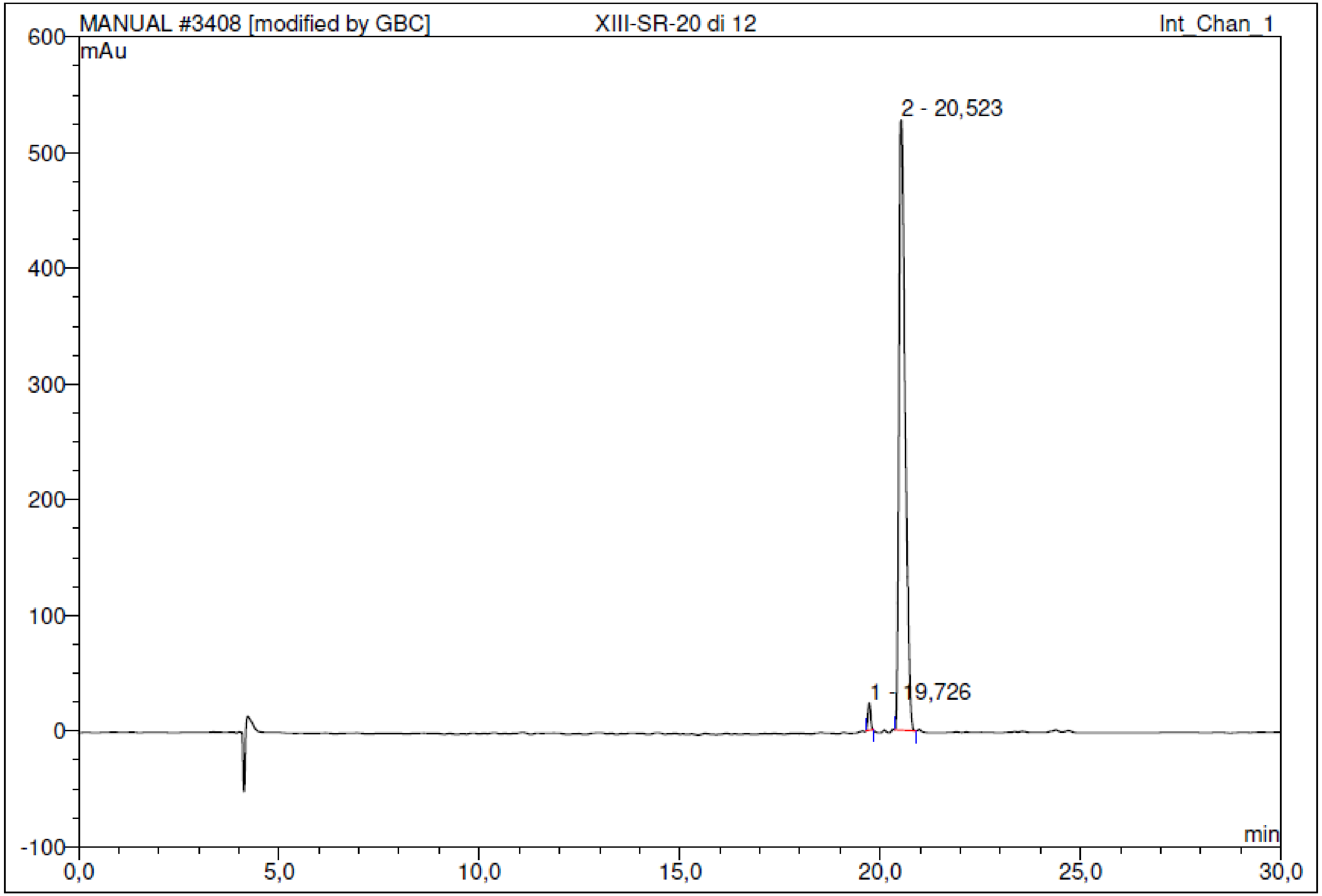

No.	Ret.Time min	Peak Name	Height mAu	Area mAu*min	Rel.Area %	Amount	Type
1	19,73	n.a.	23,266	1,890	1,97	n.a.	BMB*
2	20,52	n.a.	527,679	93,981	98,03	n.a.	BMB
Total:			550,945	95,871	100,00	0,000	

Figure S11. HPLC analysis of **12**.

Spectrum Name: XIII-SR-20_pt
Start Ion: 50
End Ion: 700
Source: APCI + 10.0µA 400C
Capillary: 150V 300C Offset: 25V Span: 0V

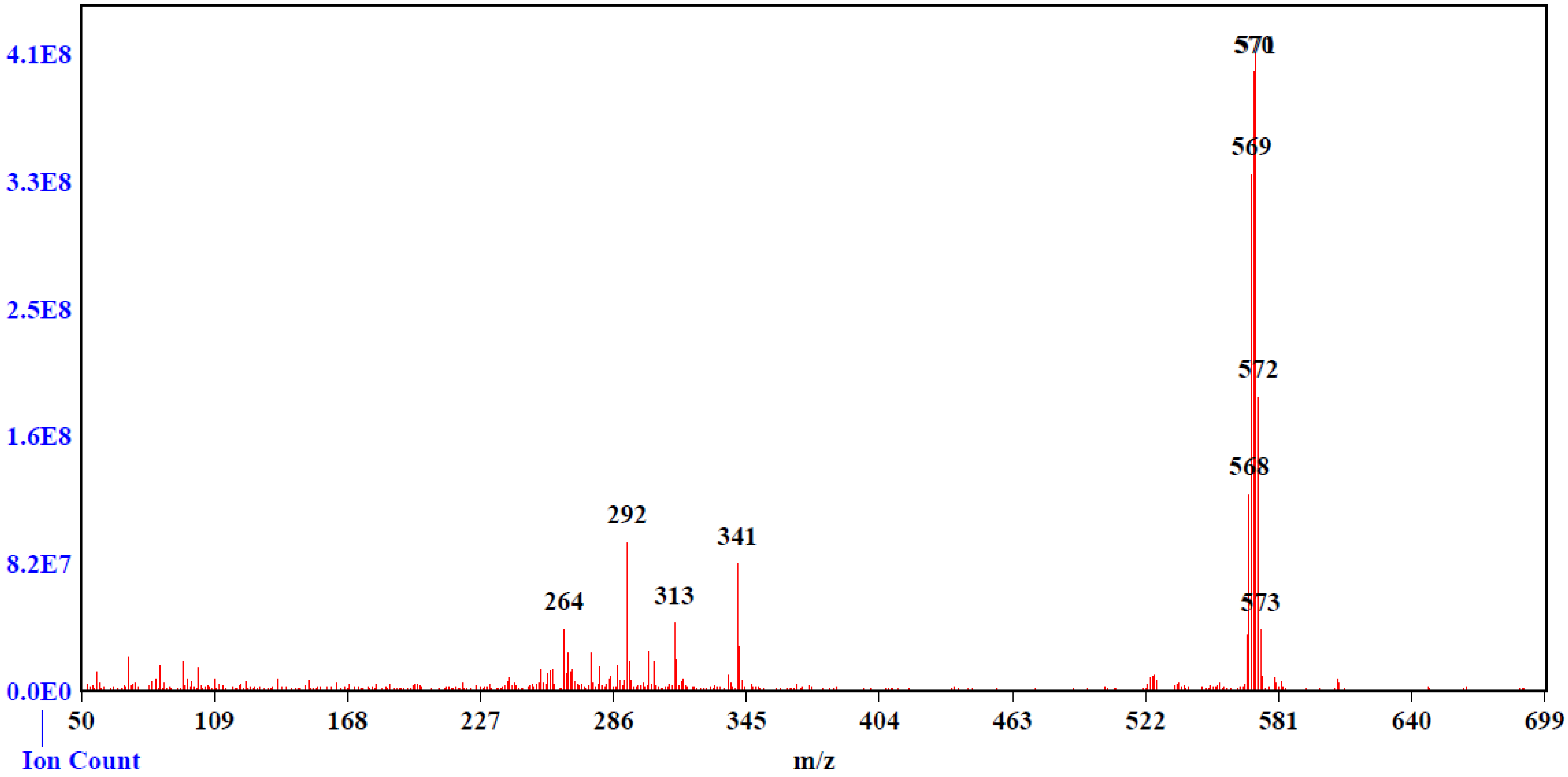

Figure S12. MS spectrum of **12**.

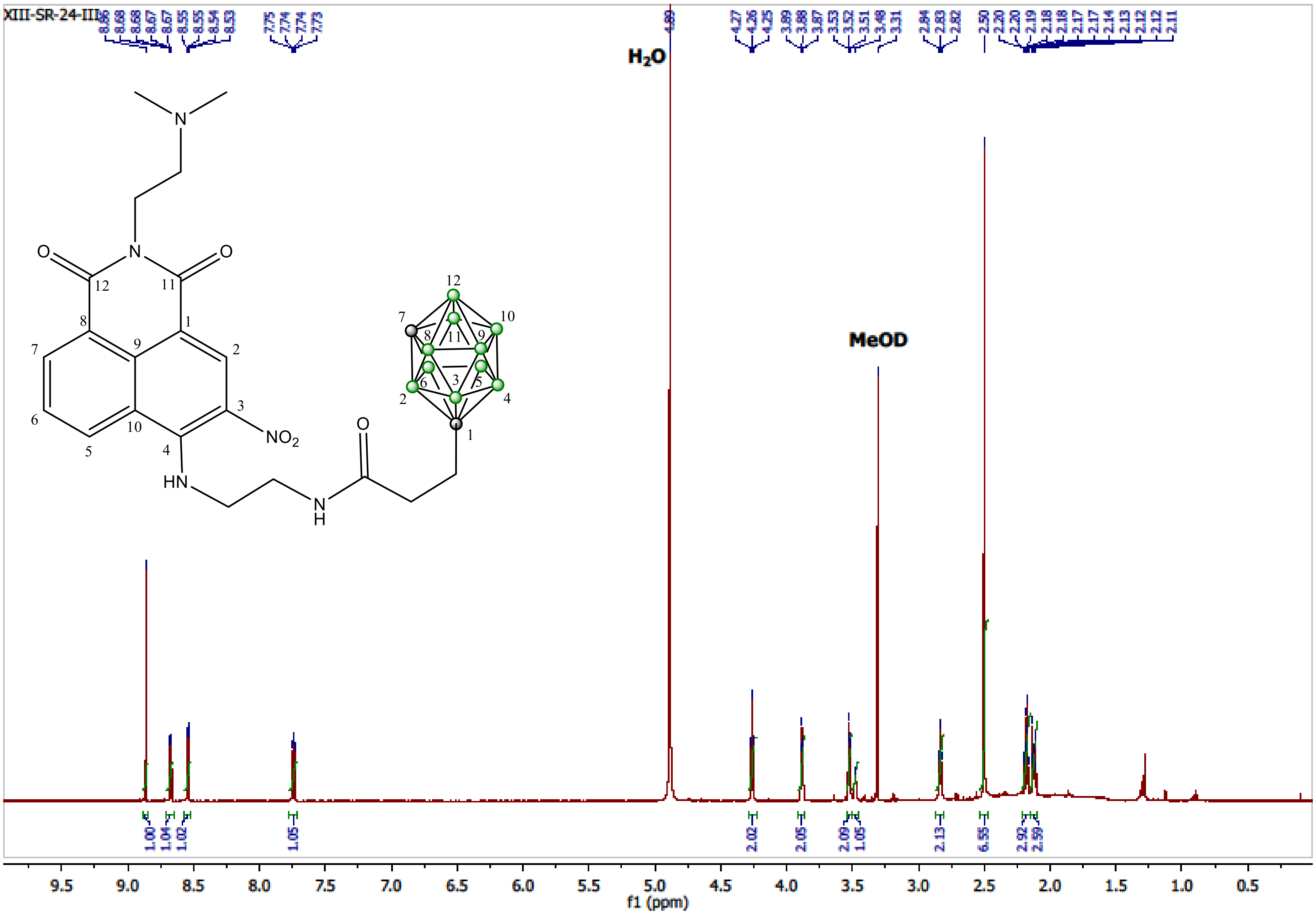

Figure S13. ^{1}H-NMR spectrum of **13**.

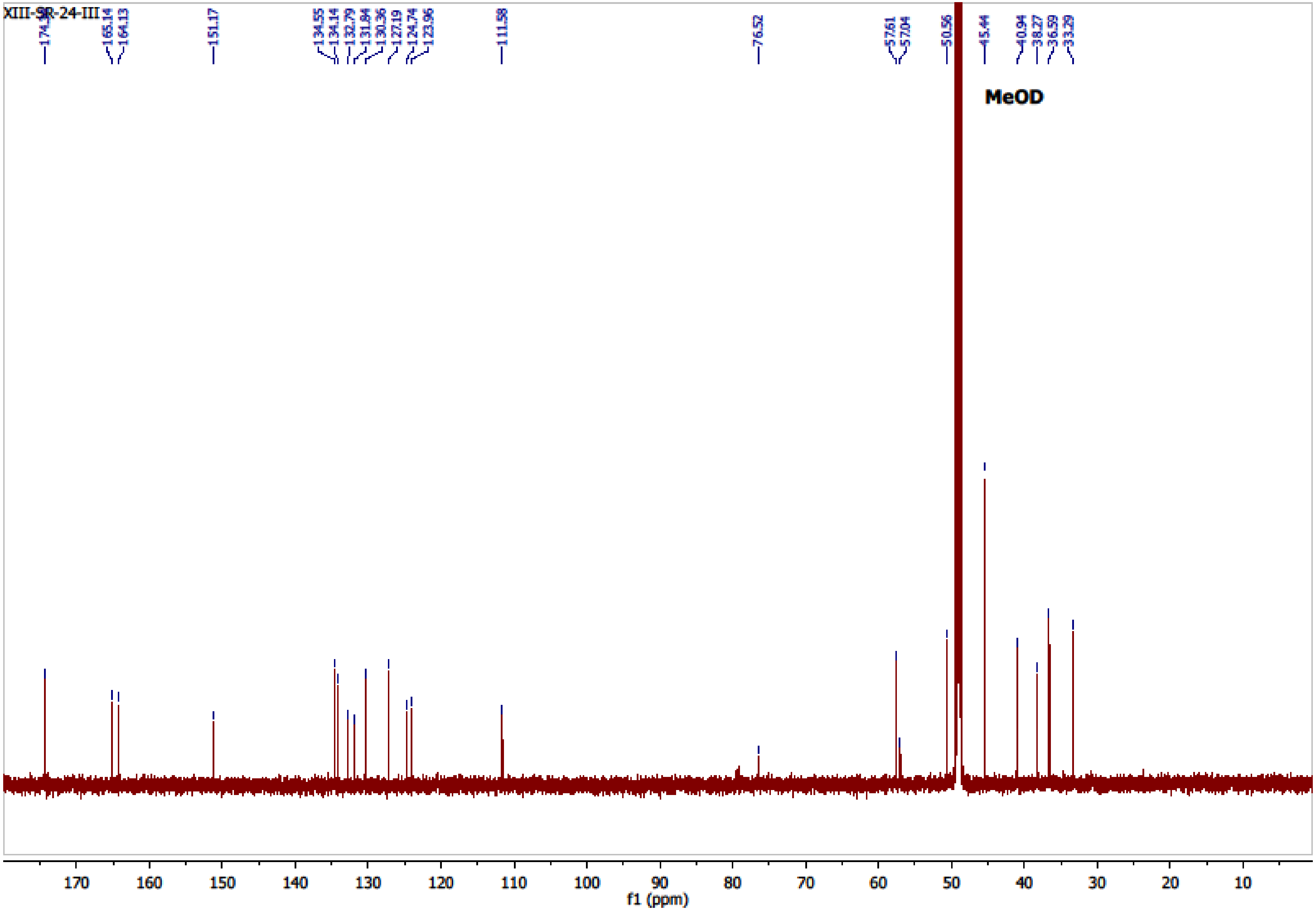

Figure S14. ^{13}C-NMR spectrum of **13**.

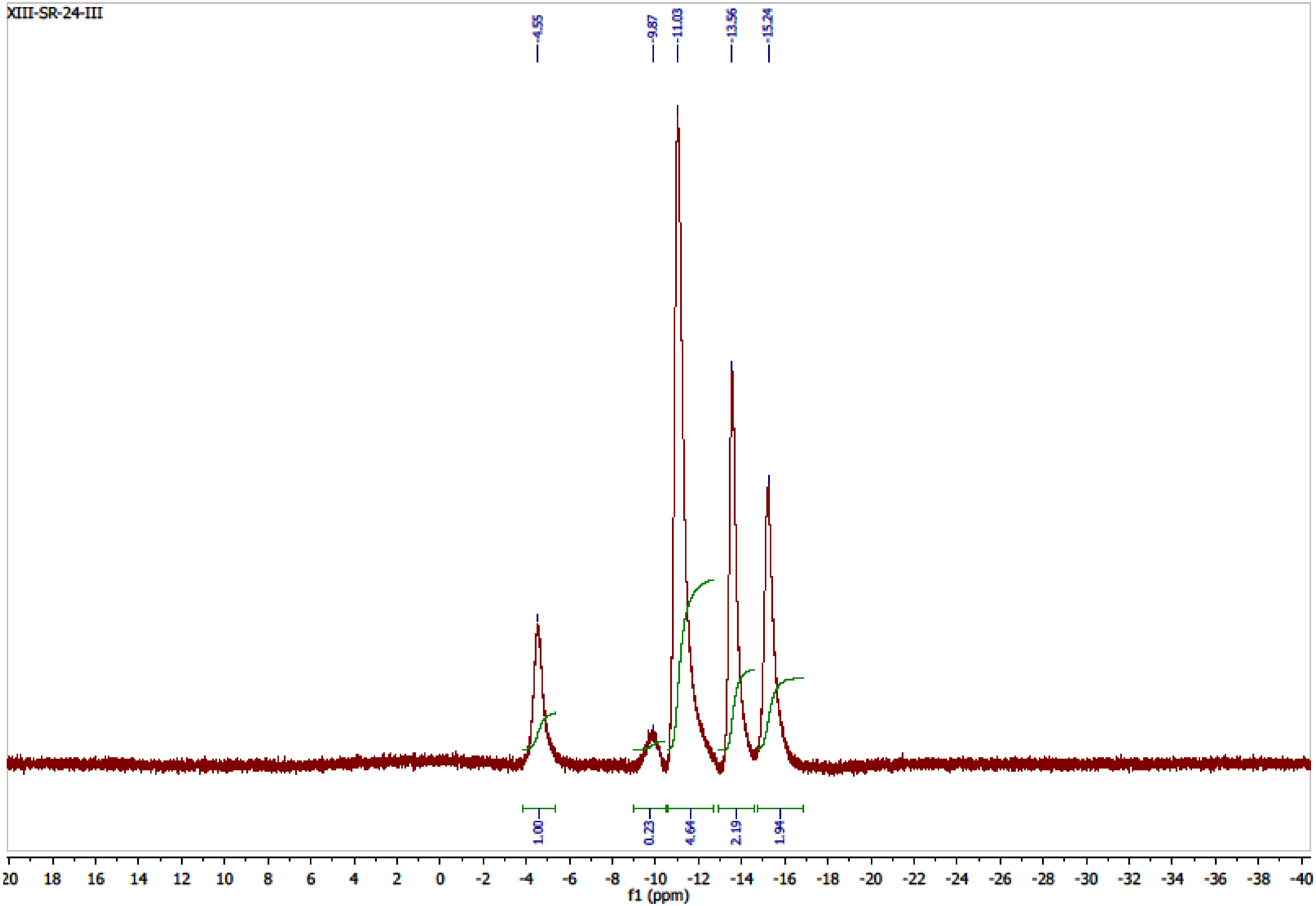

Figure S15. ^{11}B-NMR {^{1}H BB} spectrum of **13**.

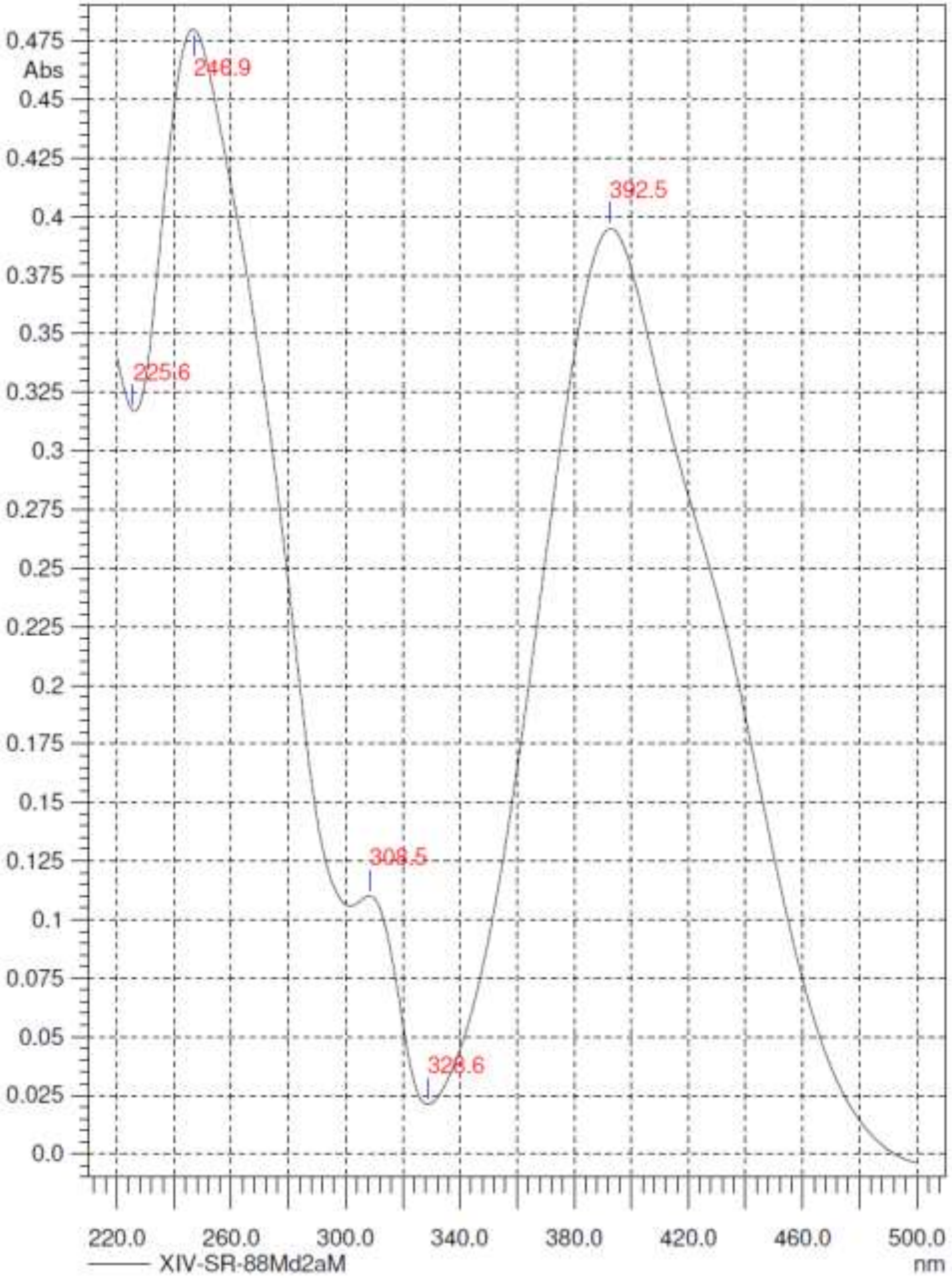

Figure S16. UV spectrum of **13**.

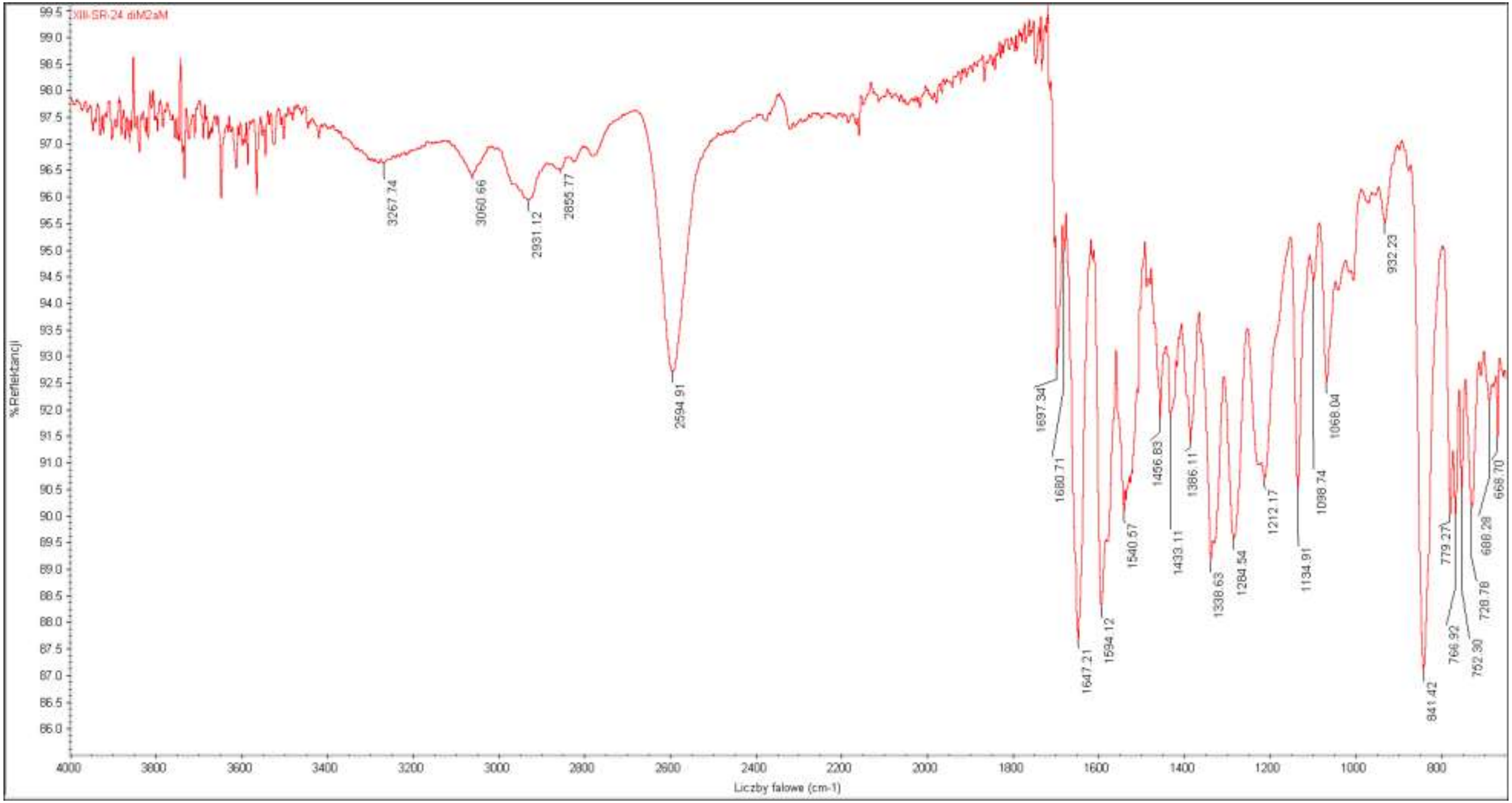

Figure S17. IR spectrum of **13**.

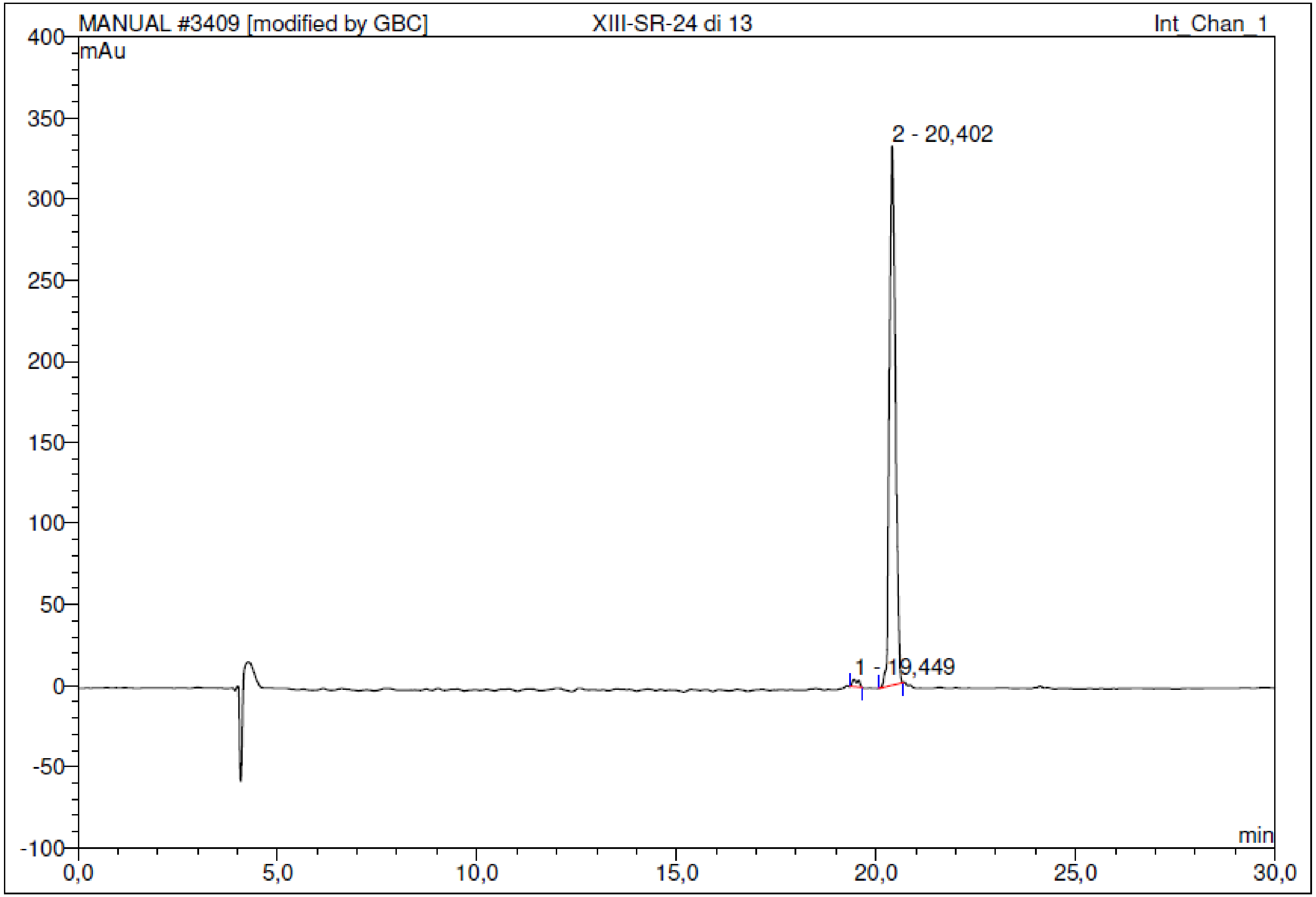

No.	Ret.Time min	Peak Name	Height mAu	Area mAu*min	Rel.Area %	Amount	Type
1	19,45	n.a.	4,394	0,744	1,26	n.a.	BMB*
2	20,40	n.a.	332,632	58,210	98,74	n.a.	BMB
Total:			337,026	58,954	100,00	0,000	

Figure S18. HPLC analysis of **13**.

Spectrum Name: XIII-SR-24_pt
Start Ion: 50
End Ion: 800
Source: APCI + 10.0µA 400C
Capillary: 150V 300C Offset: 25V Span: 0V

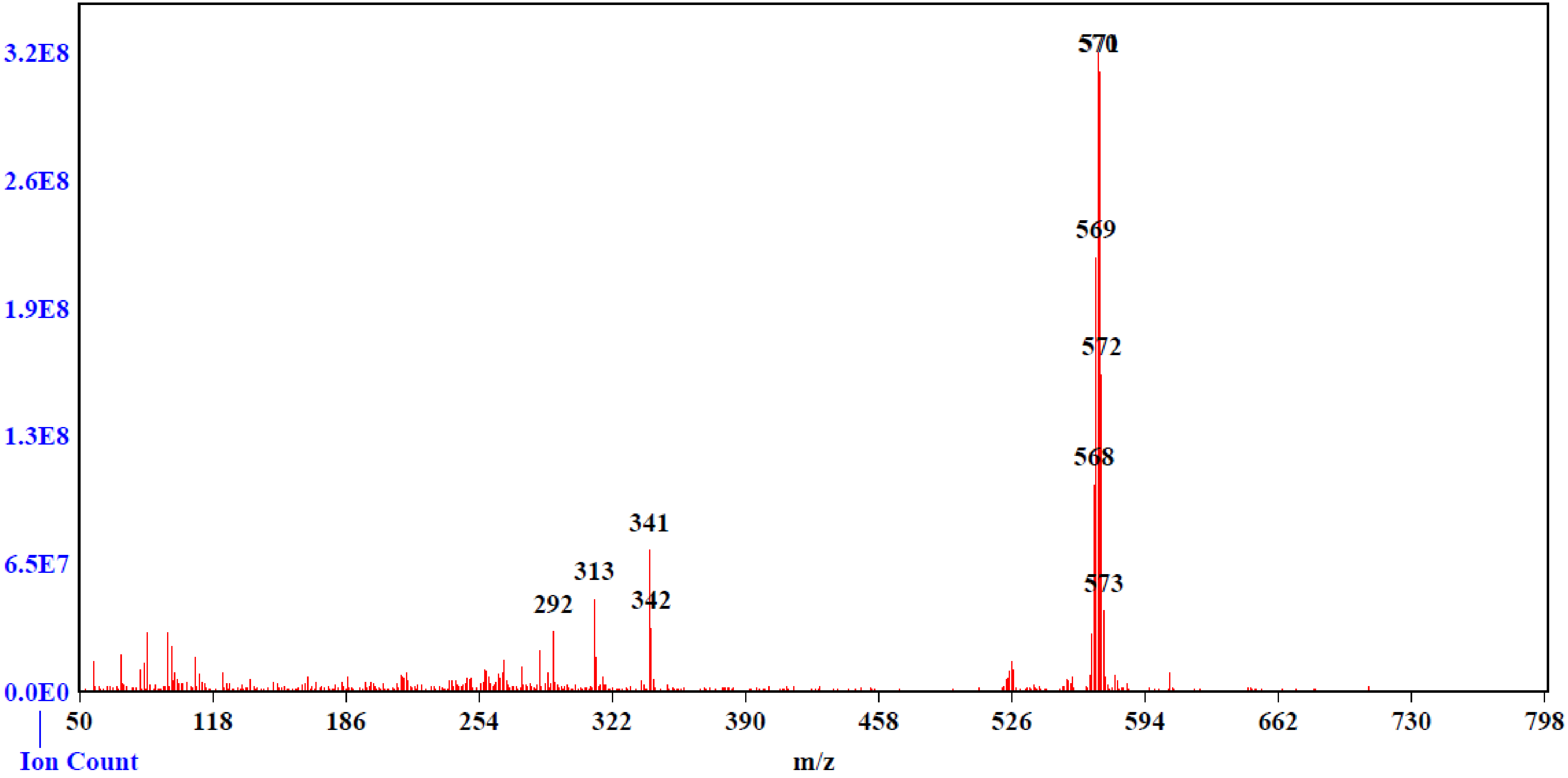

Figure S19. MS spectrum of **13**.

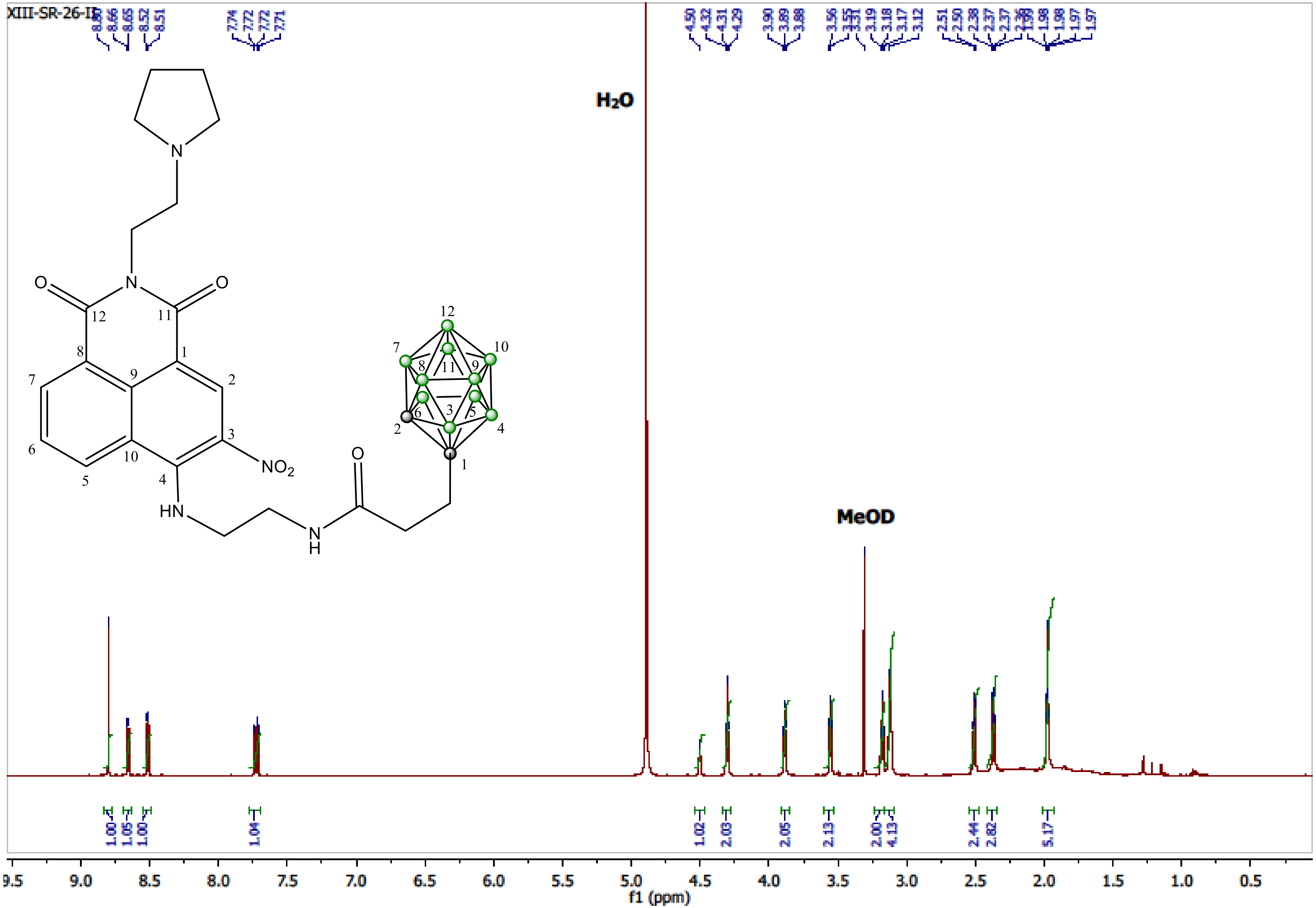

Figure S20. ^{1}H-NMR spectrum of **14**.

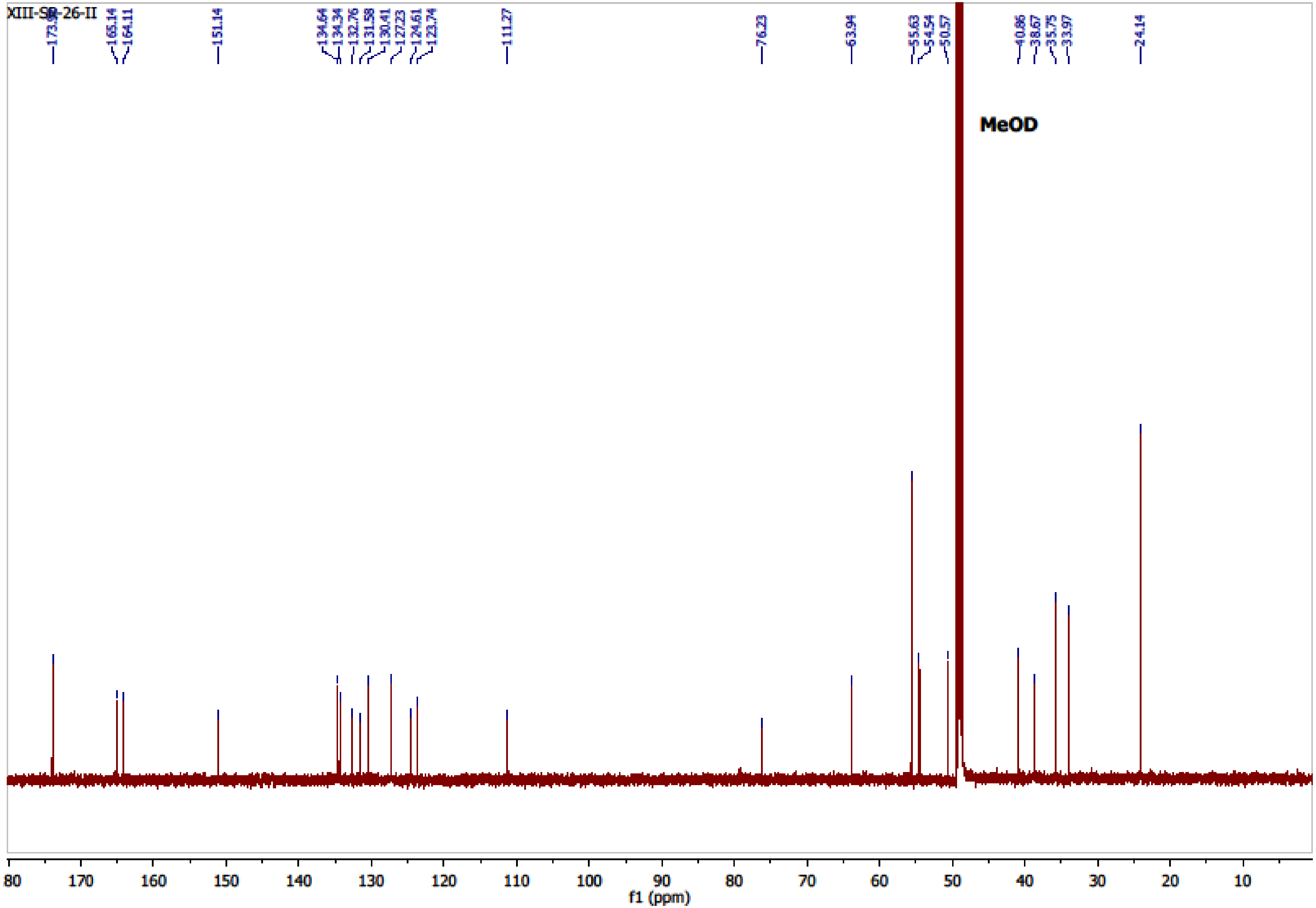

Figure S21. ^{13}C-NMR spectrum of **14**.

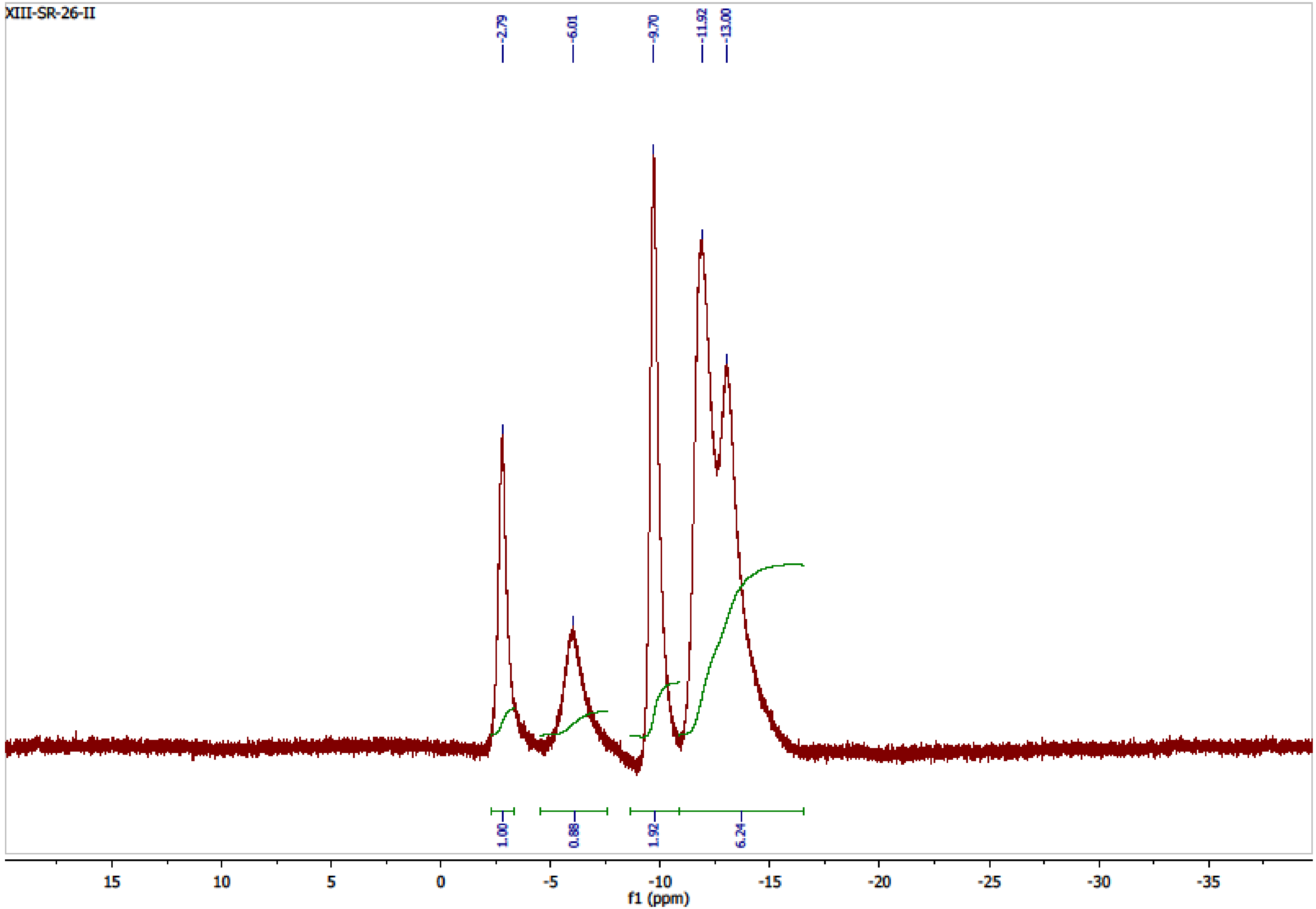

Figure S22. ^{11}B-NMR {^{1}H BB} spectrum of **14**.

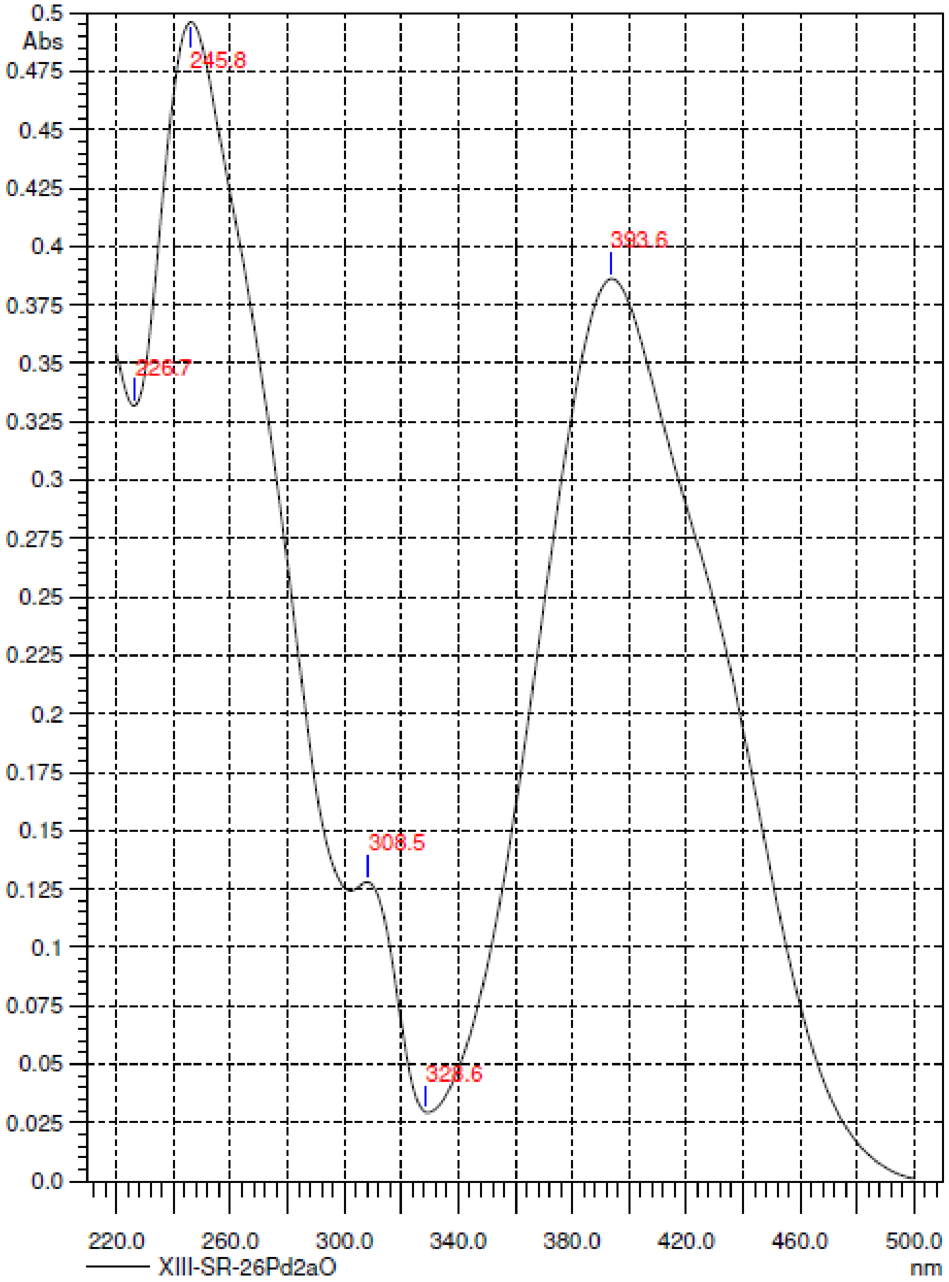

Figure S23. UV spectrum of **14**.

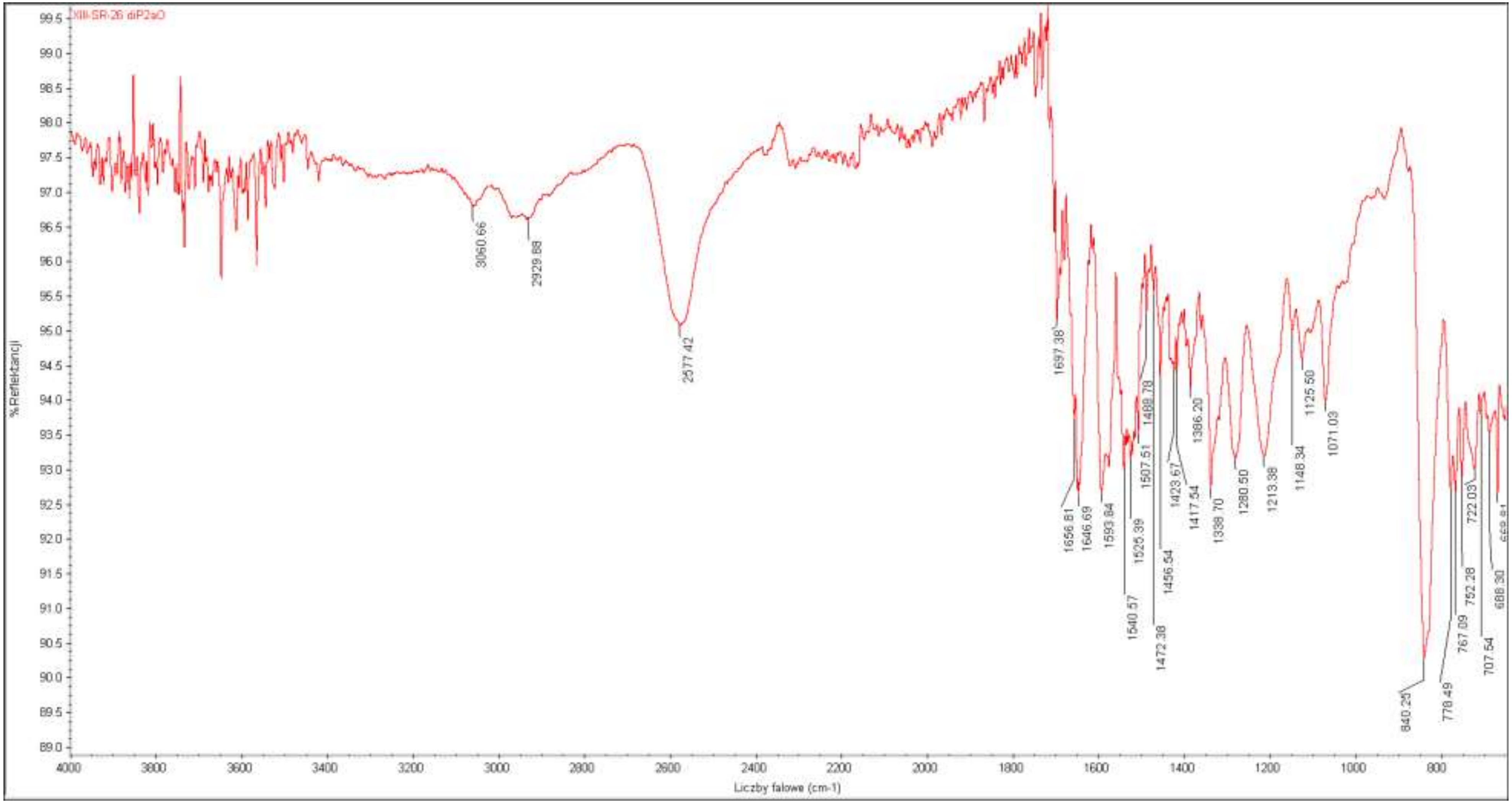

Figure S24. IR spectrum of **14**.

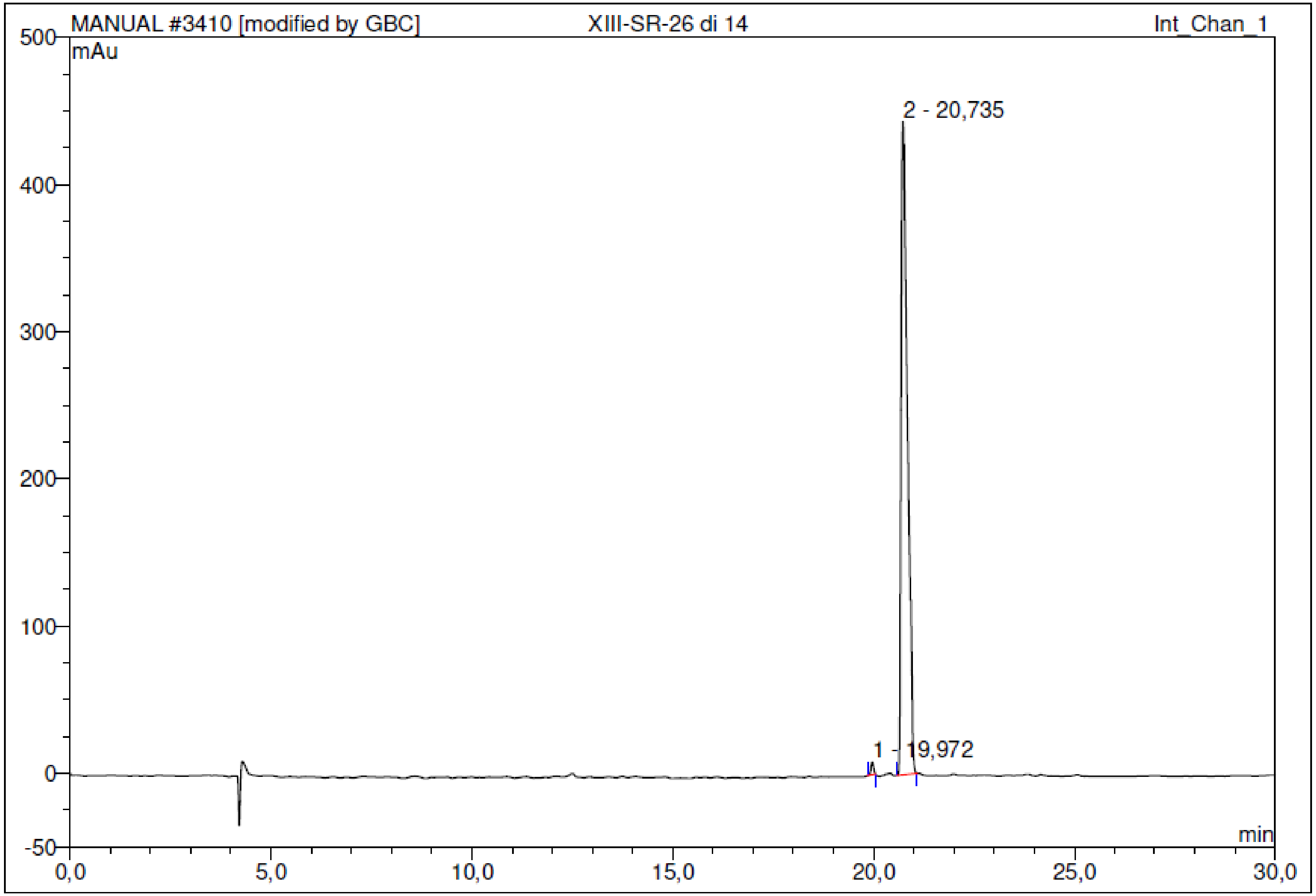

No.	Ret.Time min	Peak Name	Height mAu	Area mAu*min	Rel.Area %	Amount	Type
1	19,97	n.a.	8,397	0,643	0,80	n.a.	BMB*
2	20,74	n.a.	443,860	80,227	99,20	n.a.	BMB
**Total:**			452,257	80,870	100,00	0,000	

Figure S25. HPLC analysis of **14**.

Spectrum Name: XIII-SR-26_pt
Start Ion: 50
End Ion: 800
Source: APCI + 10.0µA 400C
Capillary: 150V 300C Offset: 25V Span: 0V

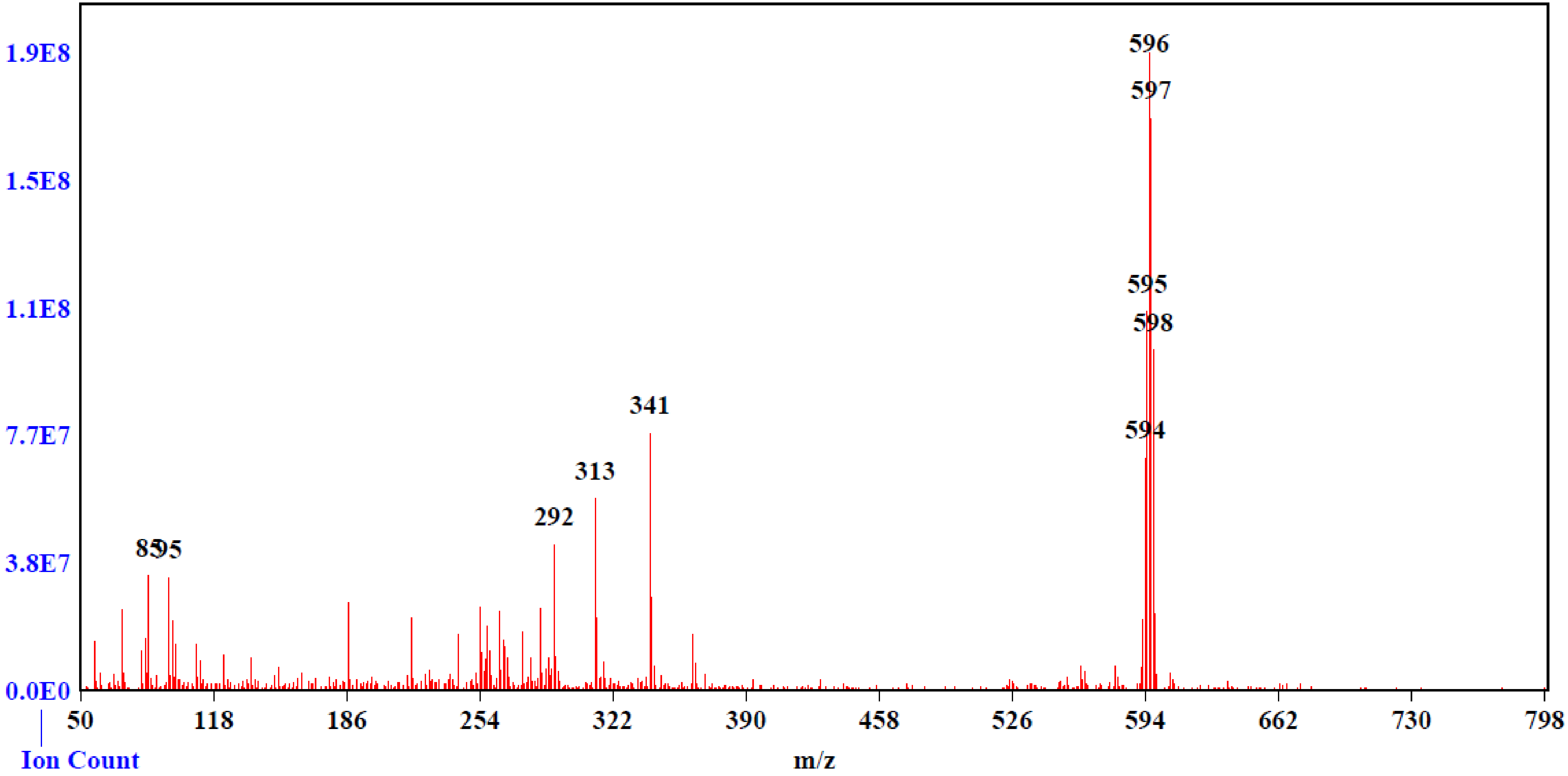

Figure S26. MS spectrum of **14**.

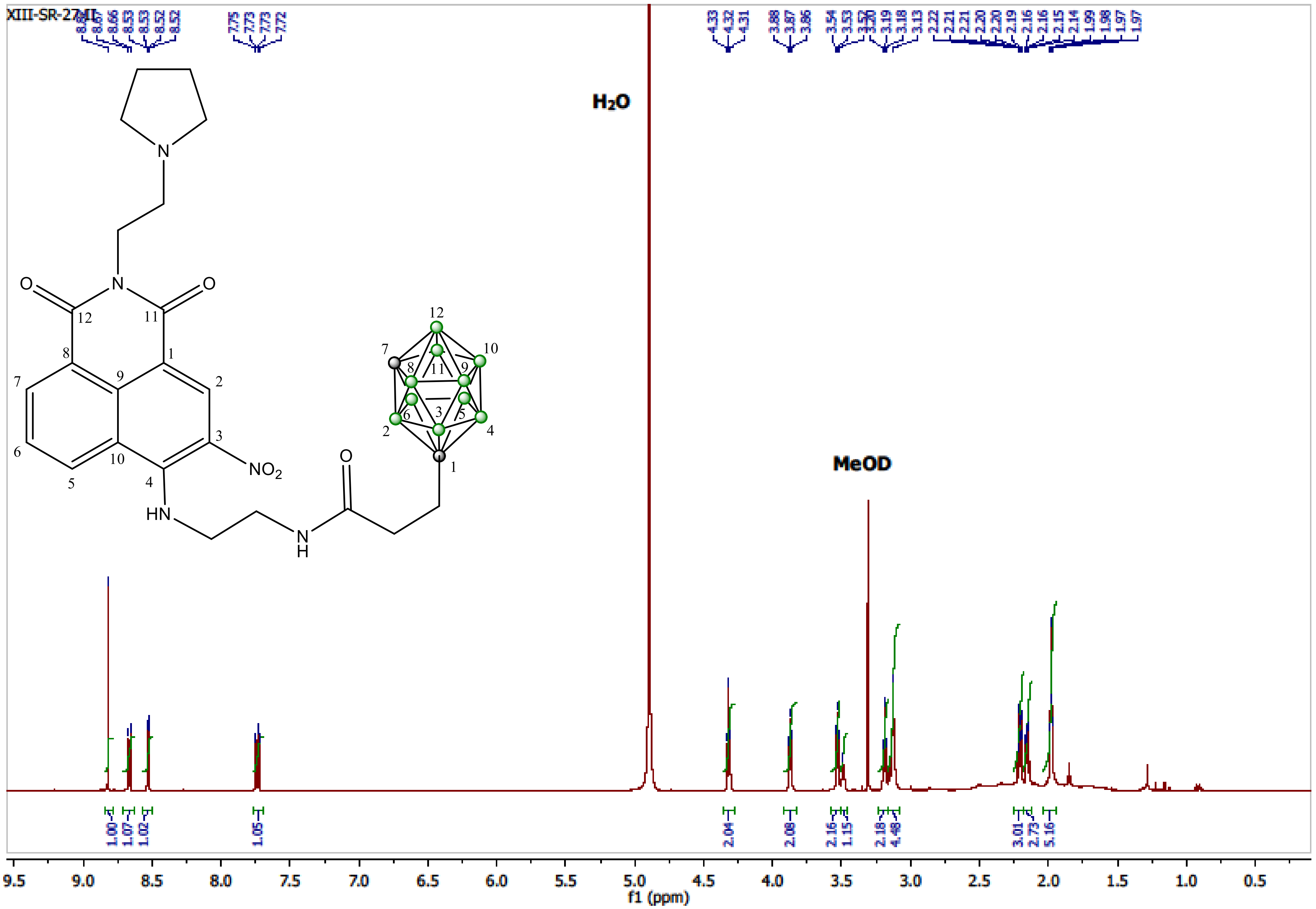

Figure S27. ^{1}H-NMR spectrum of **15**.

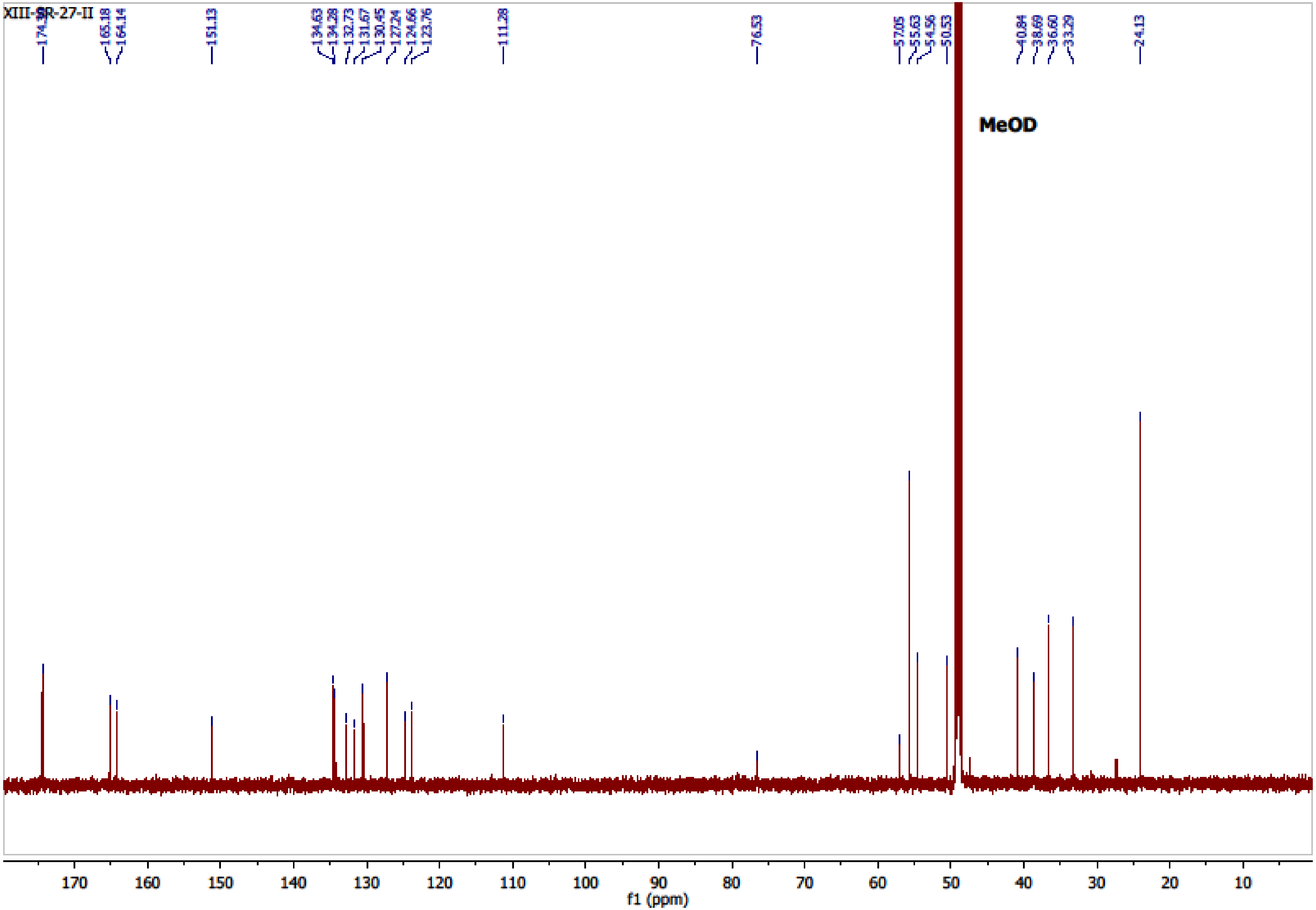

Figure S28. ^{13}C-NMR spectrum of **15**.

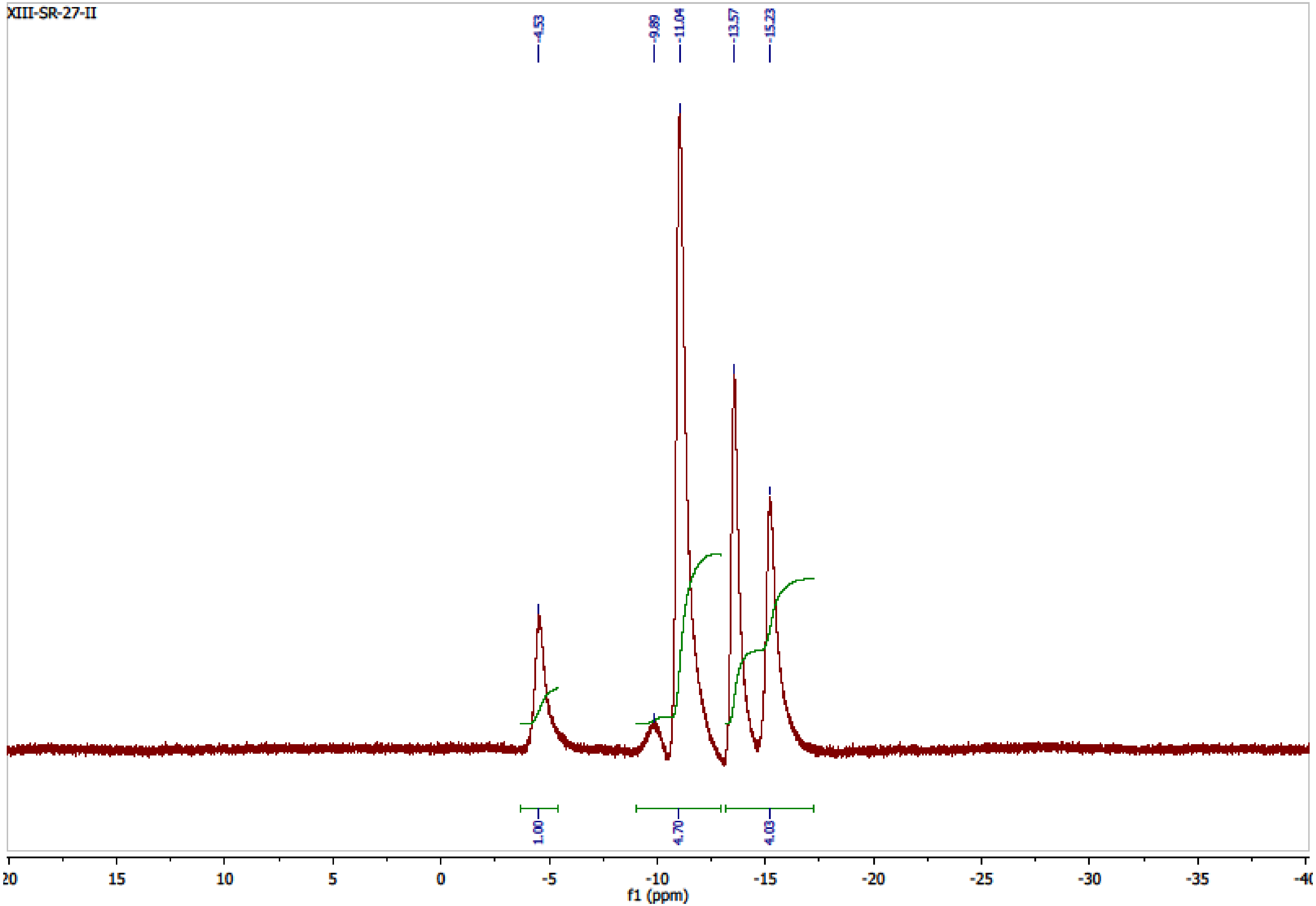

Figure S29. ^{11}B-NMR {^{1}H BB} spectrum of **15**.

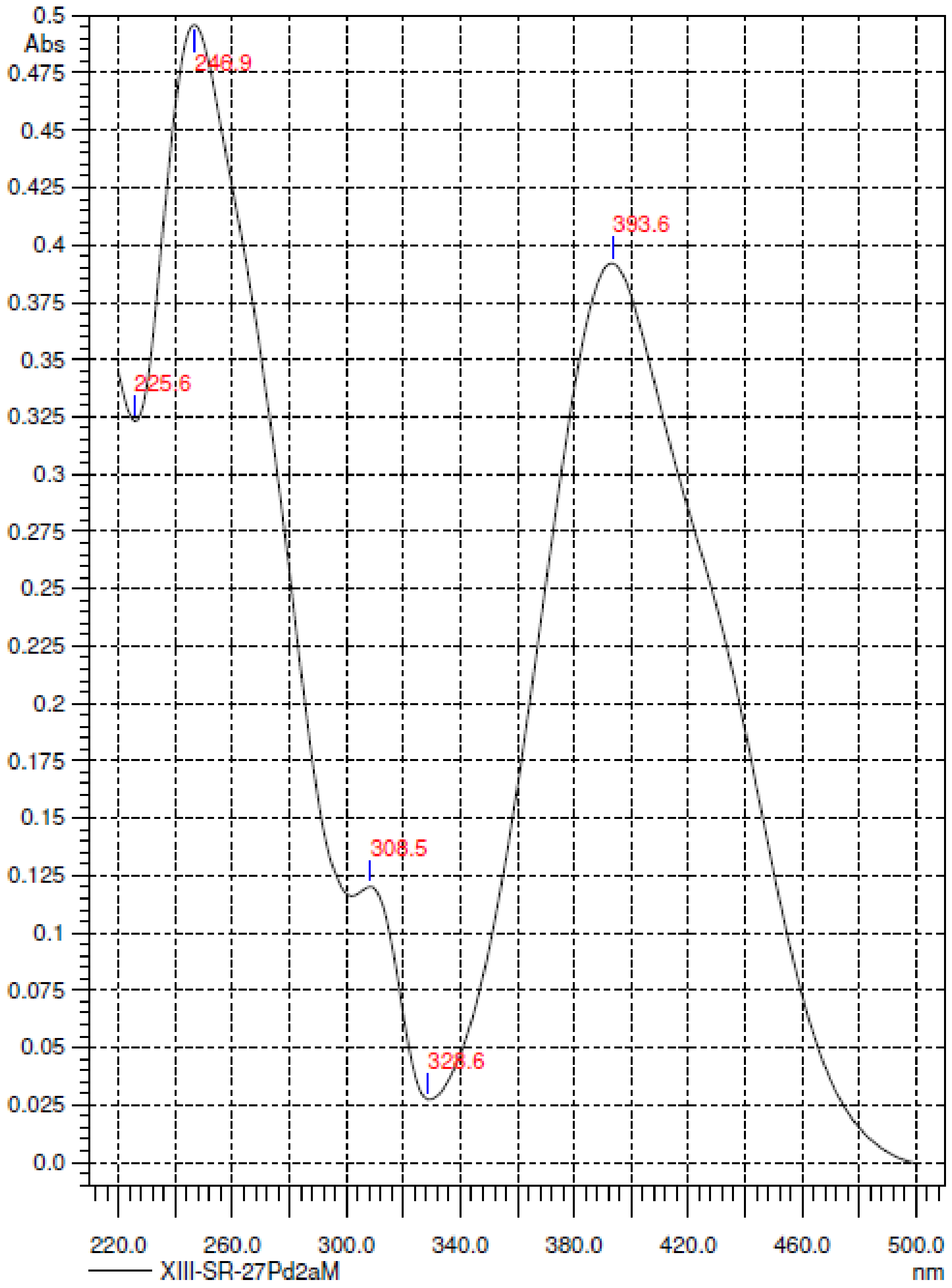

Figure S30. UV spectrum of **15**.

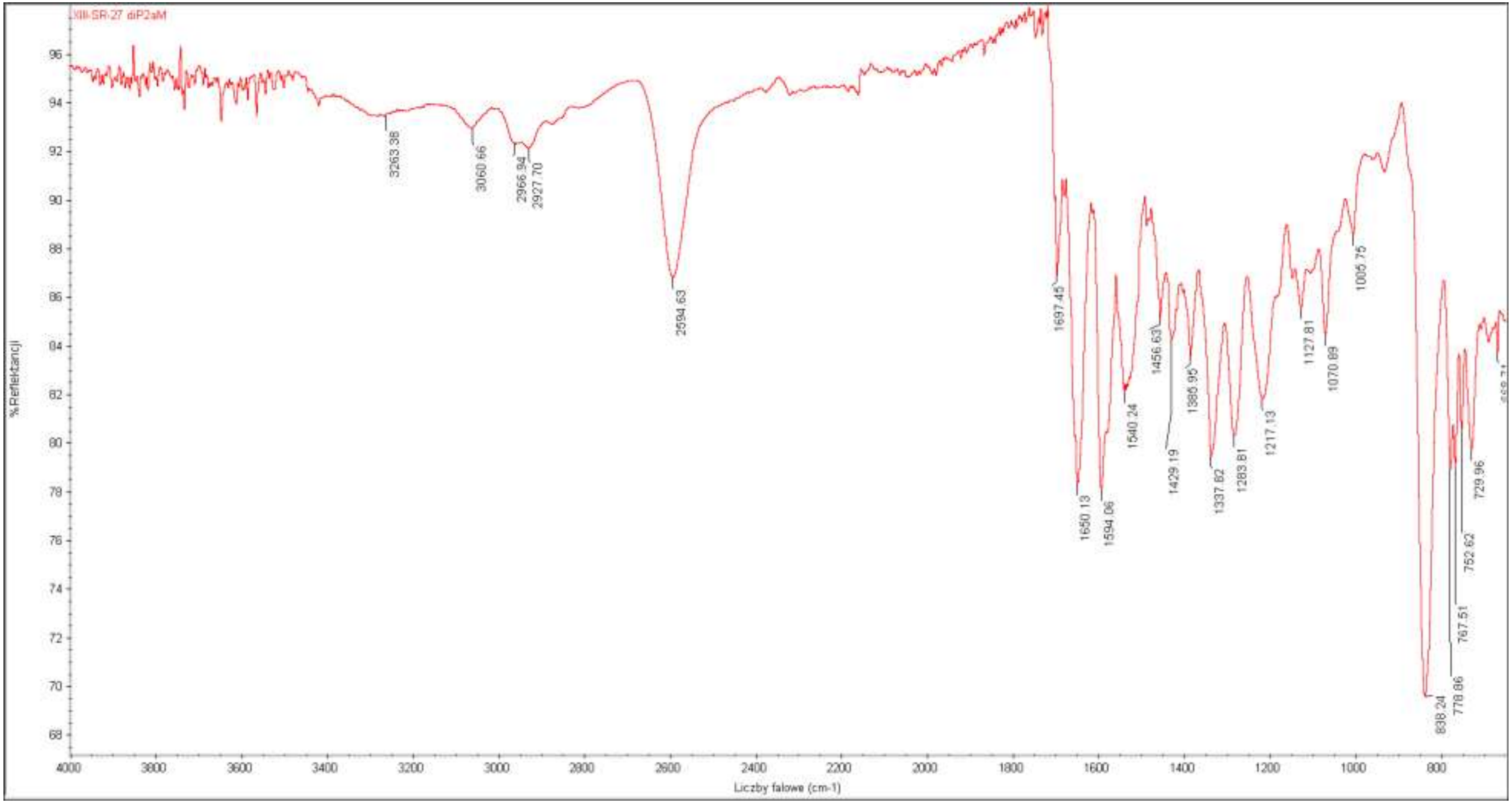

Figure S31. IR spectrum of **15**.

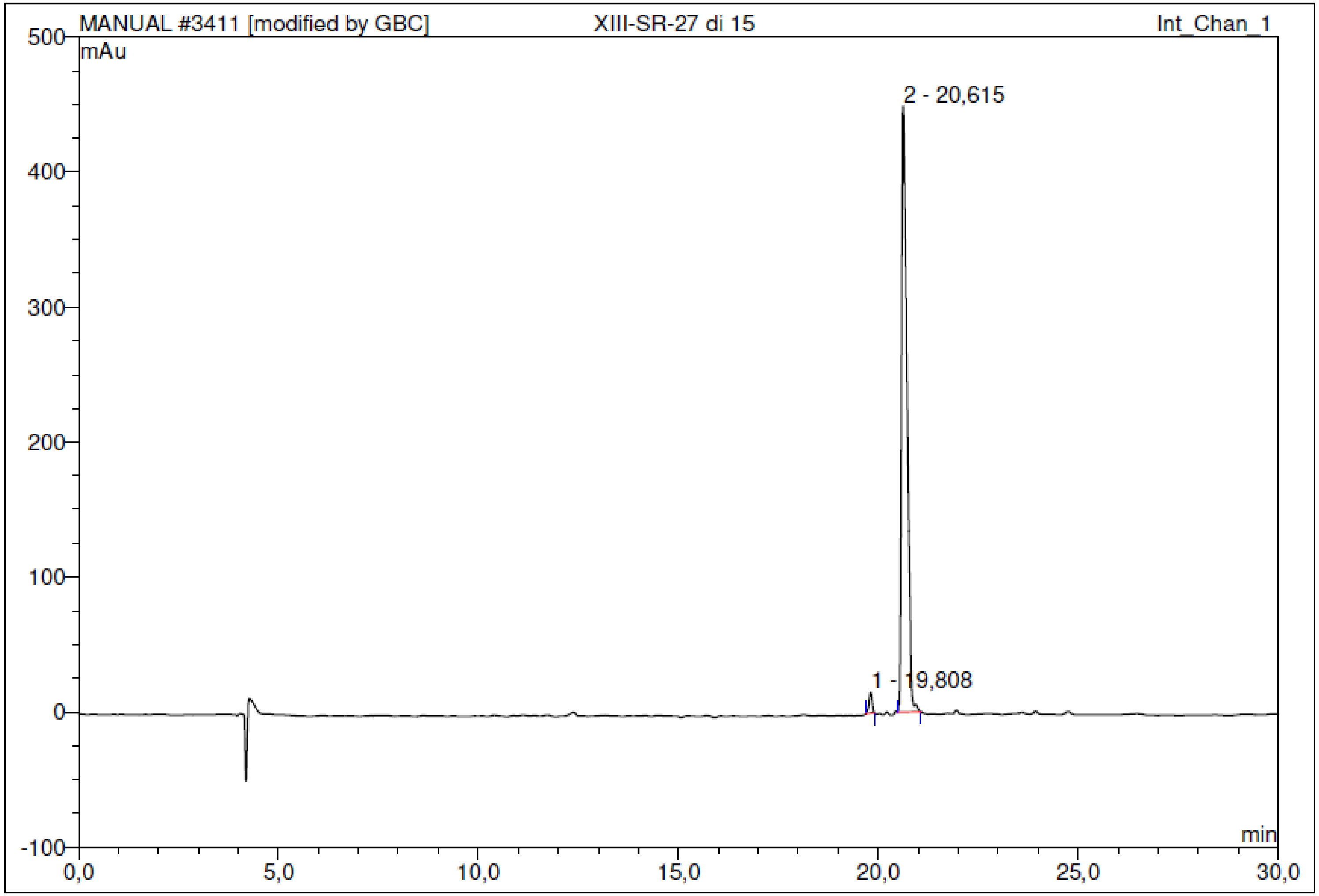

No.	Ret.Time min	Peak Name	Height mAu	Area mAu*min	Rel.Area %	Amount	Type
1	19,81	n.a.	15,266	1,385	1,83	n.a.	BMB*
2	20,61	n.a.	448,748	74,487	98,17	n.a.	BMB*
Total:			464,014	75,872	100,00	0,000	

Figure S32. HPLC analysis of **15**.

Spectrum Name: XIII-SR-27_pt
Start Ion: 50
End Ion: 800
Source: APCI + 10.0µA 400C
Capillary: 150V 300C Offset: 25V Span: 0V

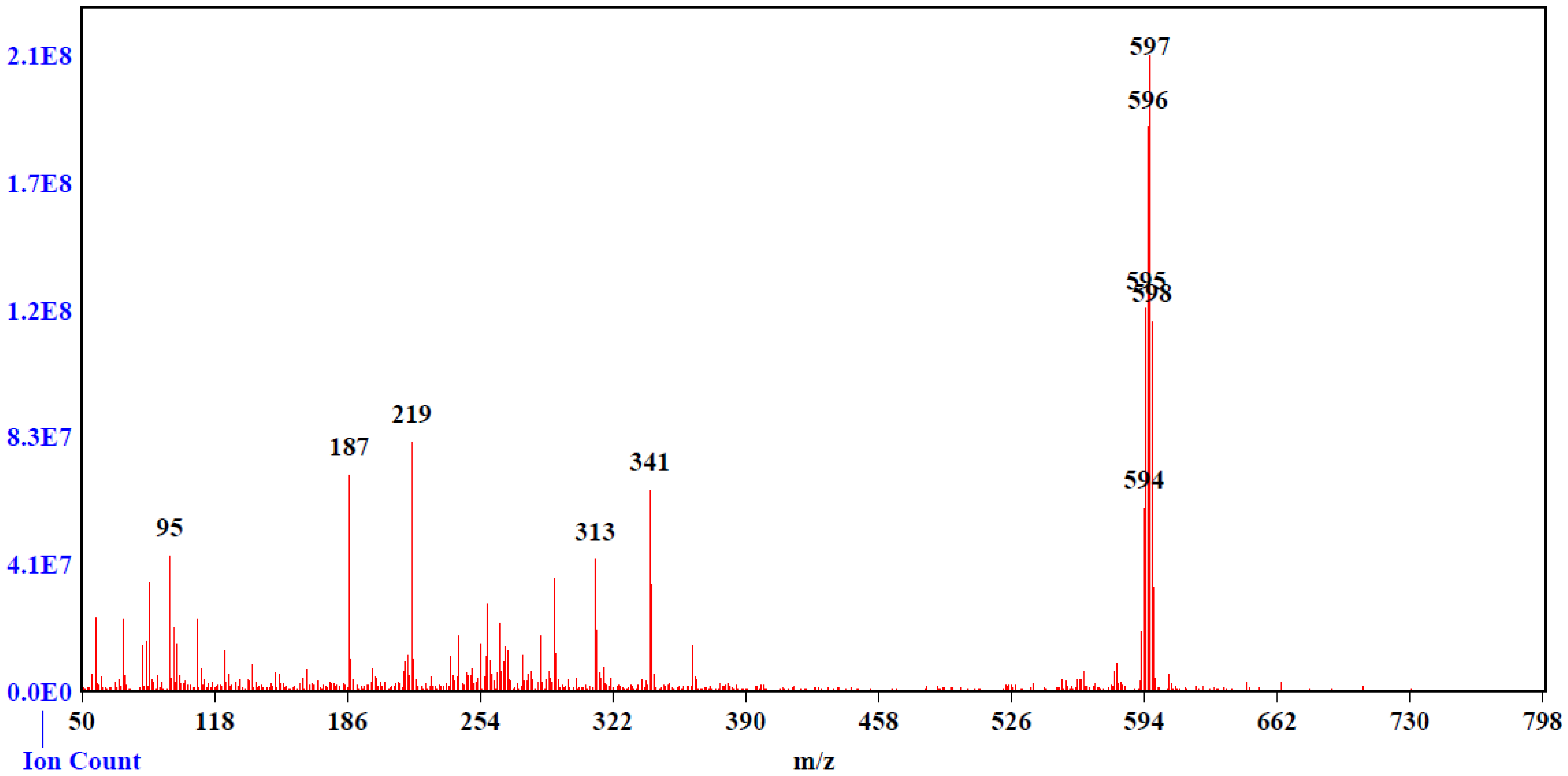

Figure S33. MS spectrum of **15**.

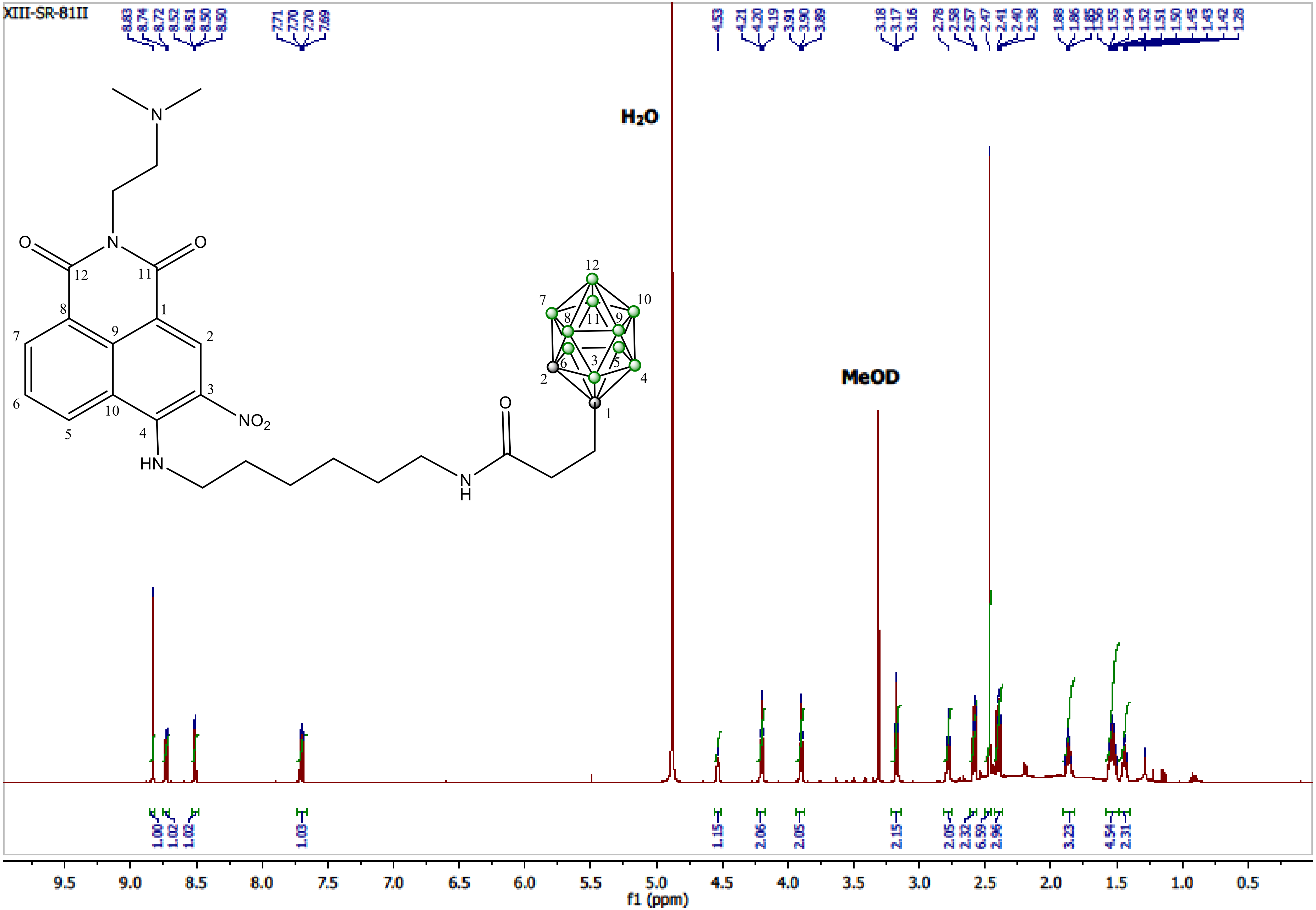

Figure S34. ^{1}H-NMR spectrum of **16**.

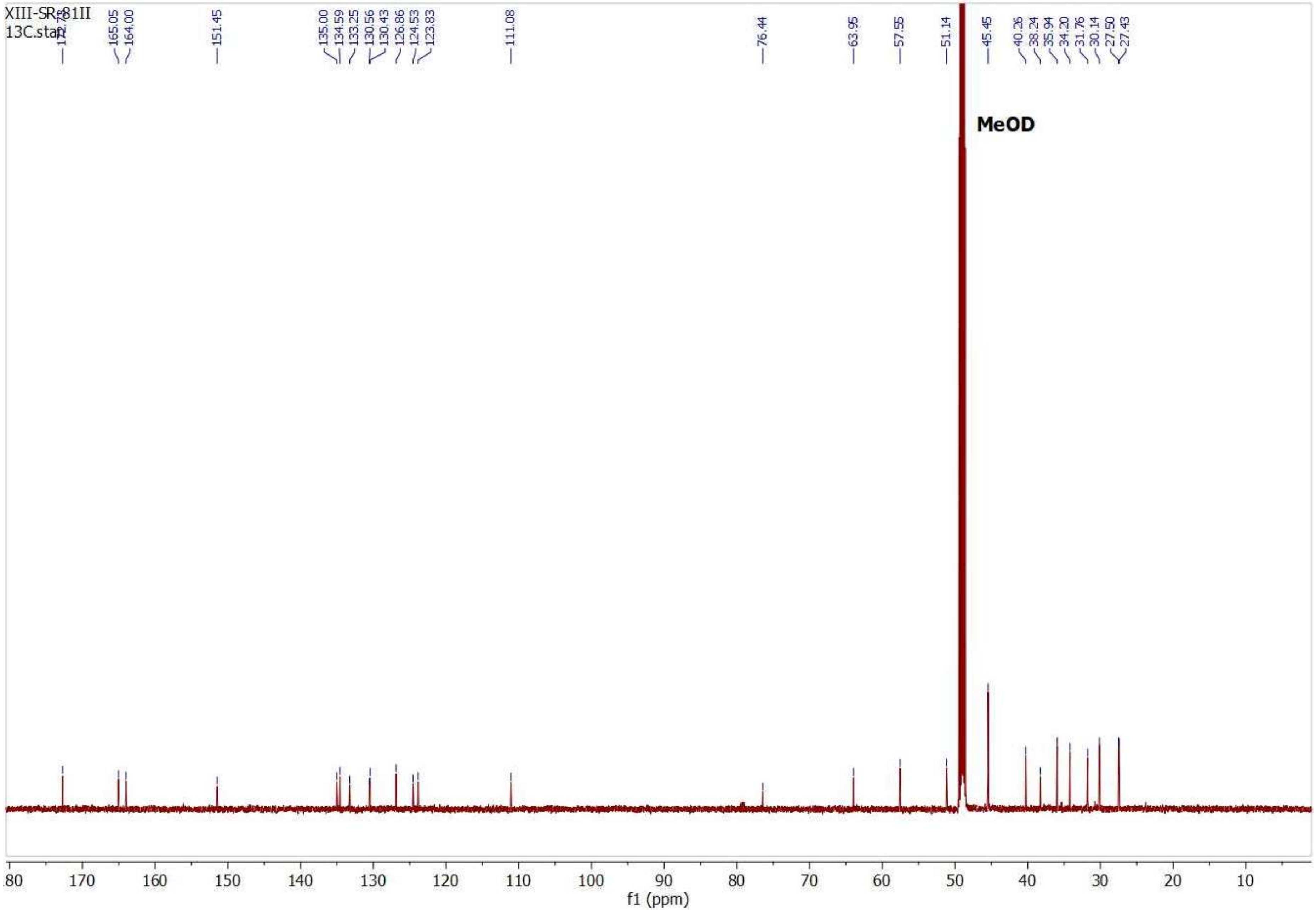

Figure S35. ^{13}C-NMR spectrum of **16**.

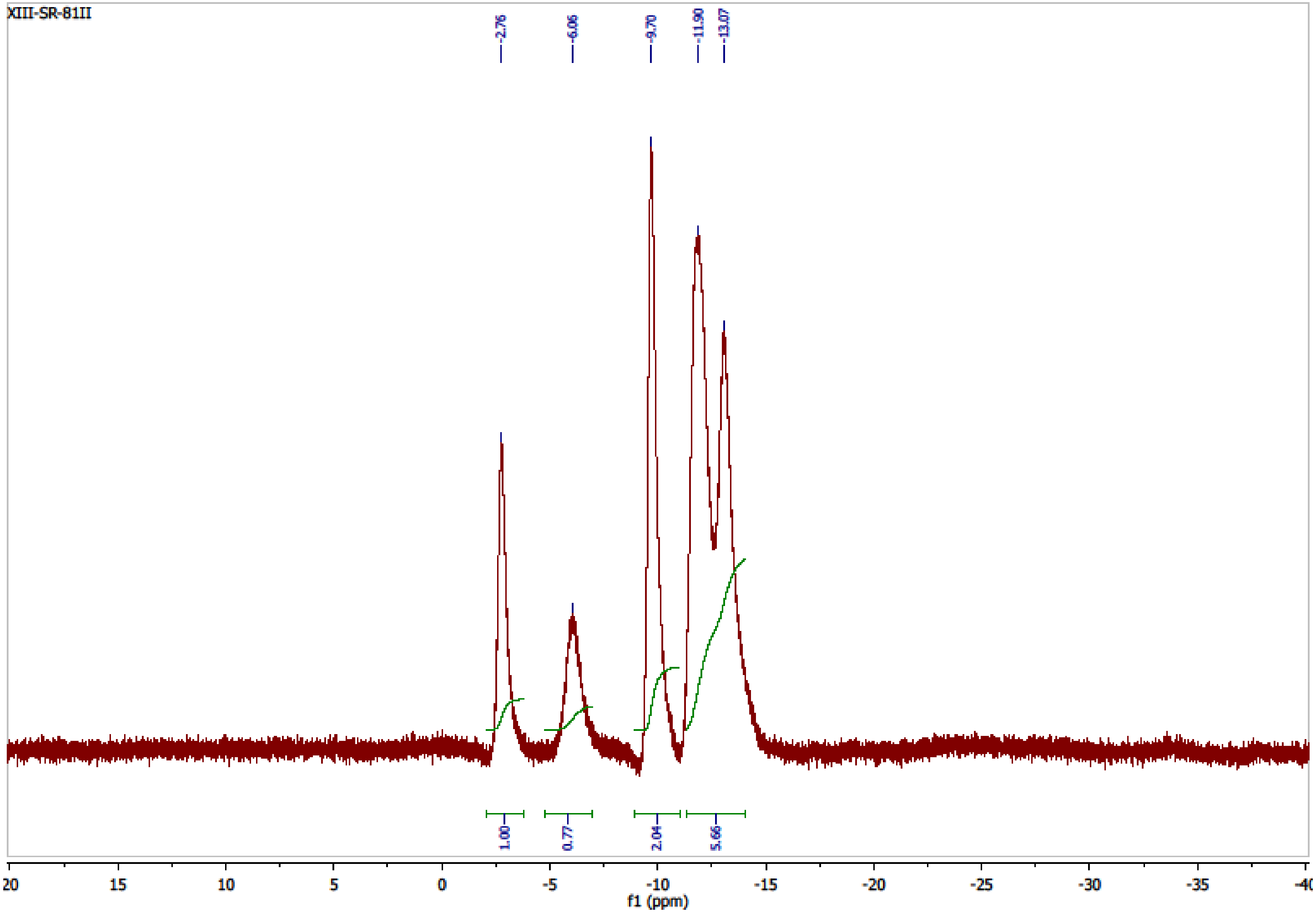

Figure S36. ^{11}B-NMR {^{1}H BB} spectrum of **16**.

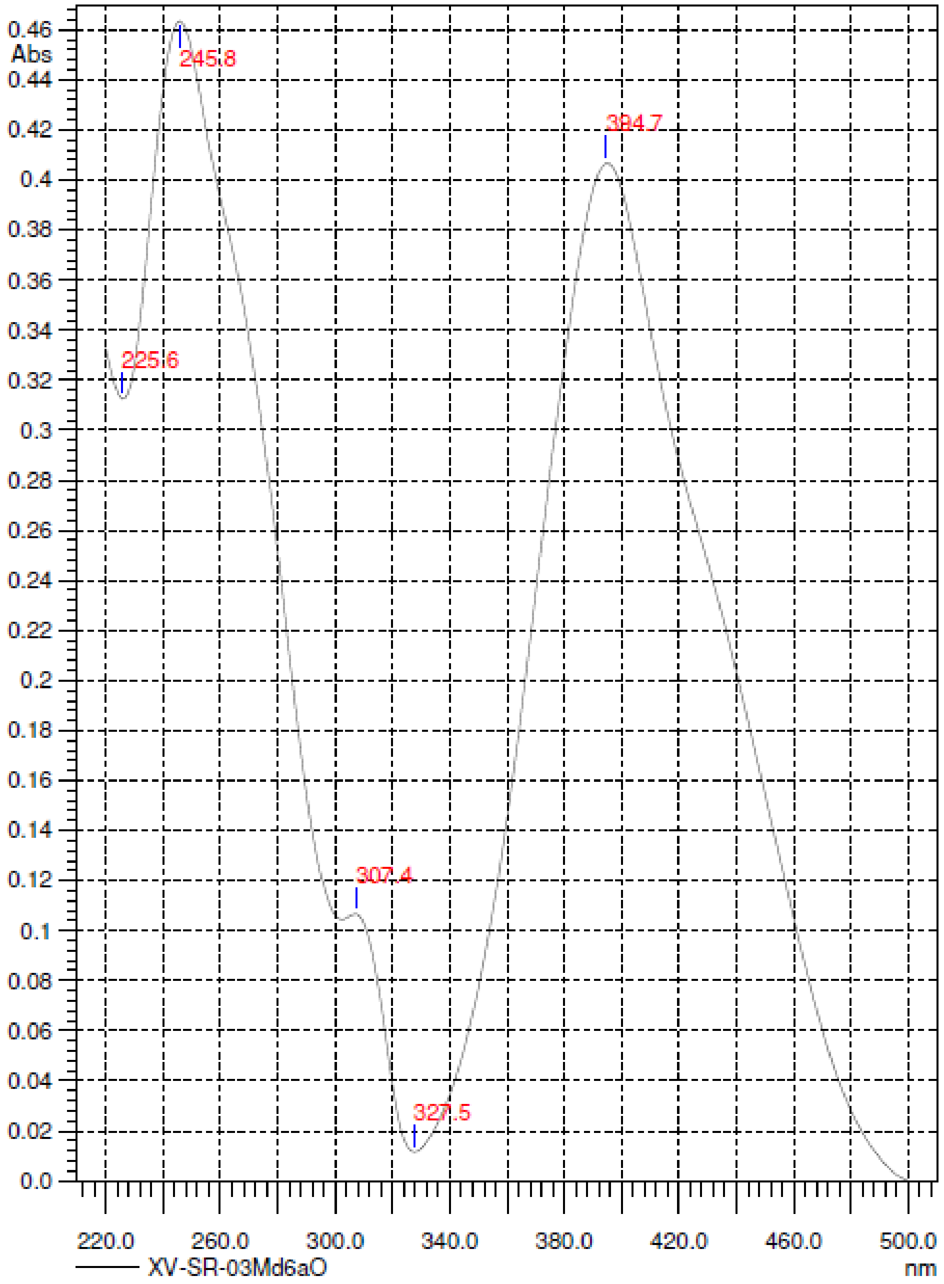

Figure S37. UV spectrum of **16**.

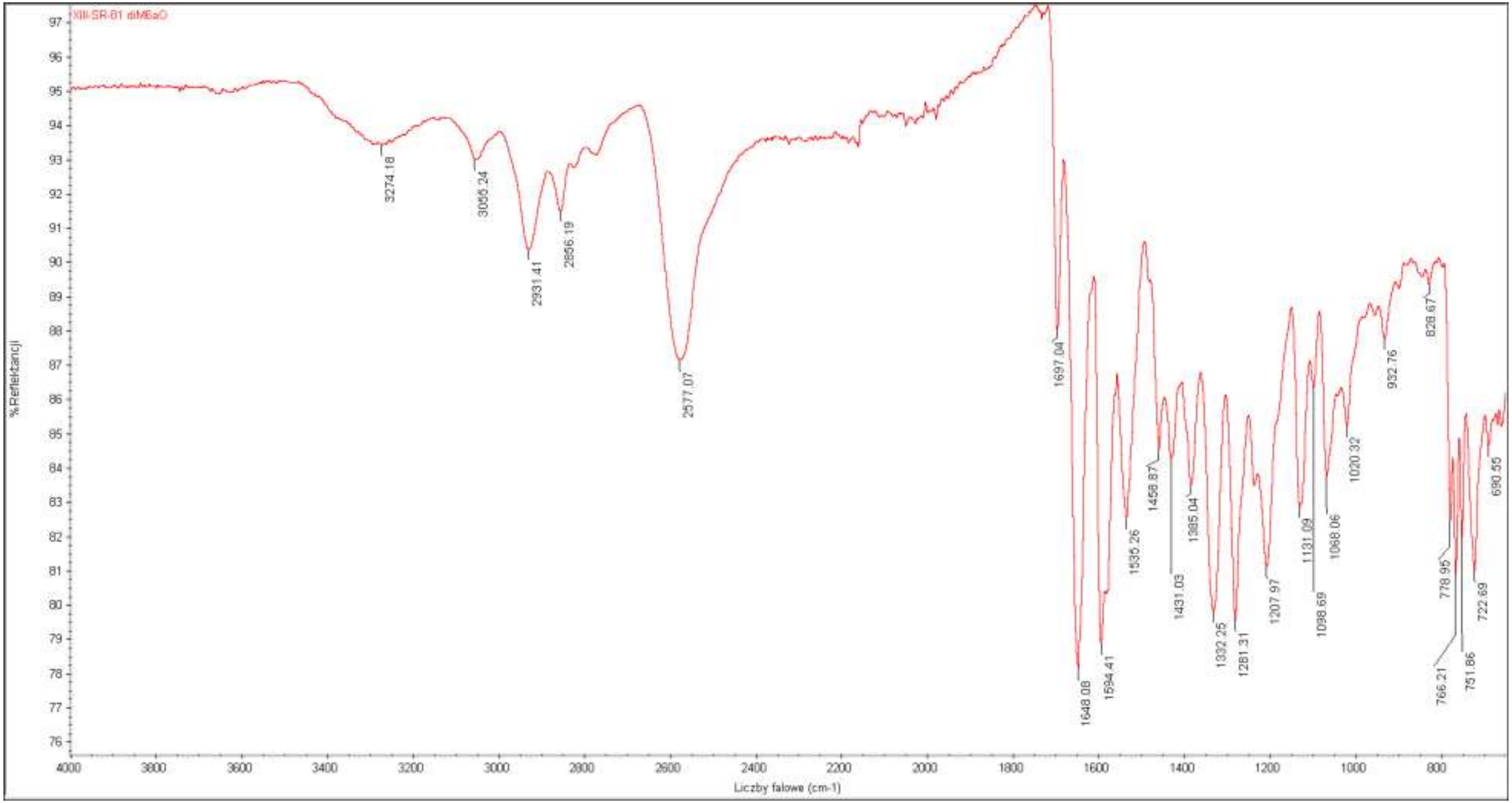

Figure S38. IR spectrum of **16**.

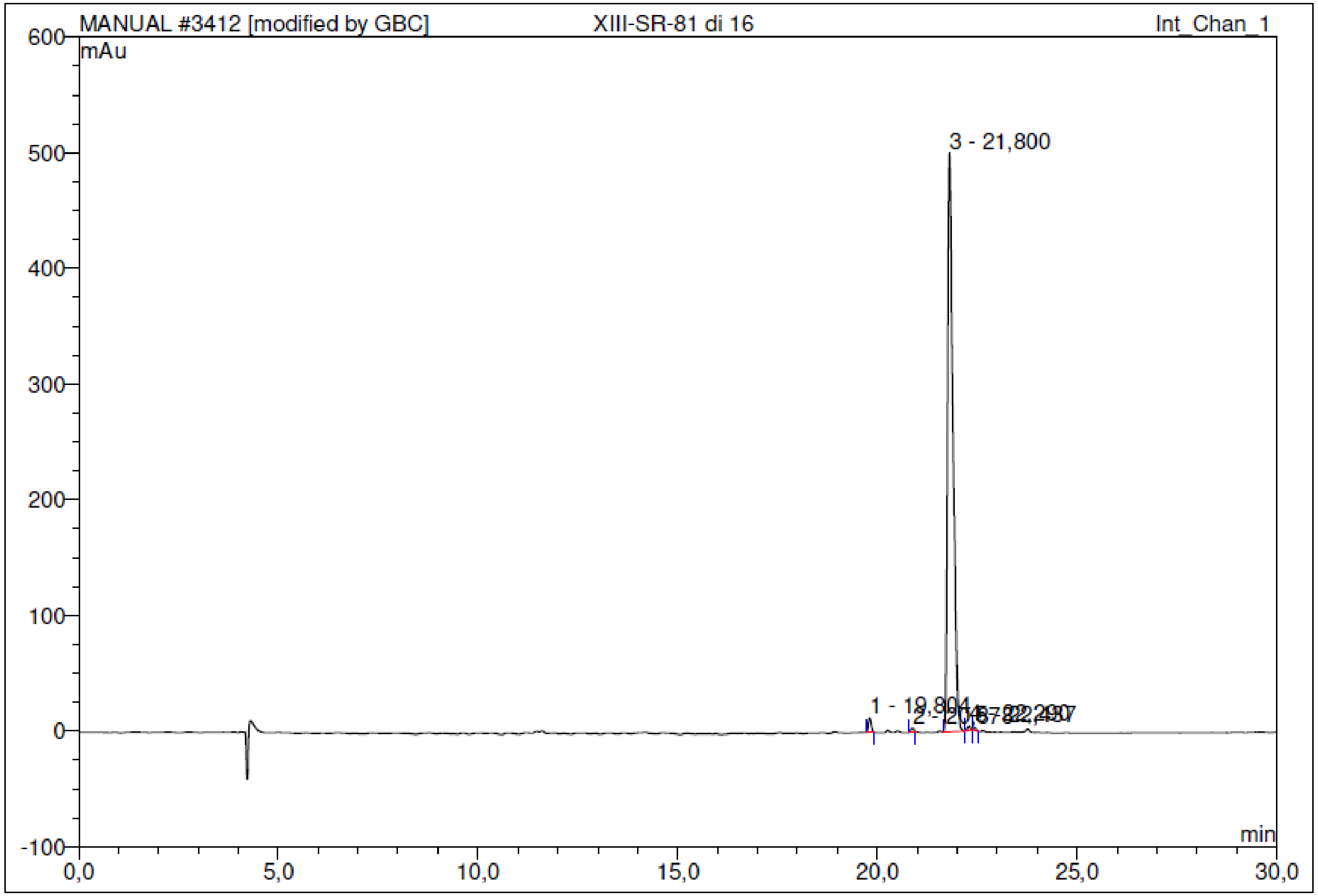

No.	Ret.Time min	Peak Name	Height mAu	Area mAu*min	Rel.Area %	Amount	Type
1	19,80	n.a.	12,151	0,982	1,19	n.a.	BMB*
2	20,88	n.a.	3,788	0,292	0,35	n.a.	BMB*
3	21,80	n.a.	500,521	81,053	97,95	n.a.	BMB
4	22,29	n.a.	3,597	0,273	0,33	n.a.	BMb*
5	22,44	n.a.	2,320	0,151	0,18	n.a.	bMB*
Total:			522,377	82,750	100,00	0,000	

Figure S39. HPLC analysis of **16**.

Spectrum Name: XIII-SR-81_pt
Start Ion: 200
End Ion: 800
Source: APCI + 10.0µA 400C
Capillary: 150V 300C Offset: 25V Span: 0V

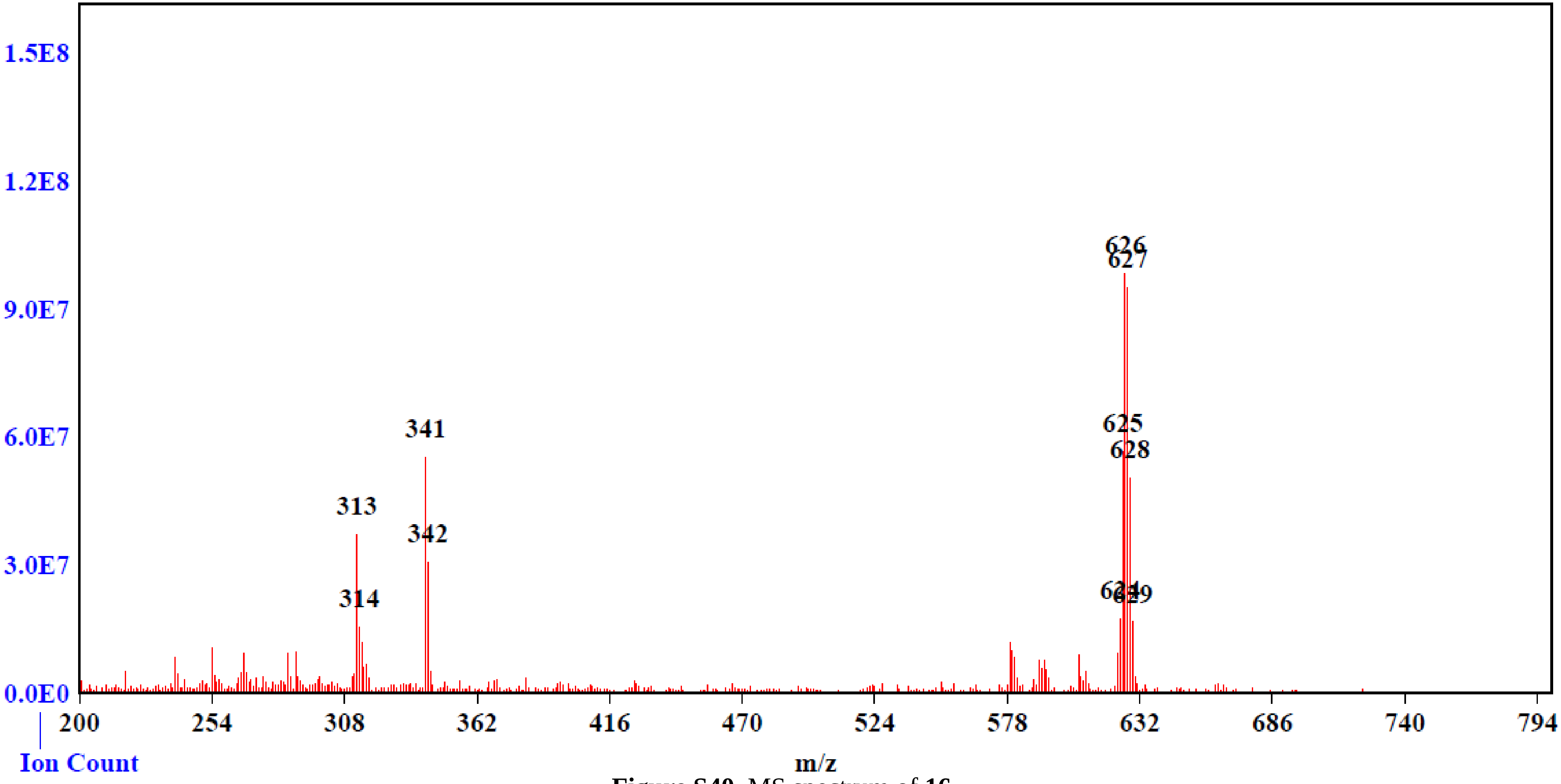

Figure S40. MS spectrum of **16**.

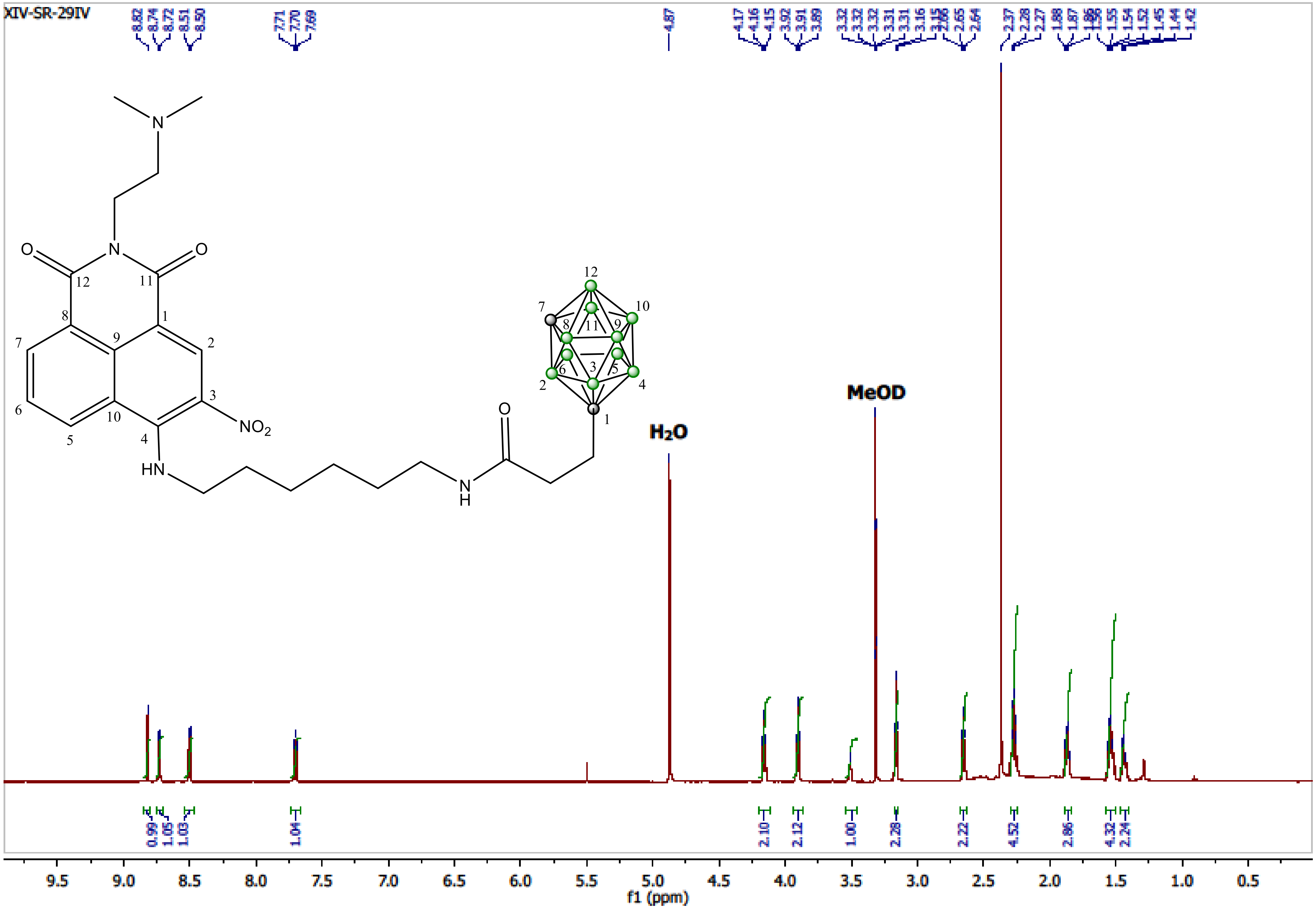

Figure S41. ^{1}H-NMR spectrum of **17**.

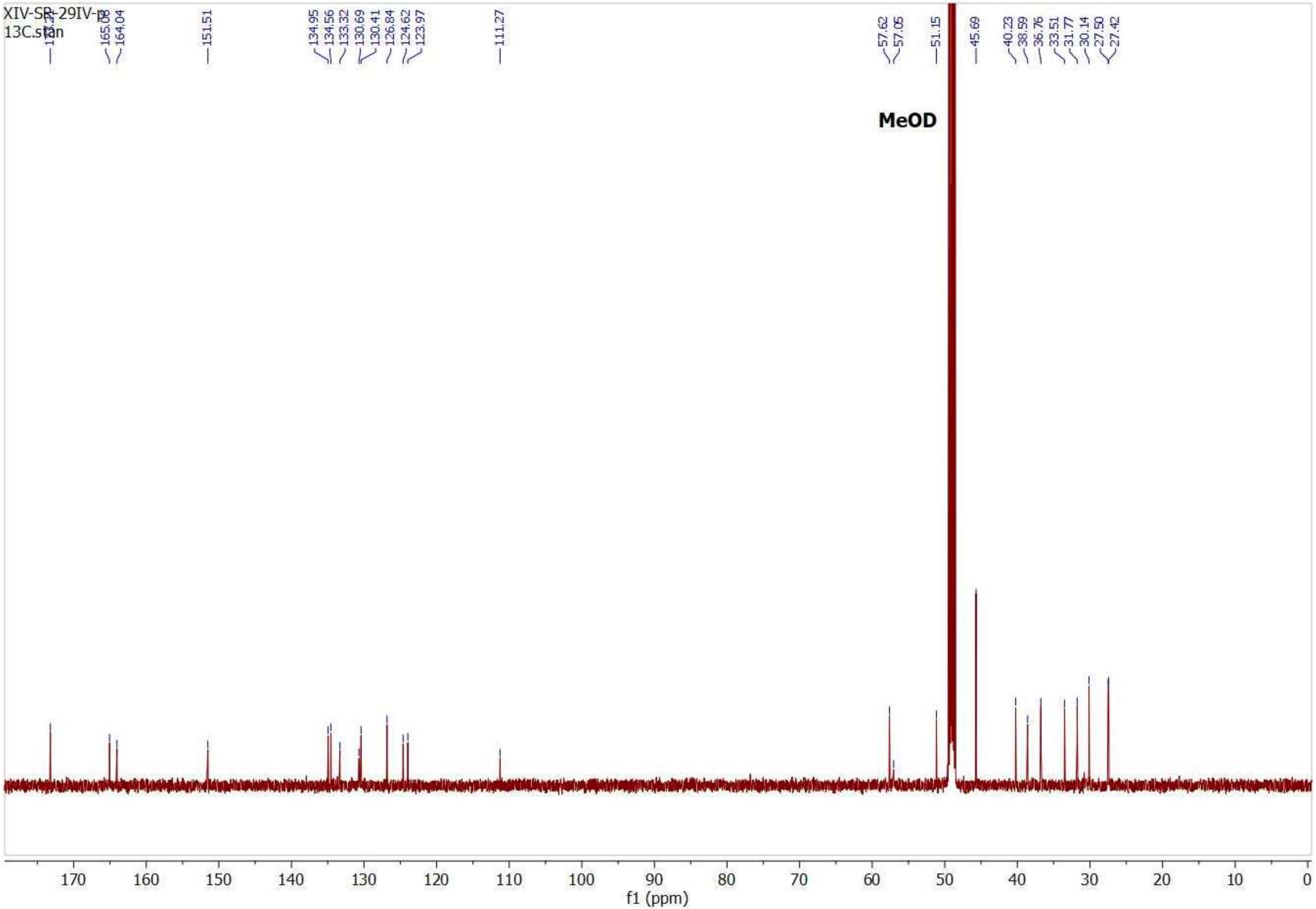

Figure S42. ^{13}C-NMR spectrum of **17**.

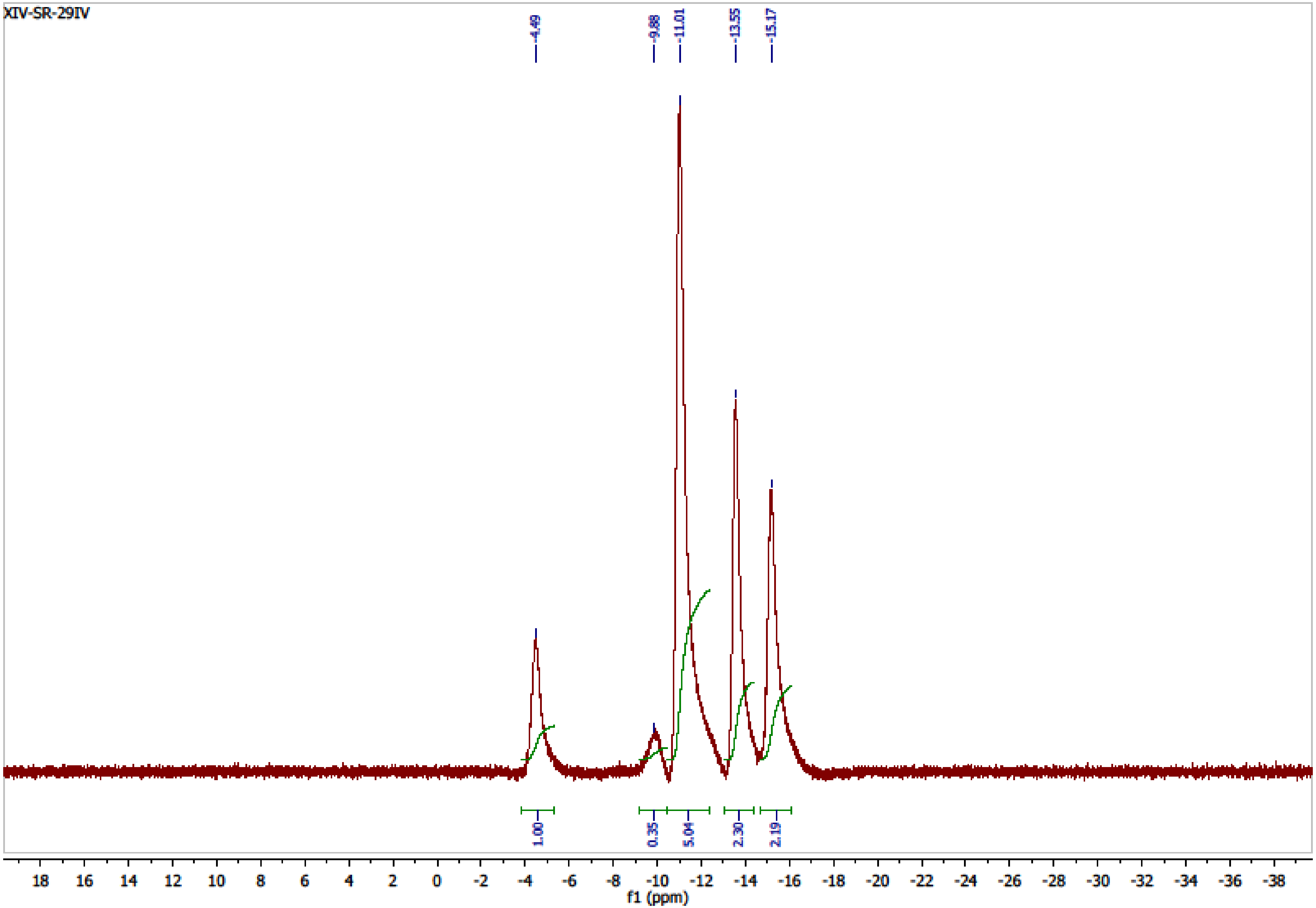

Figure S43. ^{11}B-NMR {^{1}H BB} spectrum of **17**.

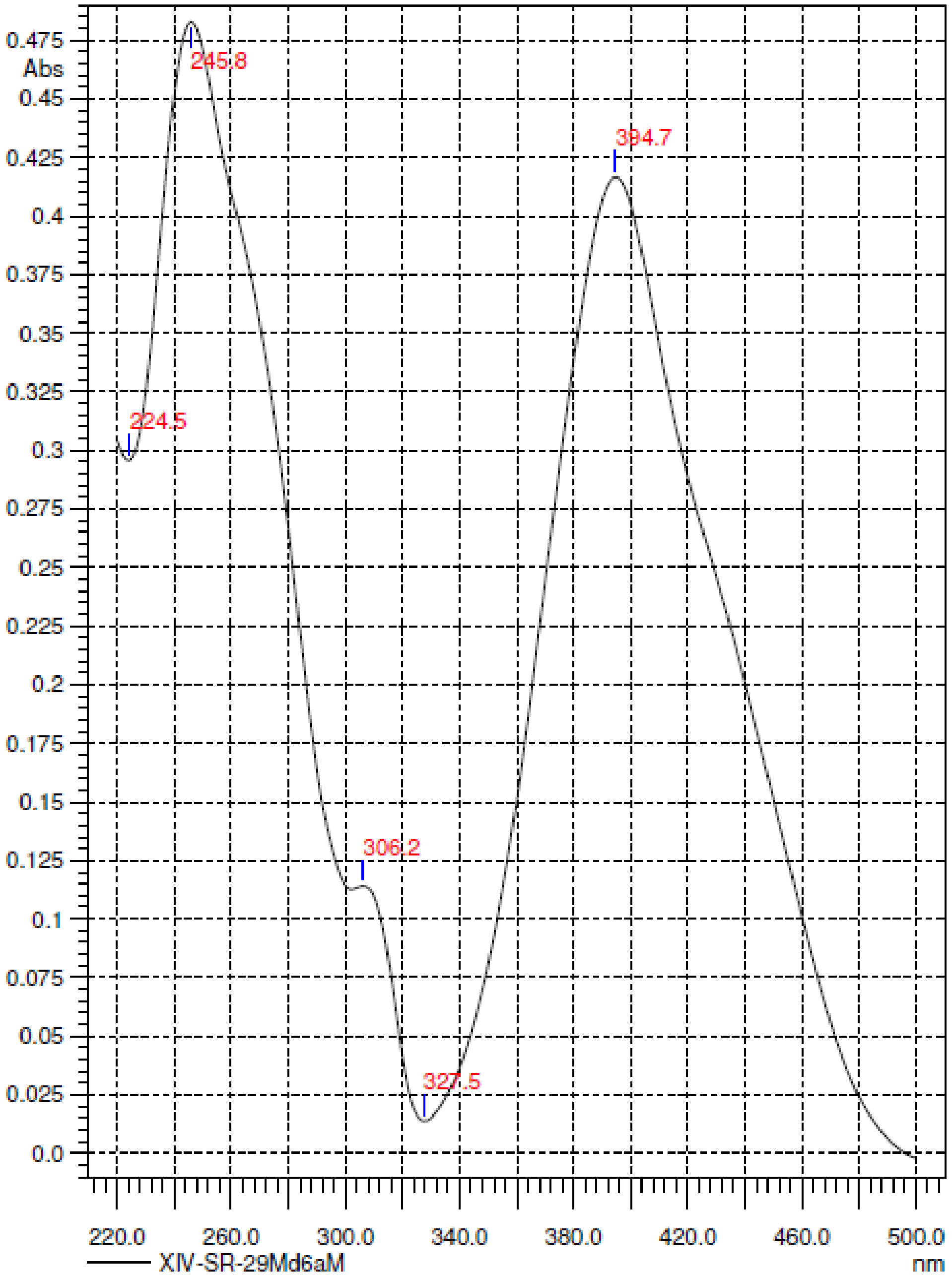

Figure S44. UV spectrum of **17**.

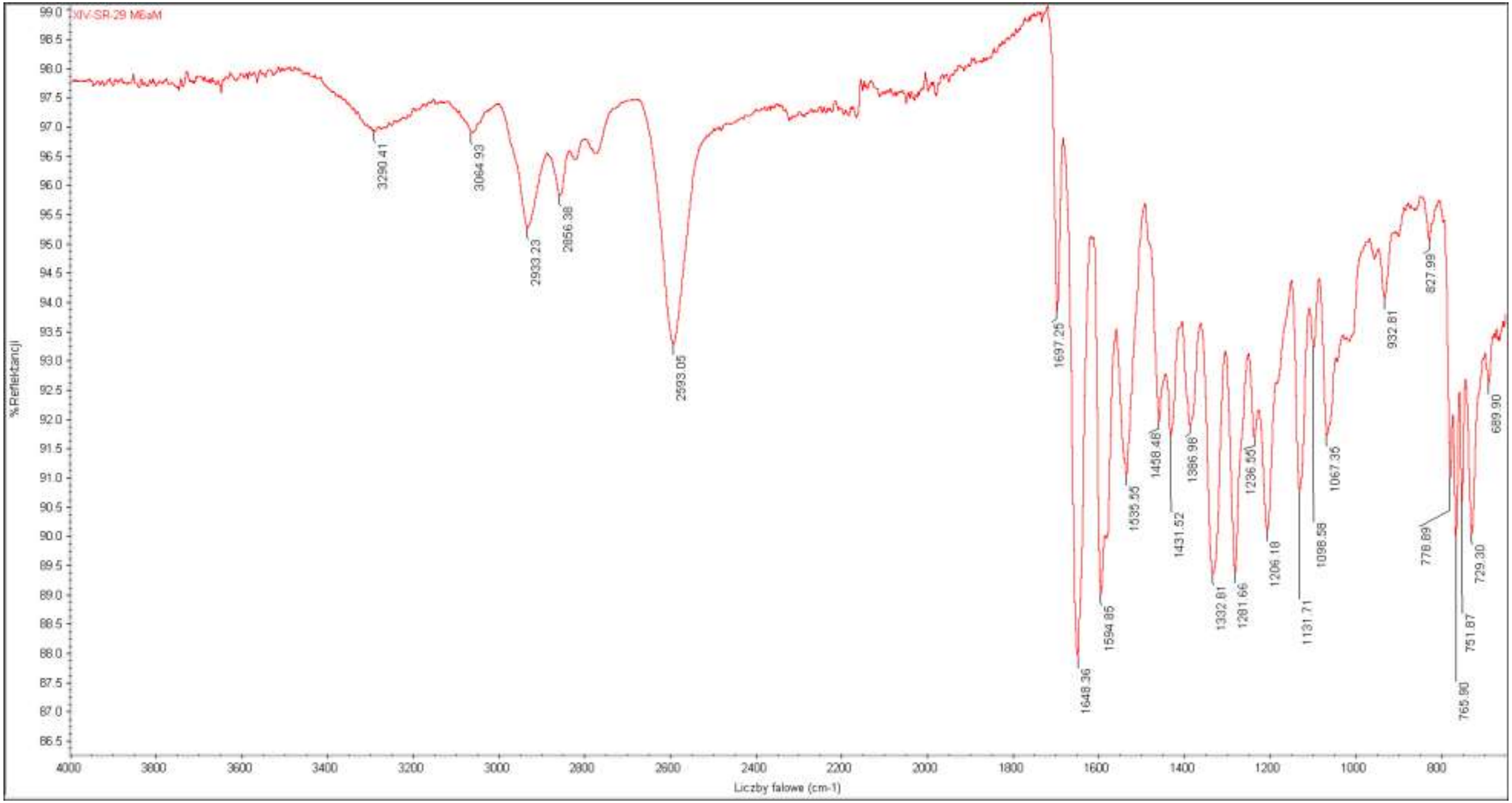

Figure S45. IR spectrum of **17**.

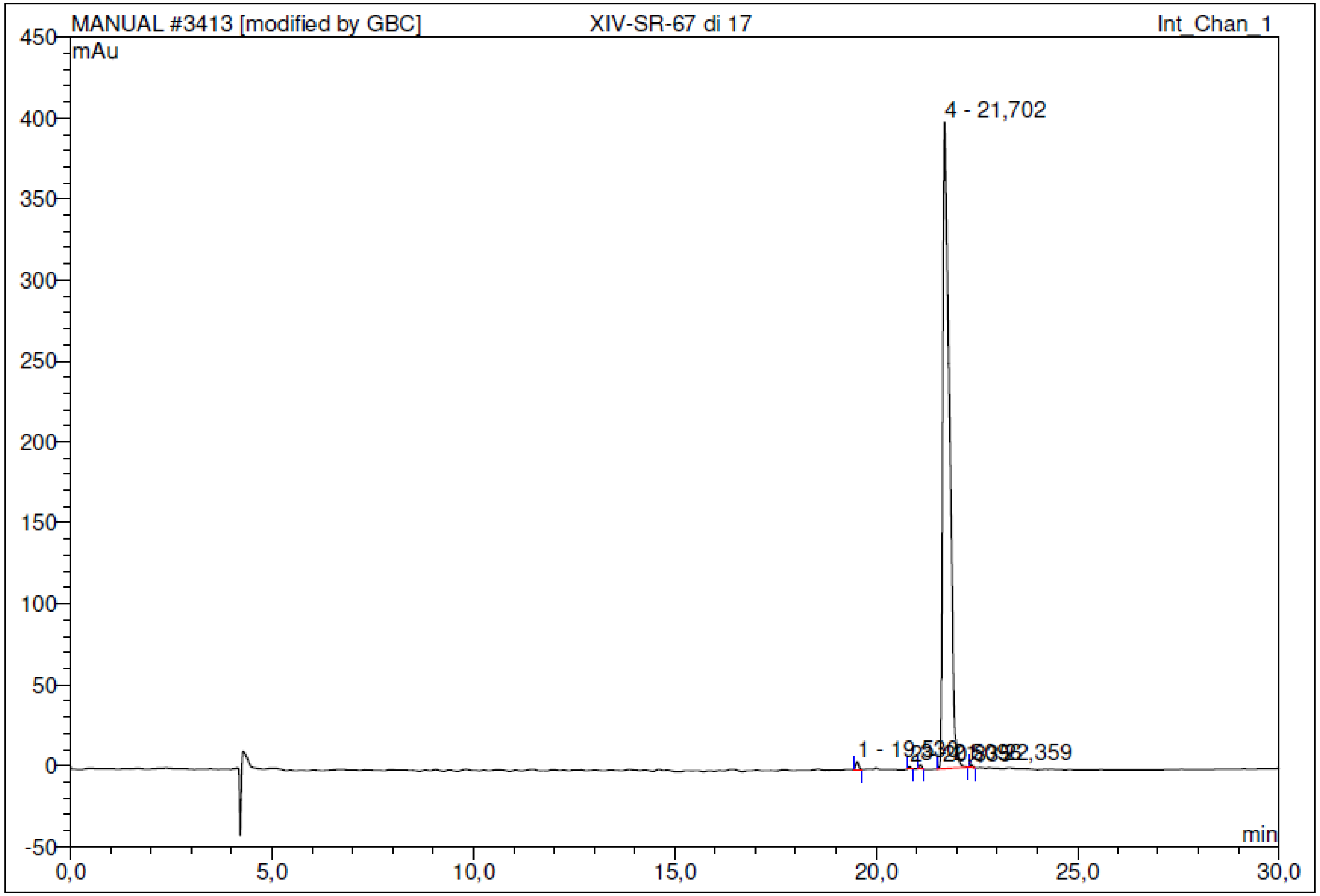

No.	Ret.Time min	Peak Name	Height mAu	Area mAu*min	Rel.Area %	Amount	Type
1	19,53	n.a.	4,357	0,345	0,45	n.a.	BMB*
2	20,84	n.a.	1,570	0,106	0,14	n.a.	BMB*
3	21,10	n.a.	2,061	0,148	0,19	n.a.	BMB*
4	21,70	n.a.	399,510	76,322	99,09	n.a.	BMB
5	22,36	n.a.	1,355	0,106	0,14	n.a.	BMB*
Total:			408,852	77,027	100,00	0,000	

Figure S46. HPLC analysis of **17**.

Spectrum Name: XIV-SR-67_pt
Start Ion: 100
End Ion: 700
Source: APCI + 10.0µA 400C
Capillary: 150V 300C Offset: 25V Span: 0V

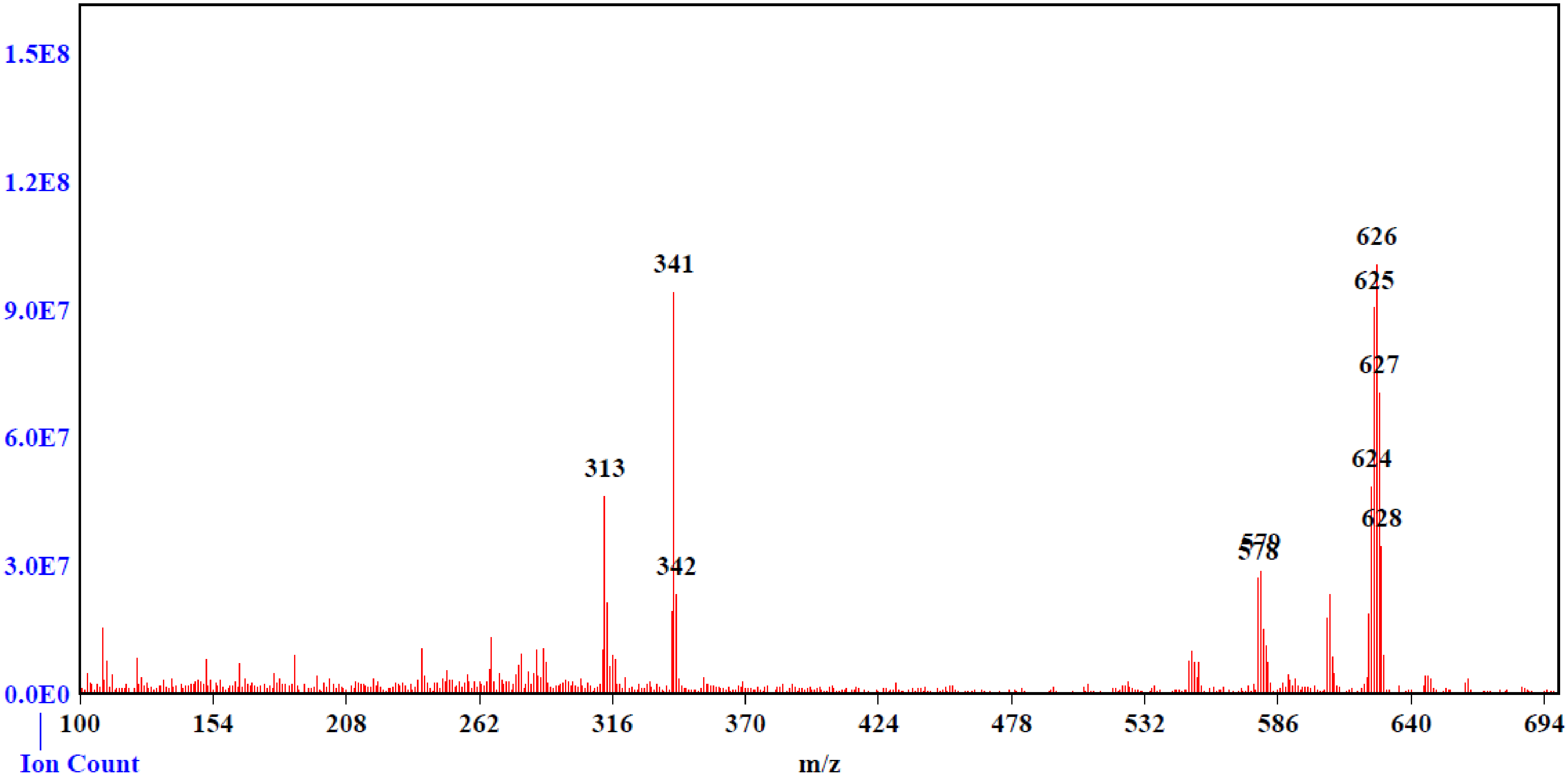

Figure S47. MS spectrum of **17**.

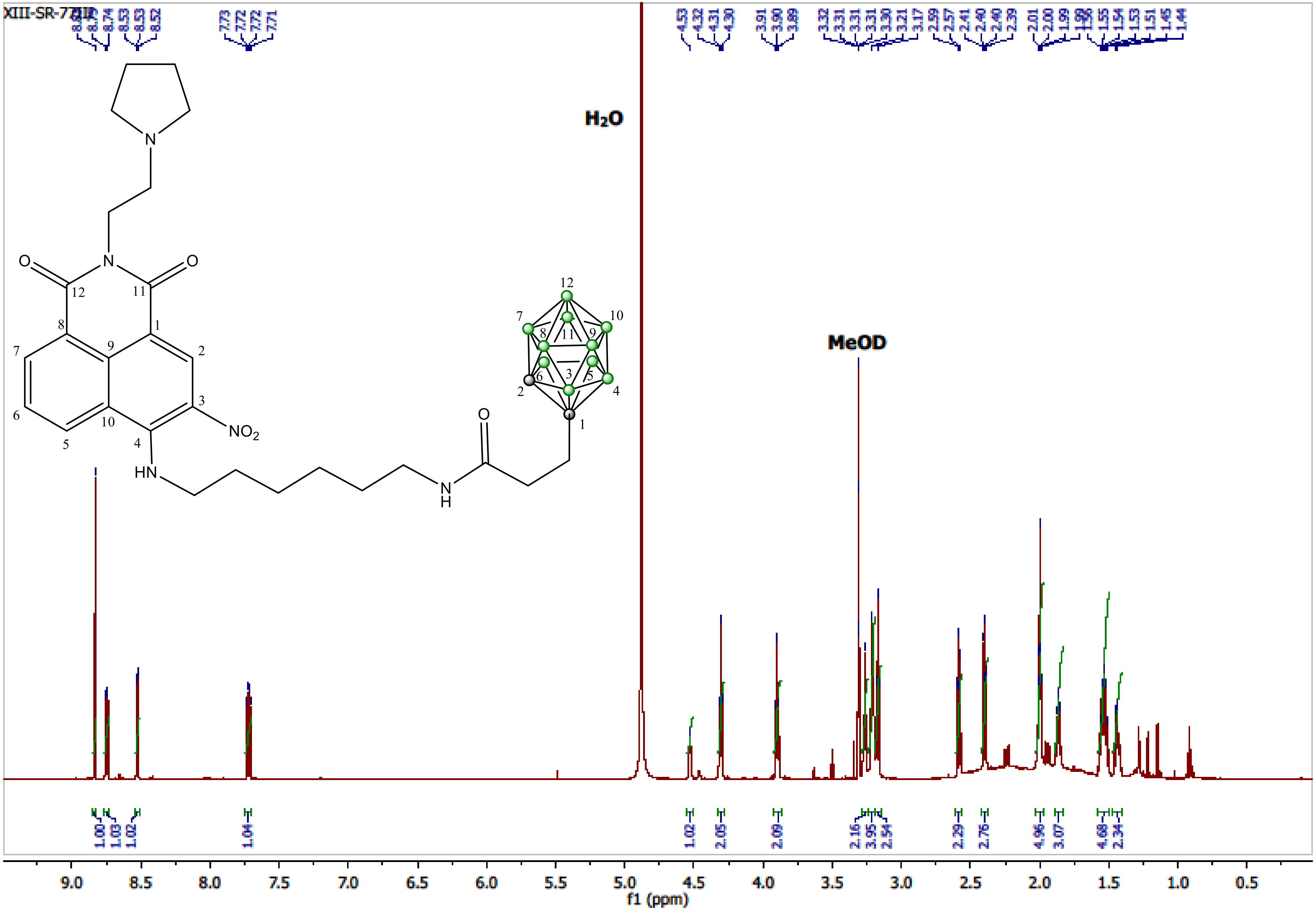

Figure S48. ^{1}H-NMR spectrum of **18**.

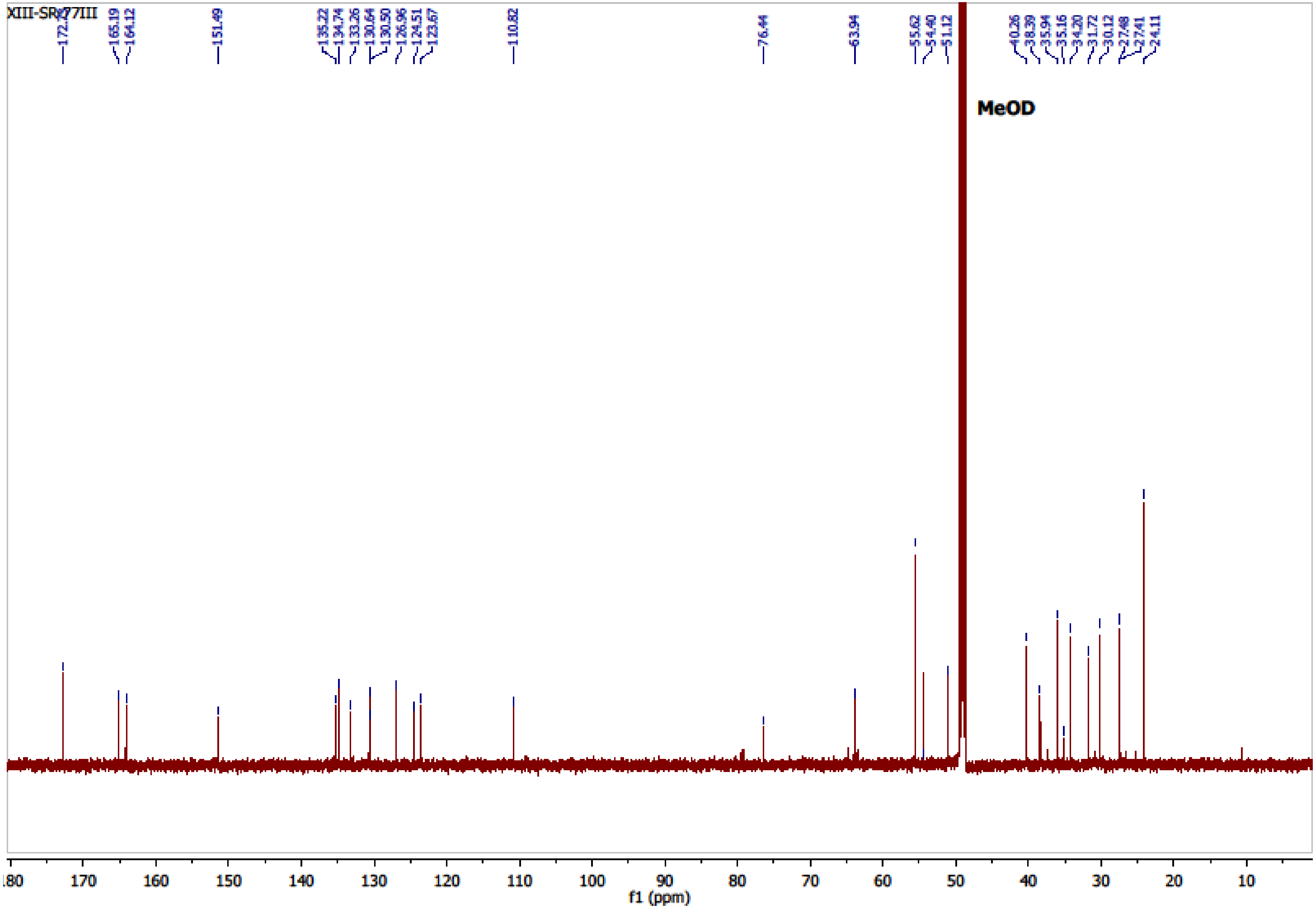

Figure S49. ^{13}C-NMR spectrum of **18**.

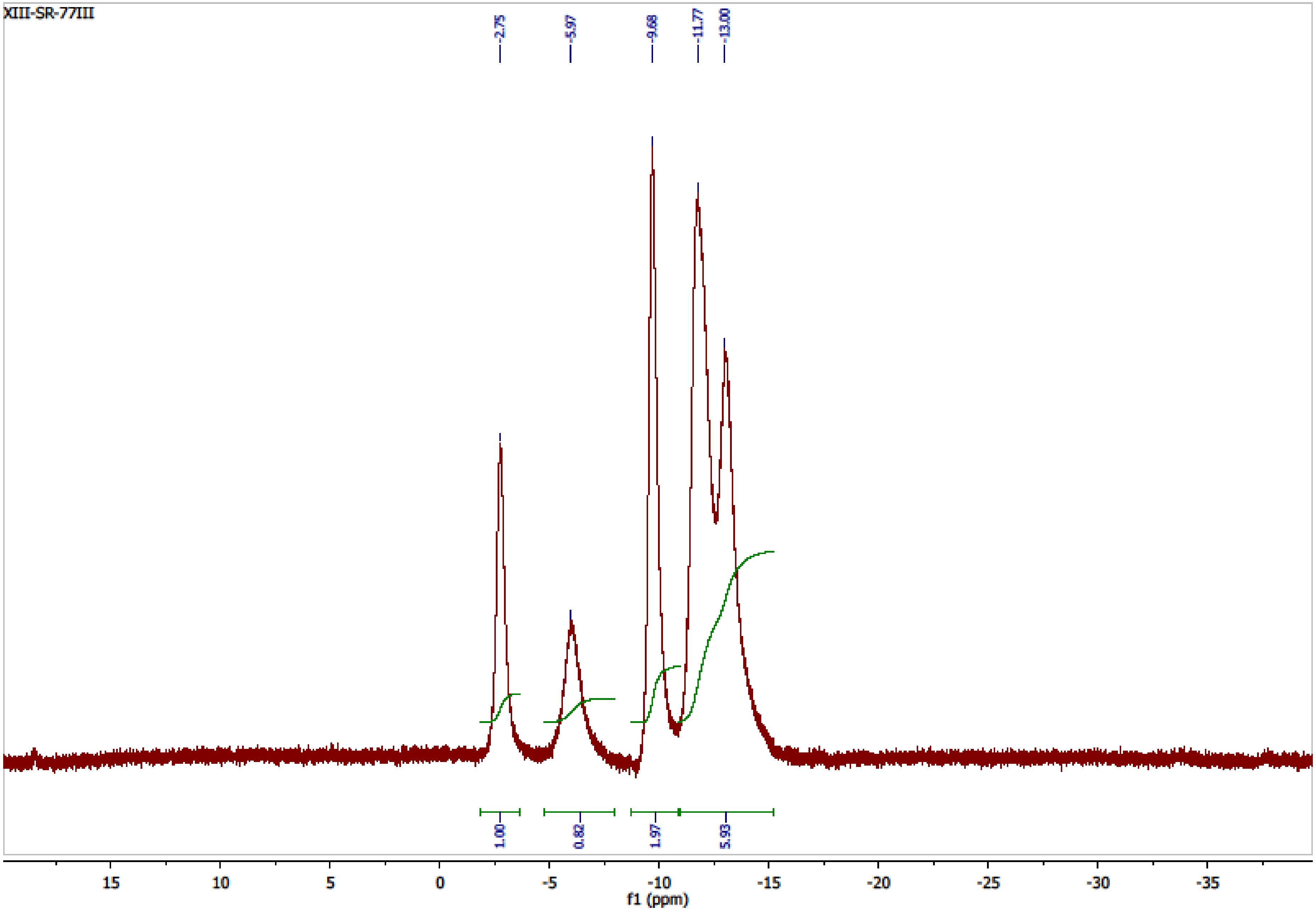

Figure S50. ^{11}B-NMR {^{1}H BB} spectrum of **18**.

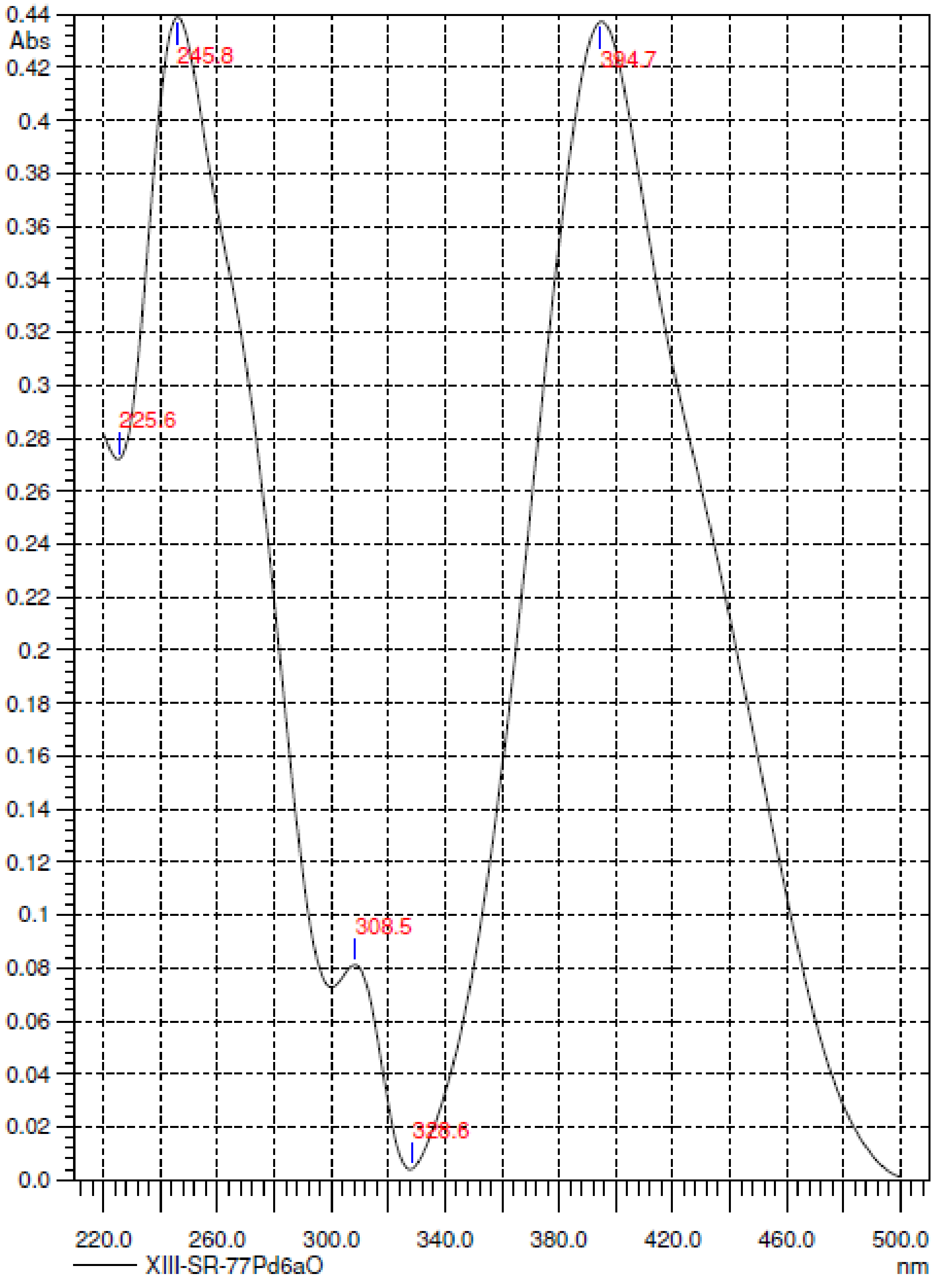

Figure S51. UV spectrum of **18**.

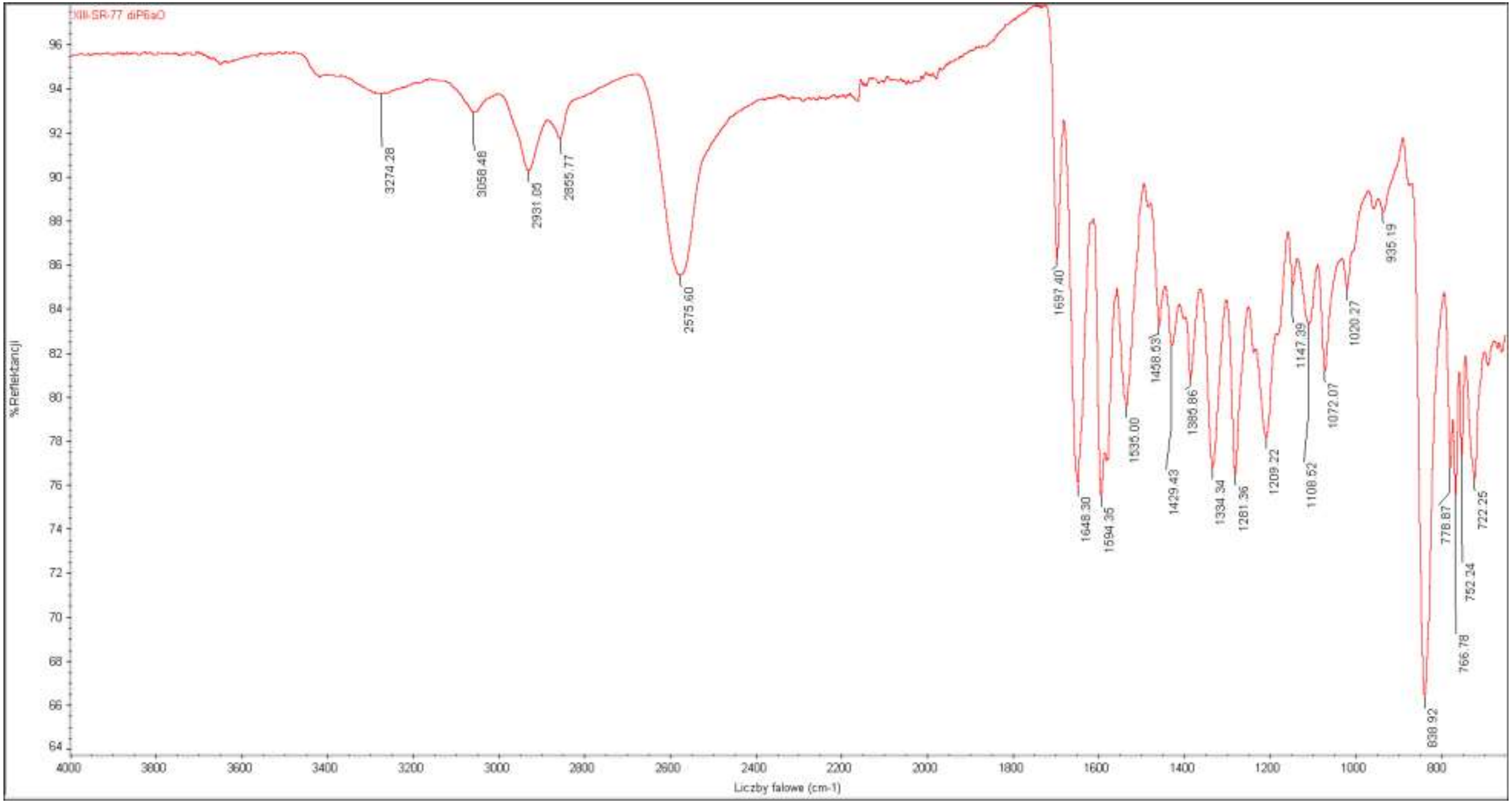

Figure S52. IR spectrum of **18**.

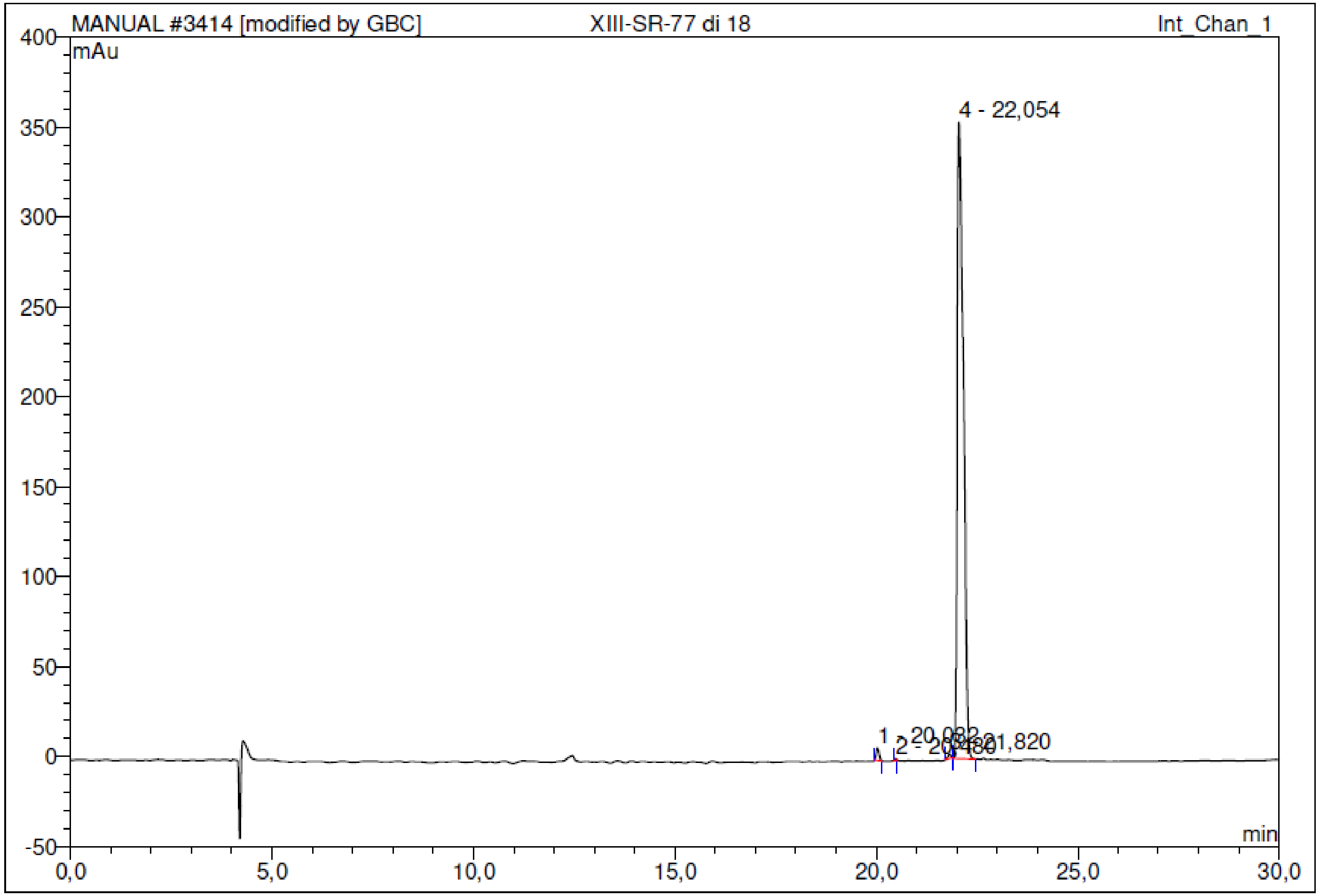

No.	Ret.Time min	Peak Name	Height mAu	Area mAu*min	Rel.Area %	Amount	Type
1	20,03	n.a.	6,908	0,554	0,87	n.a.	BMB*
2	20,48	n.a.	0,932	0,054	0,08	n.a.	BMB*
3	21,82	n.a.	2,642	0,204	0,32	n.a.	BMB*
4	22,05	n.a.	353,901	63,199	98,73	n.a.	BMB
Total:			364,383	64,012	100,00	0,000	

Figure S53. HPLC analysis of **18**.

Spectrum Name: XIII-SR-77_pt
Start Ion: 200
End Ion: 700
Source: APCI + 10.0µA 400C
Capillary: 150V 300C Offset: 25V Span: 0V

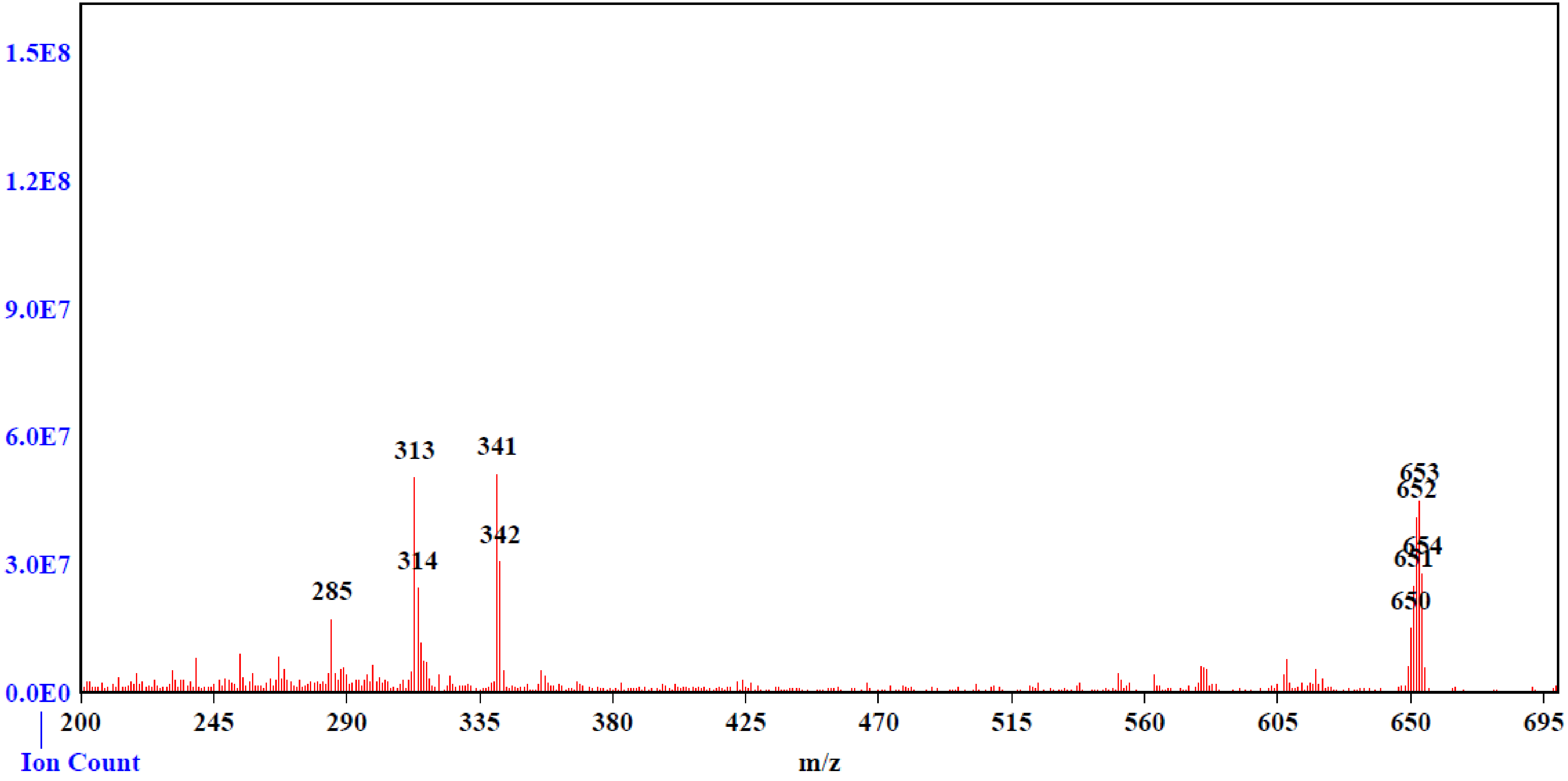

Figure S54. MS spectrum of **18**.

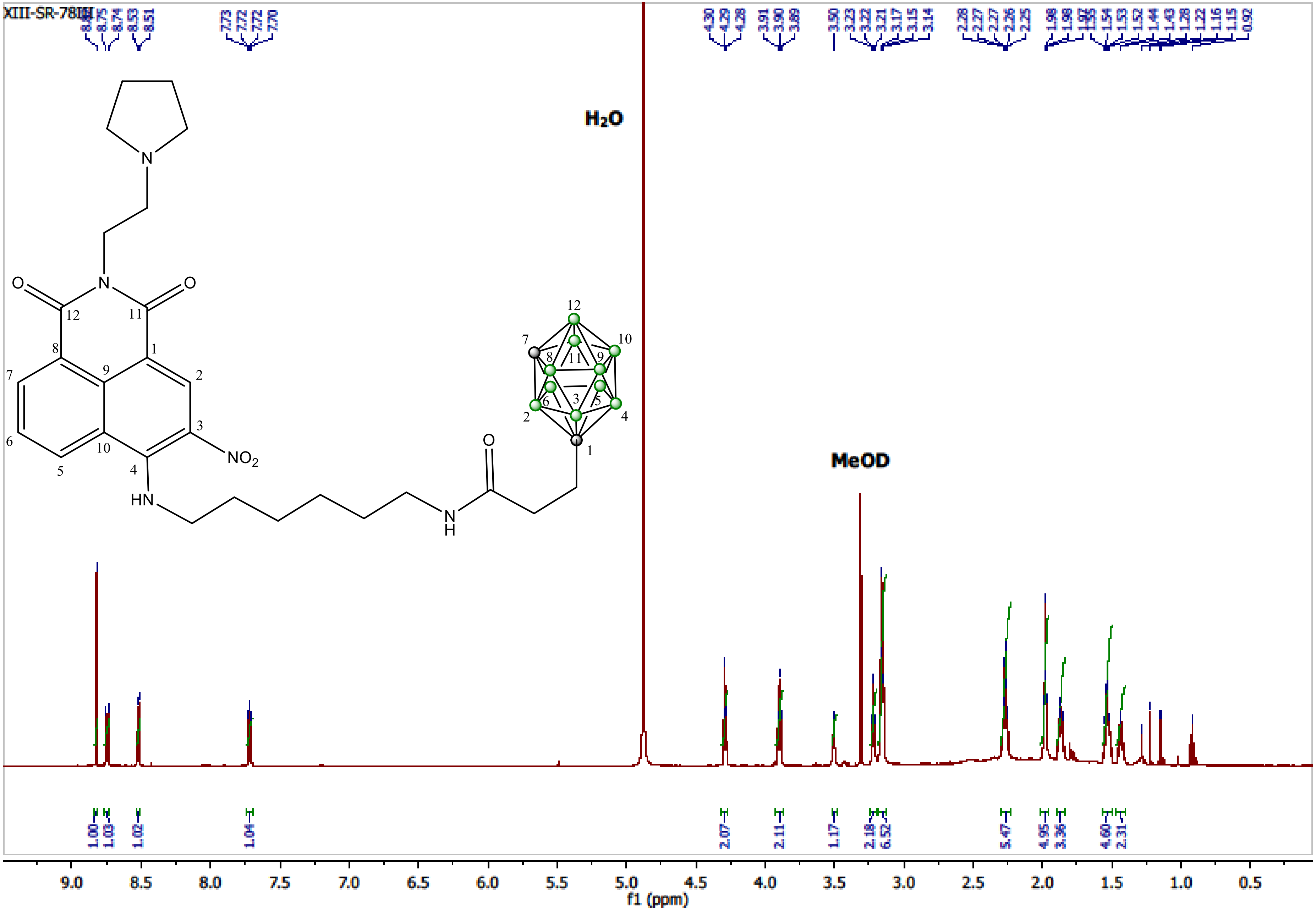

Figure S55. ^{1}H-NMR spectrum of **19**.

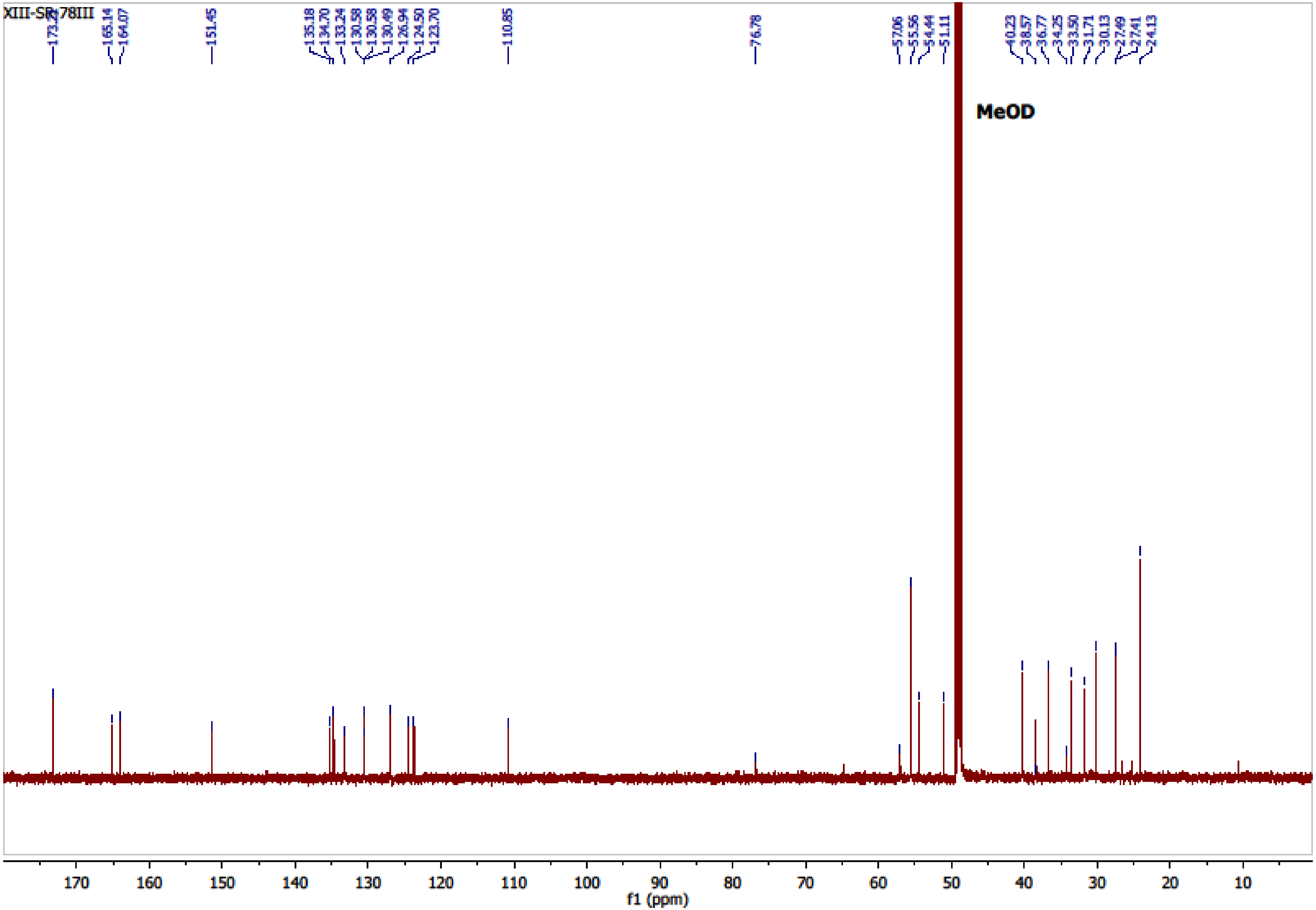

Figure S56. ^{13}C-NMR spectrum of **19**.

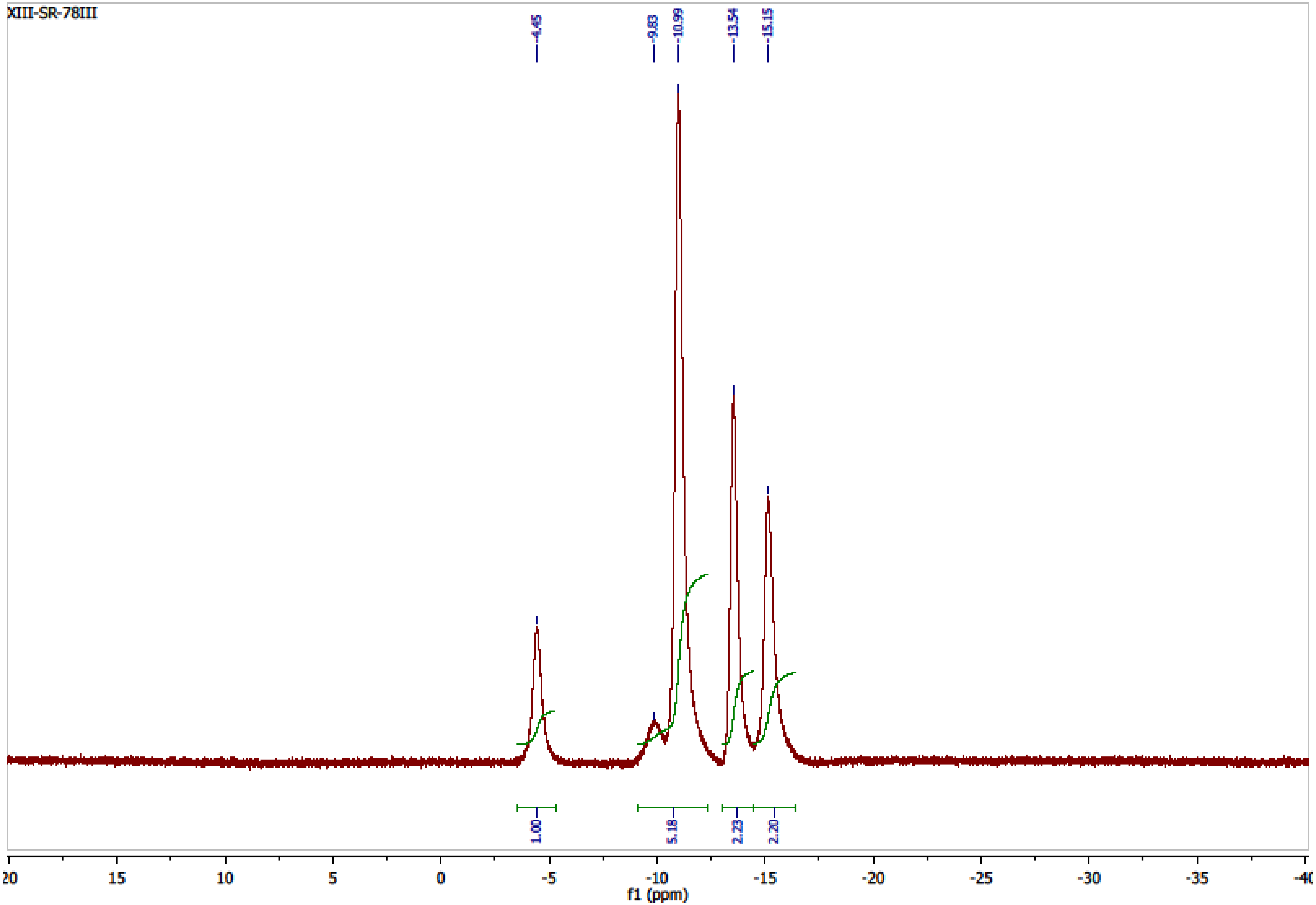

Figure S57. ^{11}B-NMR {^{1}H BB} spectrum of **19**.

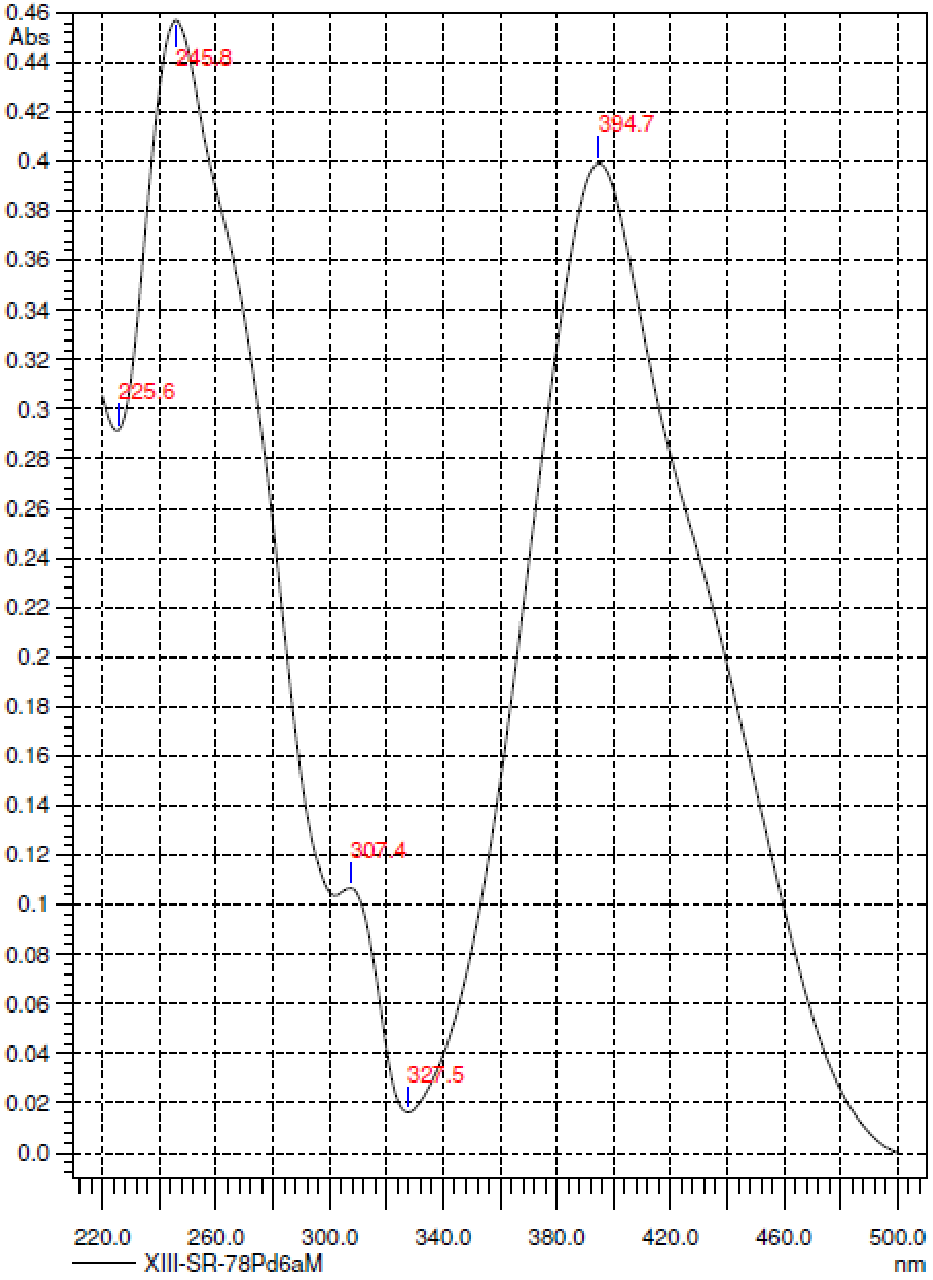

Figure S58. UV spectrum of **19**.

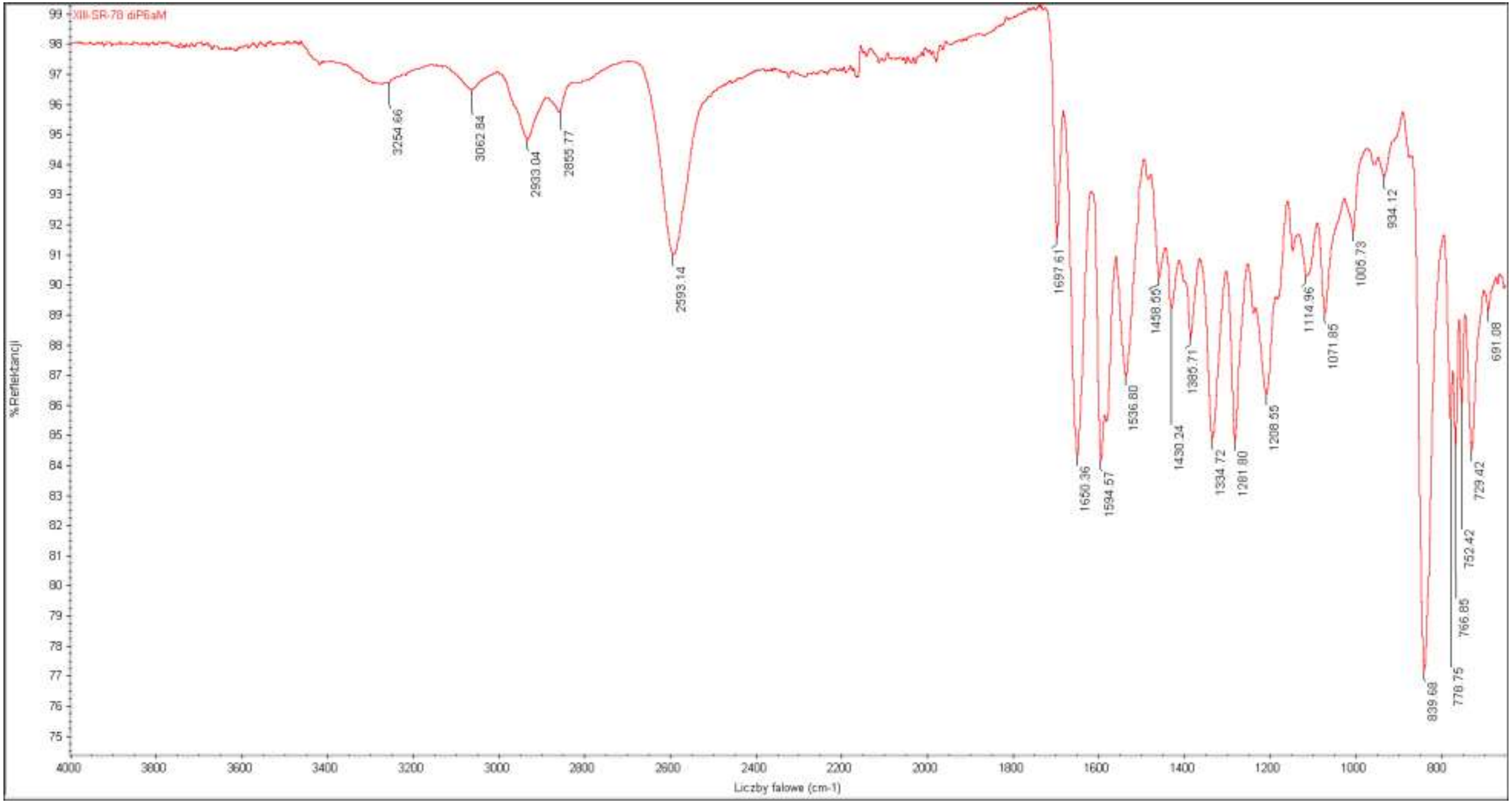

Figure S59. IR spectrum of **19**.

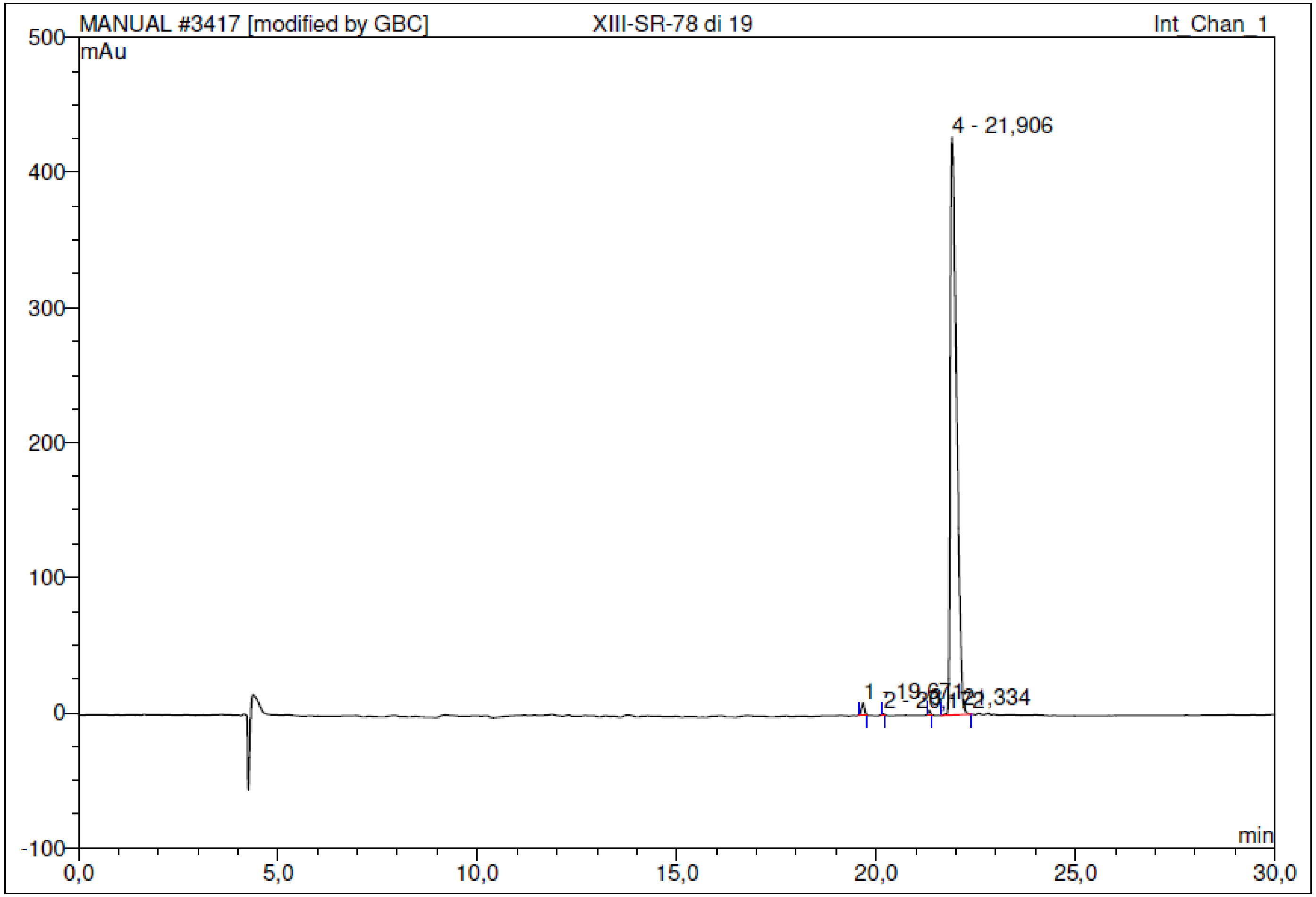

No.	Ret.Time min	Peak Name	Height mAu	Area mAu*min	Rel.Area %	Amount	Type
1	19,67	n.a.	9,031	0,850	1,08	n.a.	BMB*
2	20,17	n.a.	1,003	0,055	0,07	n.a.	BMB*
3	21,33	n.a.	2,851	0,187	0,24	n.a.	BMB*
4	21,91	n.a.	427,629	77,312	98,61	n.a.	BMB
Total:			440,514	78,404	100,00	0,000	

Figure S60. HPLC analysis of **19**.

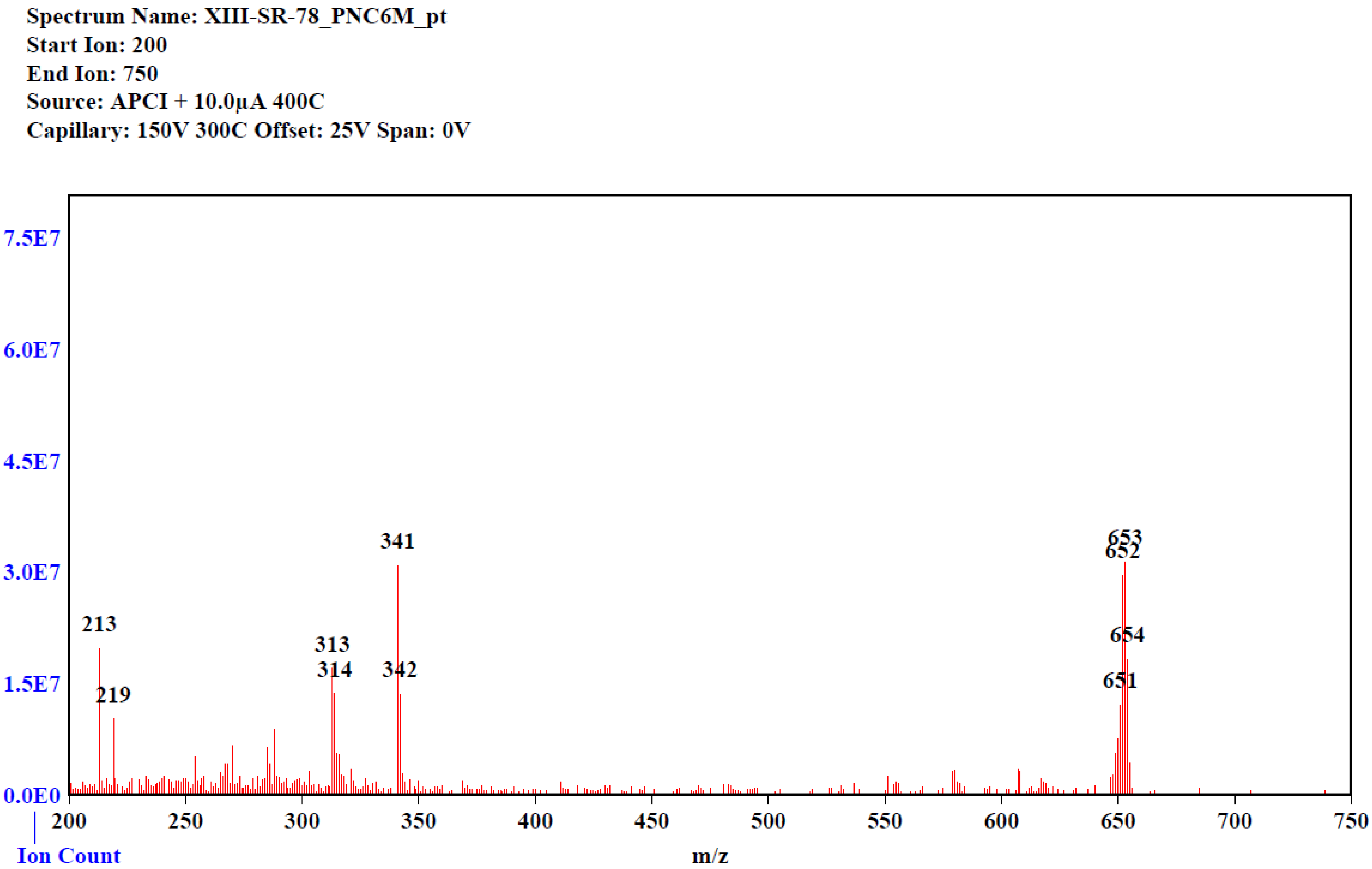

Figure S61. MS spectrum of **19**.

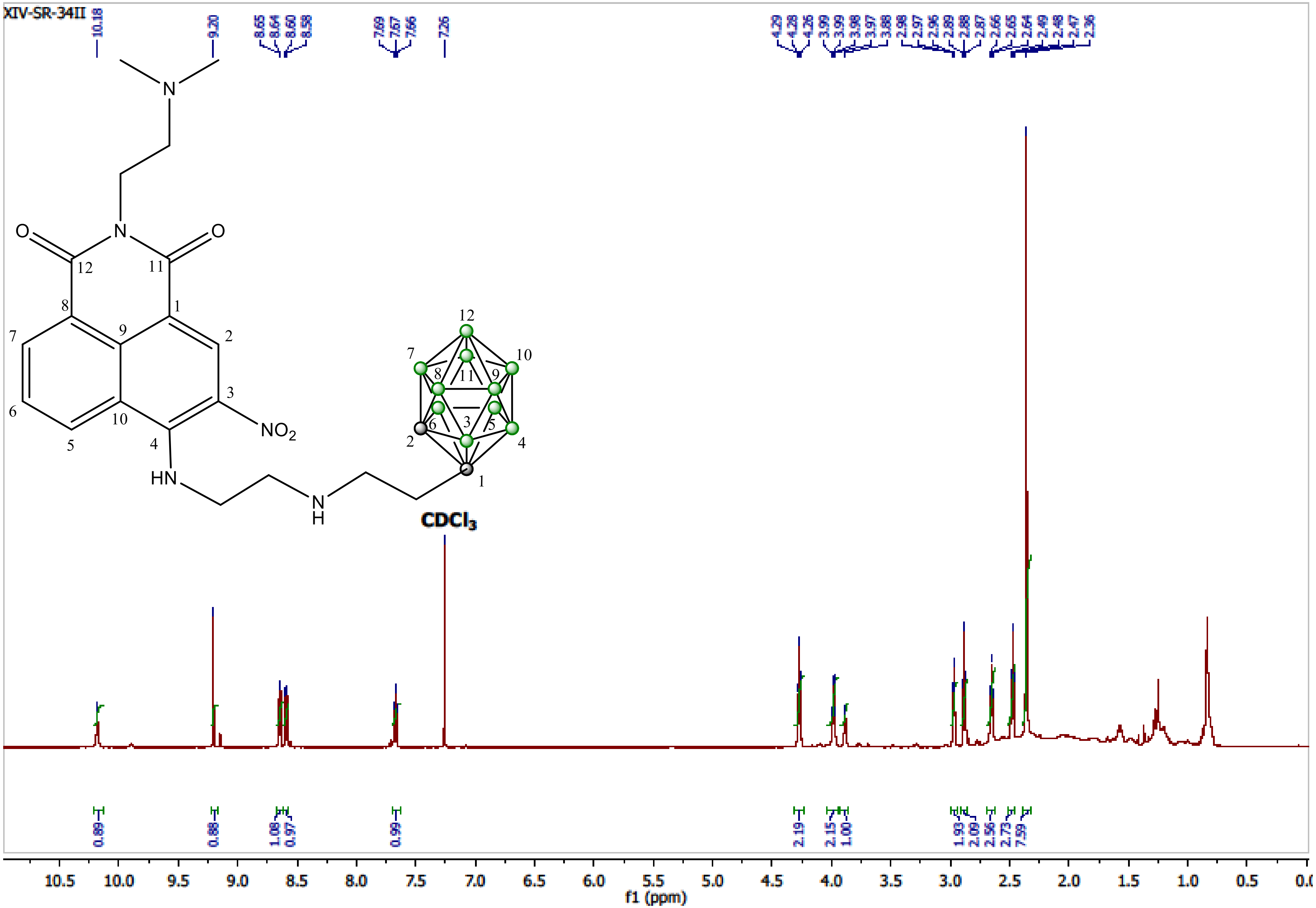

Figure S62. ^{1}H-NMR spectrum of **30**.

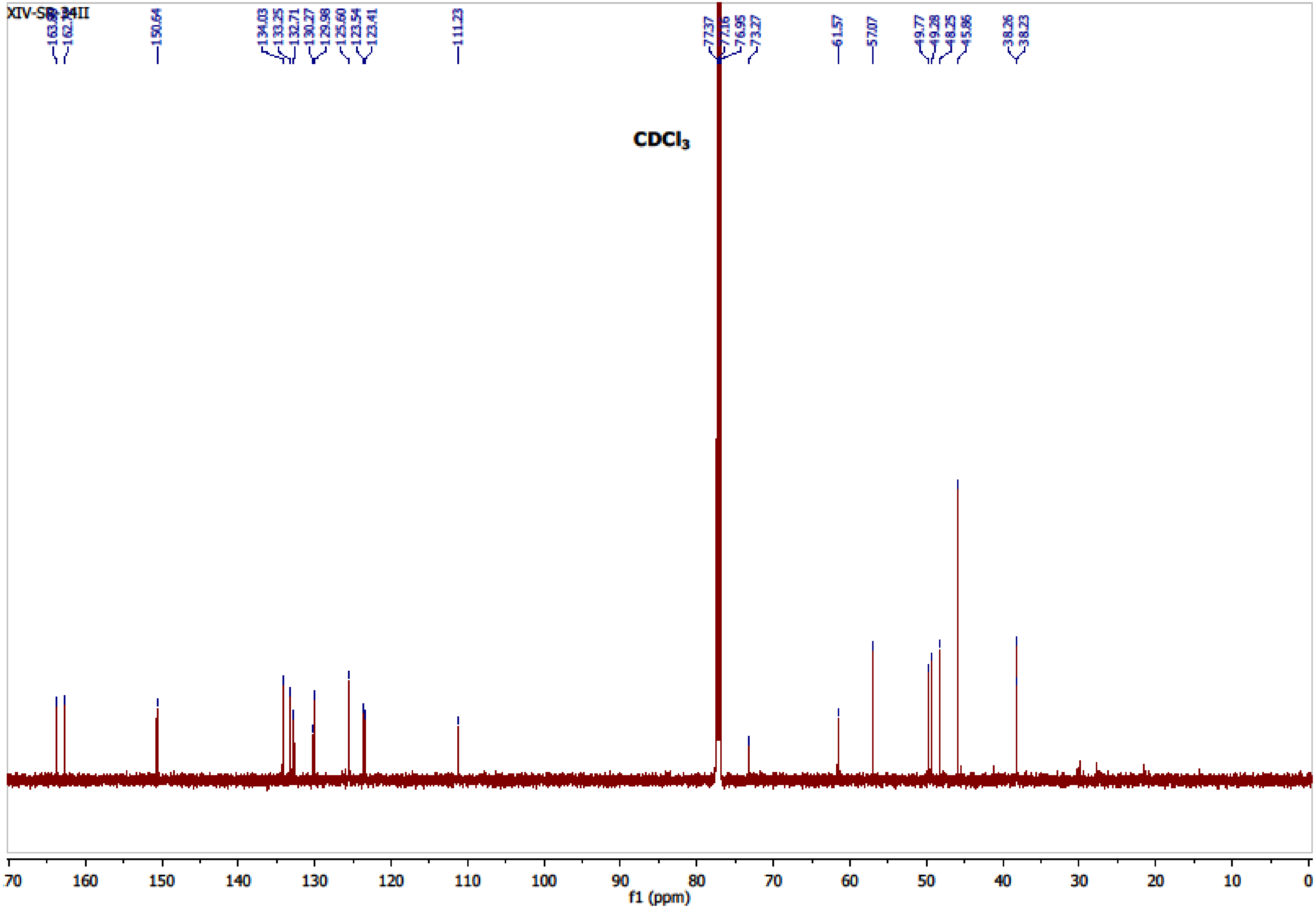

Figure S63. ^{13}C-NMR spectrum of **30**.

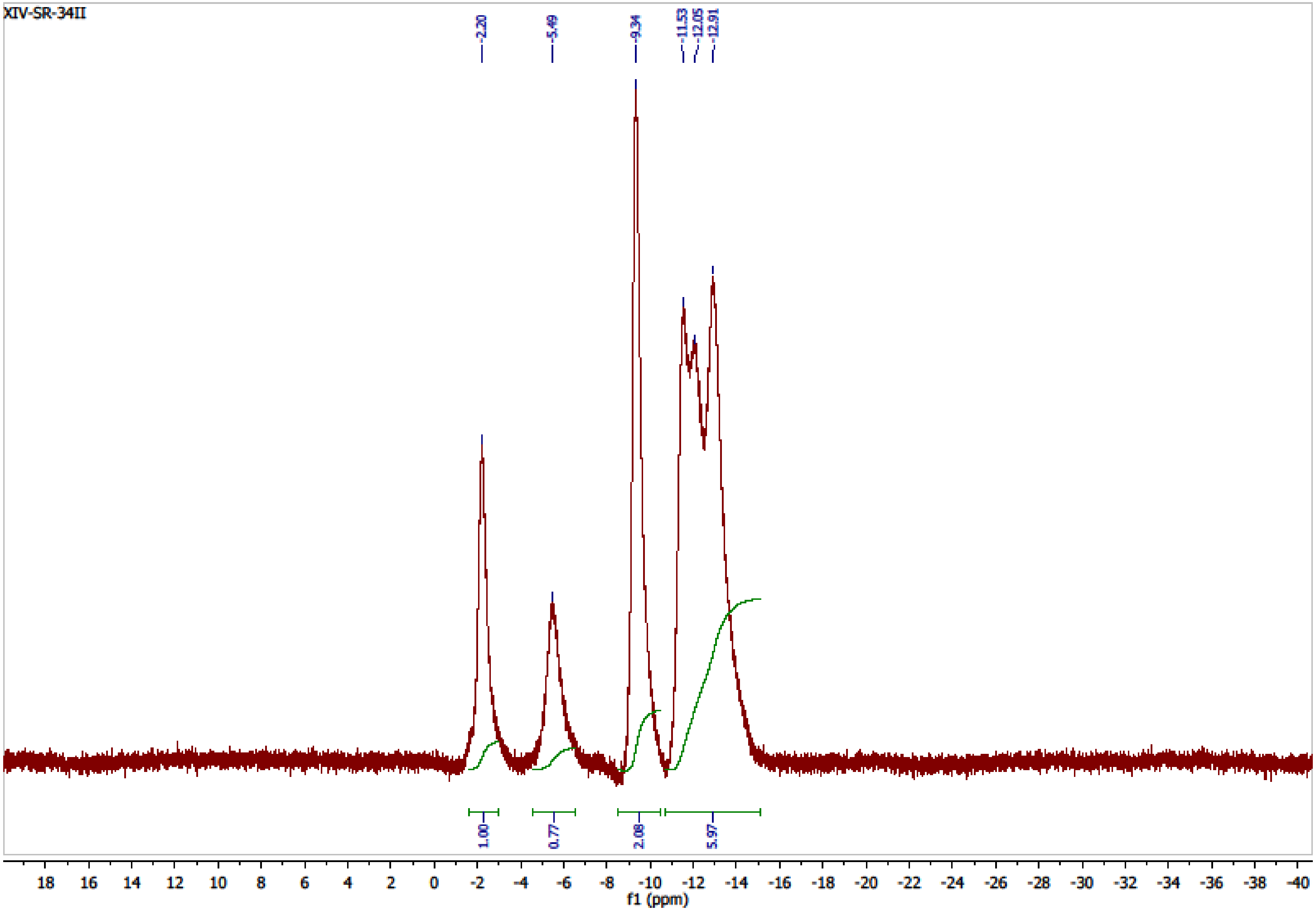

Figure S64. ^{11}B-NMR {^{1}H BB} spectrum of **30**.

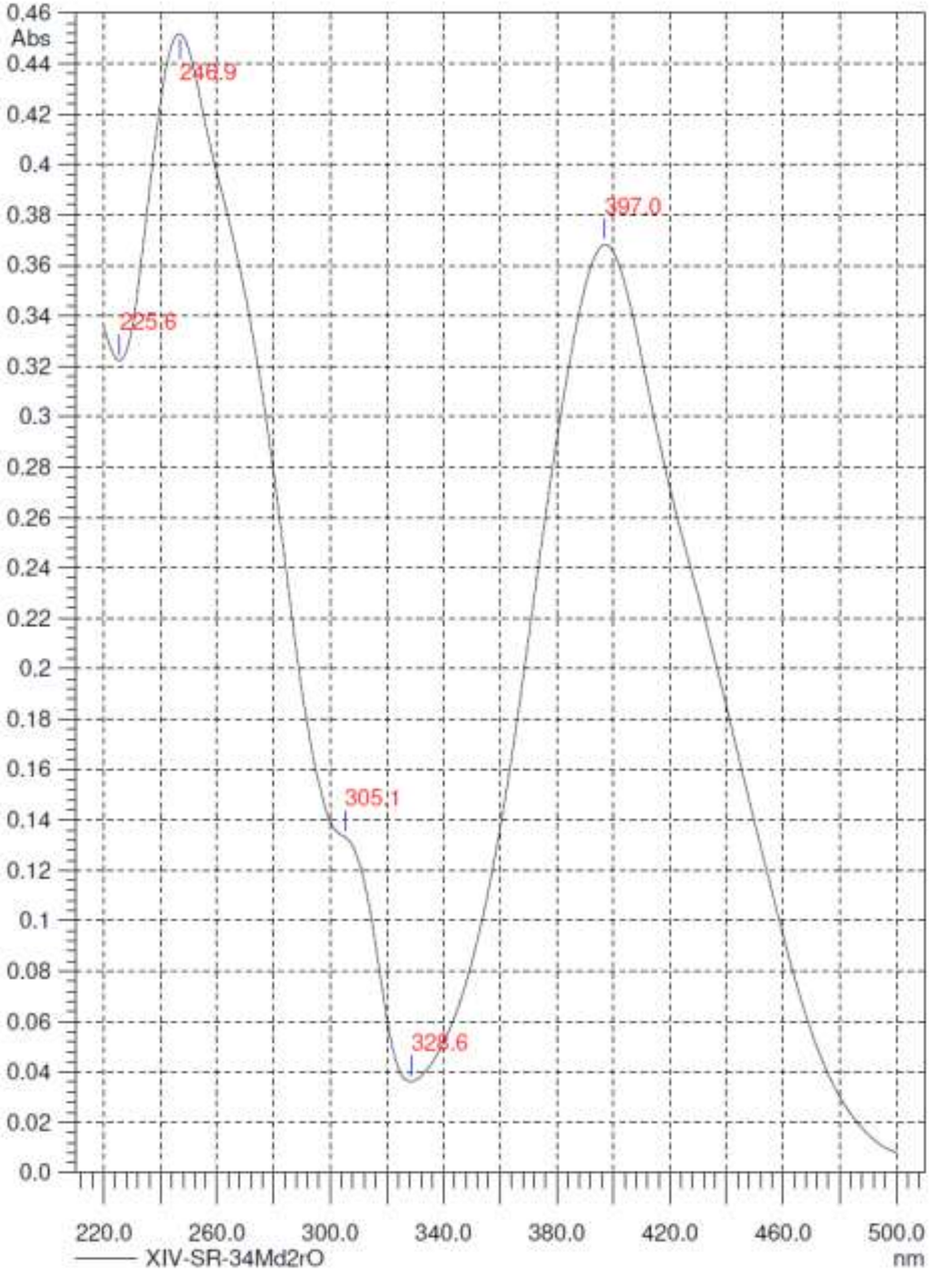

Figure S65. UV spectrum of **30**.

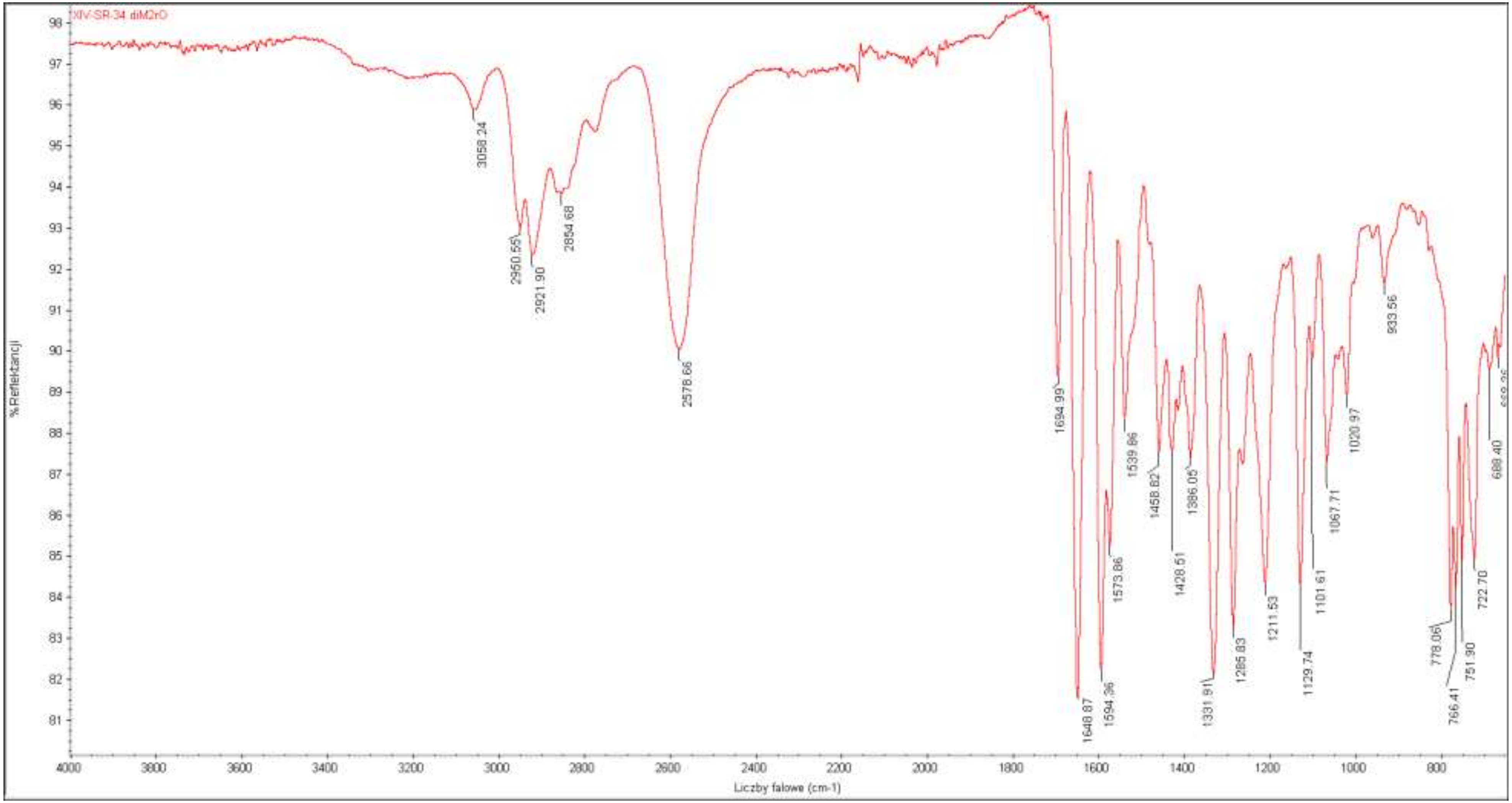

Figure S66. IR spectrum of **30**.

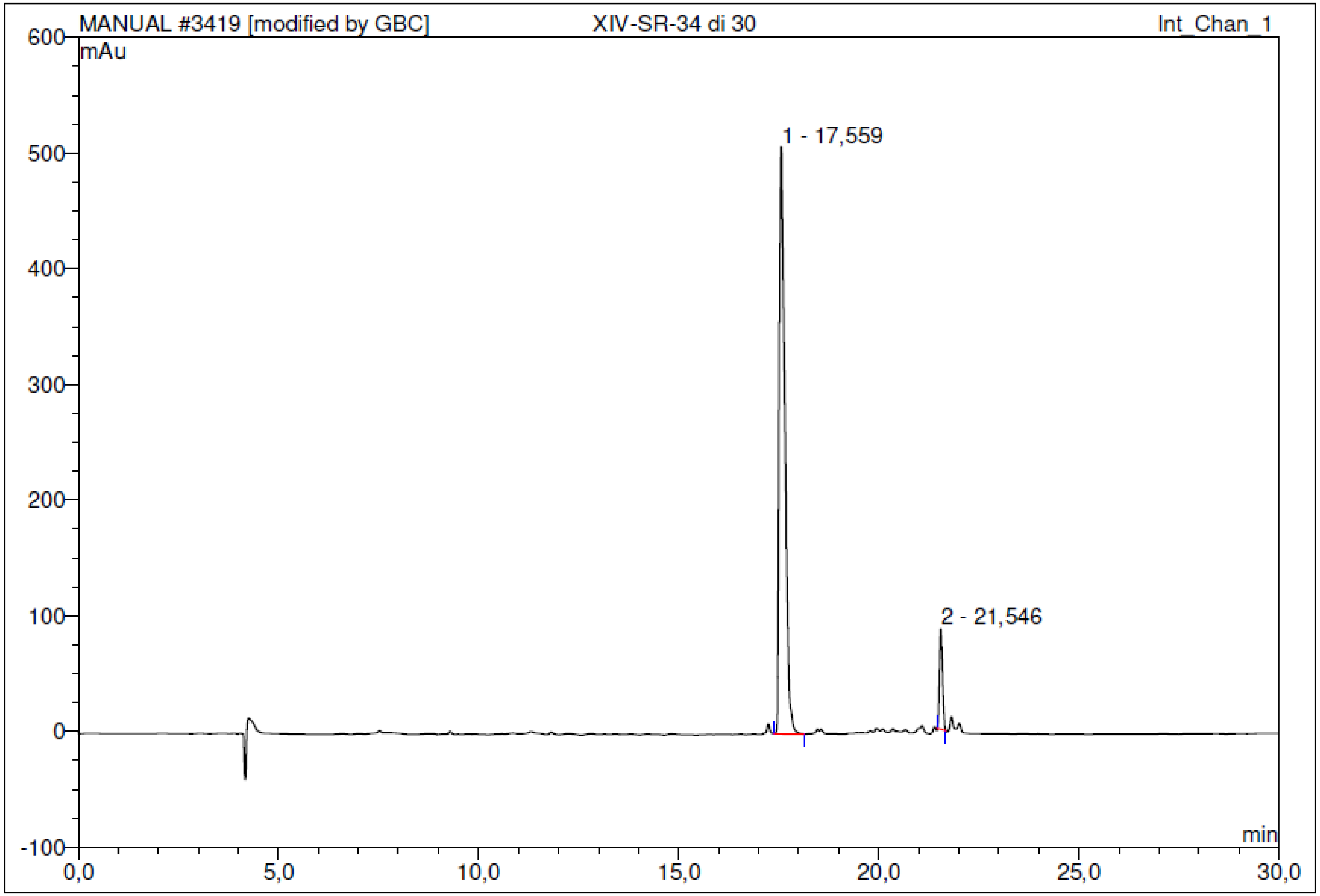

No.	Ret.Time min	Peak Name	Height mAu	Area mAu*min	Rel.Area %	Amount	Type
1	17,56	n.a.	507,426	81,171	91,44	n.a.	BMB
2	21,55	n.a.	86,468	7,601	8,56	n.a.	BMB*
Total:			593,893	88,772	100,00	0,000	

Figure S67. HPLC analysis of **30**.

Spectrum Name: XIV-SR-34_pt
Start Ion: 200
End Ion: 600
Source: APCI + 10.0µA 400C
Capillary: 150V 300C Offset: 25V Span: 0V

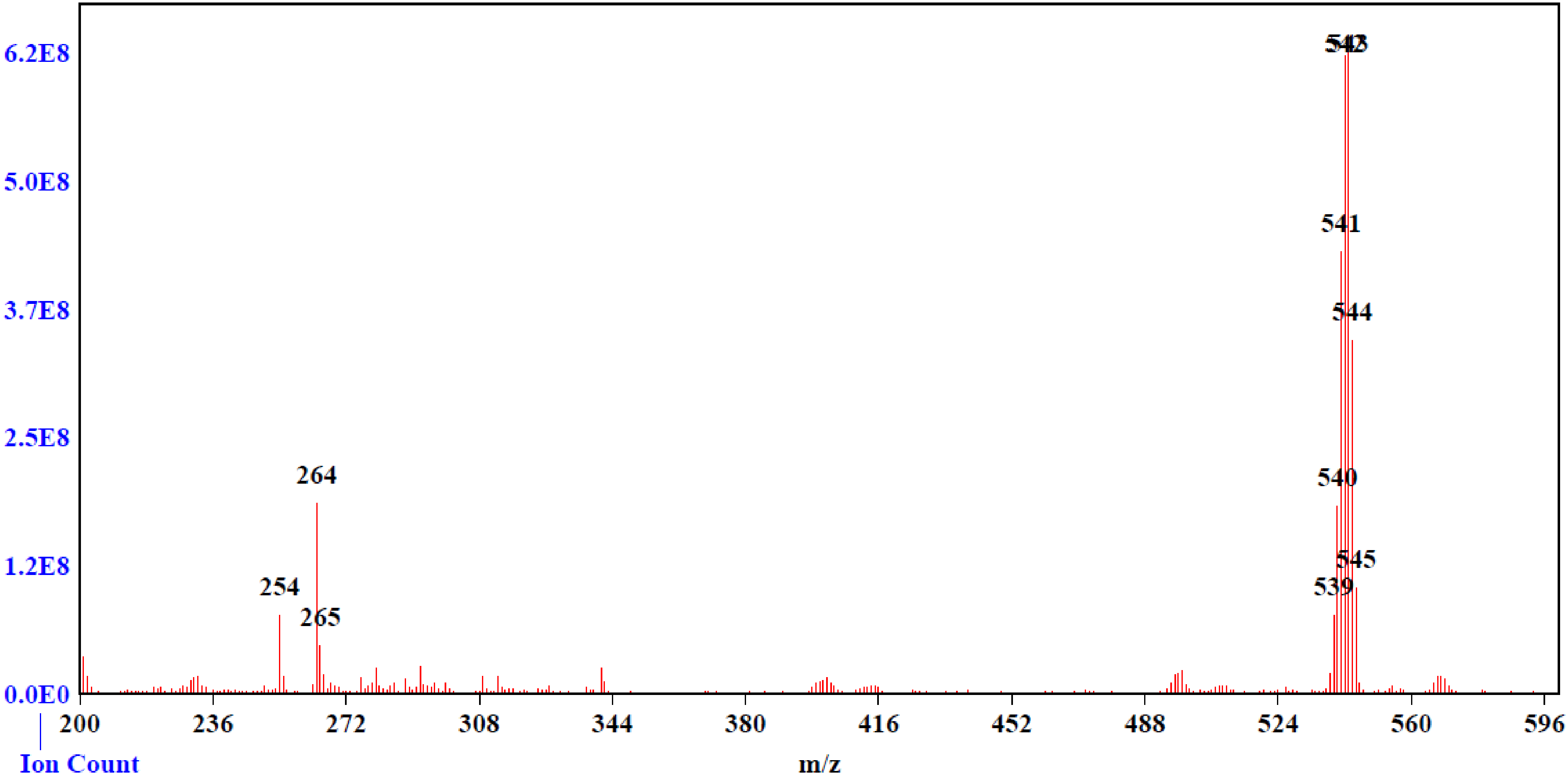

Figure S68. MS spectrum of **30**.

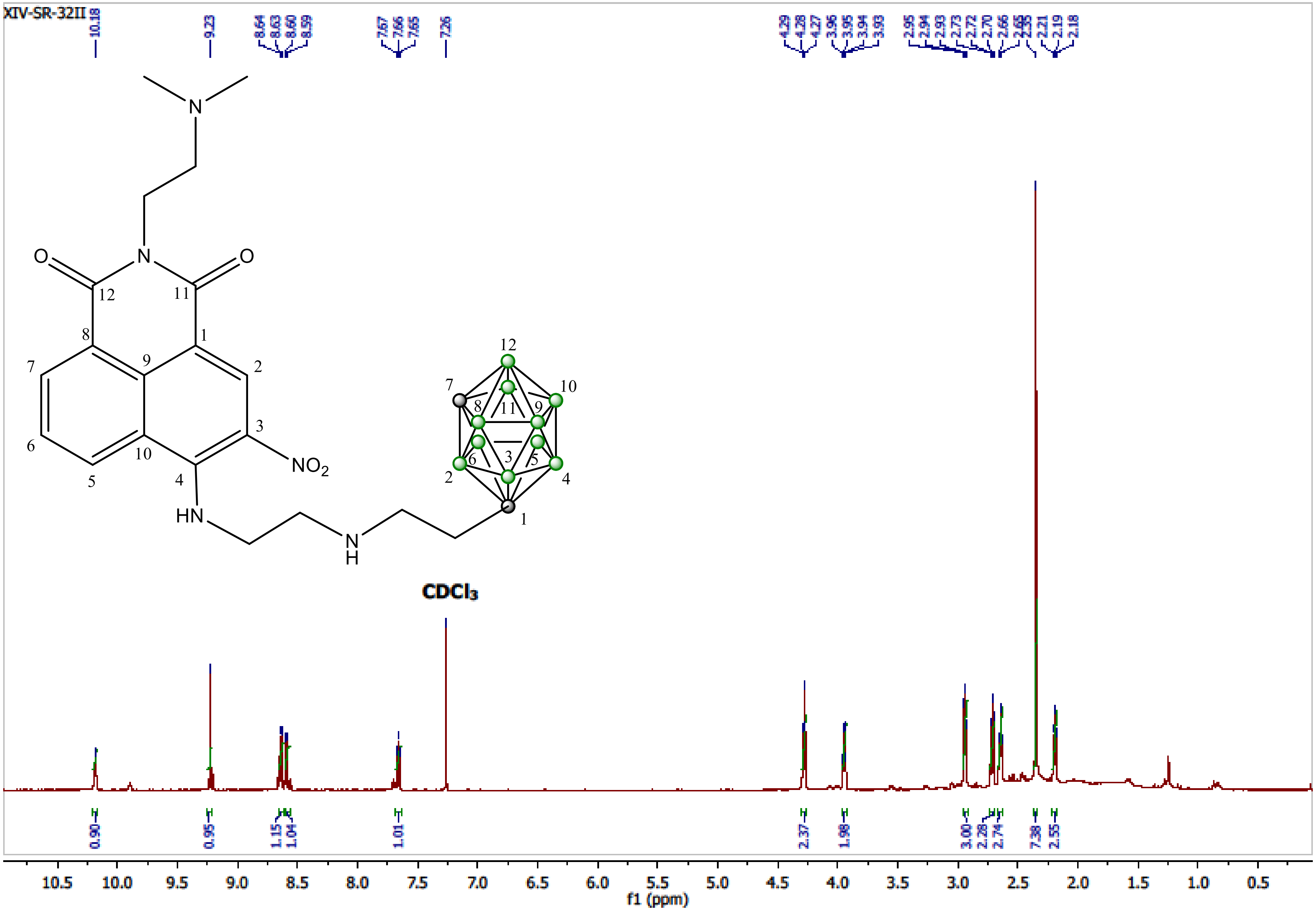

Figure S69. ^{1}H-NMR spectrum of **31**.

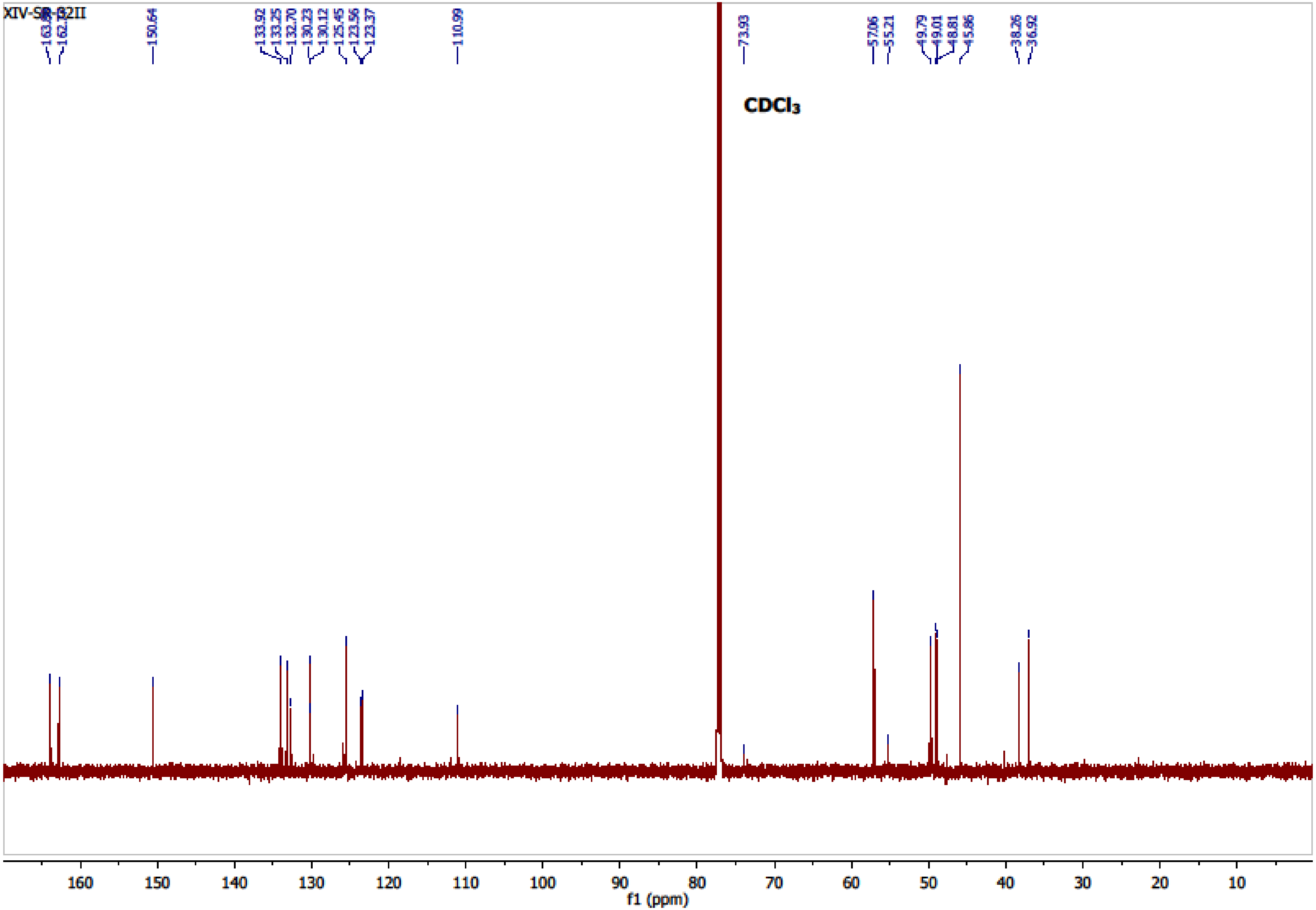

Figure S70. ^{13}C-NMR spectrum of **31**.

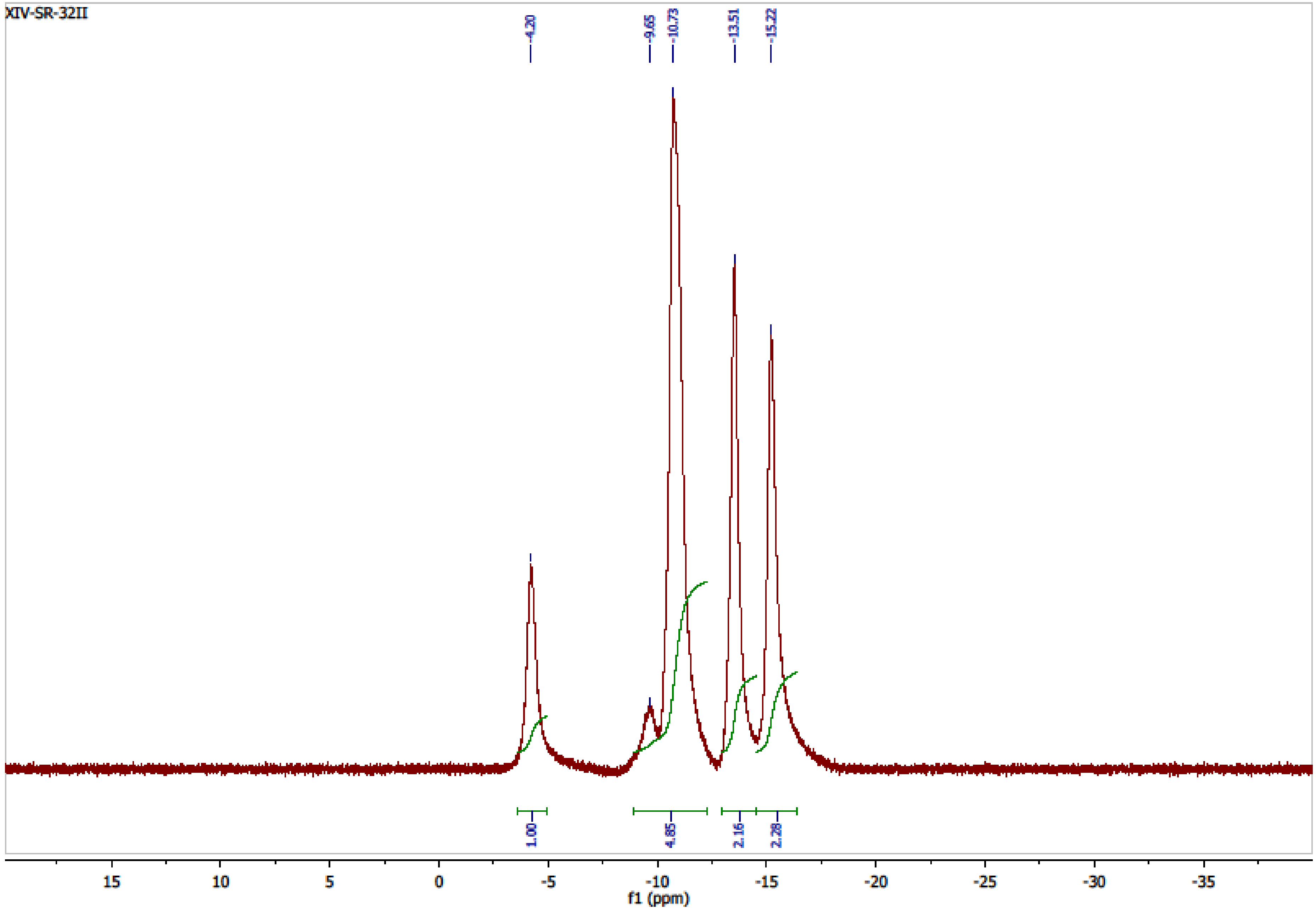

Figure S71. ^{11}B-NMR {^{1}H BB} spectrum of **31**.

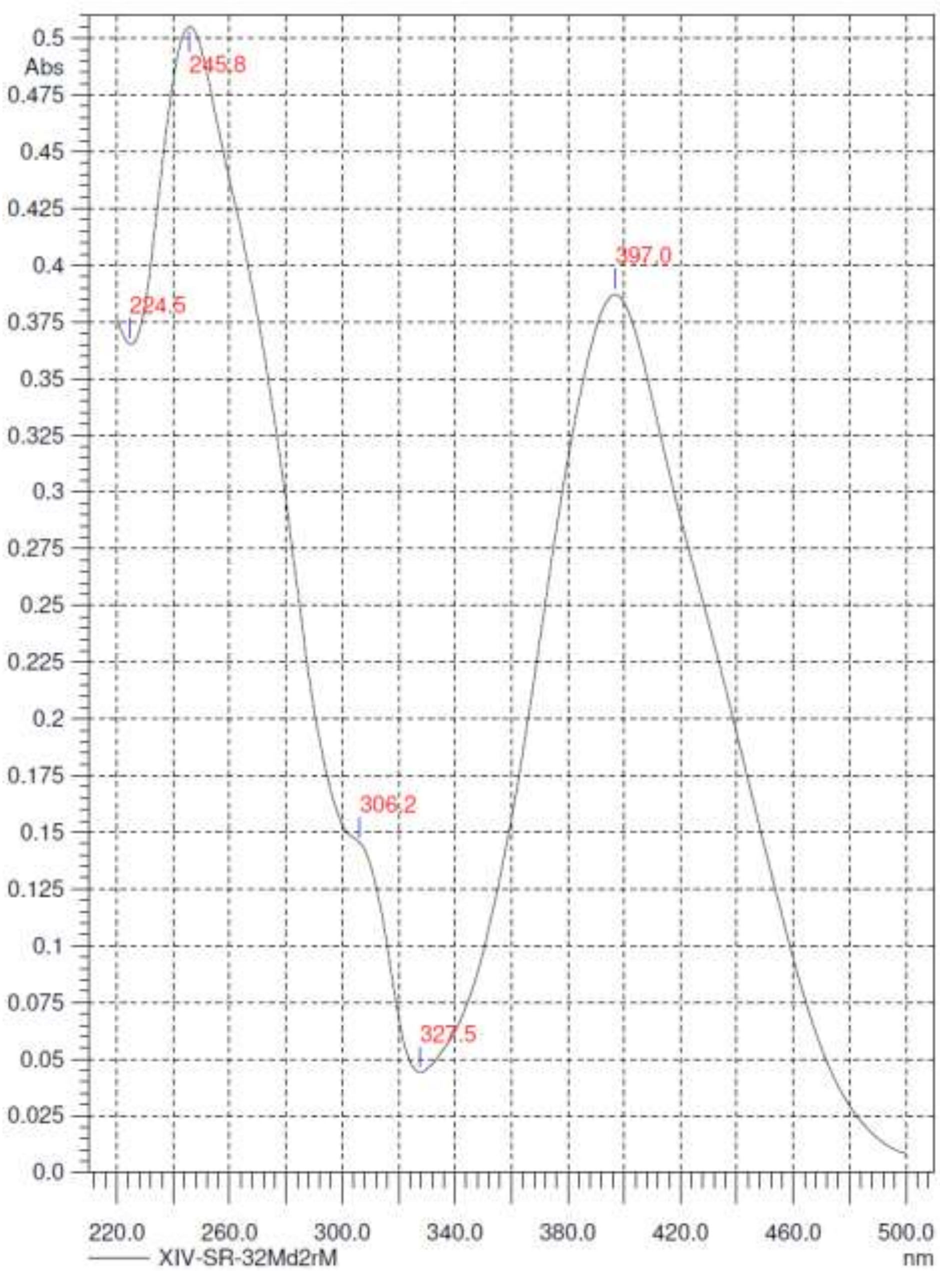

Figure S72. UV spectrum of **31**.

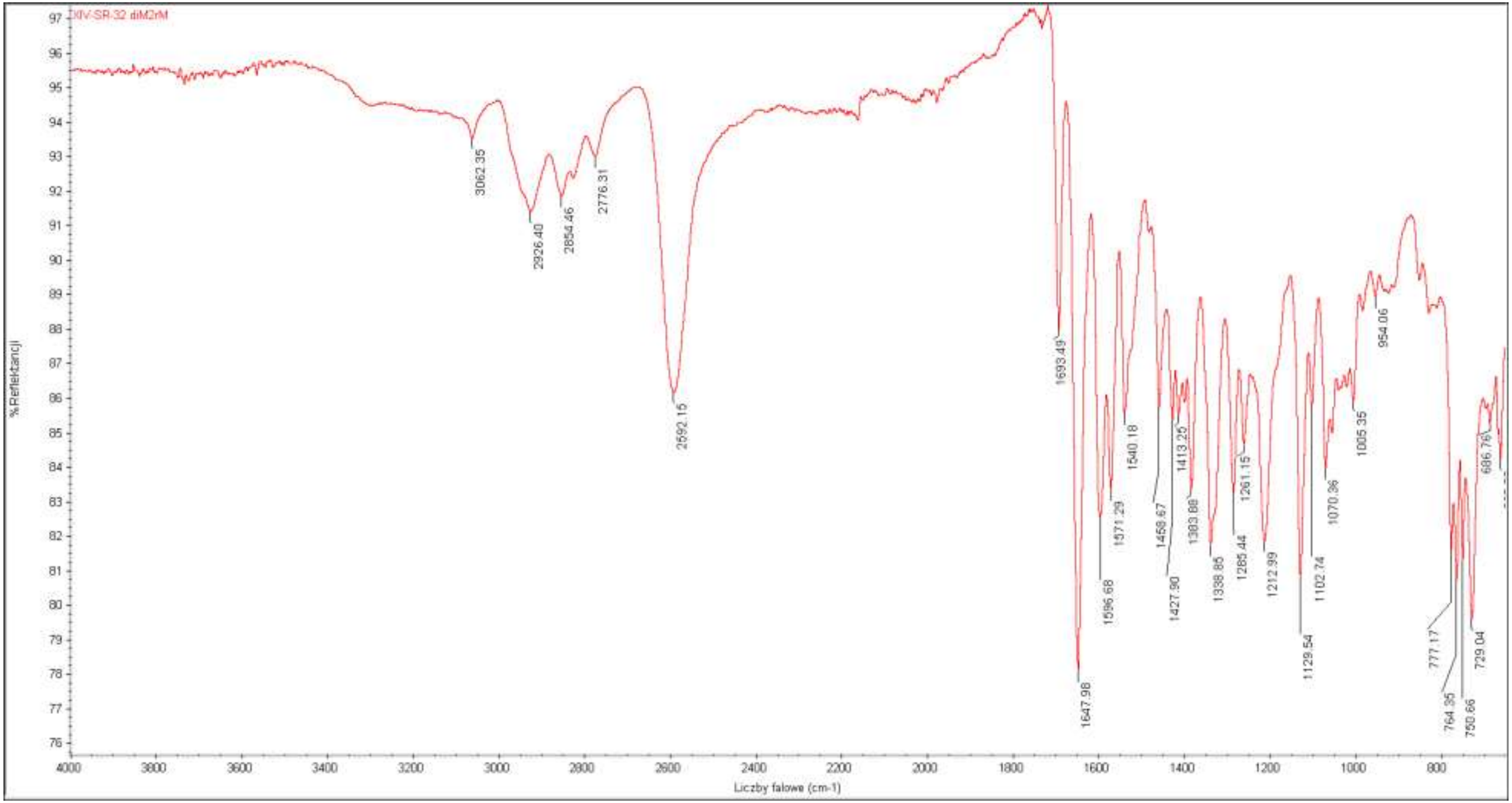

Figure S73. IR spectrum of **31**.

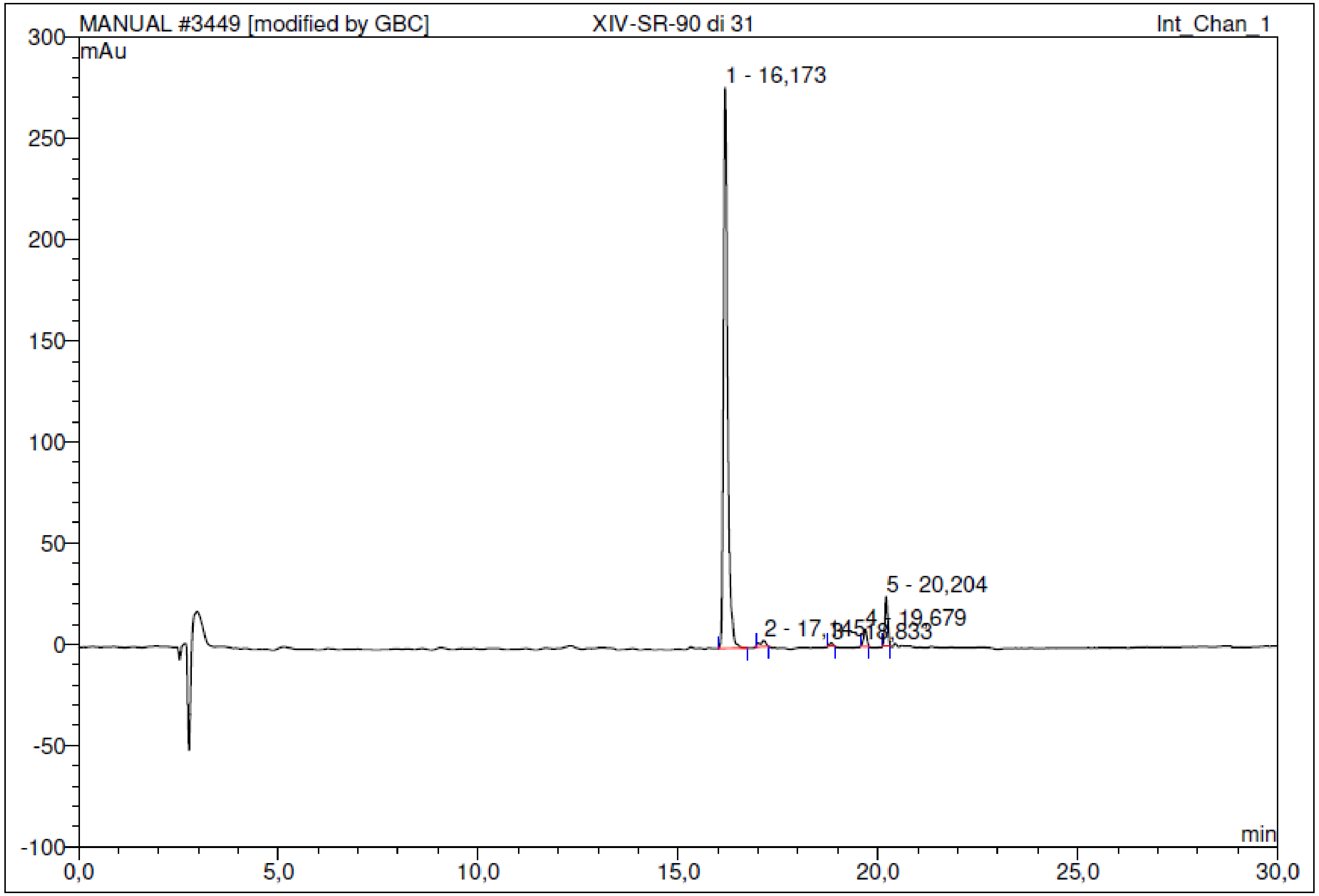

No.	Ret.Time min	Peak Name	Height mAu	Area mAu*min	Rel.Area %	Amount	Type
1	16,17	n.a.	277,329	34,692	90,84	n.a.	BMB
2	17,14	n.a.	3,004	0,509	1,33	n.a.	BMB*
3	18,83	n.a.	1,855	0,176	0,46	n.a.	BMB*
4	19,68	n.a.	8,468	0,770	2,02	n.a.	BMB*
5	20,20	n.a.	24,522	2,042	5,35	n.a.	BMB*
Total:			315,180	38,188	100,00	0,000	

Figure S74. HPLC analysis of **31**.

Spectrum Name: XIV-SR-32_pt
Start Ion: 100
End Ion: 700
Source: APCI + 10.0µA 400C
Capillary: 150V 300C Offset: 25V Span: 0V

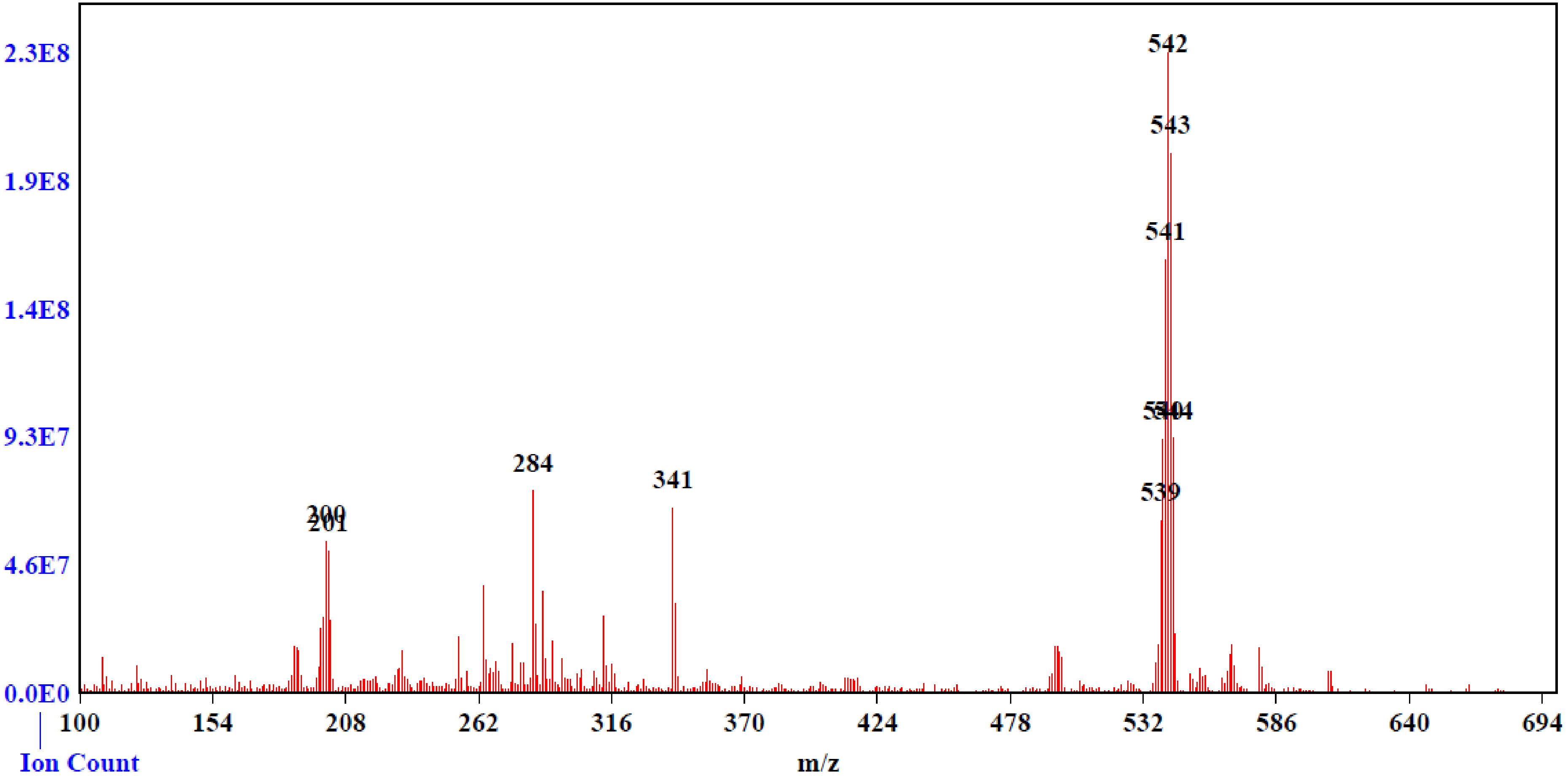

Figure S75. MS spectrum of **31**.

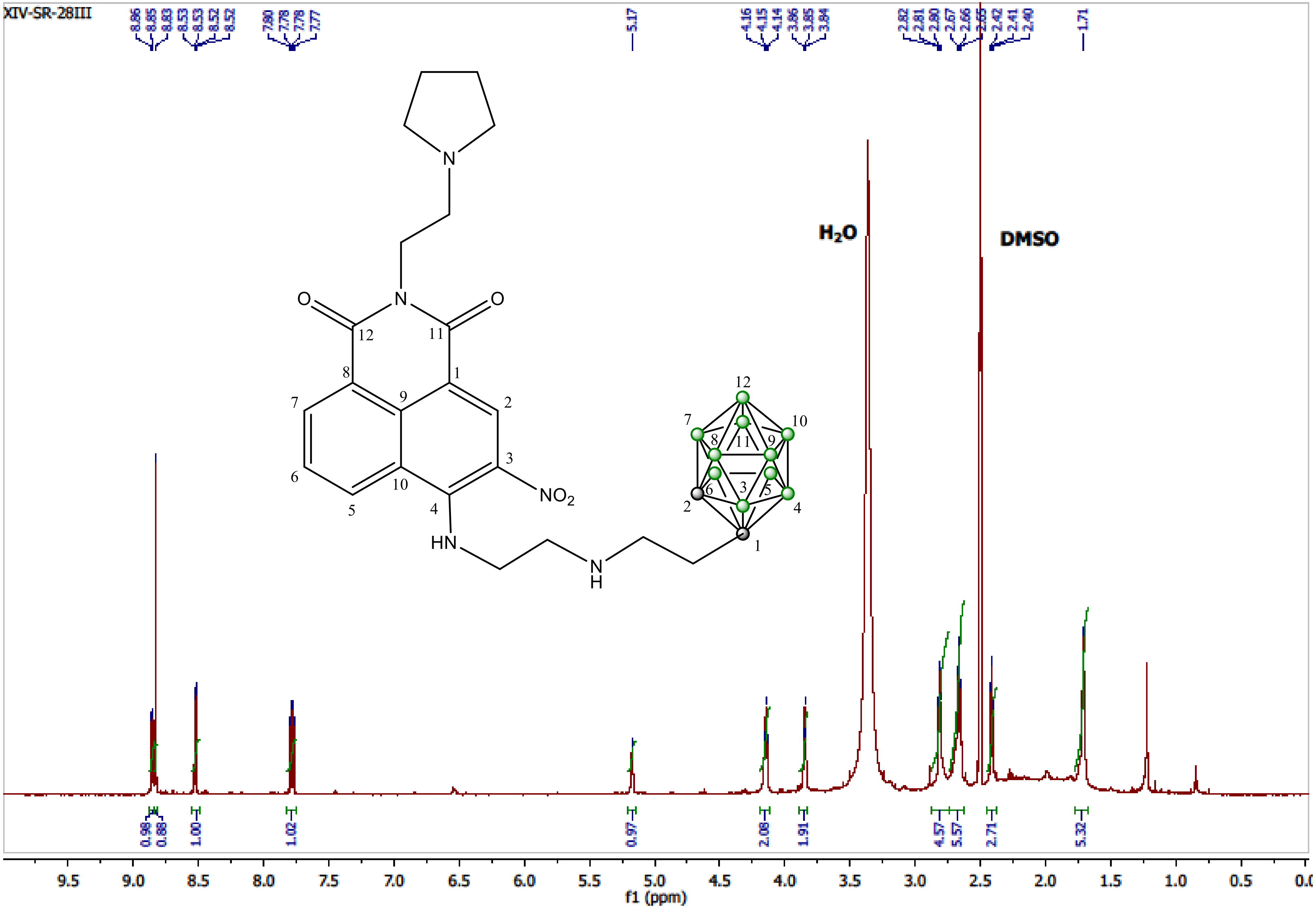

Figure S76. ^{1}H-NMR spectrum of **32**.

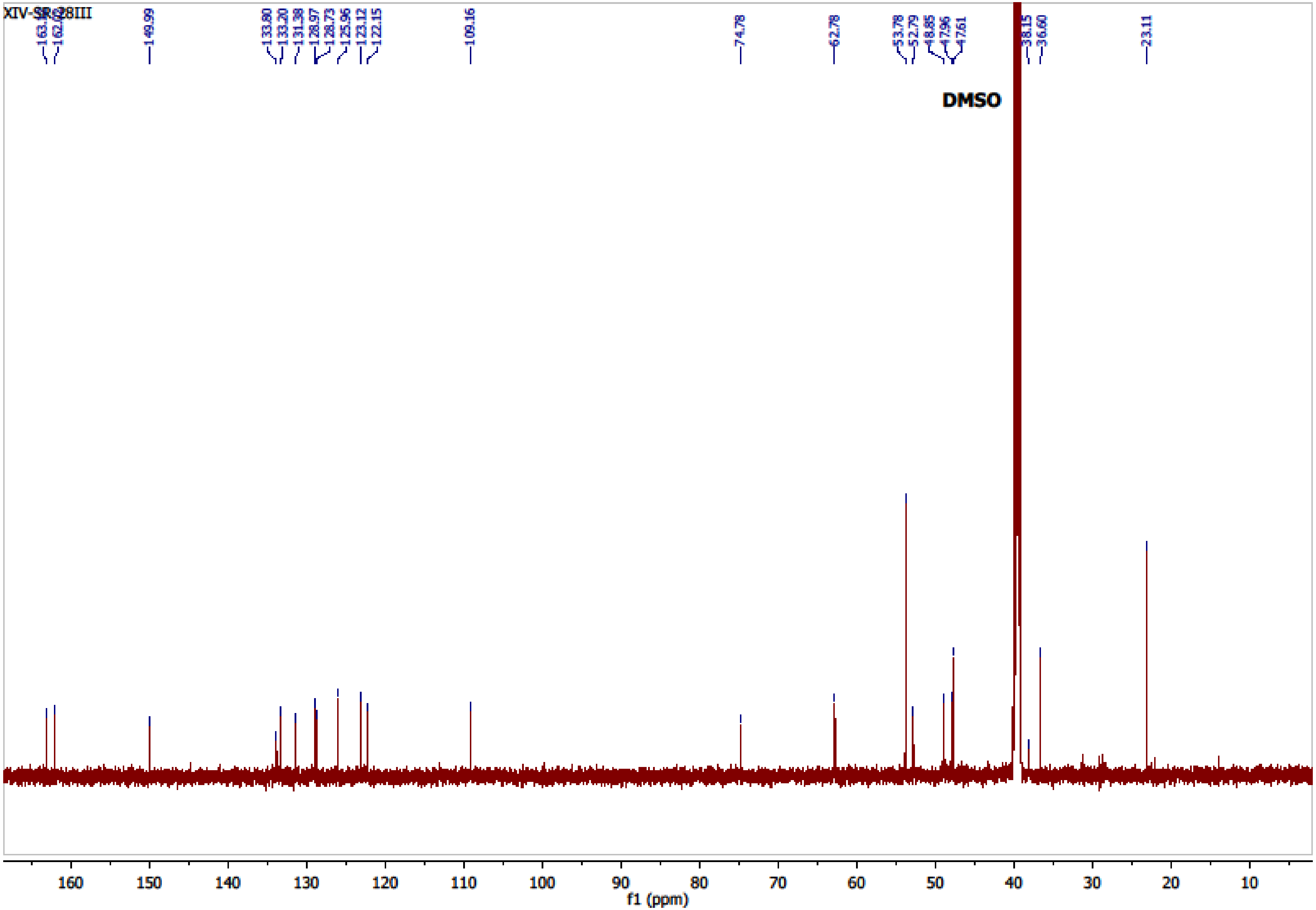

Figure S77. ^{13}C-NMR spectrum of **32**.

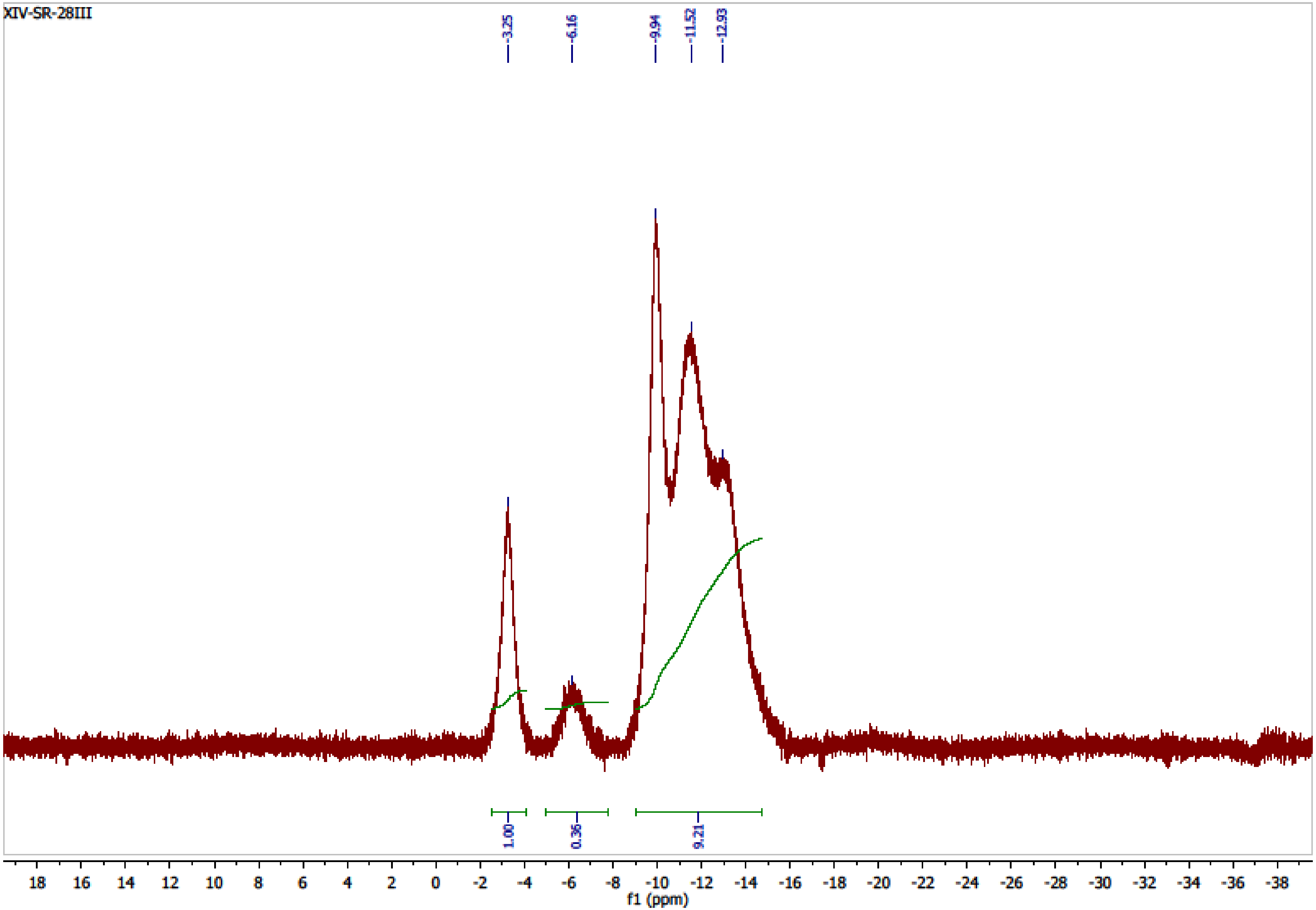

Figure S78. ^{11}B-NMR {^{1}H BB} spectrum of **32**.

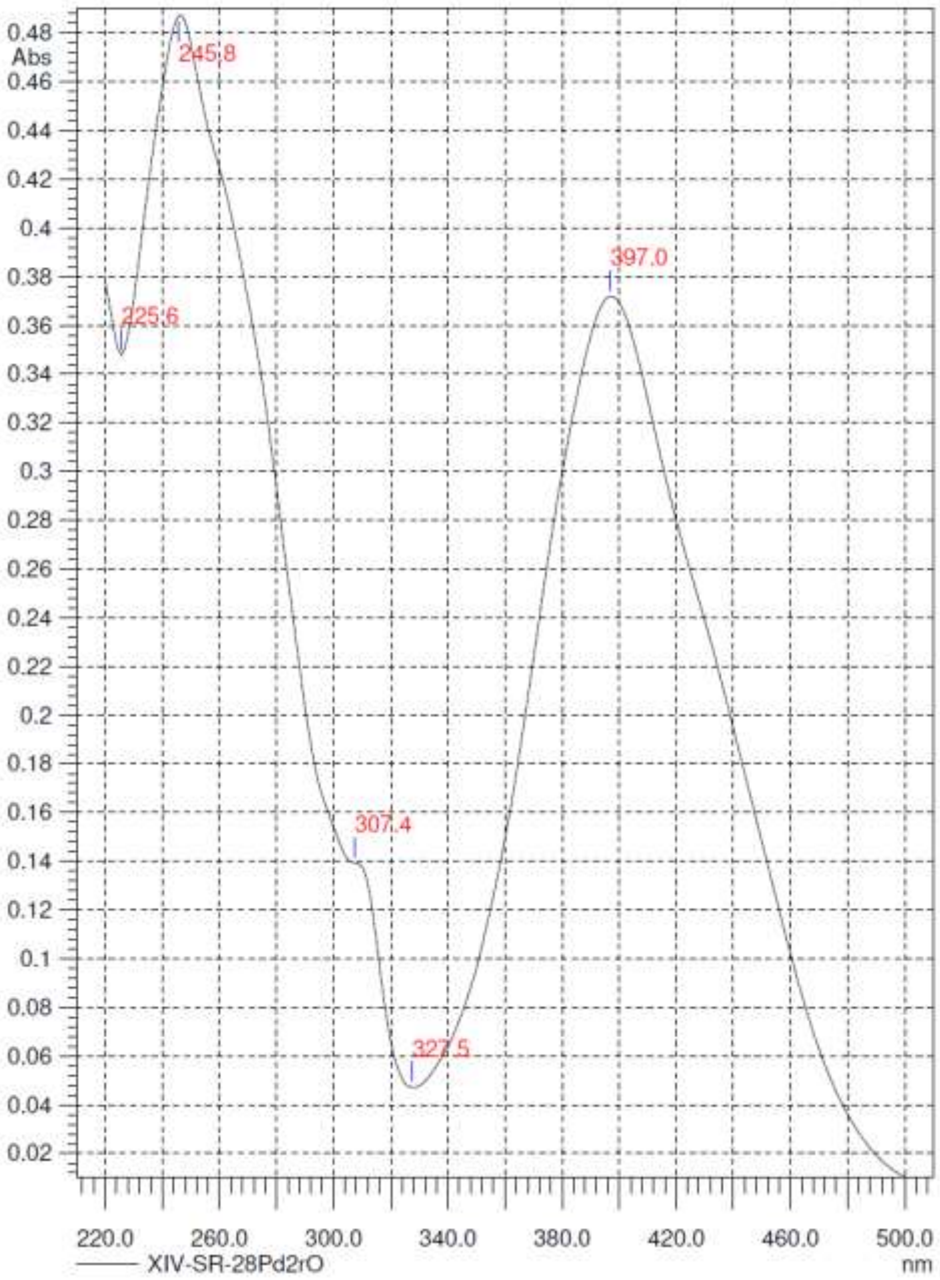

Figure S79. UV spectrum of **32**.

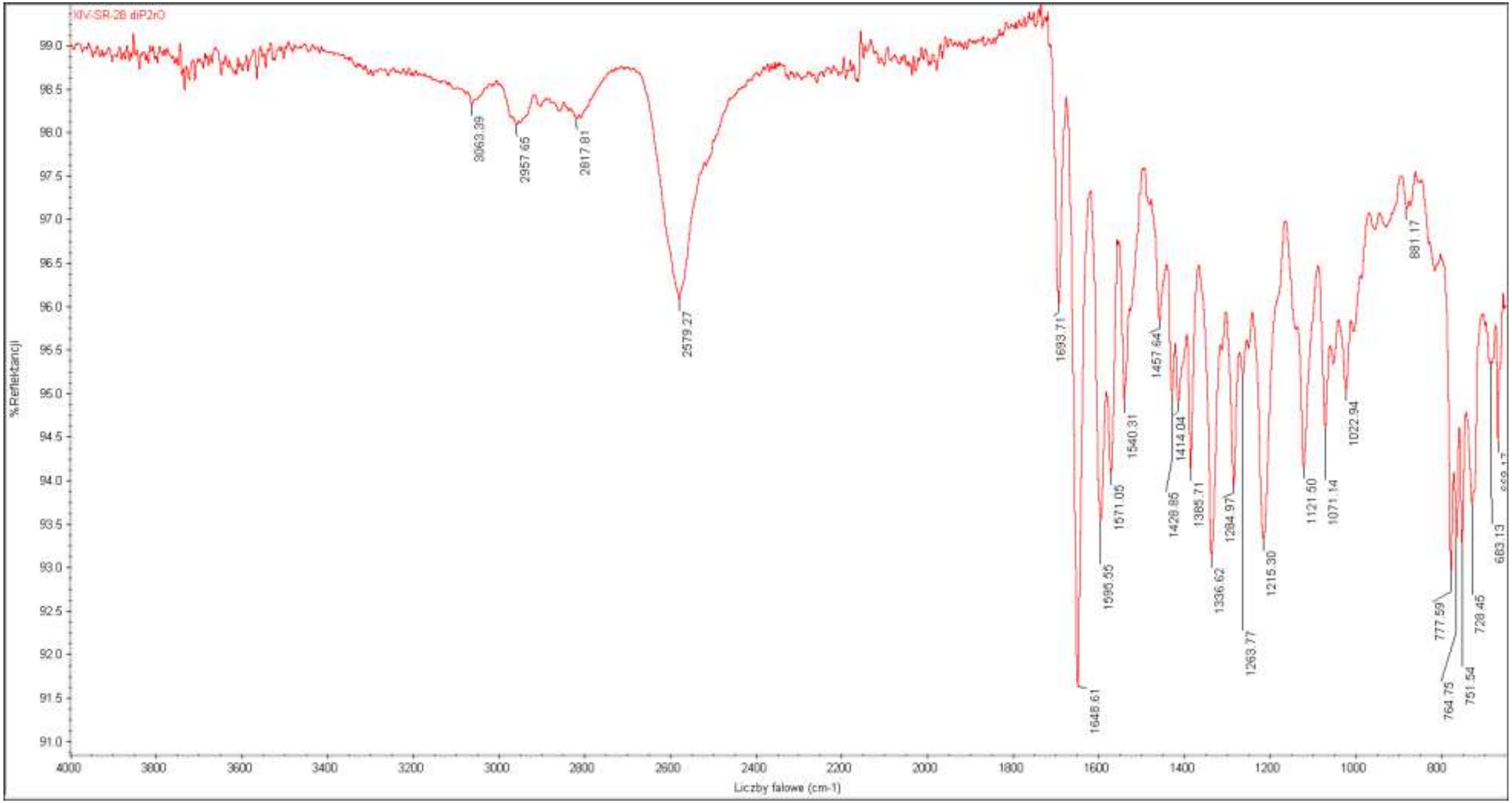

Figure S80. IR spectrum of **32**.

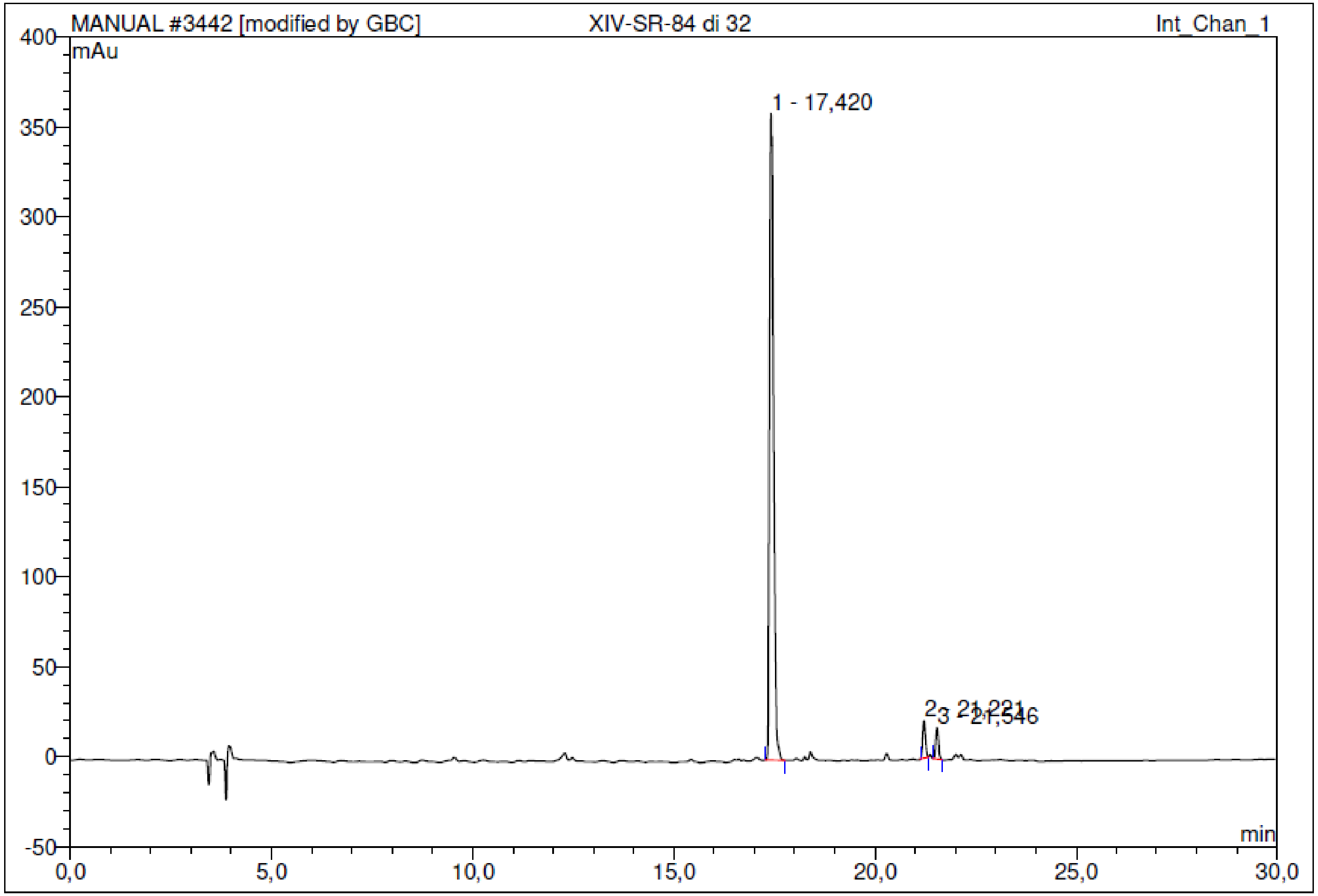

No.	Ret.Time min	Peak Name	Height mAu	Area mAu*min	Rel.Area %	Amount	Type
1	17,42	n.a.	359,402	41,299	93,03	n.a.	BMB
2	21,22	n.a.	20,728	1,673	3,77	n.a.	BMB*
3	21,55	n.a.	17,335	1,422	3,20	n.a.	BMB*
Total:			397,465	44,394	100,00	0,000	

Figure S81. HPLC analysis of **32**.

Spectrum Name: XIV-SR-84_32_pt
Start Ion: 100
End Ion: 1000
Source: APCI + 10.0µA 400C
Capillary: 150V 300C Offset: 25V Span: 0V

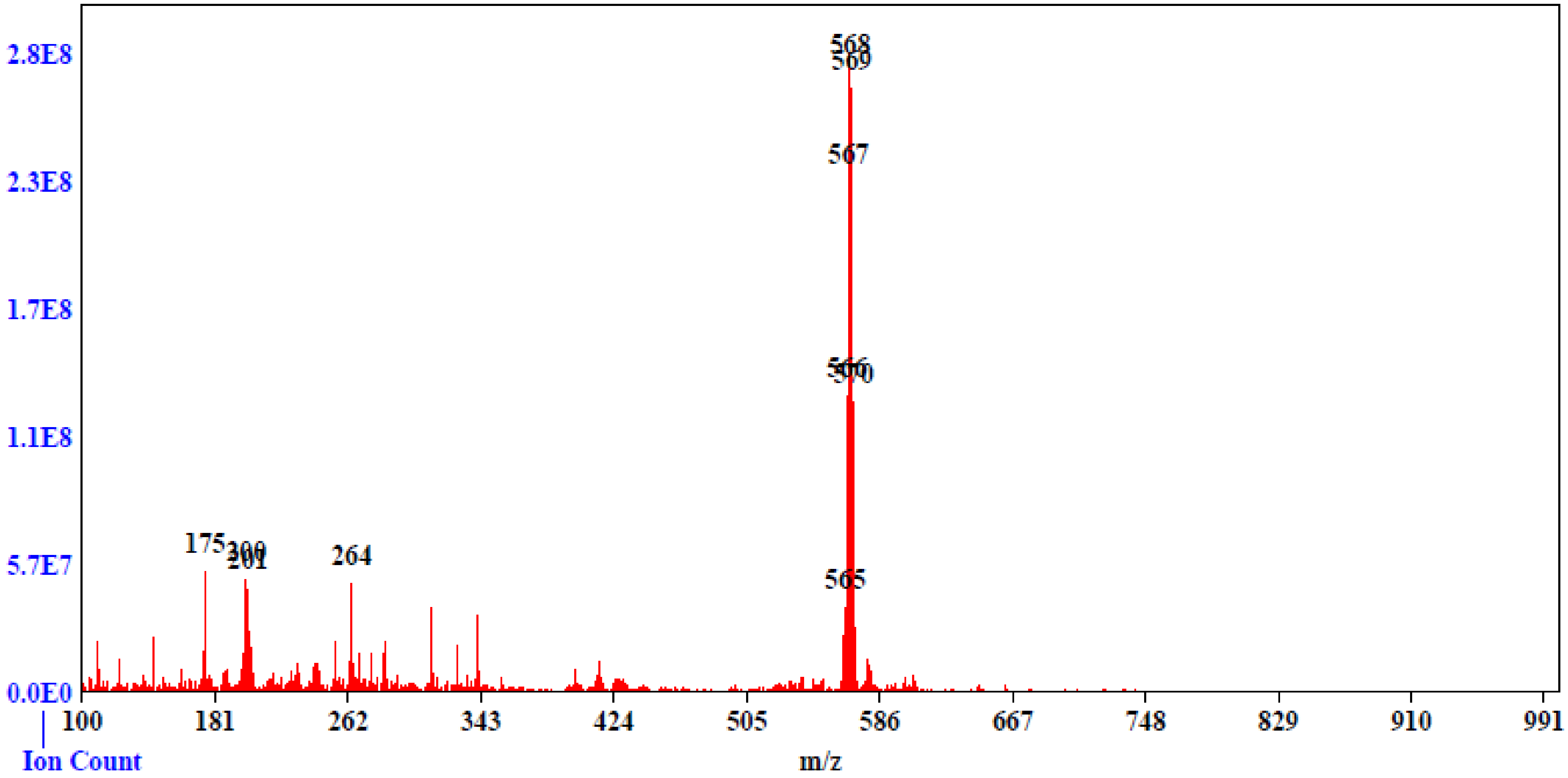

Figure S82. MS spectrum of **32**.

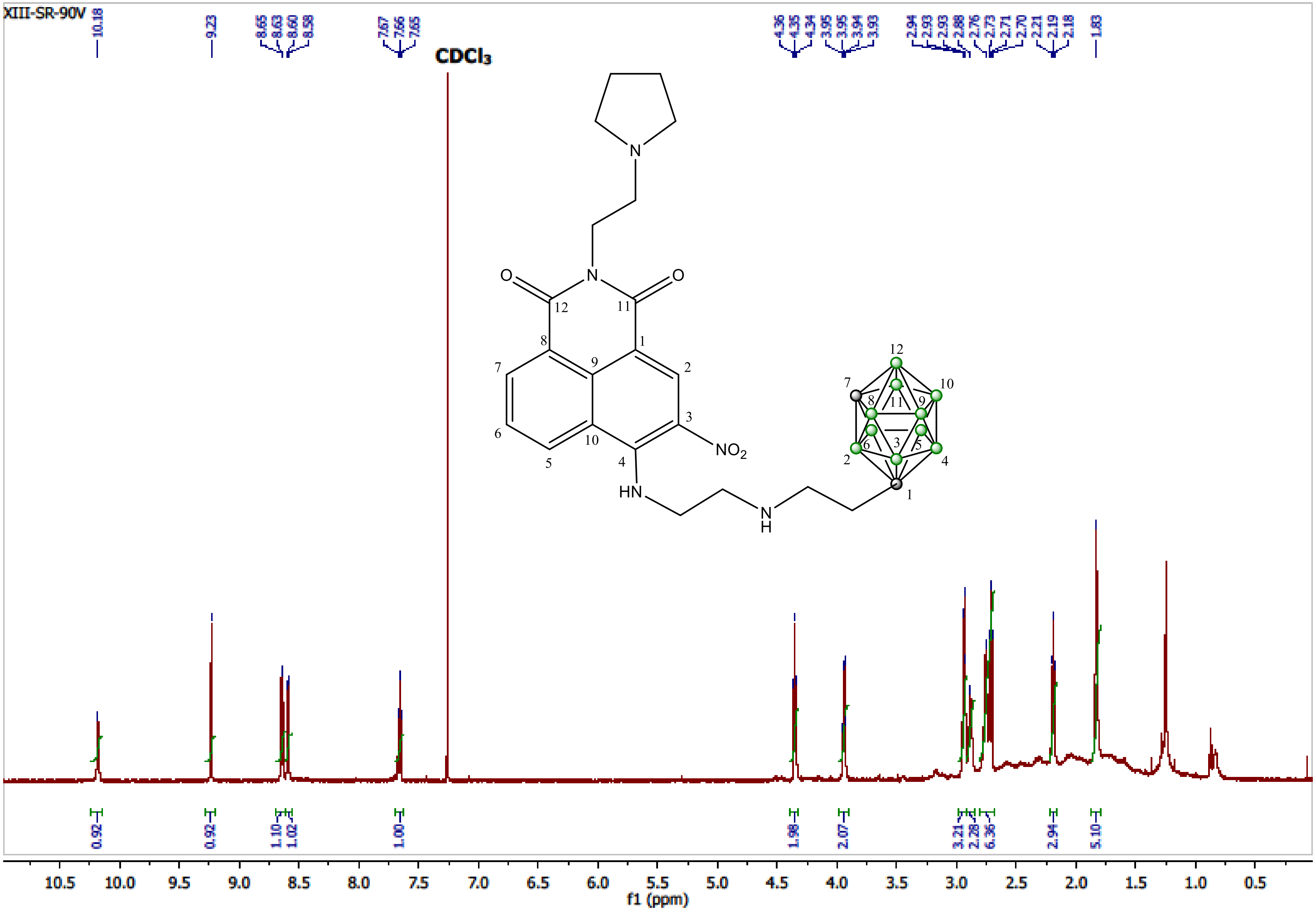

Figure S83. ^{1}H-NMR spectrum of **33**.

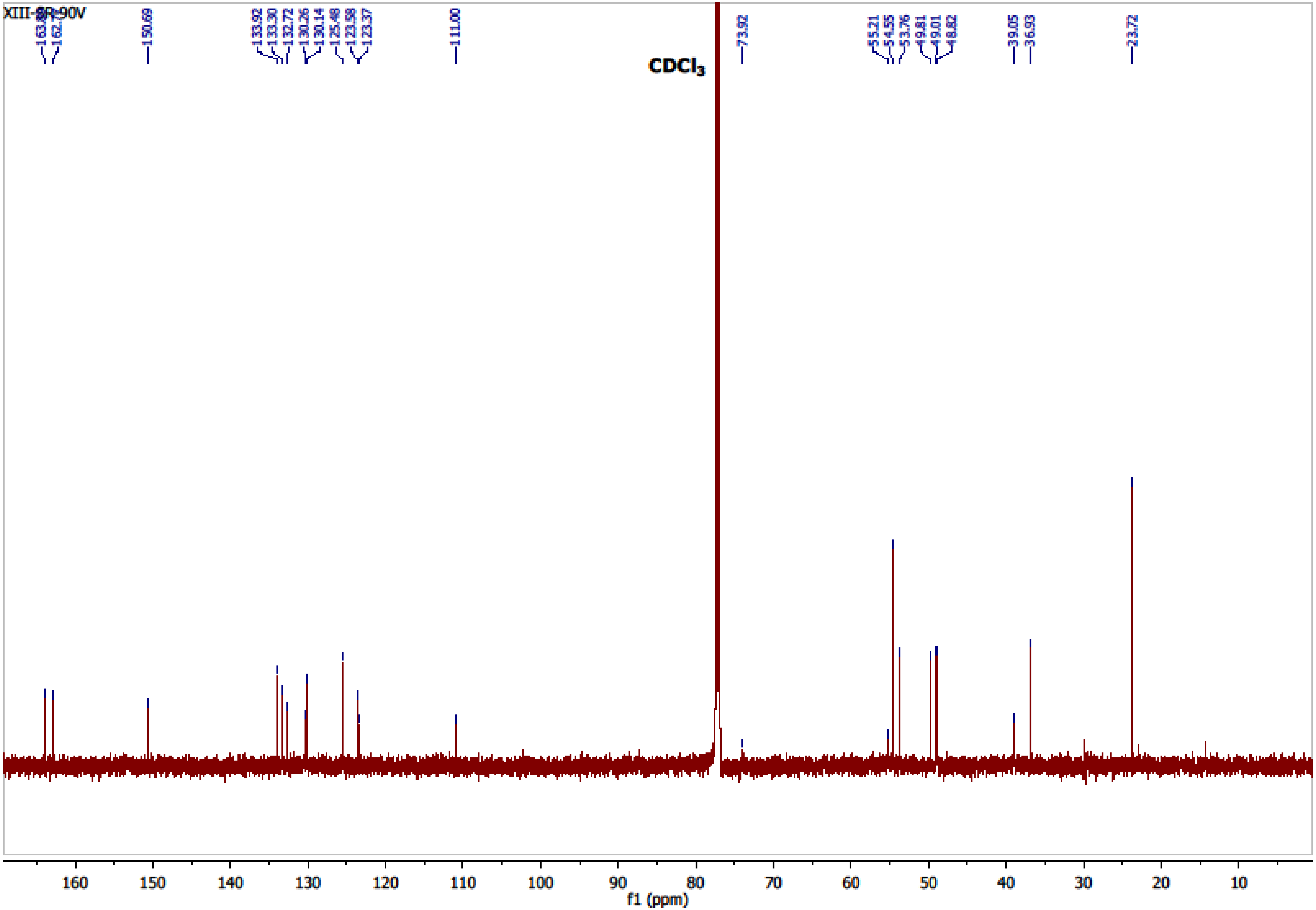

Figure S84. ^{13}C-NMR spectrum of **33**.

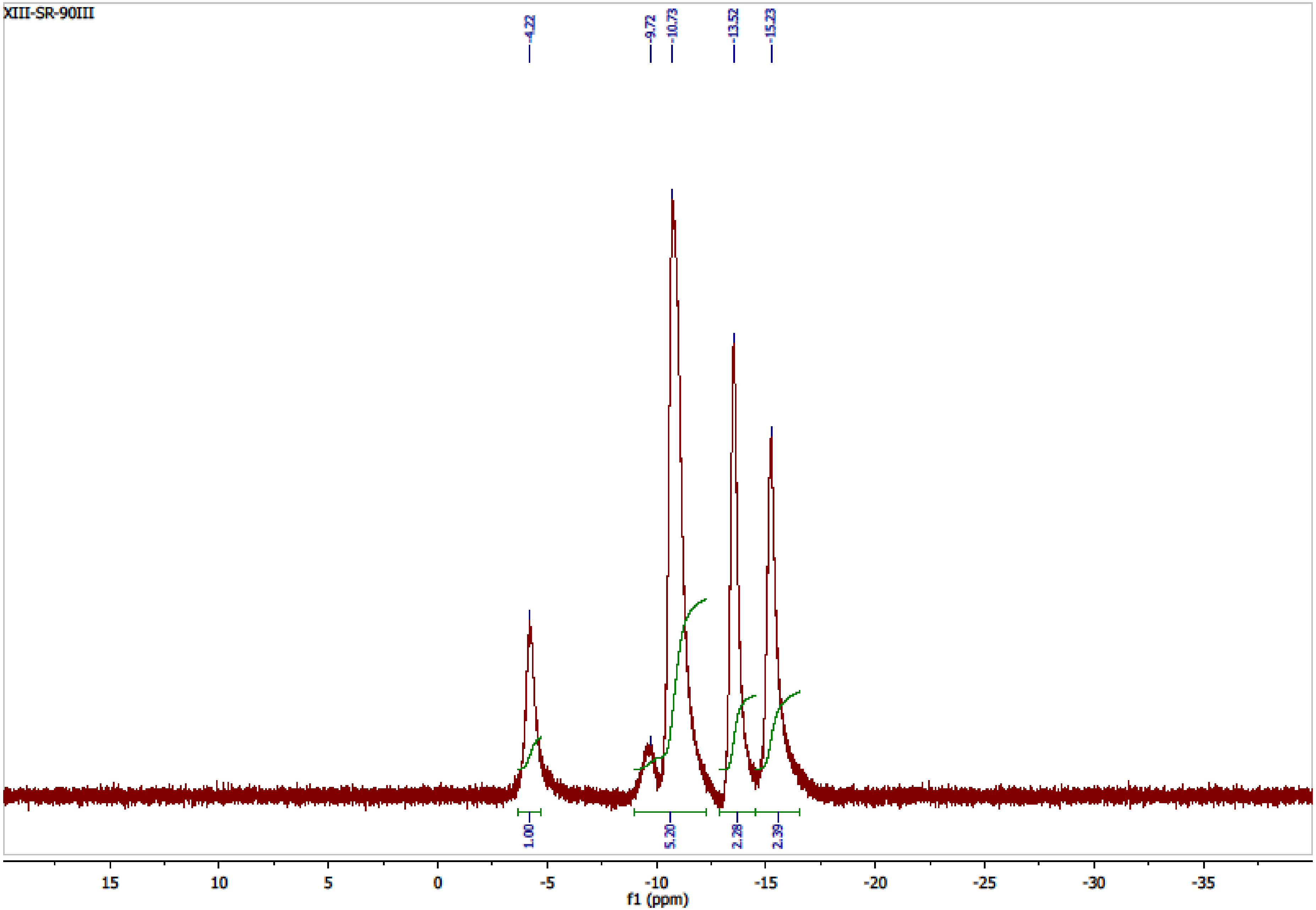

Figure S85. ^{11}B-NMR {^{1}H BB} spectrum of **33**.

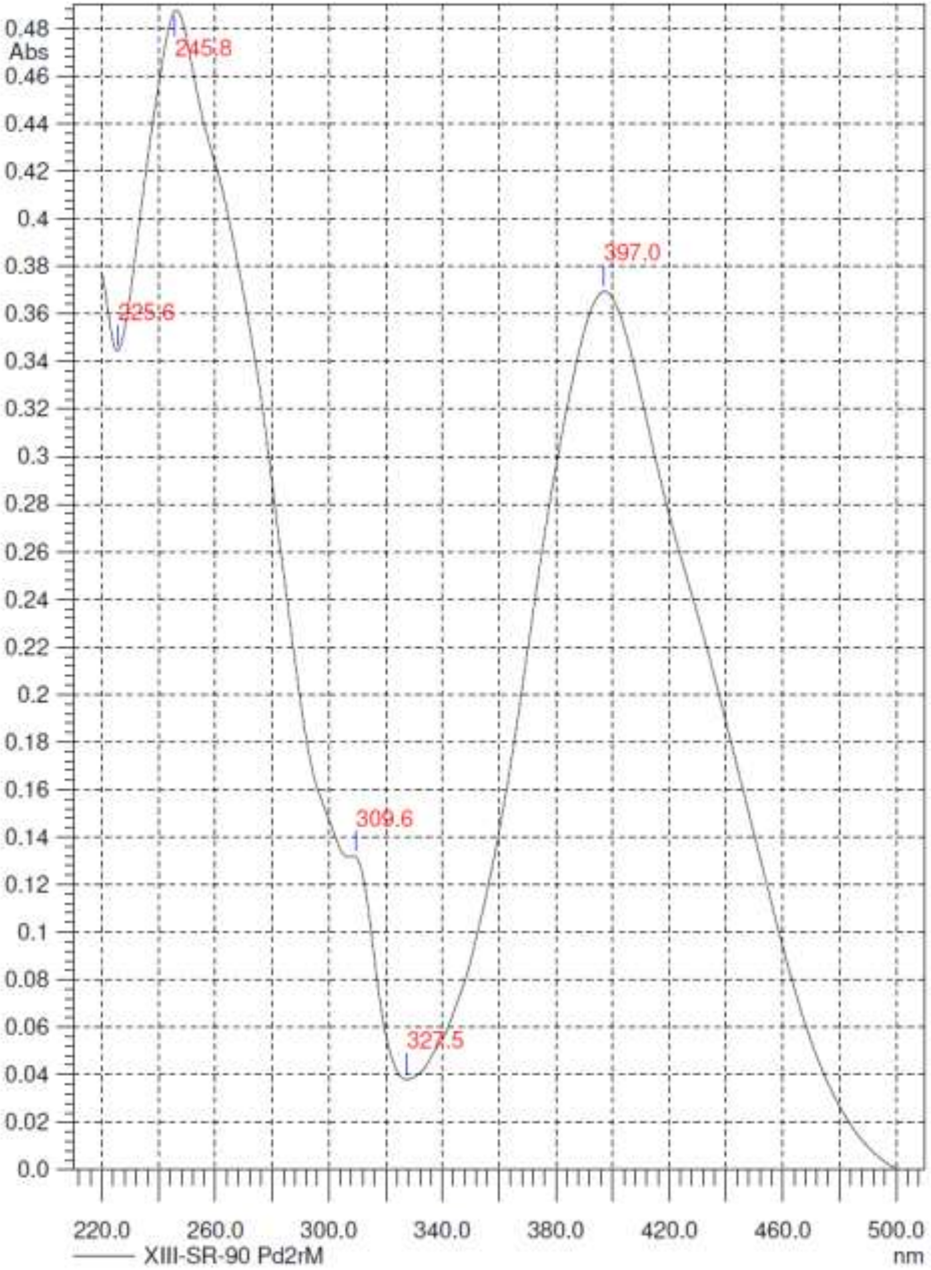

Figure S86. UV spectrum of **33**.

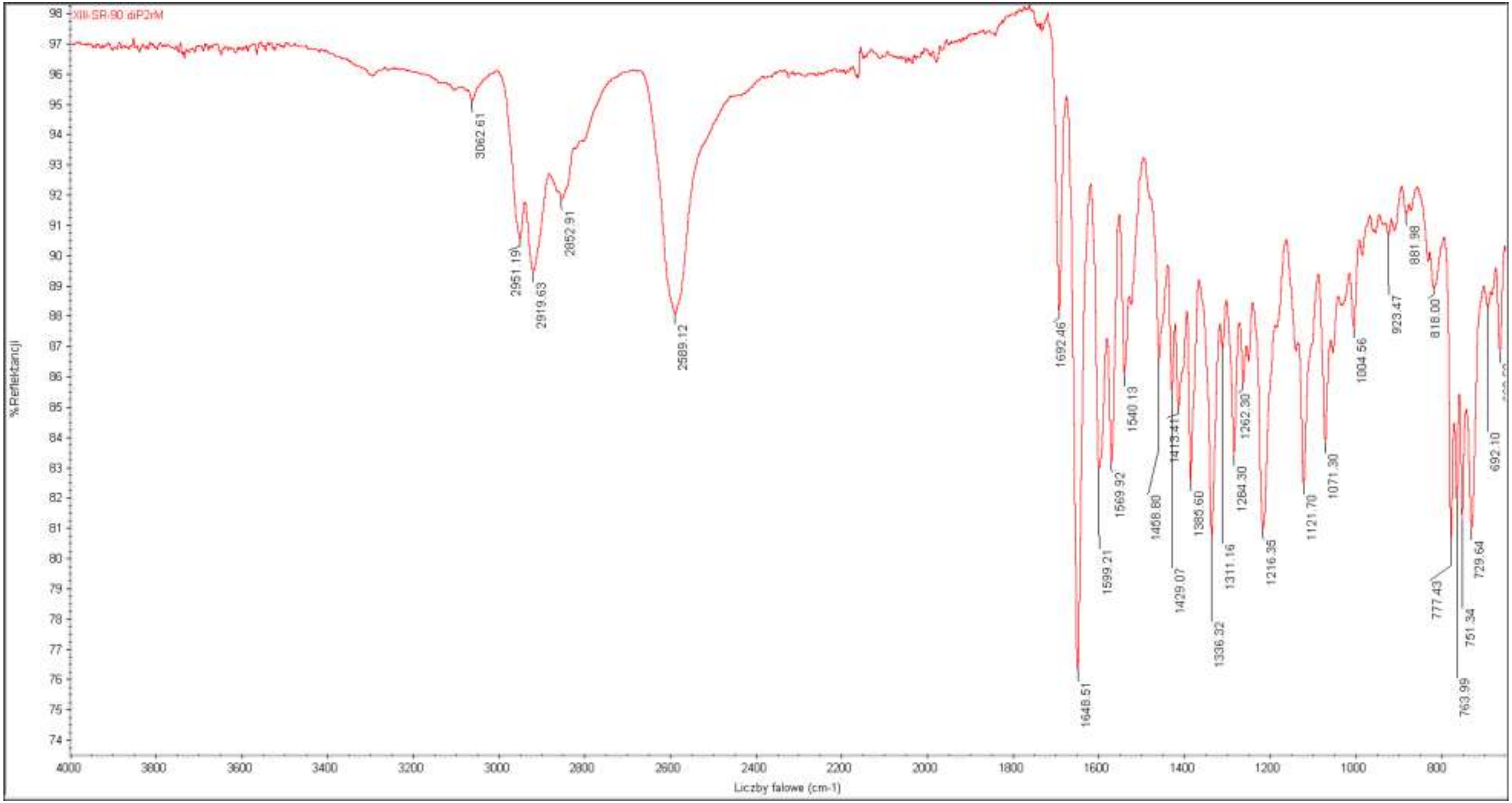

Figure S87. IR spectrum of **33**.

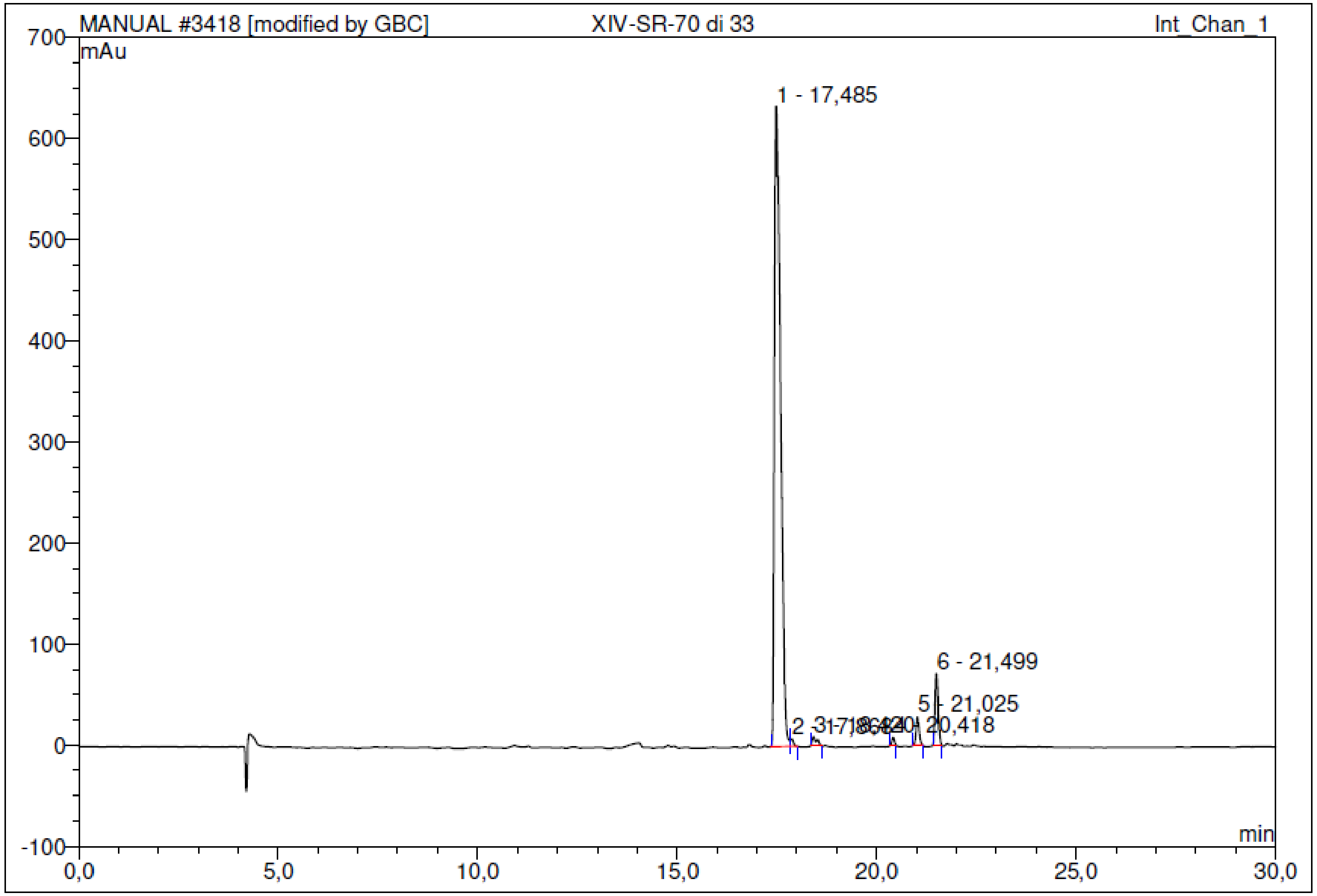

No.	Ret.Time min	Peak Name	Height mAu	Area mAu*min	Rel.Area %	Amount	Type
1	17,48	n.a.	633,219	108,121	90,64	n.a.	BM *
2	17,87	n.a.	7,304	0,703	0,59	n.a.	MB*
3	18,42	n.a.	9,048	1,142	0,96	n.a.	BMB*
4	20,42	n.a.	8,222	0,668	0,56	n.a.	BMB*
5	21,02	n.a.	28,446	2,504	2,10	n.a.	BMB*
6	21,50	n.a.	71,134	6,154	5,16	n.a.	BMB*
Total:			757,373	119,292	100,00	0,000	

Figure S88. HPLC analysis of **33**.

Spectrum Name: XIV-SR-78_33_pt
Start Ion: 100
End Ion: 1000
Source: APCI + 10.0µA 400C
Capillary: 150V 300C Offset: 25V Span: 0V

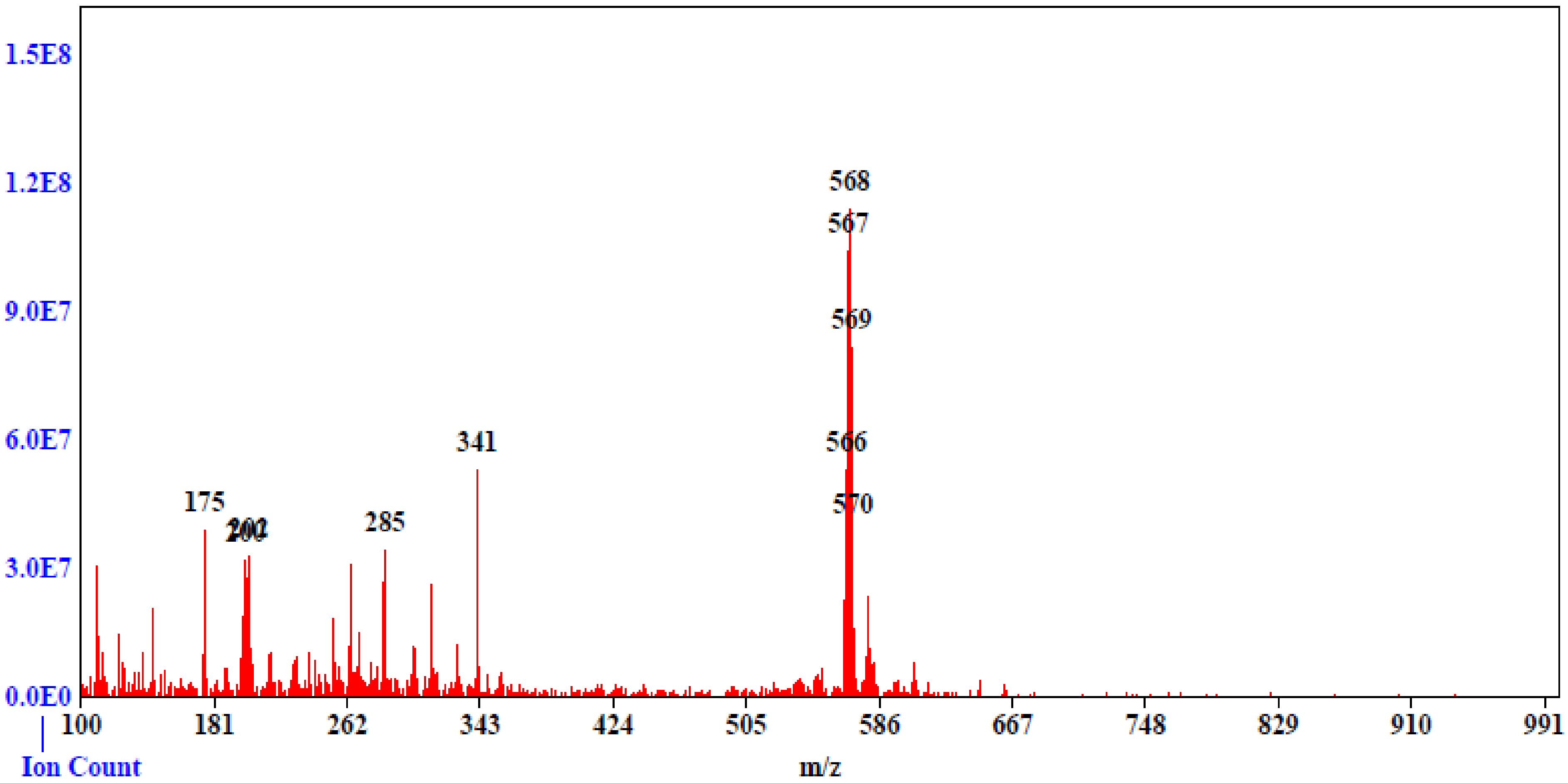

Figure S89. MS spectrum of **33**.

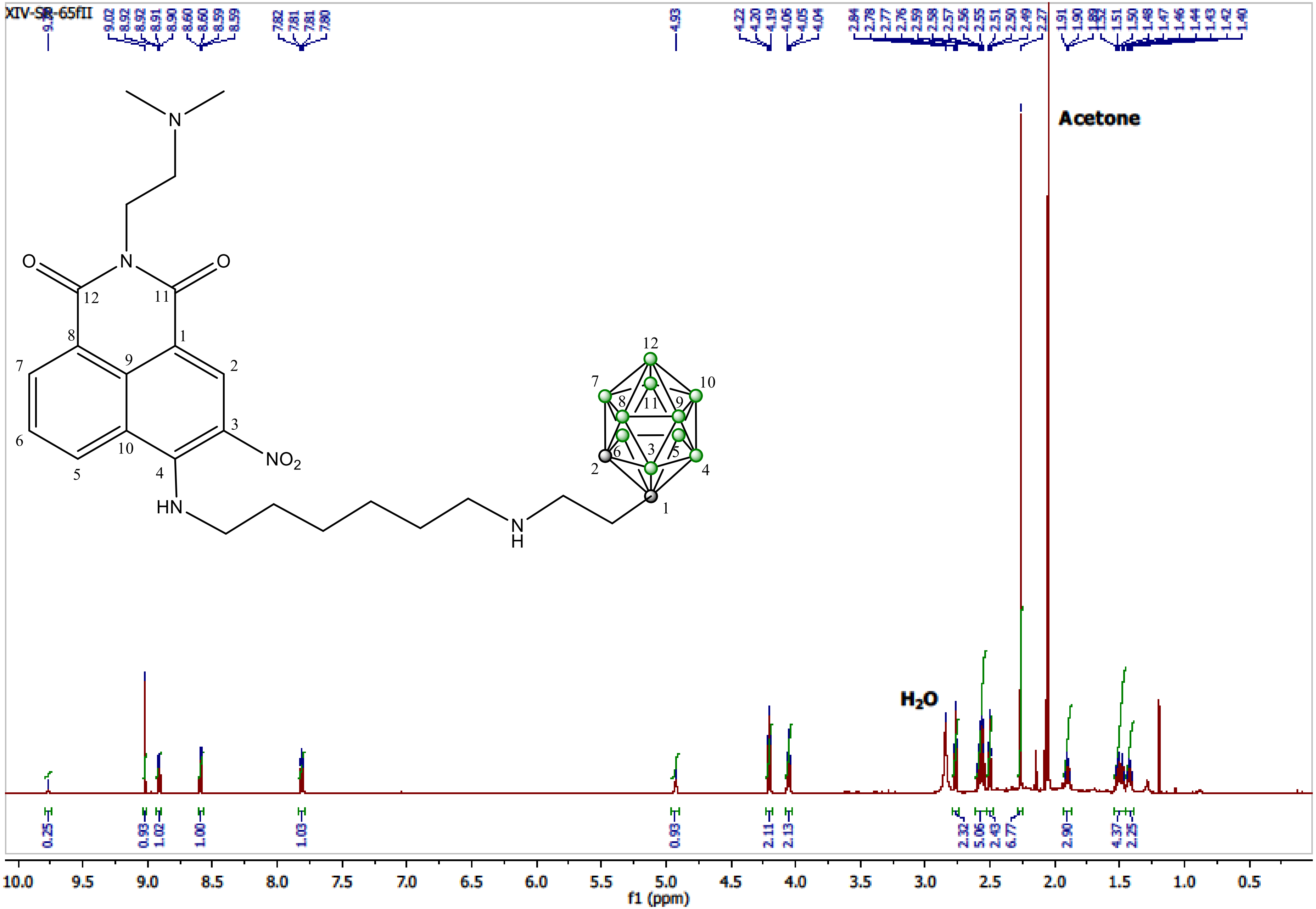

Figure S90. ^{1}H-NMR spectrum of **34**.

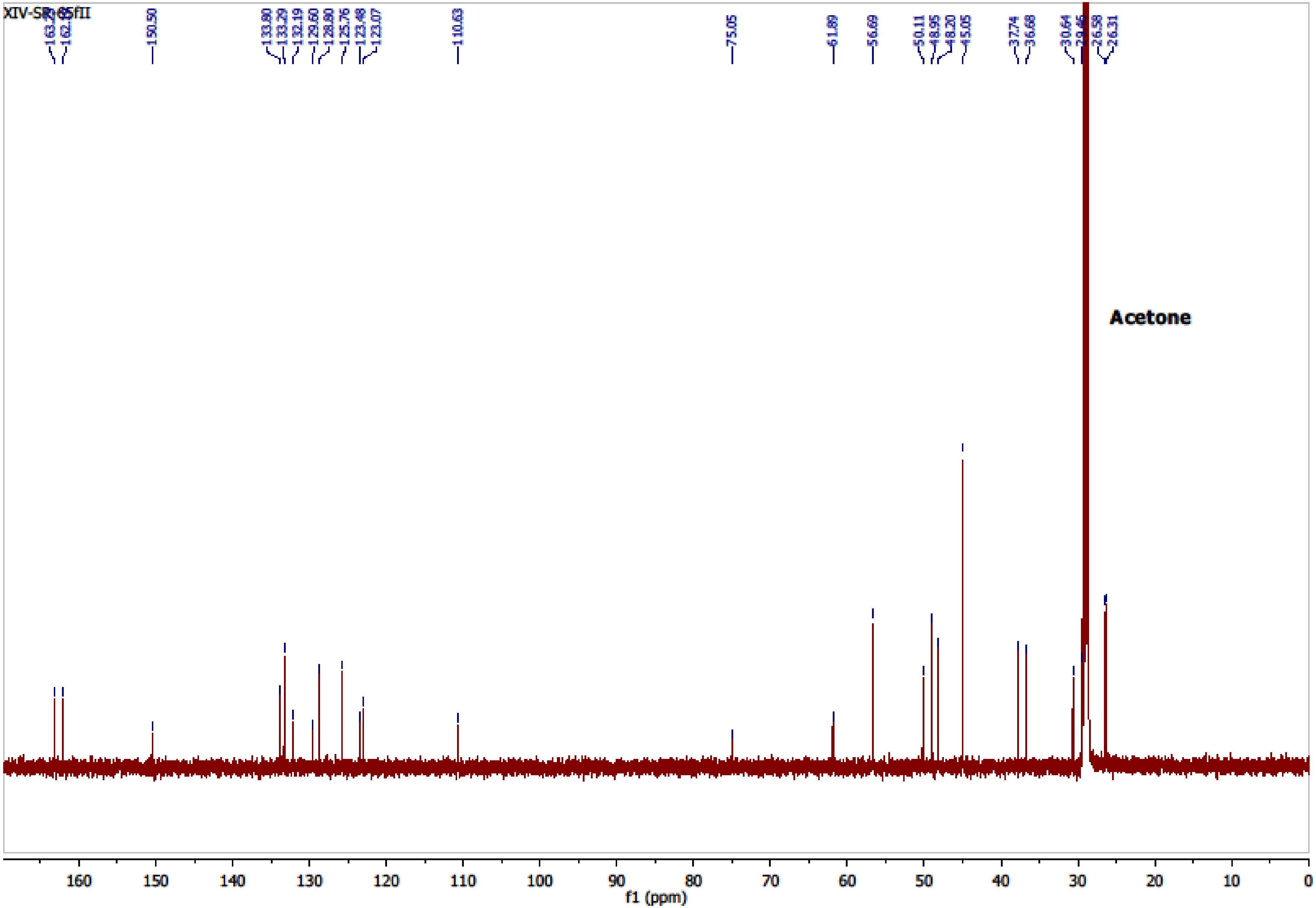

Figure S91. ^{13}C-NMR spectrum of **34**.

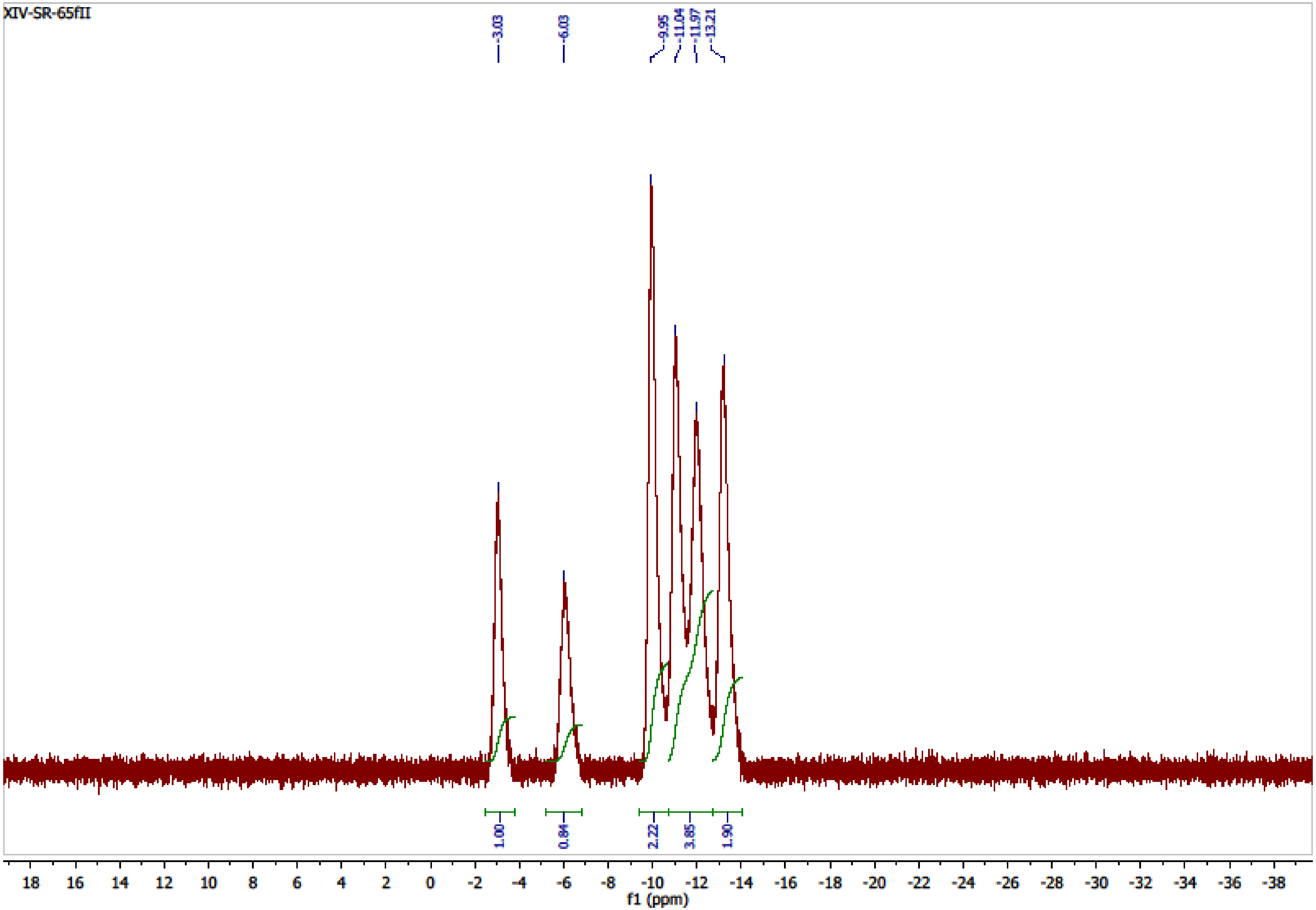

Figure S92. ^{11}B-NMR {^{1}H BB} spectrum of **34**.

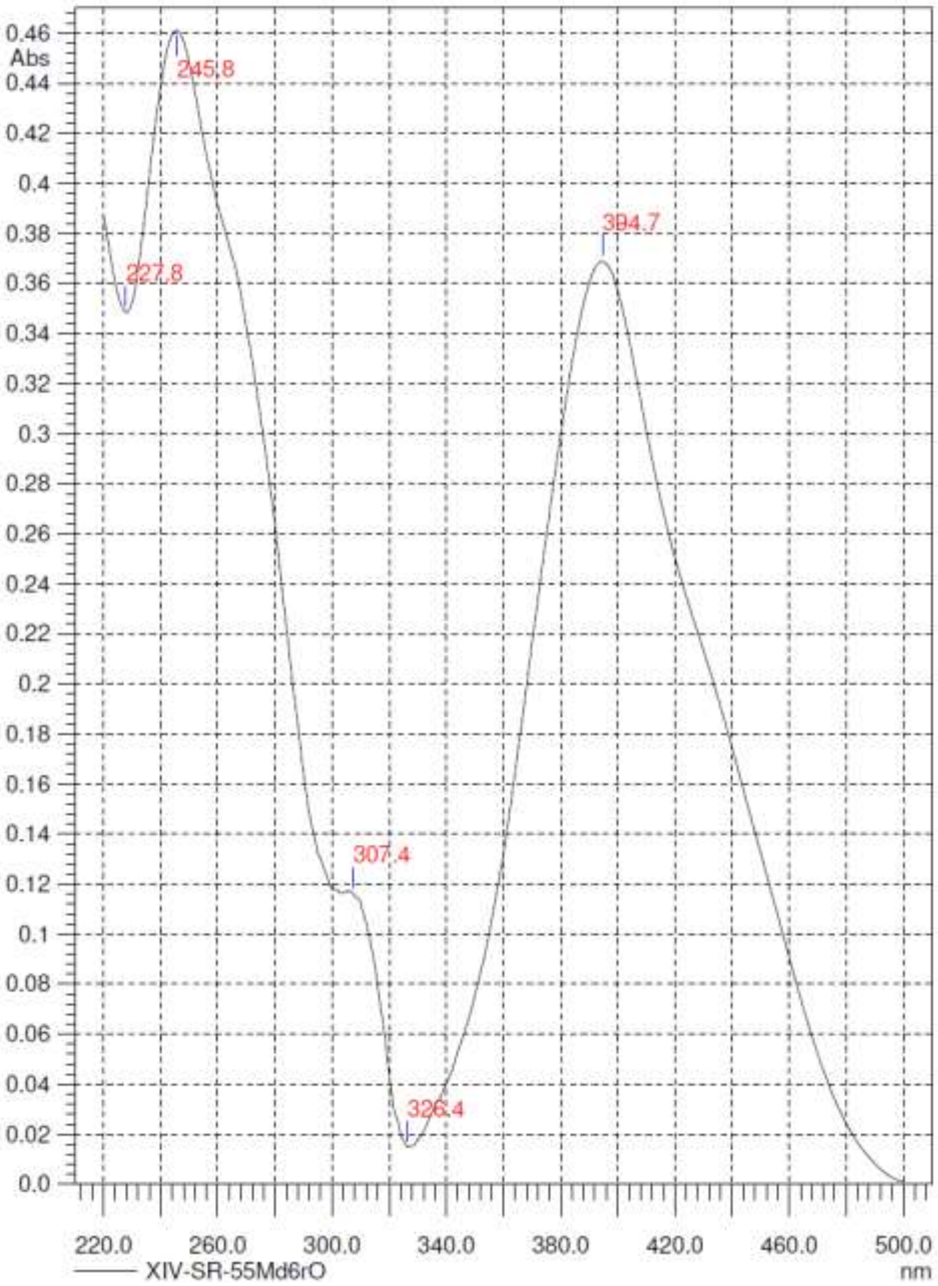

Figure S93. UV spectrum of **34**.

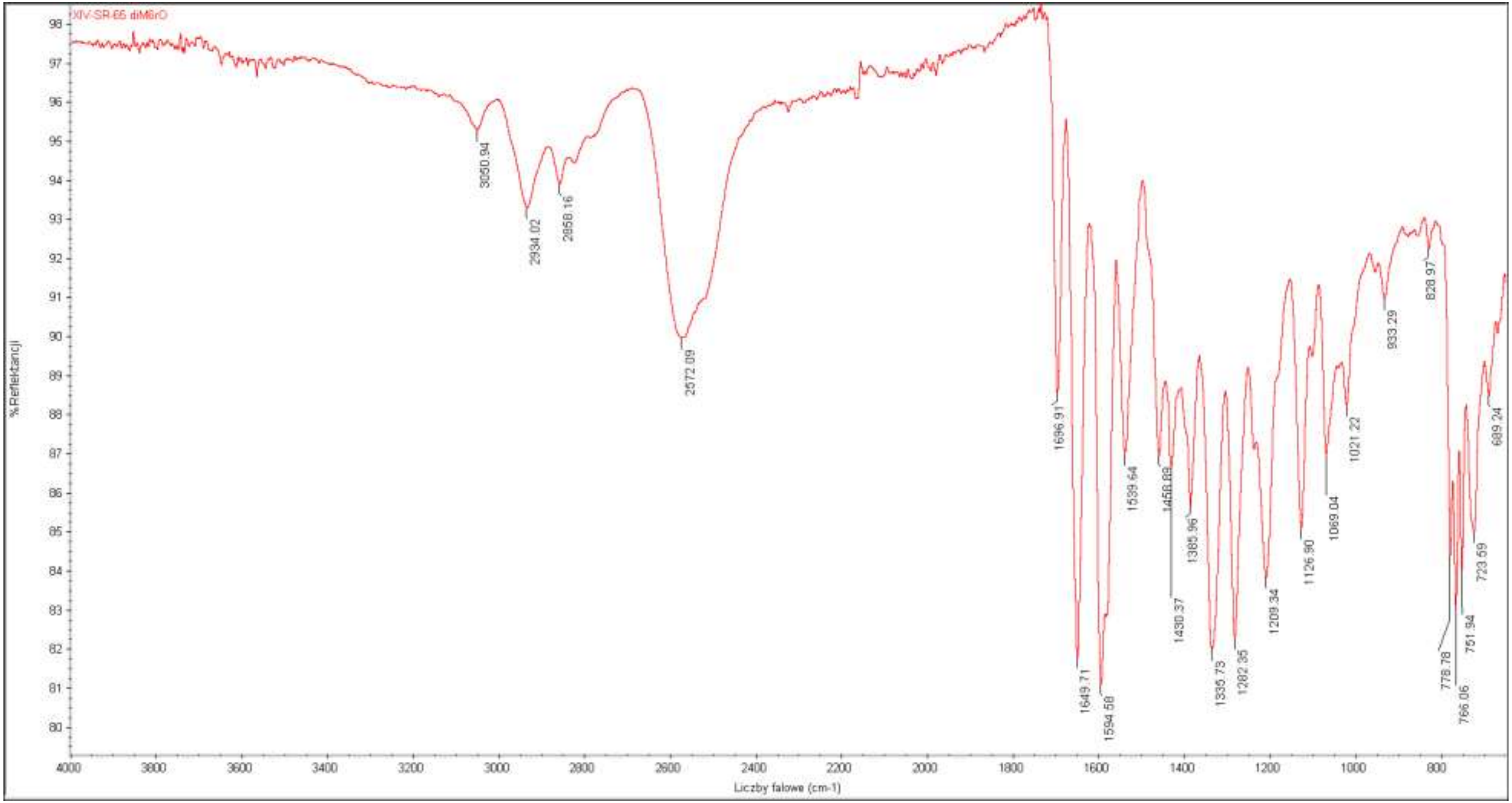

Figure S94. IR spectrum of **34**.

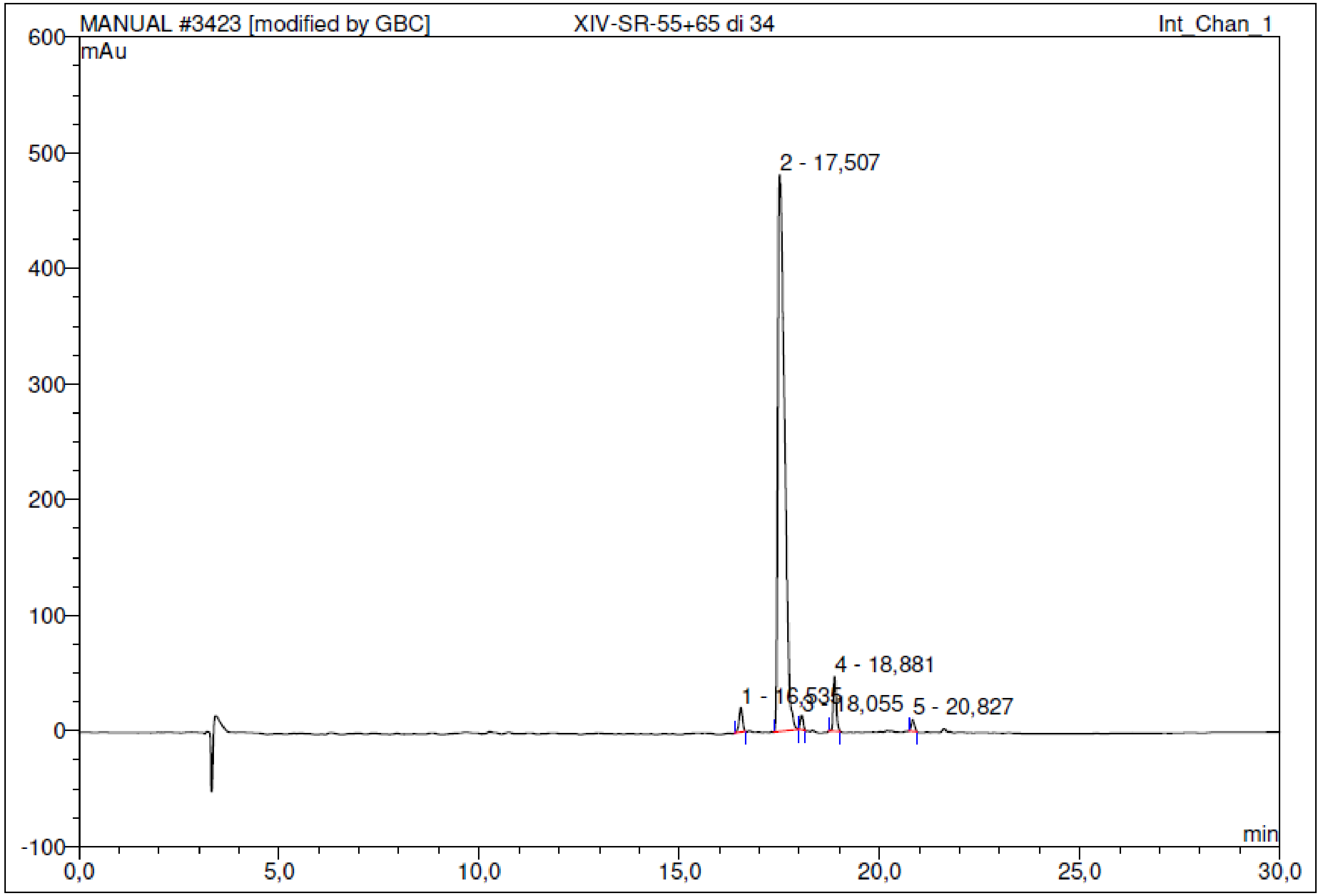

No.	Ret.Time min	Peak Name	Height mAu	Area mAu*min	Rel.Area %	Amount	Type
1	16,54	n.a.	21,171	2,028	1,99	n.a.	BMB*
2	17,51	n.a.	481,912	94,188	92,39	n.a.	BMB
3	18,06	n.a.	12,392	0,940	0,92	n.a.	BMB*
4	18,88	n.a.	47,167	3,857	3,78	n.a.	BMB*
5	20,83	n.a.	10,082	0,937	0,92	n.a.	BMB*
Total:			572,724	101,950	100,00	0,000	

Figure S95. HPLC analysis of **34**.

Spectrum Name: XV-SR-01_M6rO_pt
Start Ion: 100
End Ion: 1200
Source: APCI + 10.0µA 400C
Capillary: 150V 300C Offset: 25V Span: 0V

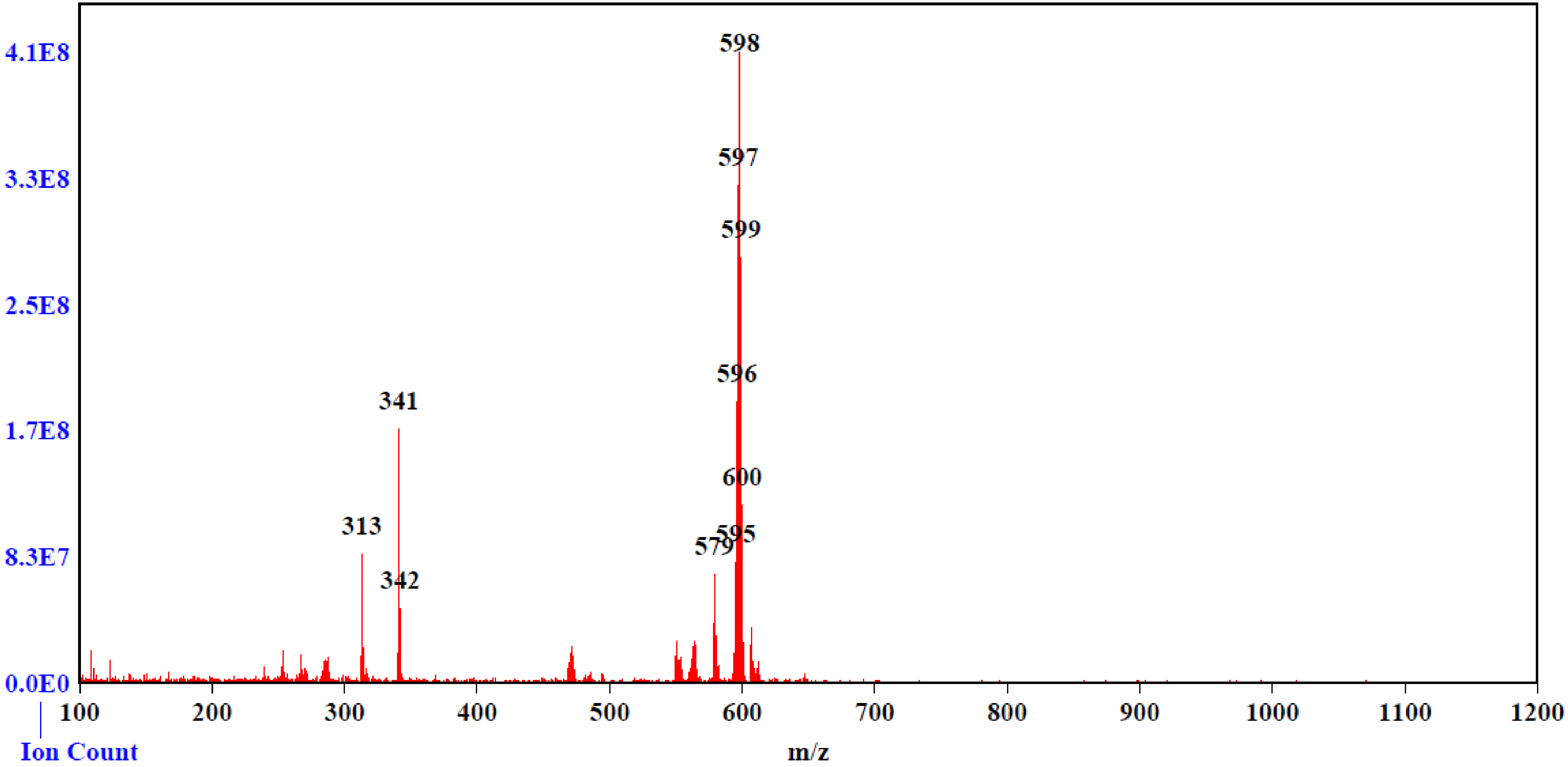

Figure S96. MS spectrum of **34**.

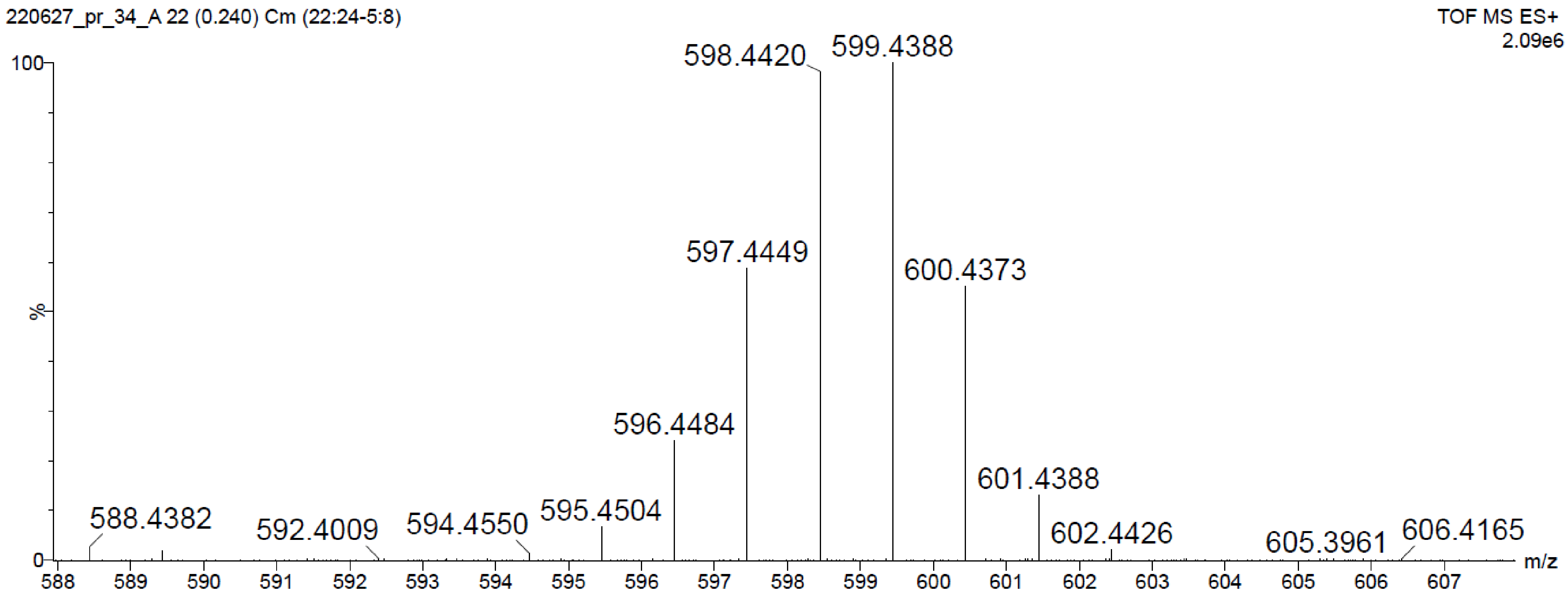

Figure S97. HRMS spectrum of **34**.

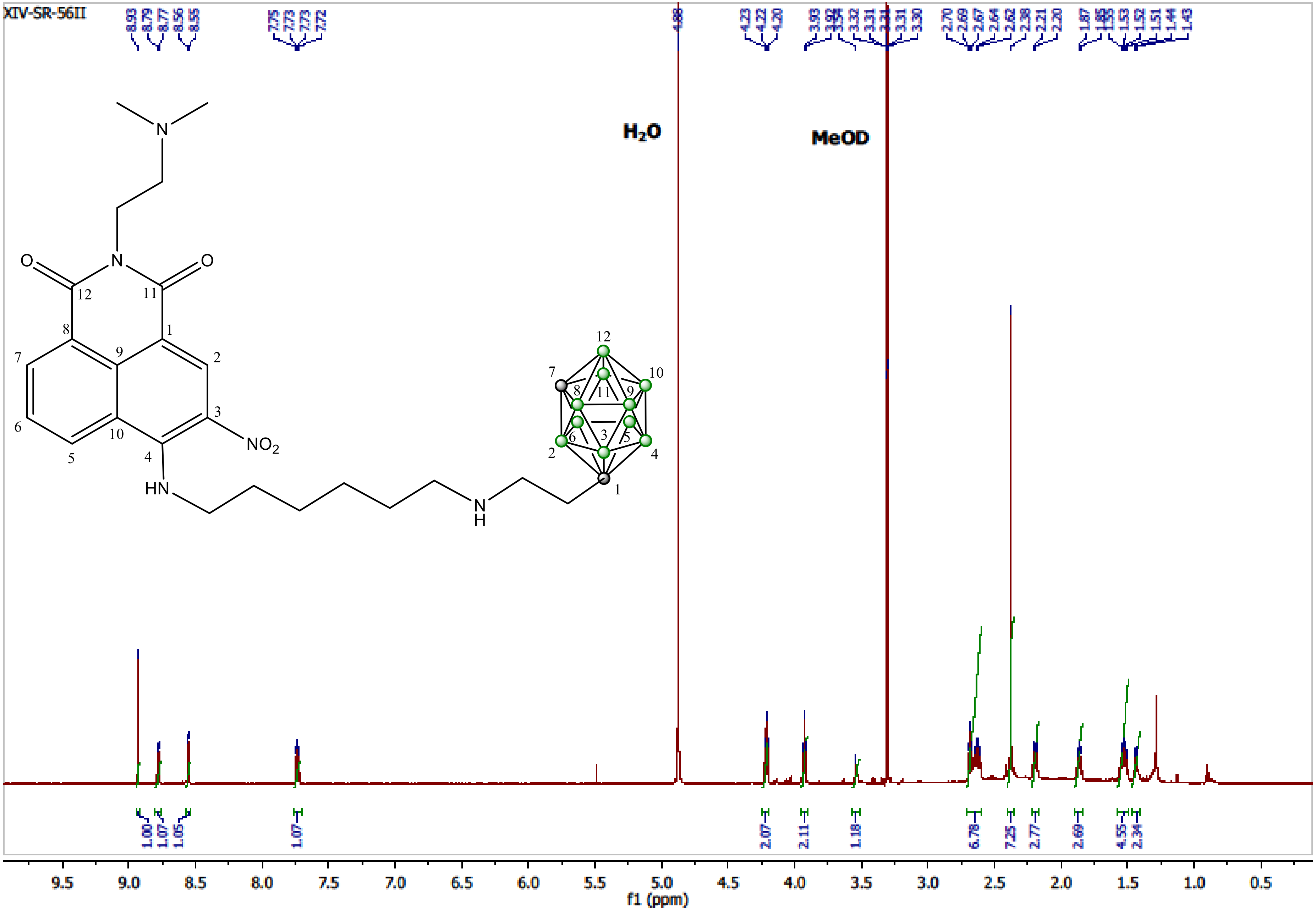

Figure S98. ^{1}H-NMR spectrum of **35**.

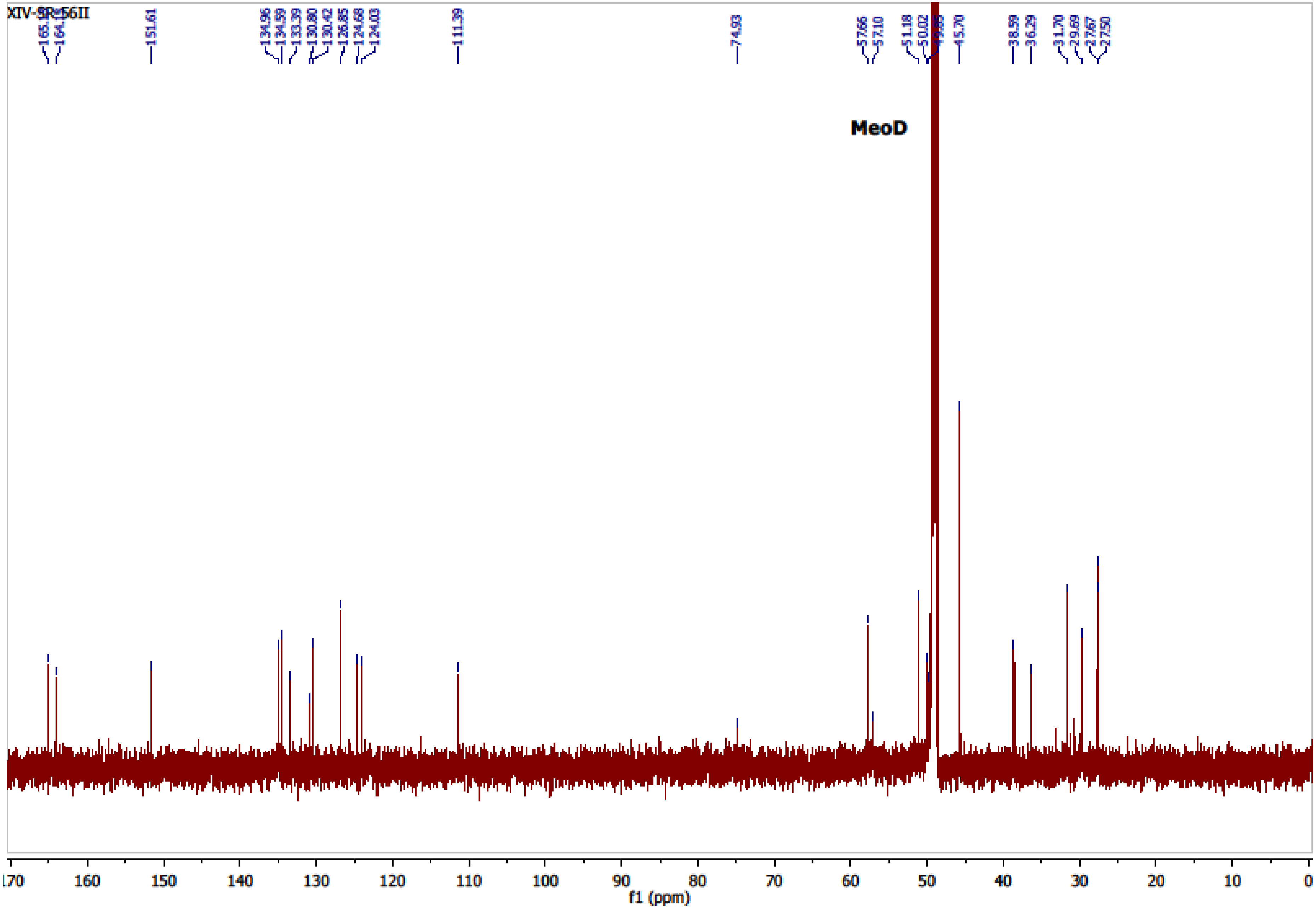

Figure S99. ^{13}C-NMR spectrum of **35**.

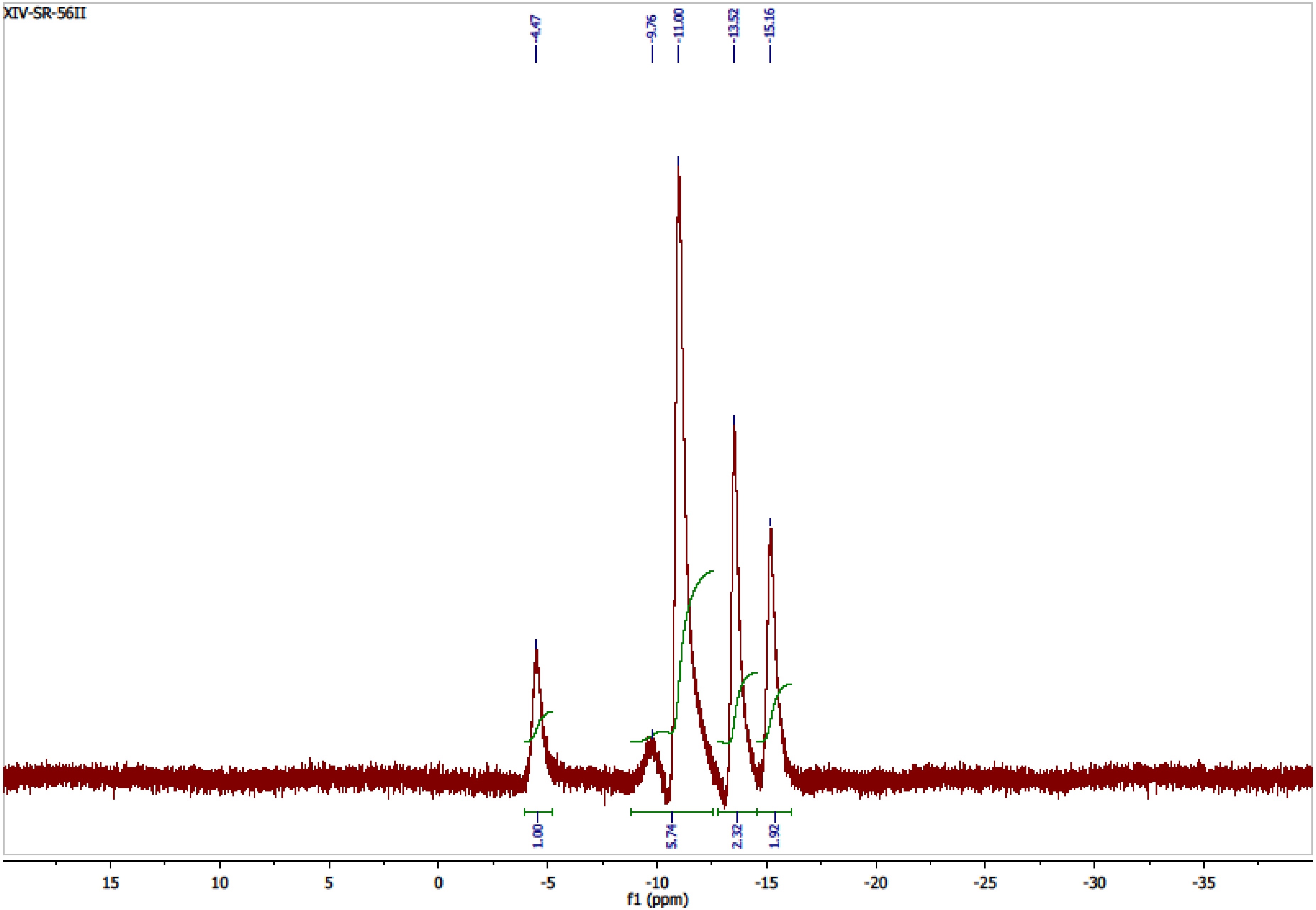

Figure S100. ^{11}B-NMR {^{1}H BB} spectrum of **35**.

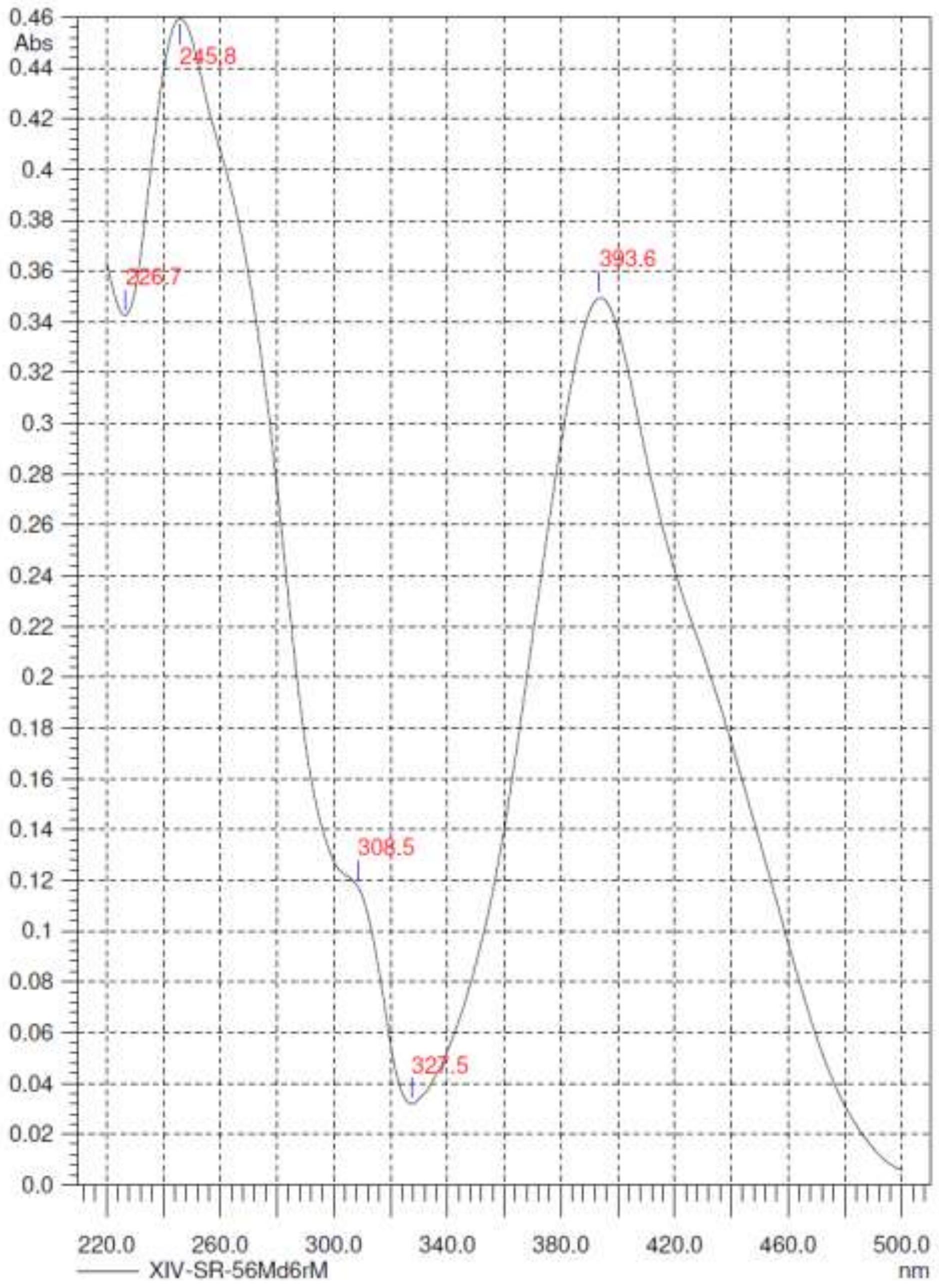

Figure S101. UV spectrum of **35**.

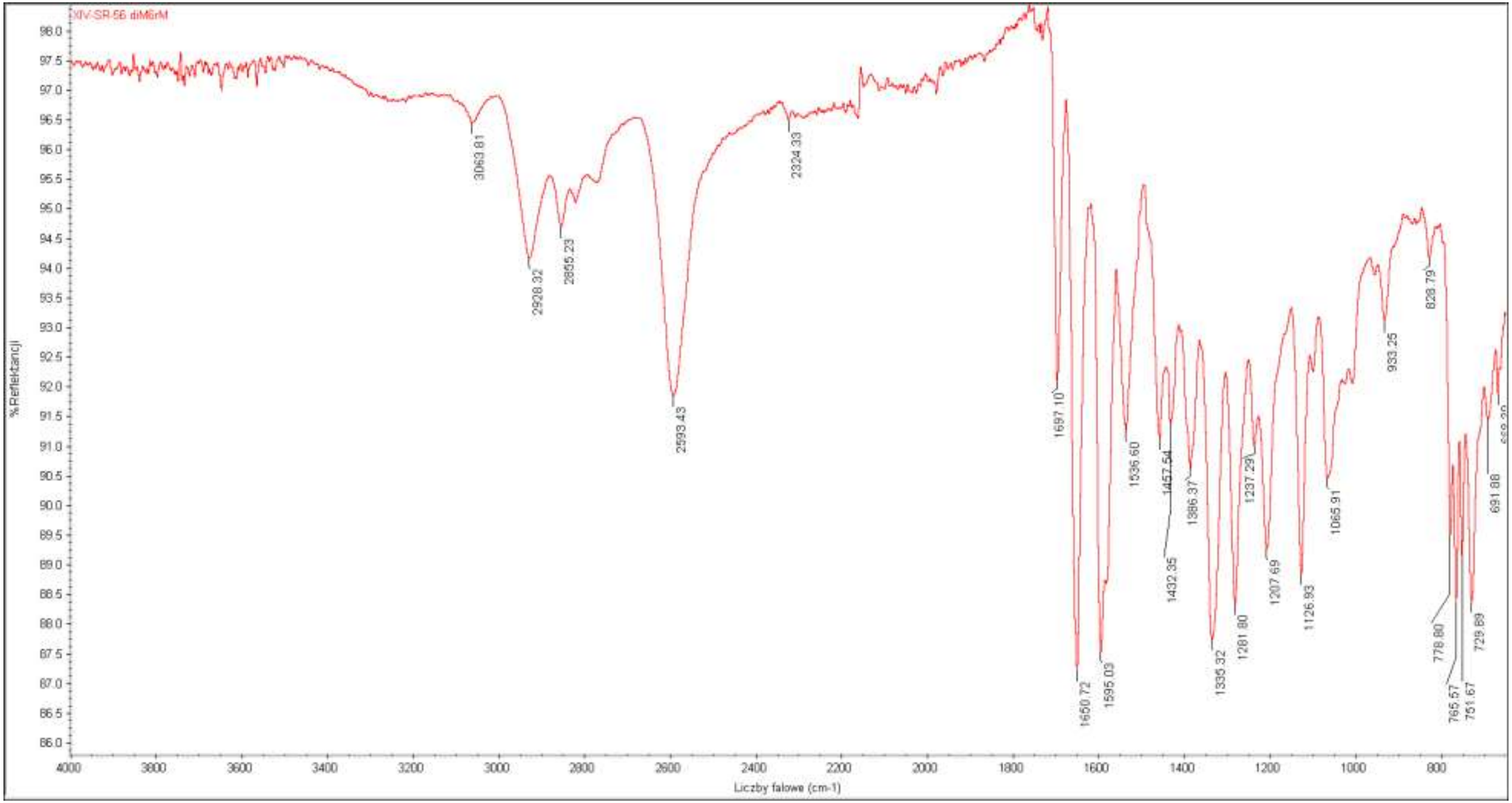

Figure S102. IR spectrum of **35**.

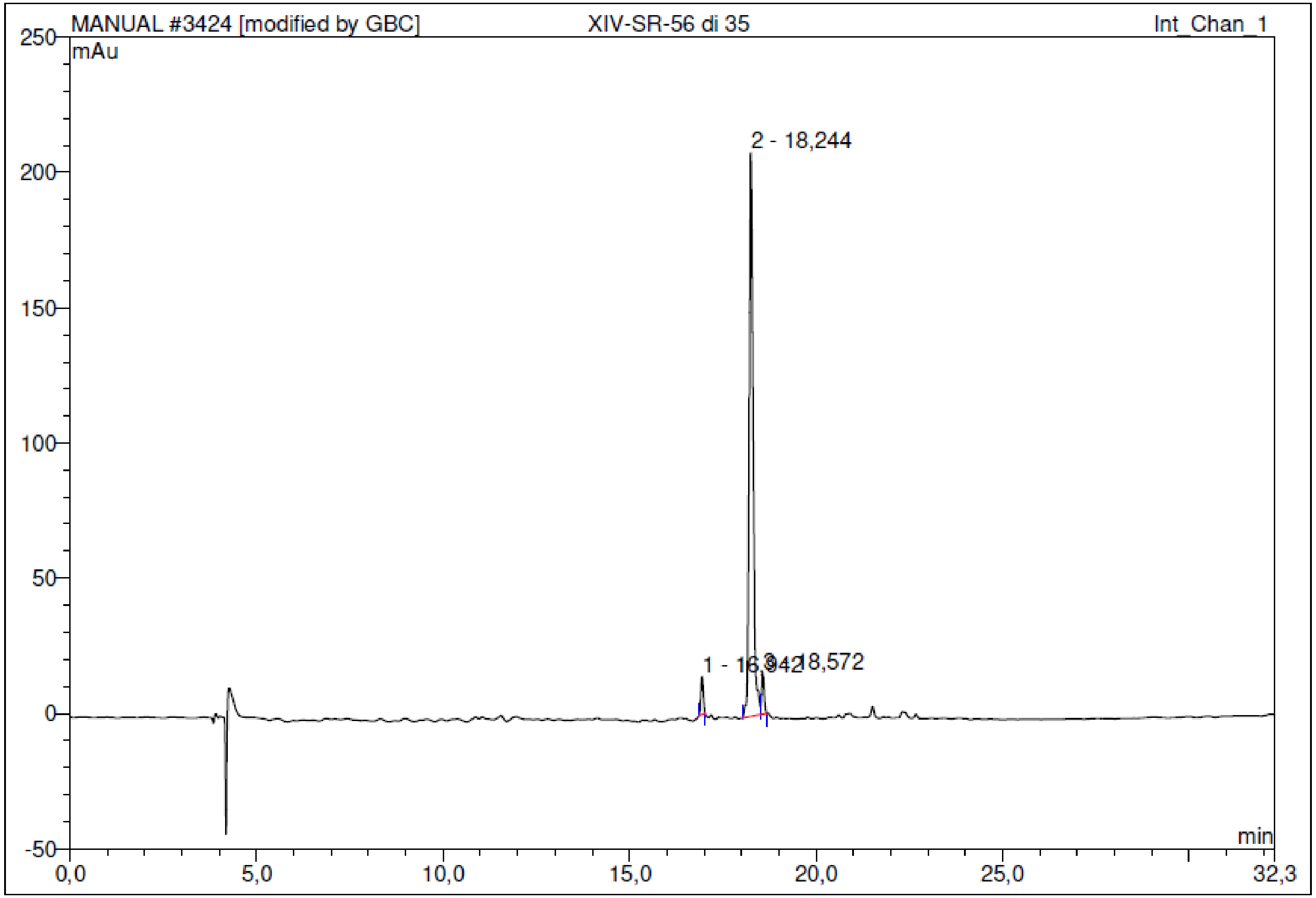

No.	Ret.Time min	Peak Name	Height mAu	Area mAu*min	Rel.Area %	Amount	Type
1	16,94	n.a.	13,970	1,031	3,68	n.a.	BMB*
2	18,24	n.a.	208,337	25,874	92,27	n.a.	BM *
3	18,57	n.a.	14,976	1,136	4,05	n.a.	MB*
Total:			237,283	28,042	100,00	0,000	

Figure S103. HPLC analysis of **35**.

Spectrum Name: XIV-SR-50_final_pt
Start Ion: 100
End Ion: 700
Source: APCI + 10.0µA 400C
Capillary: 150V 300C Offset: 25V Span: 0V

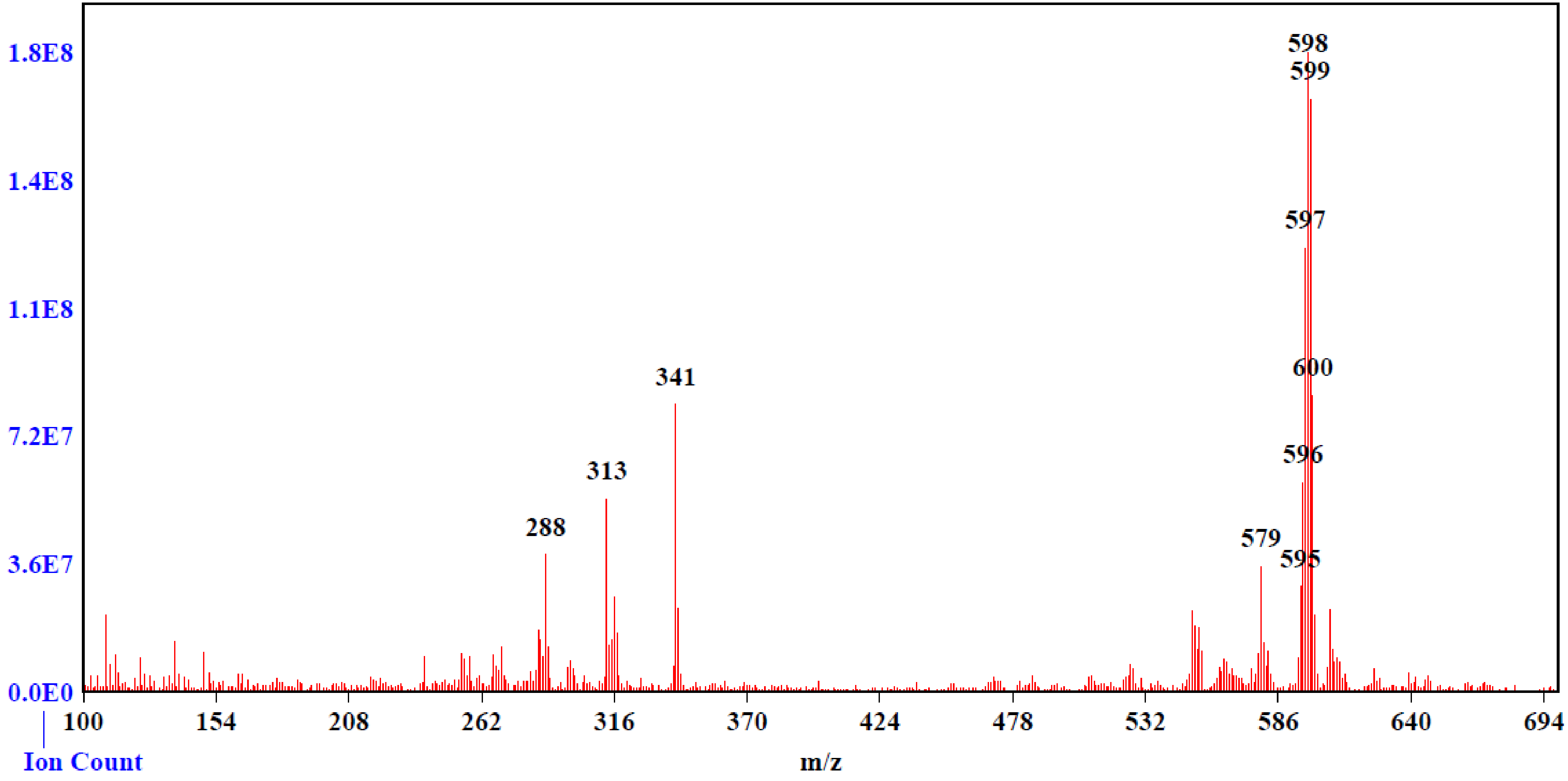

Figure S104. MS spectrum of **35**.

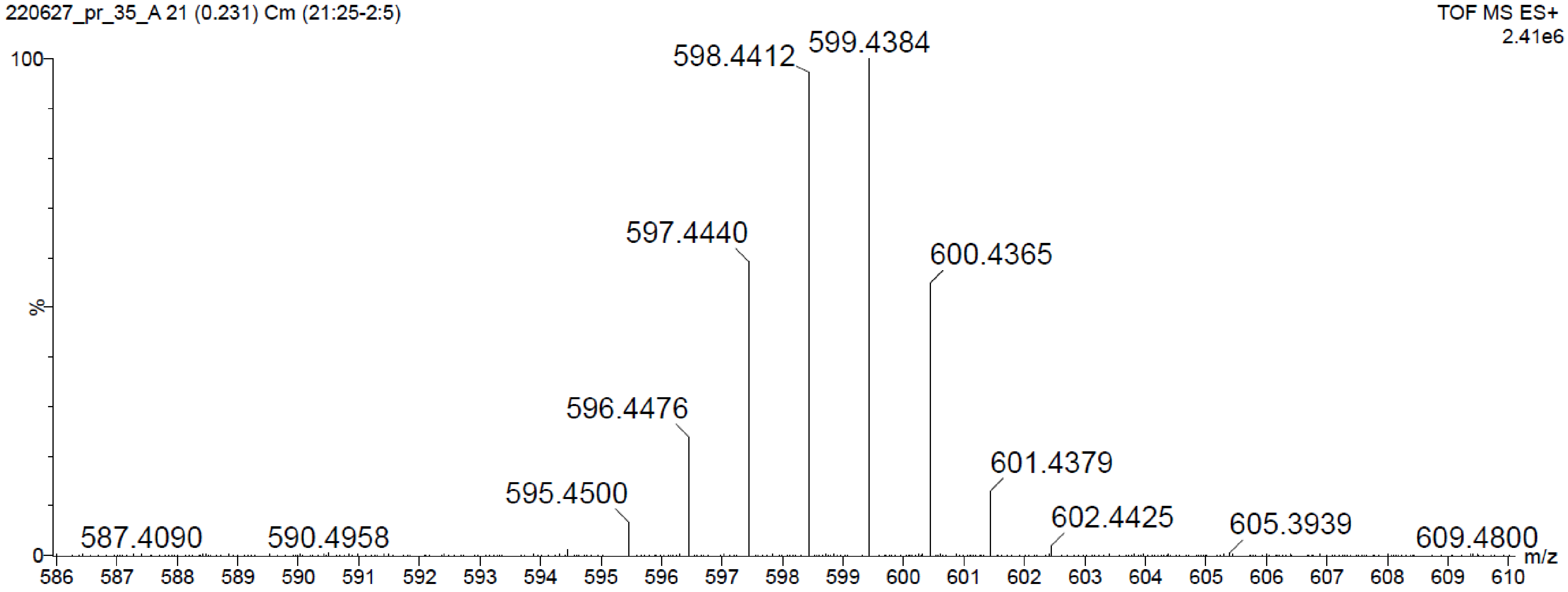

Figure S105. HRMS spectrum of **35**.

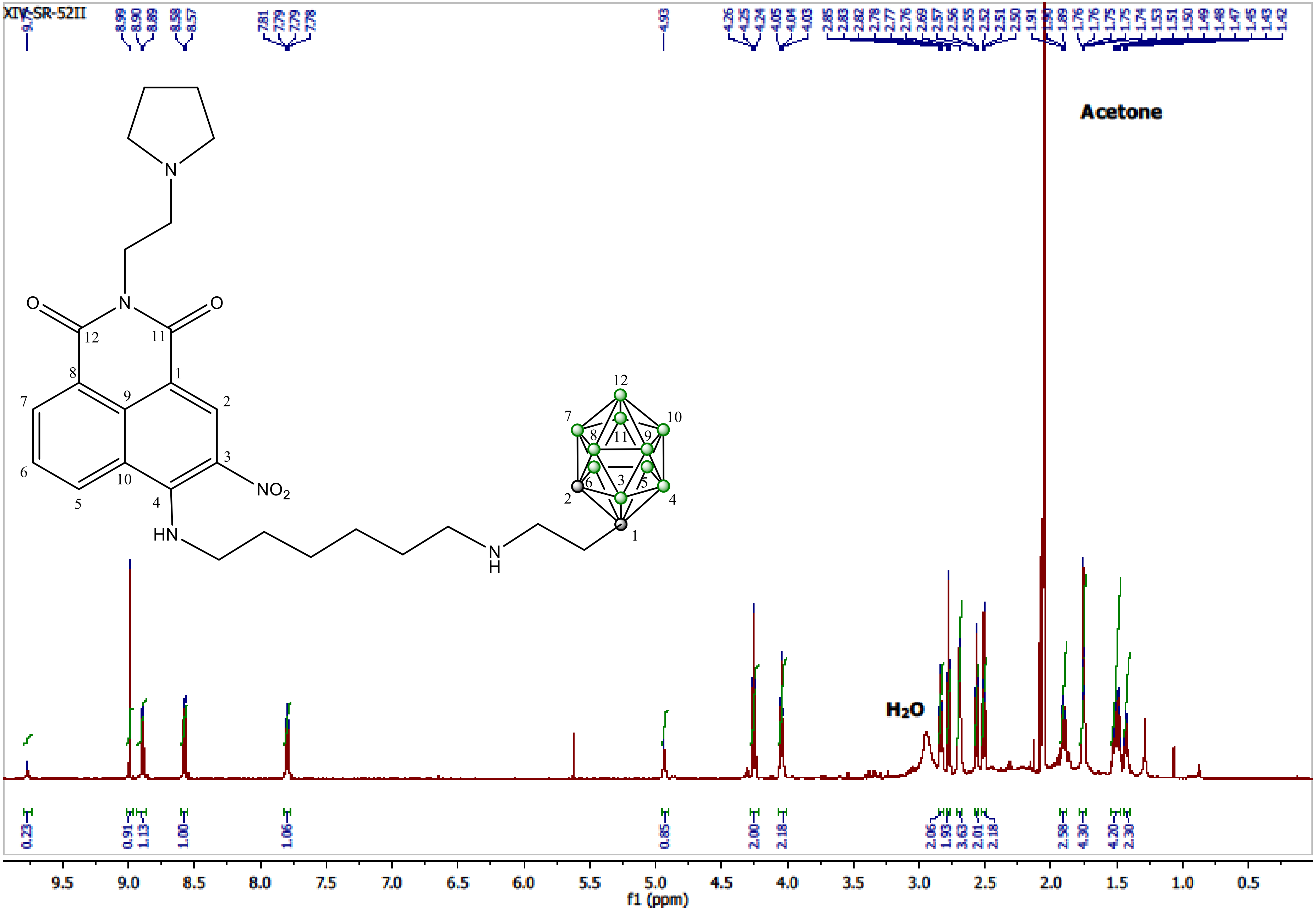

Figure S106. ^{1}H-NMR spectrum of **36**.

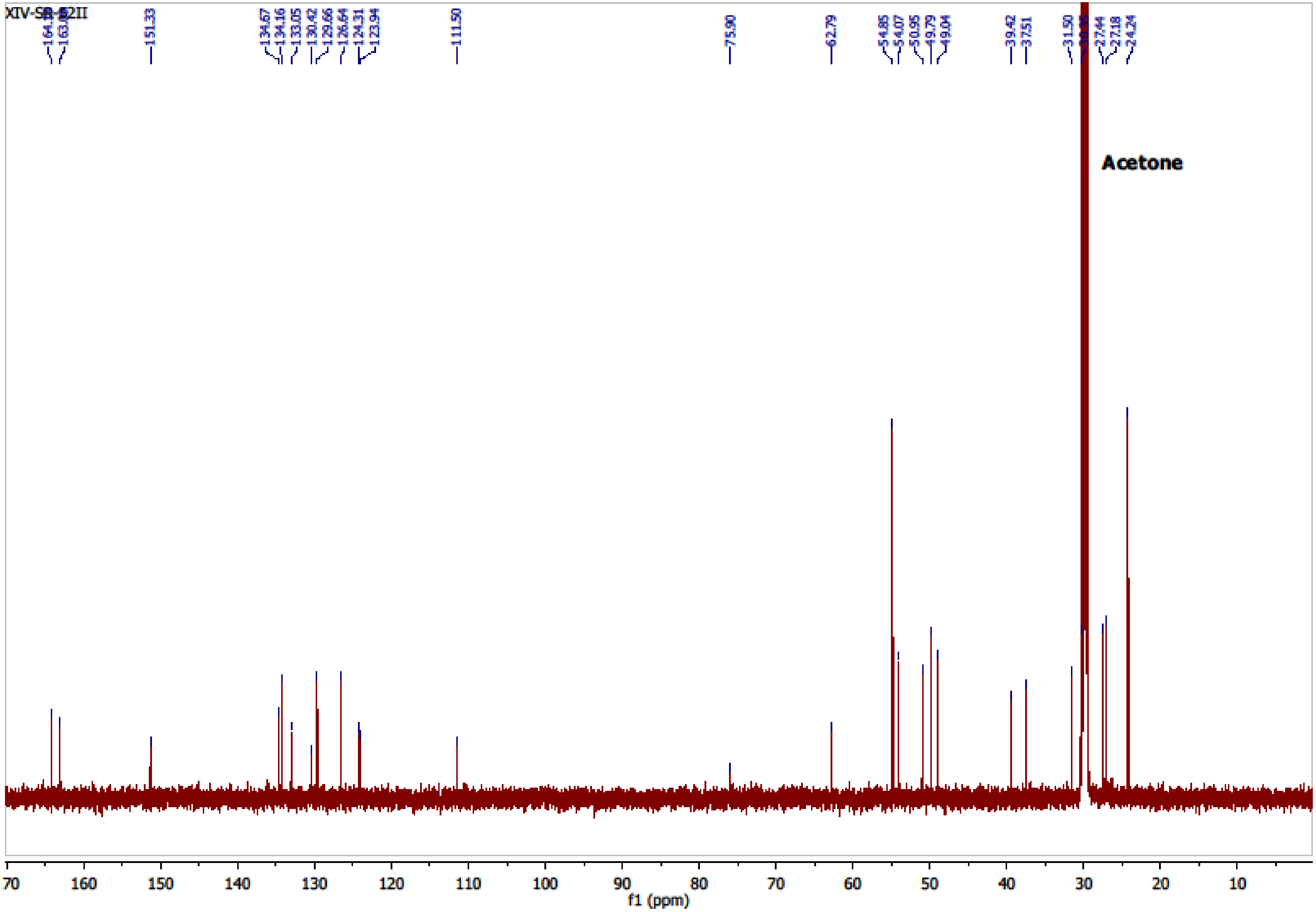

Figure S107. ^{13}C-NMR spectrum of **36**.

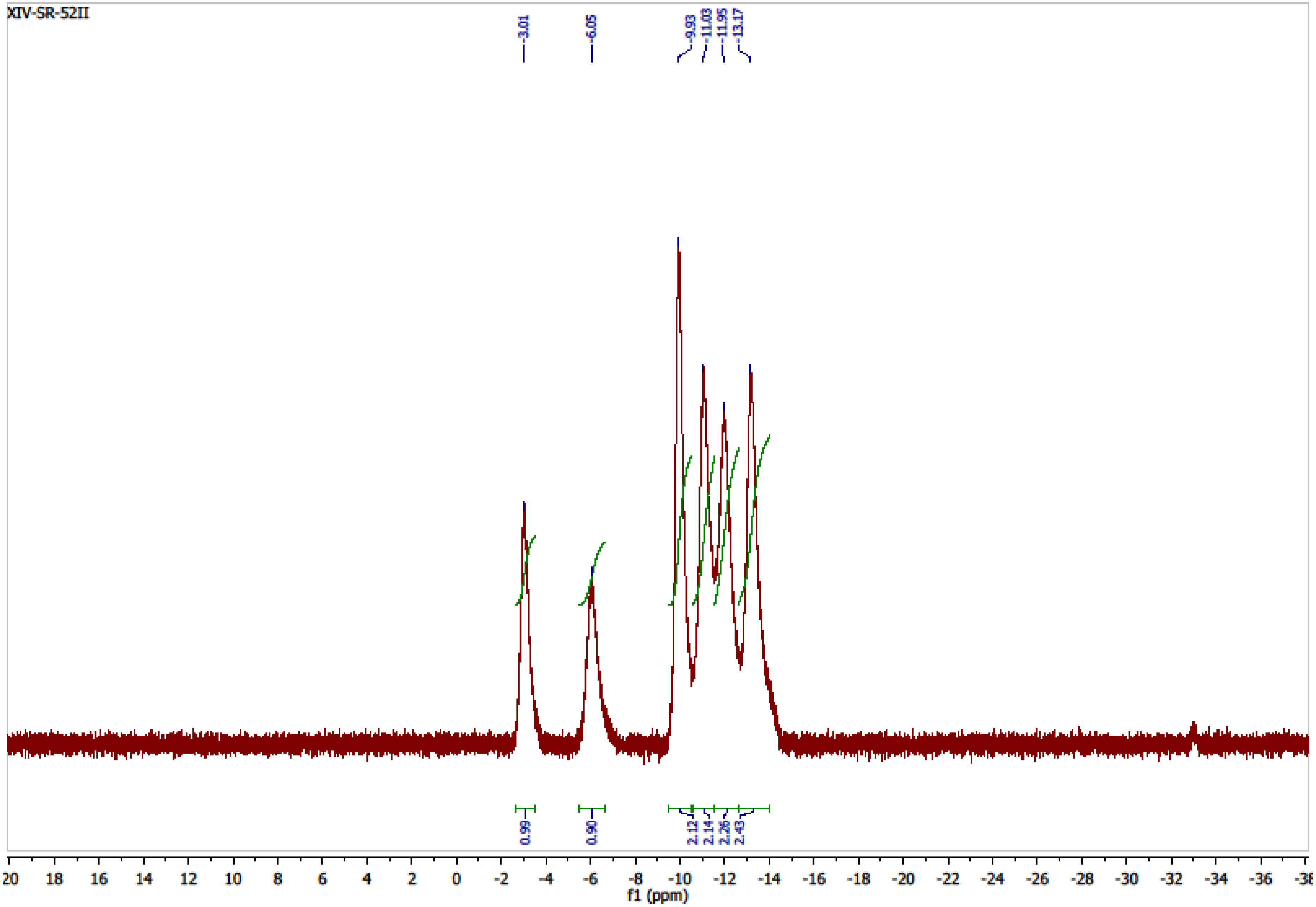

Figure S108. ^{11}B-NMR {^{1}H BB} spectrum of **36**.

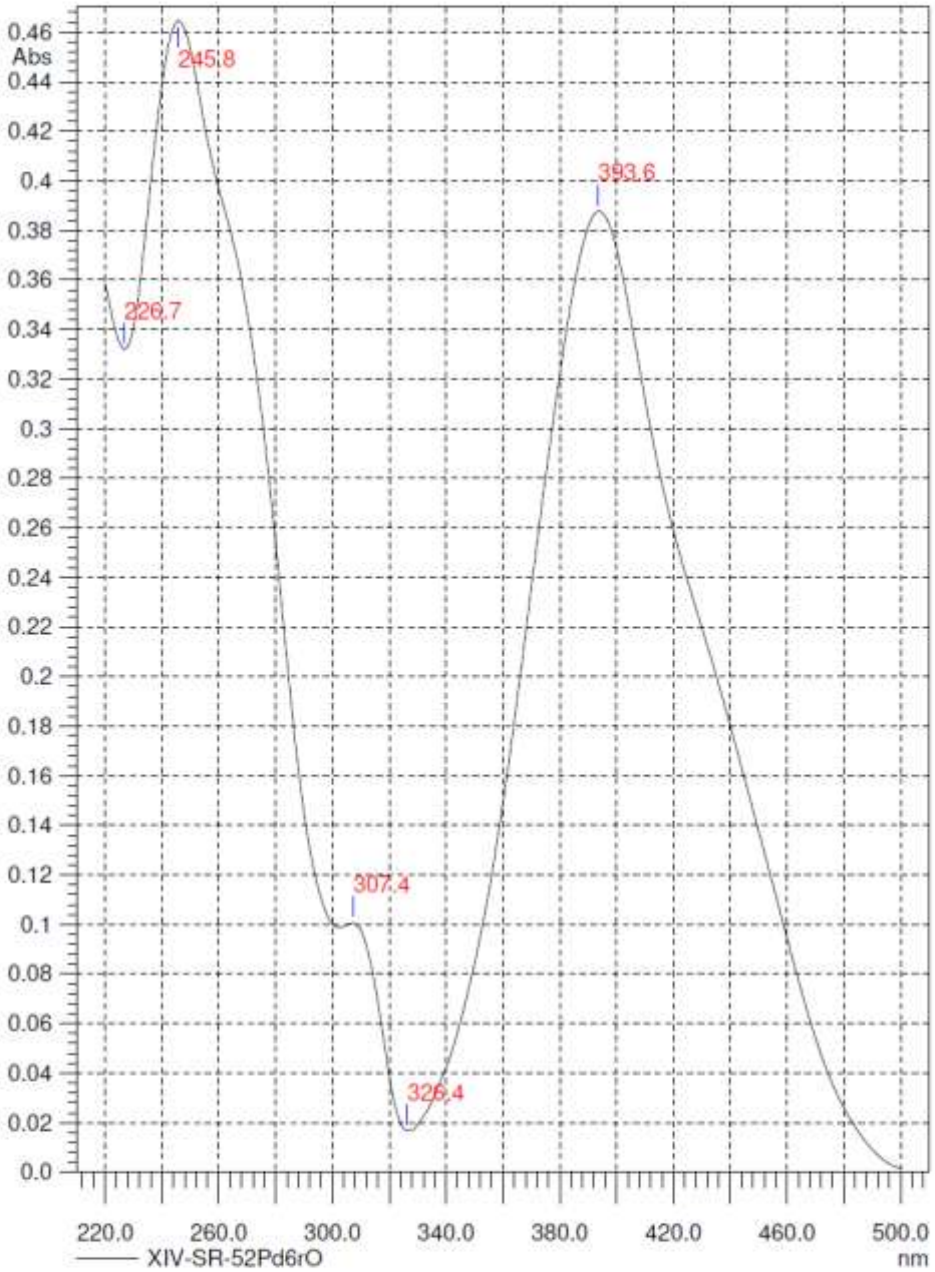

Figure S109. UV spectrum of **36**.

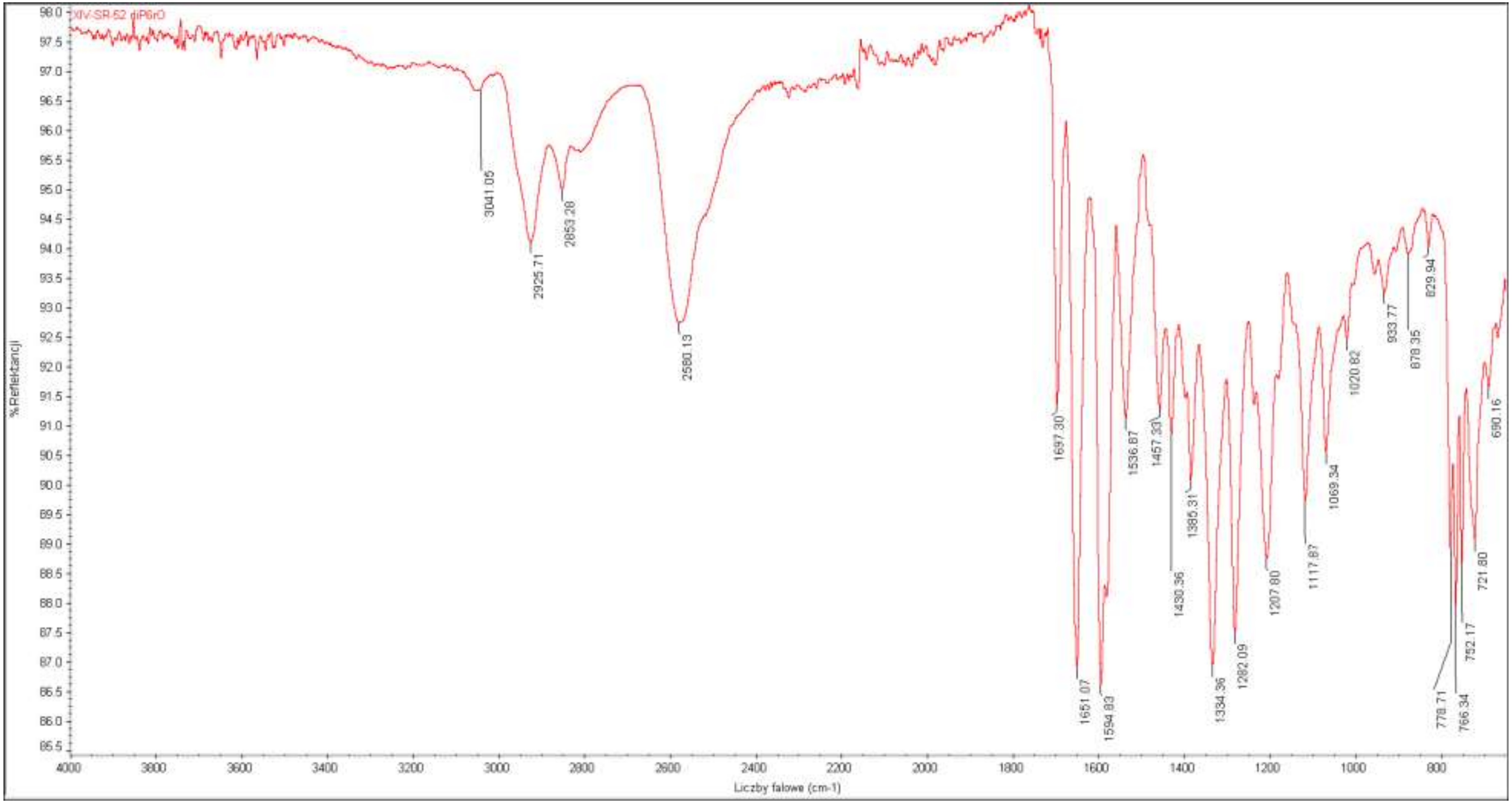

Figure S110. IR spectrum of **36**.

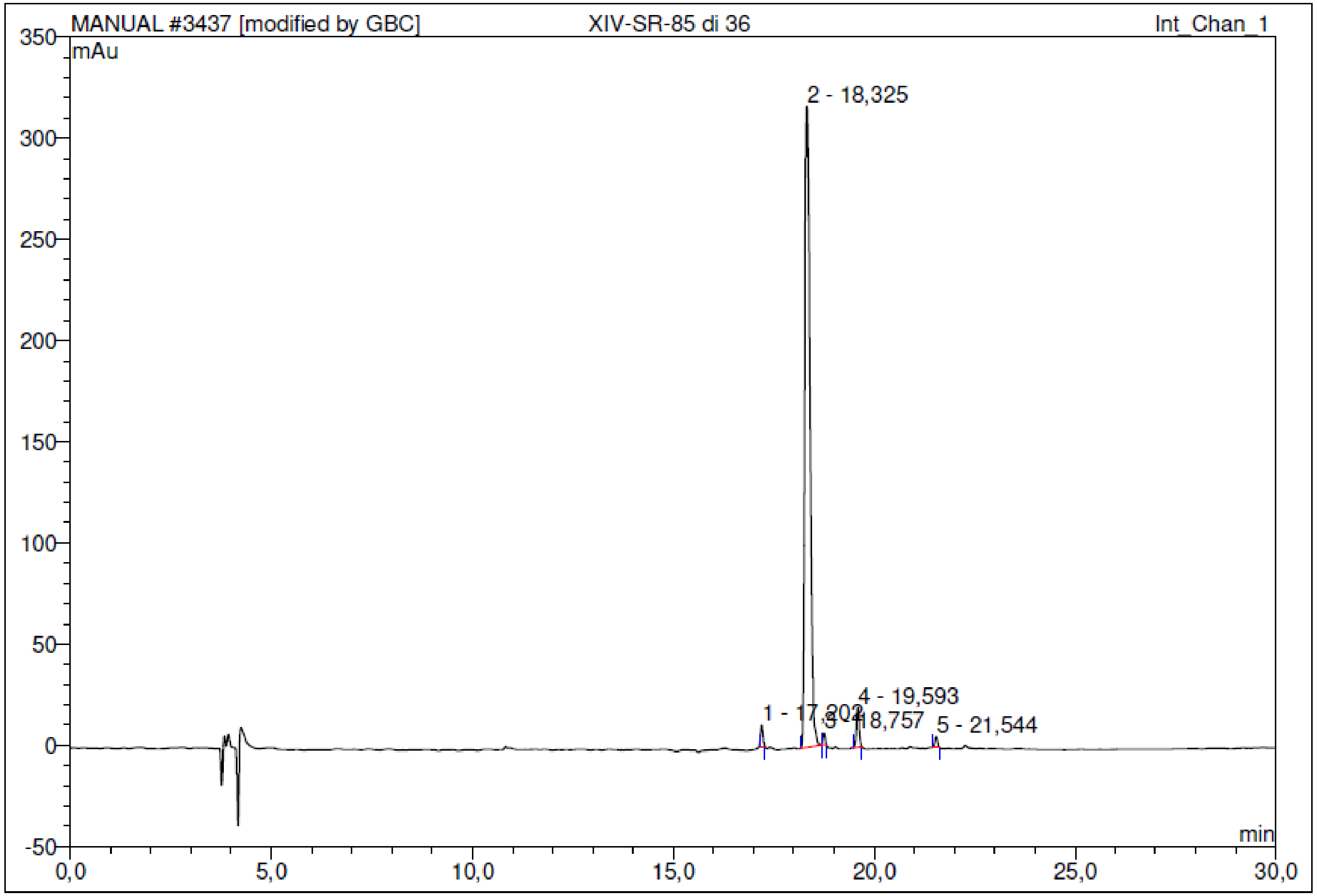

No.	Ret.Time min	Peak Name	Height mAu	Area mAu*min	Rel.Area %	Amount	Type
1	17,20	n.a.	10,502	0,755	1,54	n.a.	BMB*
2	18,32	n.a.	316,839	45,737	93,58	n.a.	BMB
3	18,76	n.a.	5,826	0,401	0,82	n.a.	BMB*
4	19,59	n.a.	19,708	1,552	3,18	n.a.	BMB*
5	21,54	n.a.	5,327	0,431	0,88	n.a.	BMB*
Total:			358,203	48,876	100,00	0,000	

Figure S111. HPLC analysis of **36**.

Spectrum Name: XIV-SR-44_36_pt
Start Ion: 100
End Ion: 1000
Source: APCI + 10.0µA 400C
Capillary: 150V 300C Offset: 25V Span: 0V

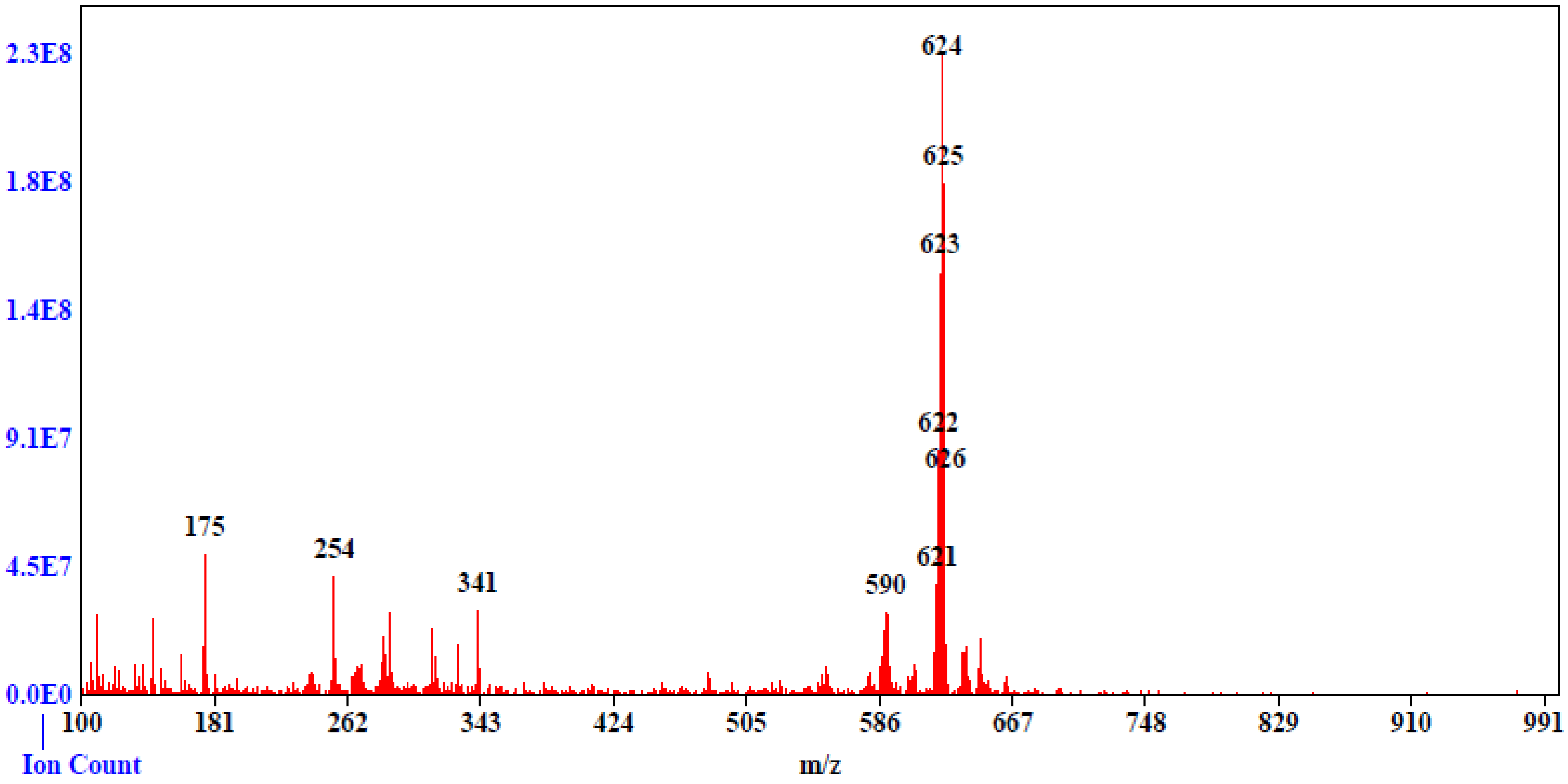

Figure S112. MS spectrum of **36**.

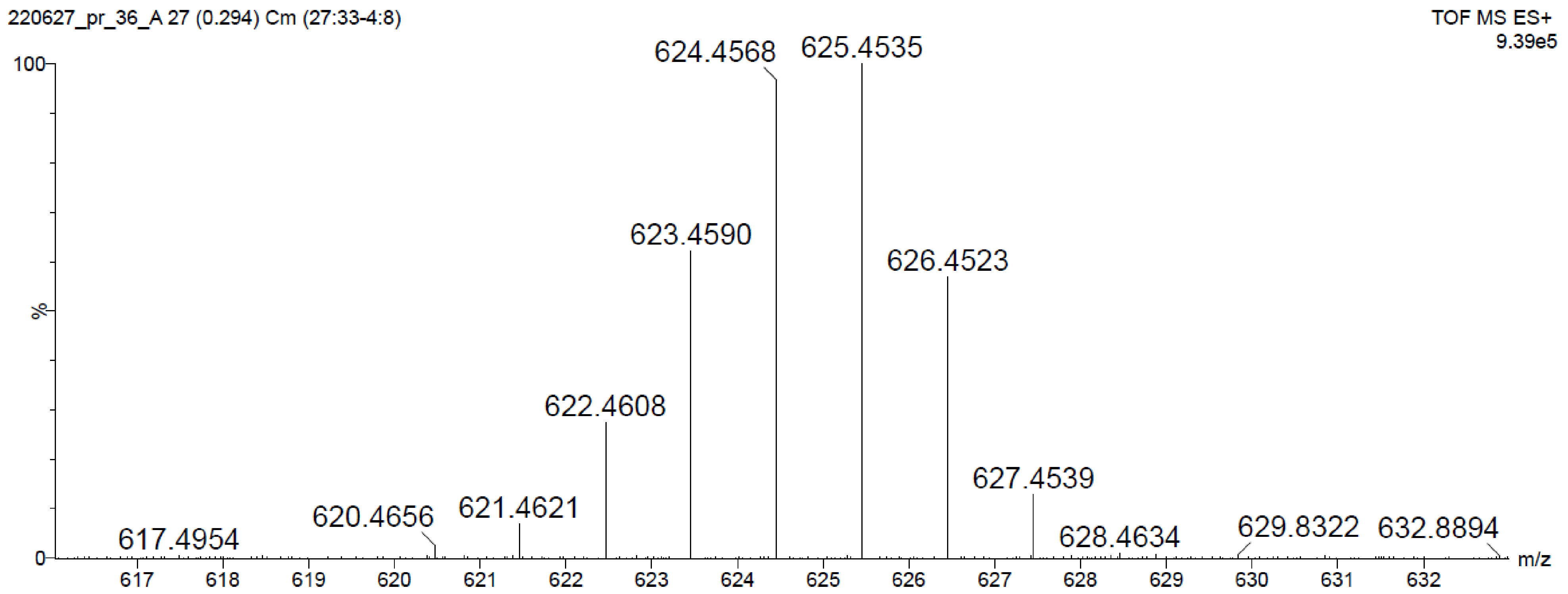

Figure S113. HRMS spectrum of **36**.

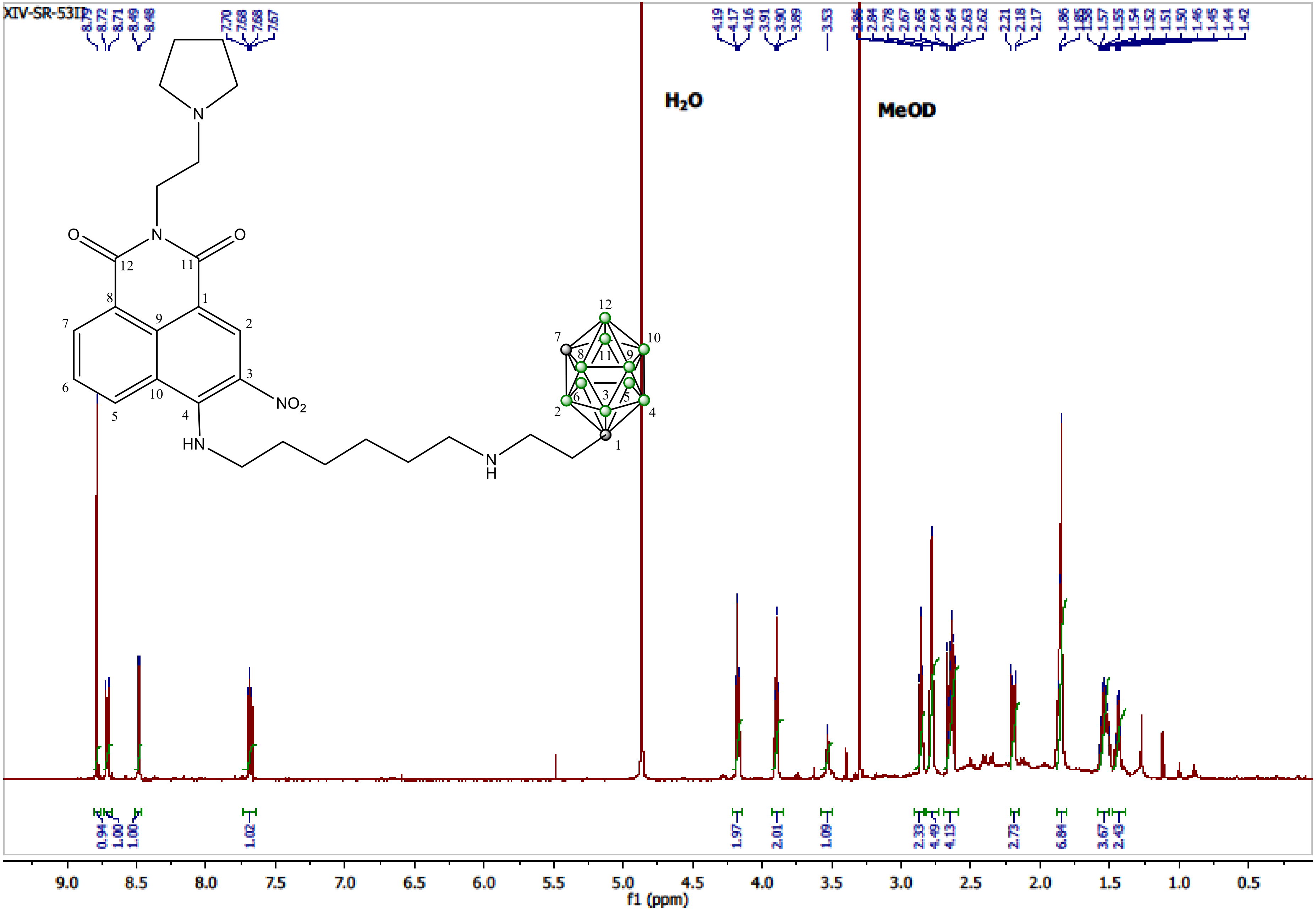

Figure S114. ^{1}H-NMR spectrum of **37**.

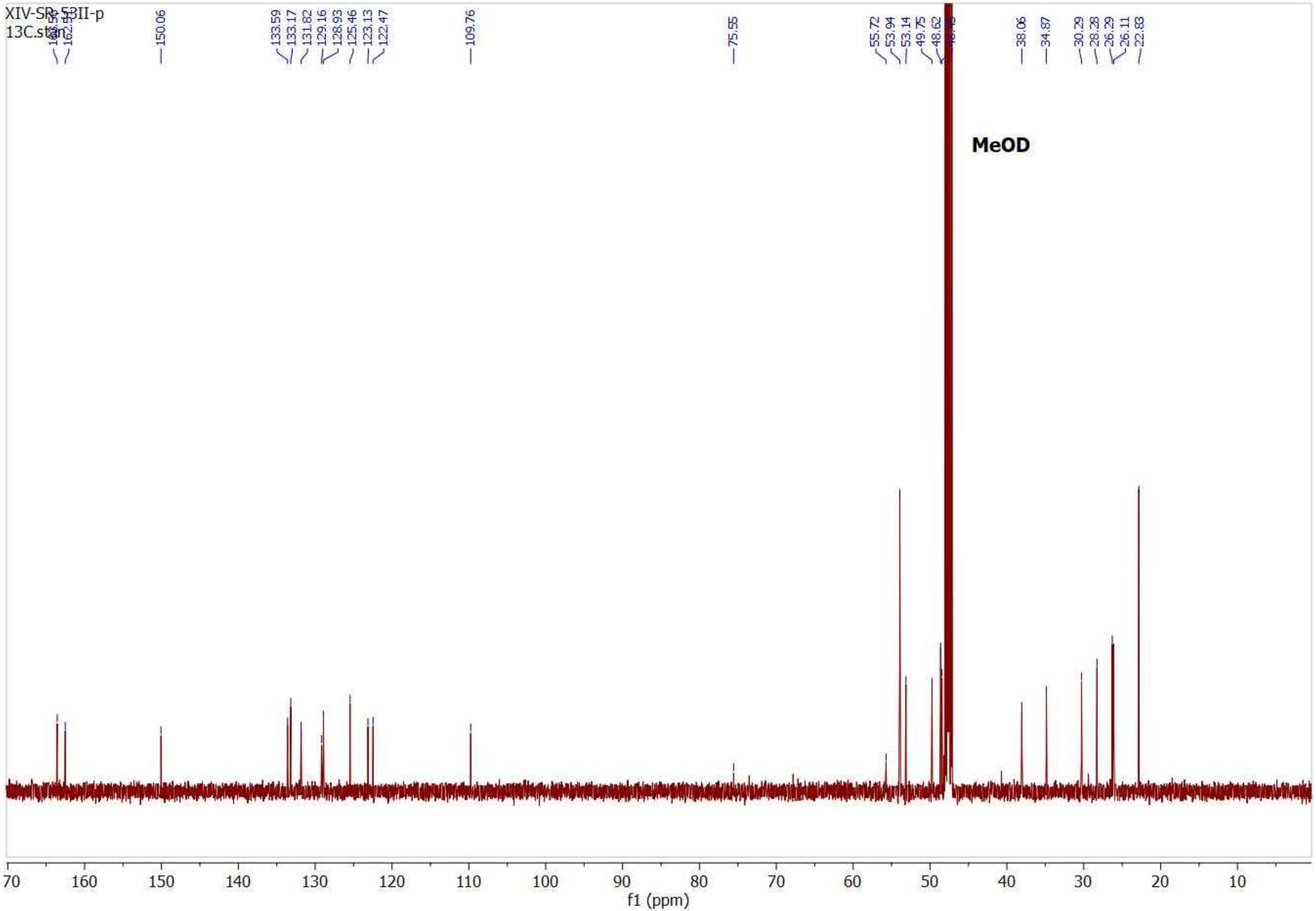

Figure S115. ^{13}C-NMR spectrum of **37**.

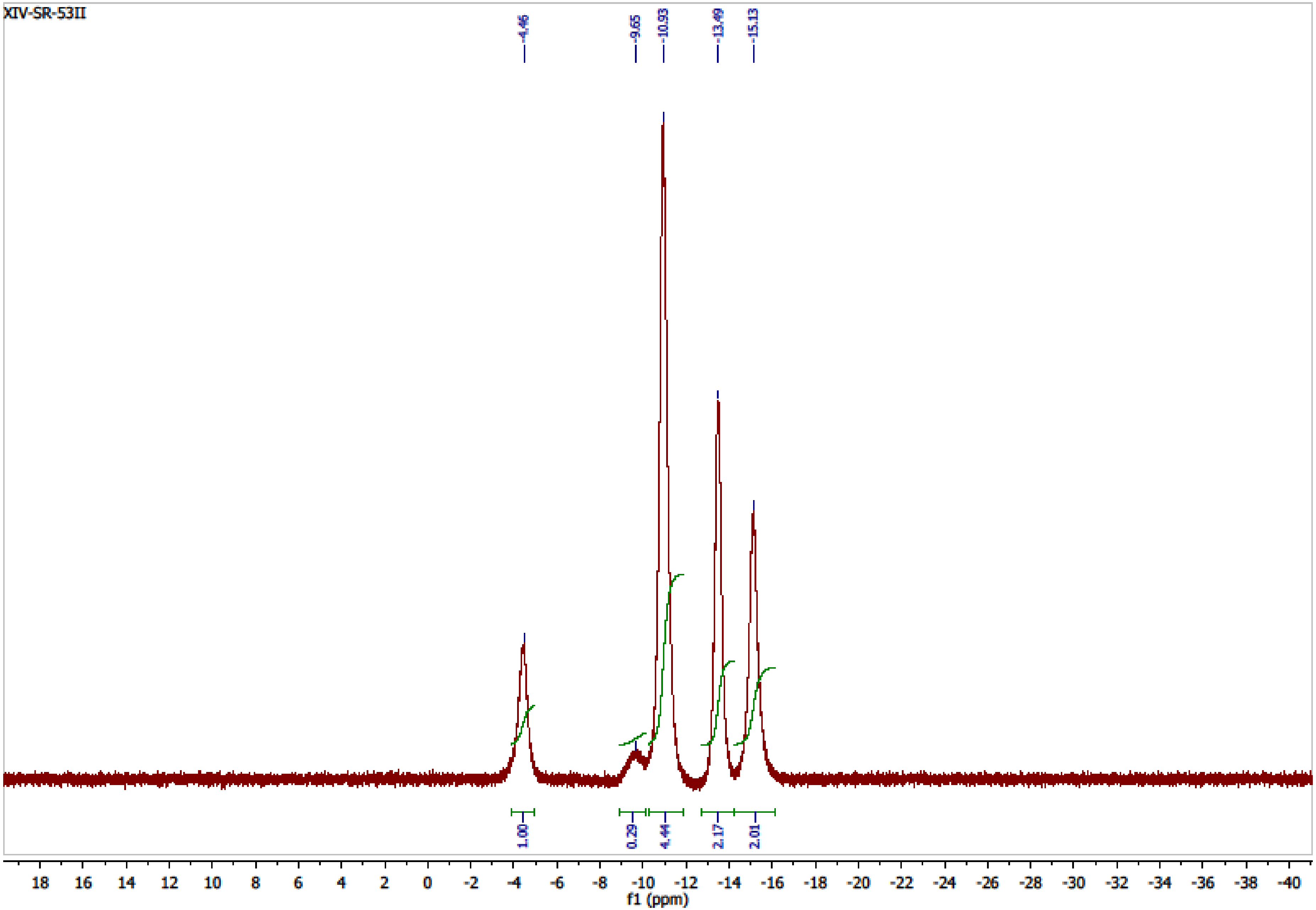

Figure S116. ^{11}B-NMR {^{1}H BB} spectrum of **37**.

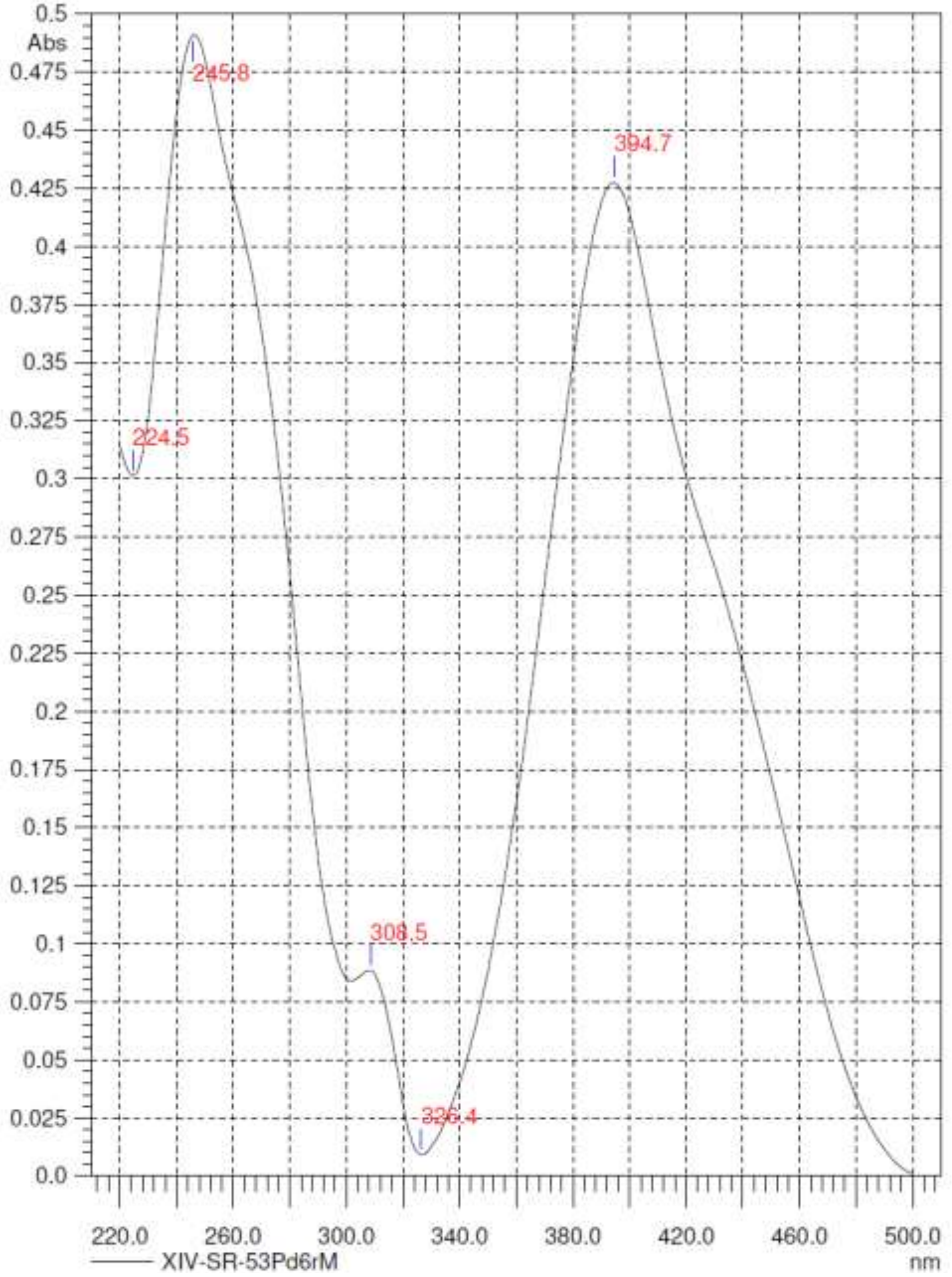

Figure S117. UV spectrum of **37**.

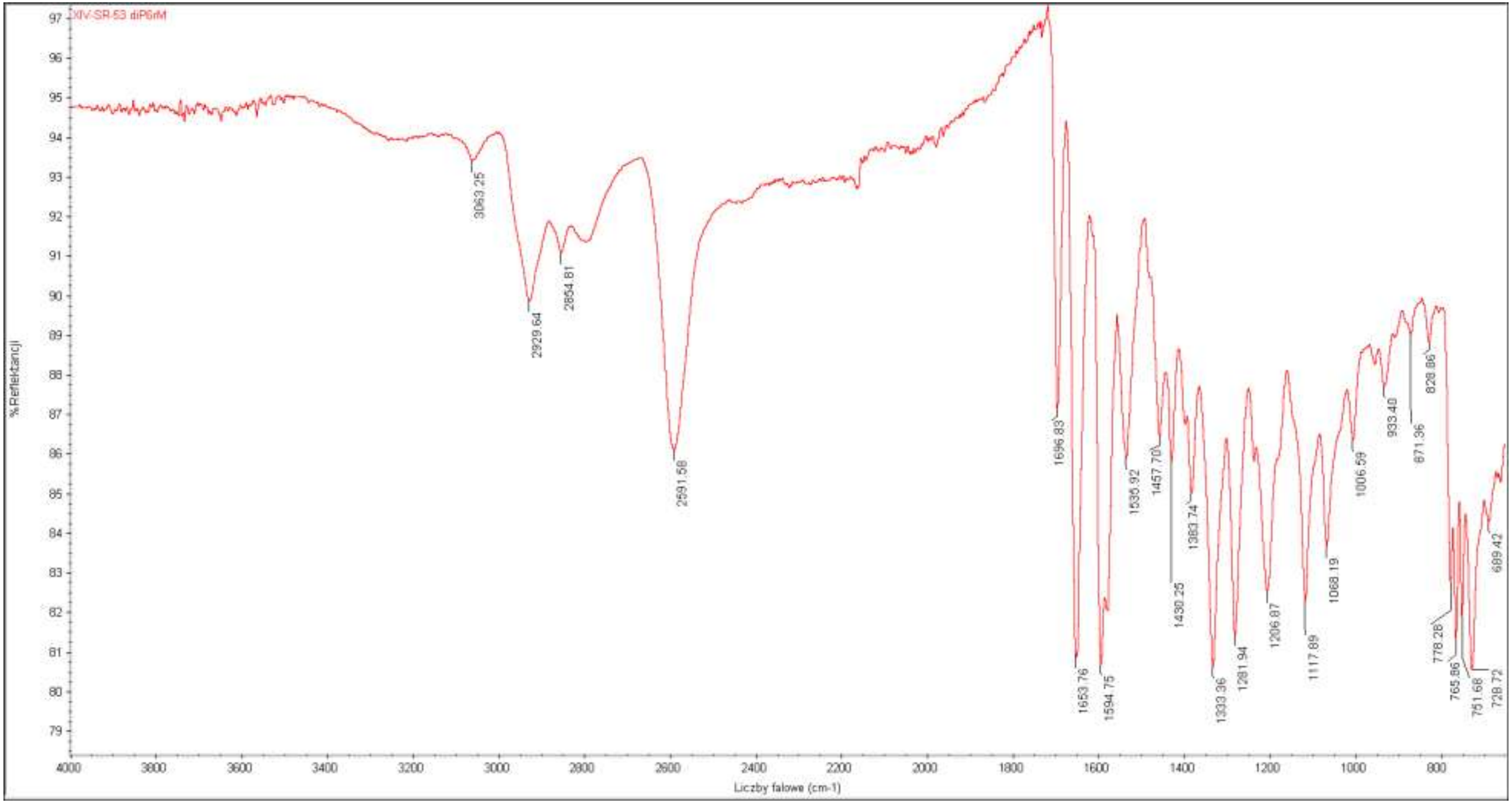

Figure S118. IR spectrum of **37**.

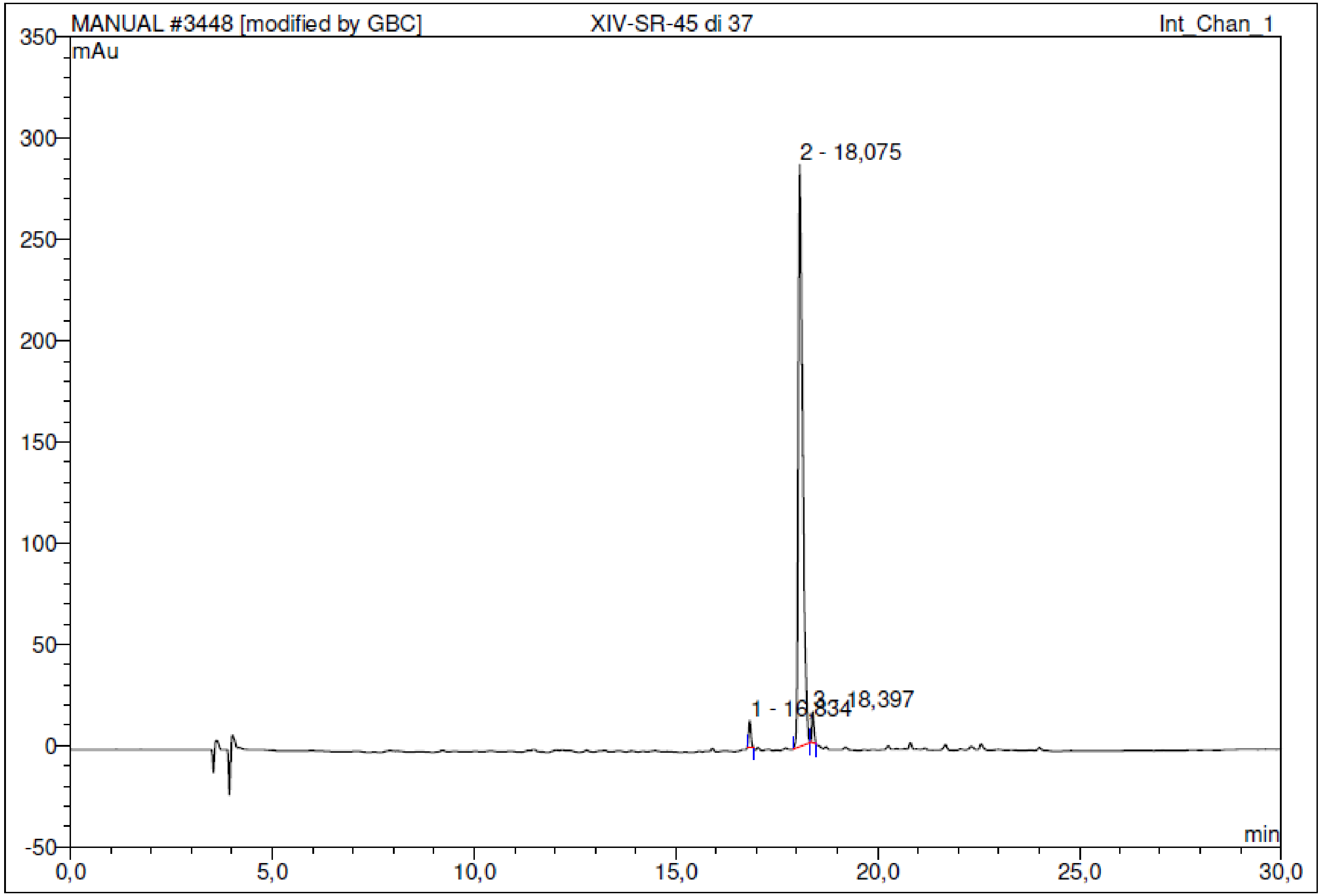

No.	Ret.Time min	Peak Name	Height mAu	Area mAu*min	Rel.Area %	Amount	Type
1	16,83	n.a.	13,356	0,935	2,40	n.a.	BMB*
2	18,08	n.a.	287,392	36,992	95,00	n.a.	BMB
3	18,40	n.a.	15,244	1,010	2,60	n.a.	BMB*
Total:			315,992	38,938	100,00	0,000	

Figure S119. HPLC analysis of **37**.

Spectrum Name: XIV-SR-45_37_pt
Start Ion: 100
End Ion: 1000
Source: APCI + 10.0μA 400C
Capillary: 150V 300C Offset: 25V Span: 0V

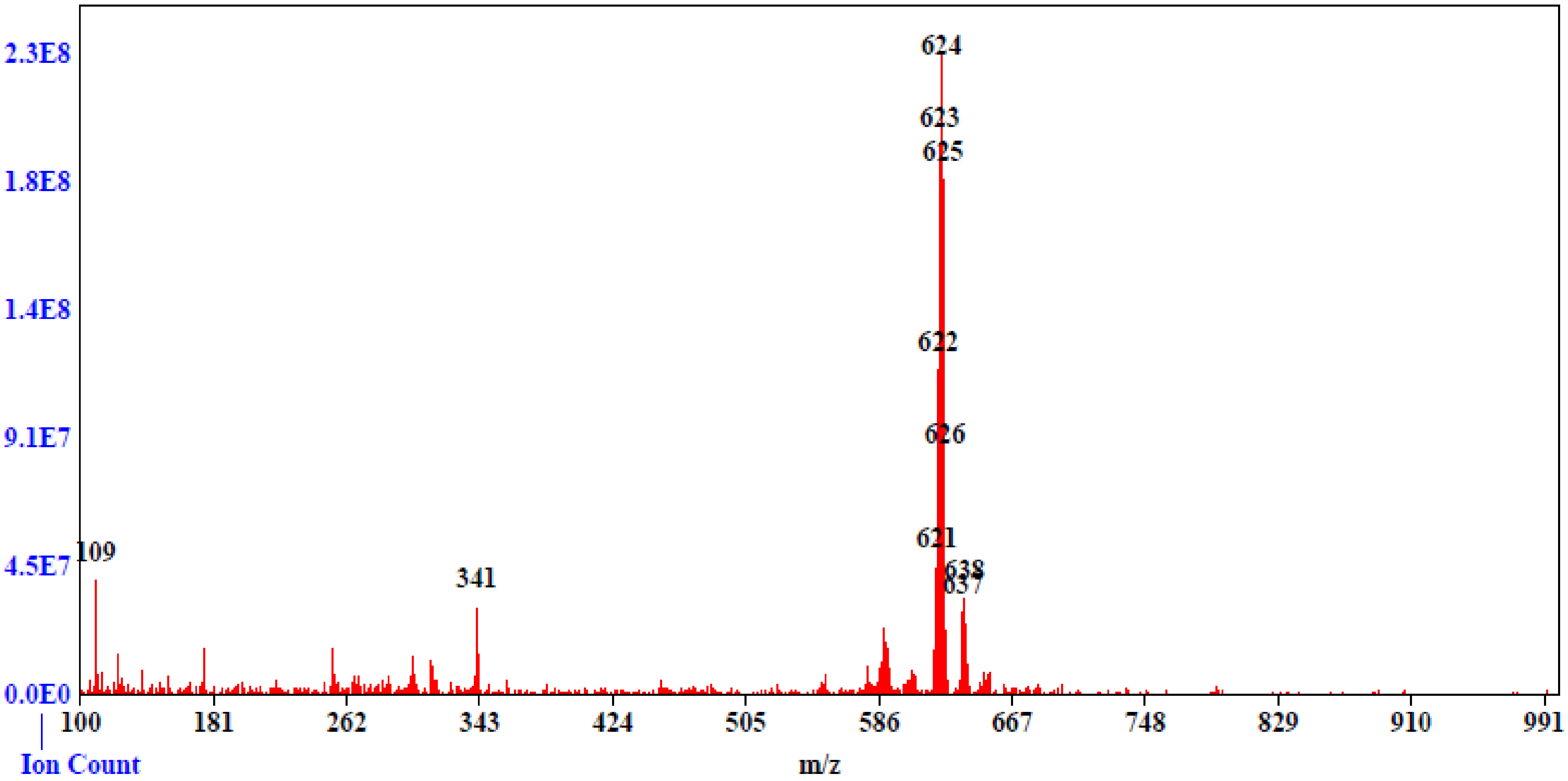

Figure S120. MS spectrum of **37**.

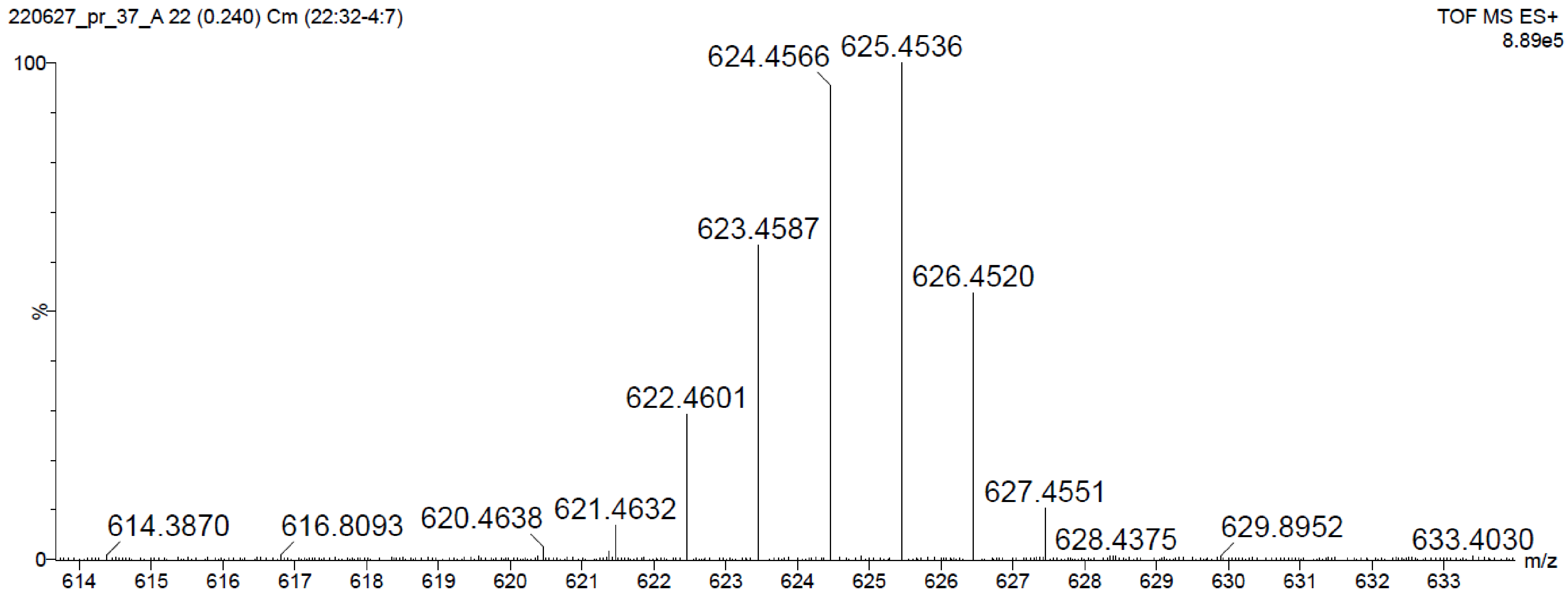

Figure S121. HRMS spectrum of **37**.

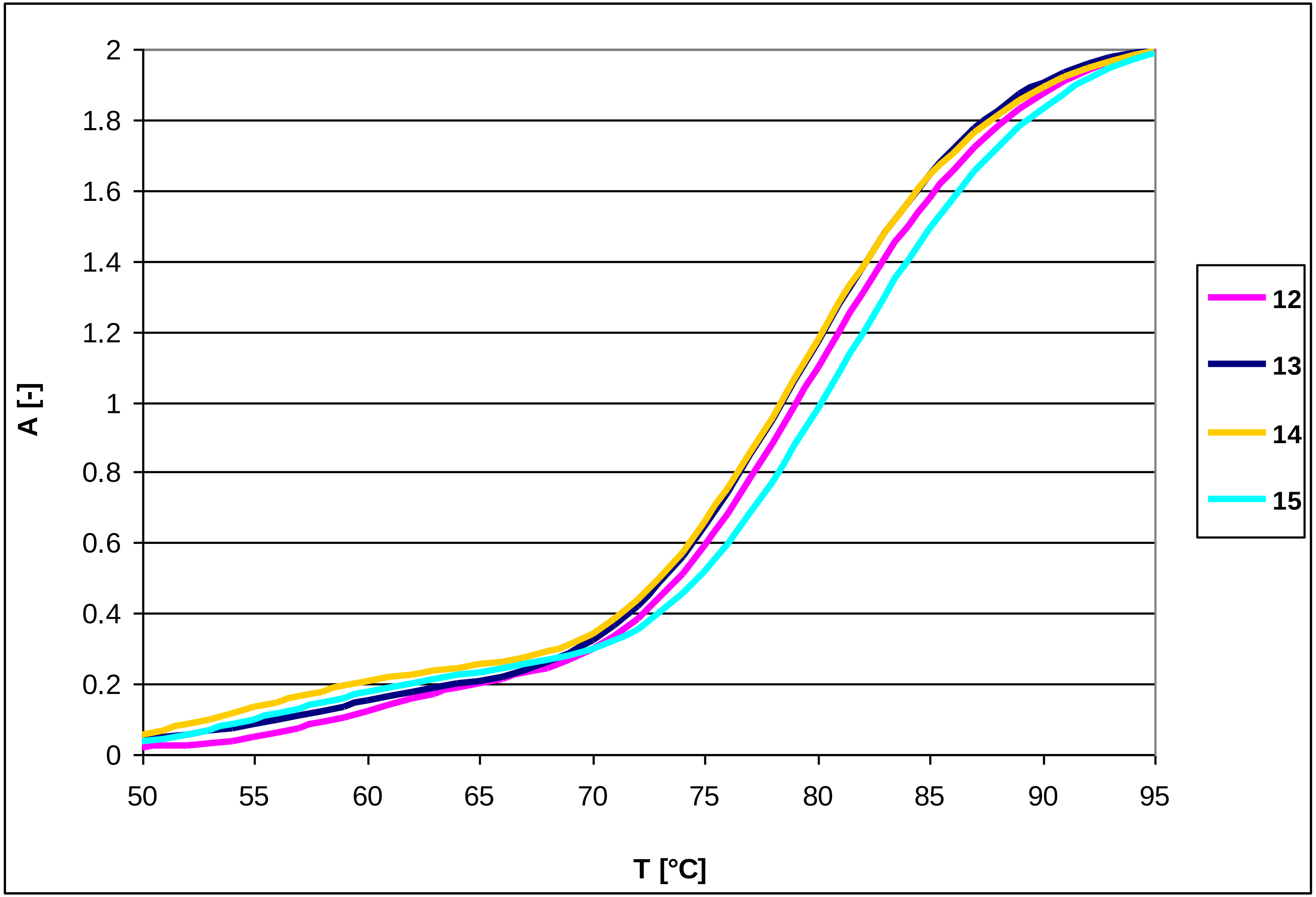

Figure S122. Melting curves of ct-DNA upon addition of **12-15** (c (ct-DNA) = 2 × 10^{-5} mol dm^{-3}) at molar ratio r = 0.3 (r = [compound]/[ct-DNA]), sodium cacodylate buffer (pH 7.0, 20 mM).

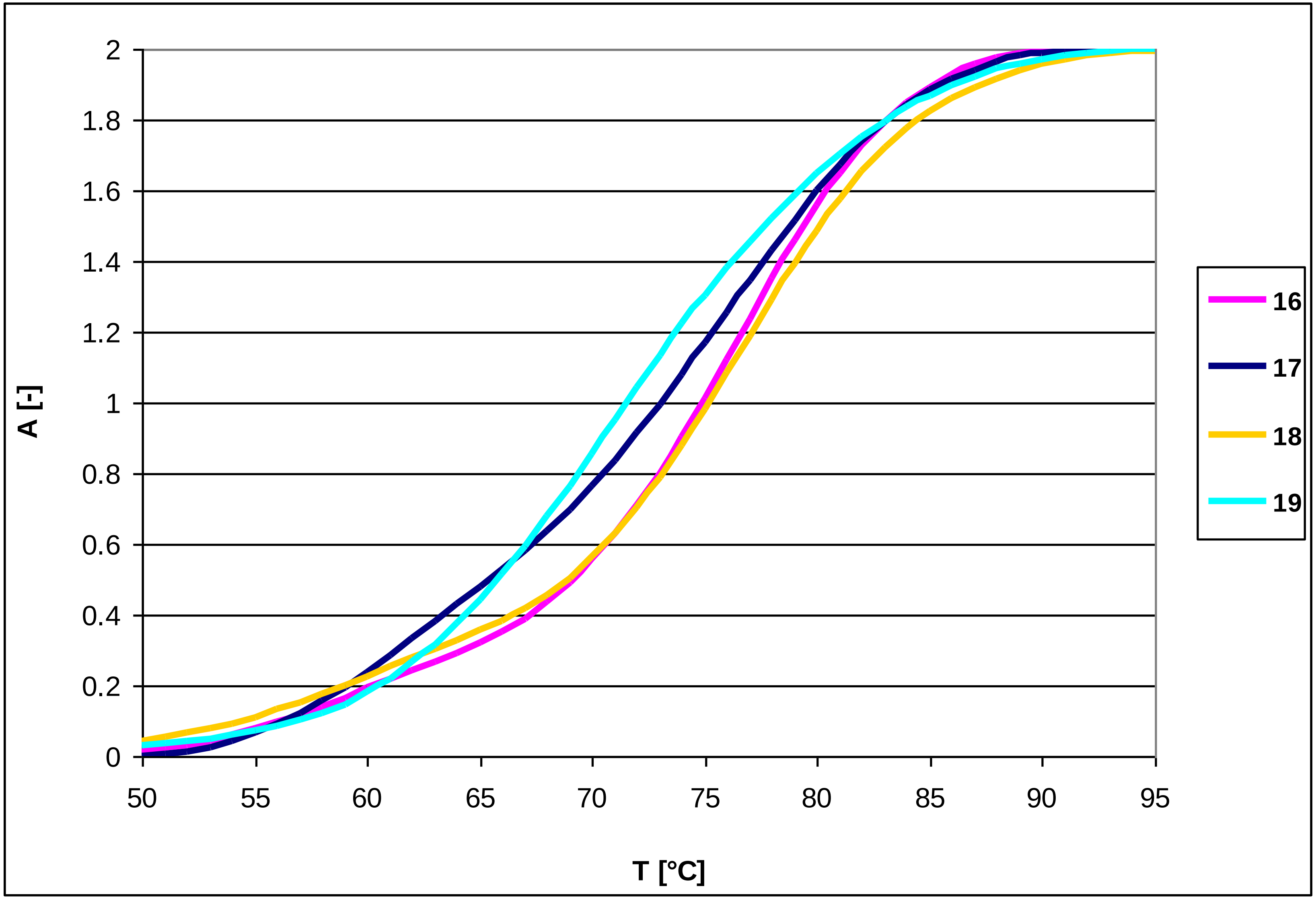

Figure S123. Melting curves of ct-DNA upon addition of **16-19** (c (ct-DNA) = 2 × 10^{-5} mol dm^{-3}) at molar ratio r = 0.3 (r = [compound]/[ct-DNA]), sodium cacodylate buffer (pH 7.0, 20 mM).

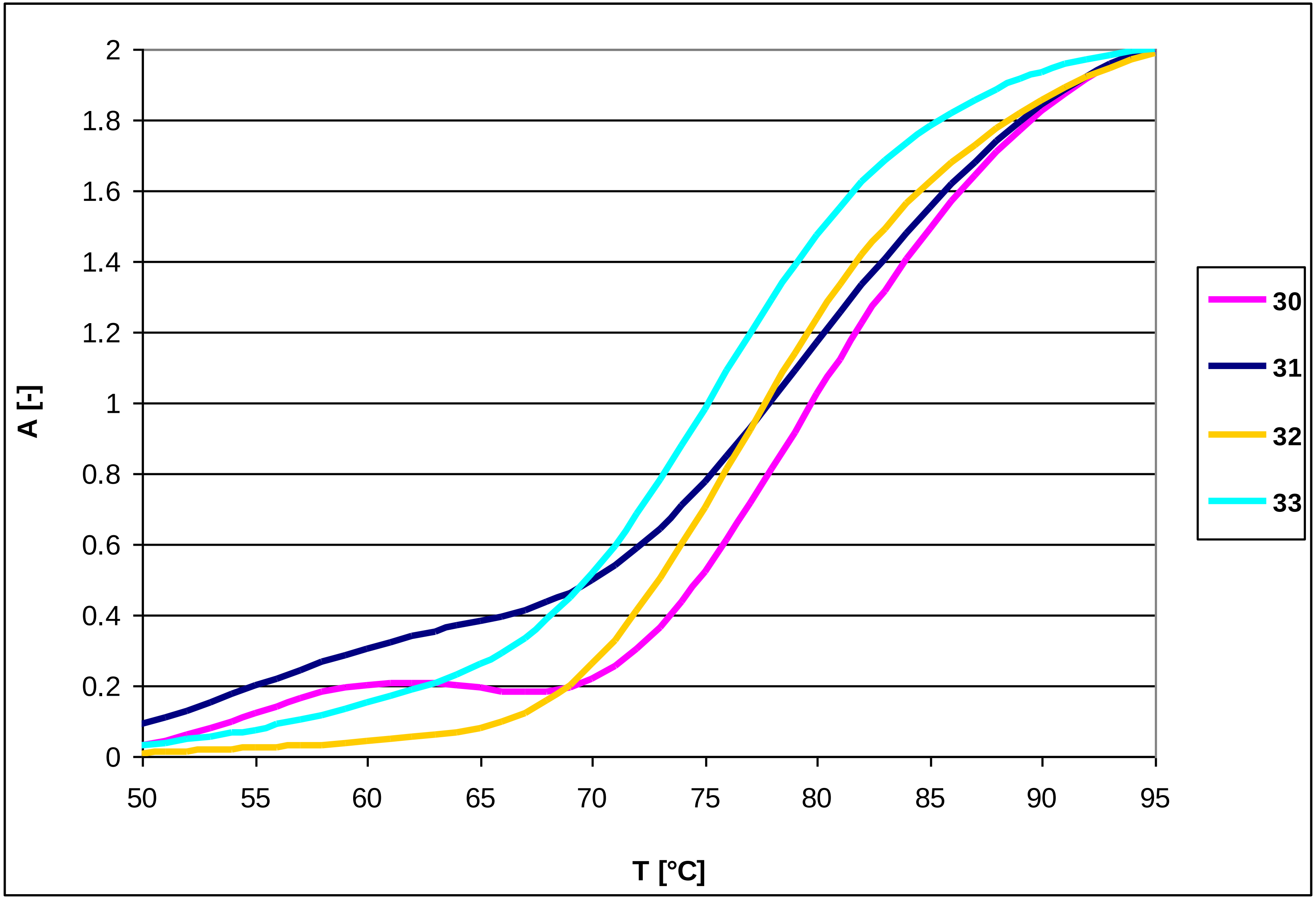

Figure S124. Melting curves of ct-DNA upon addition of **30-33** (c (ct-DNA) = 2 × 10^{-5} mol dm^{-3}) at molar ratio r = 0.3 (r = [compound]/[ct-DNA]), sodium cacodylate buffer (pH 7.0, 20 mM).

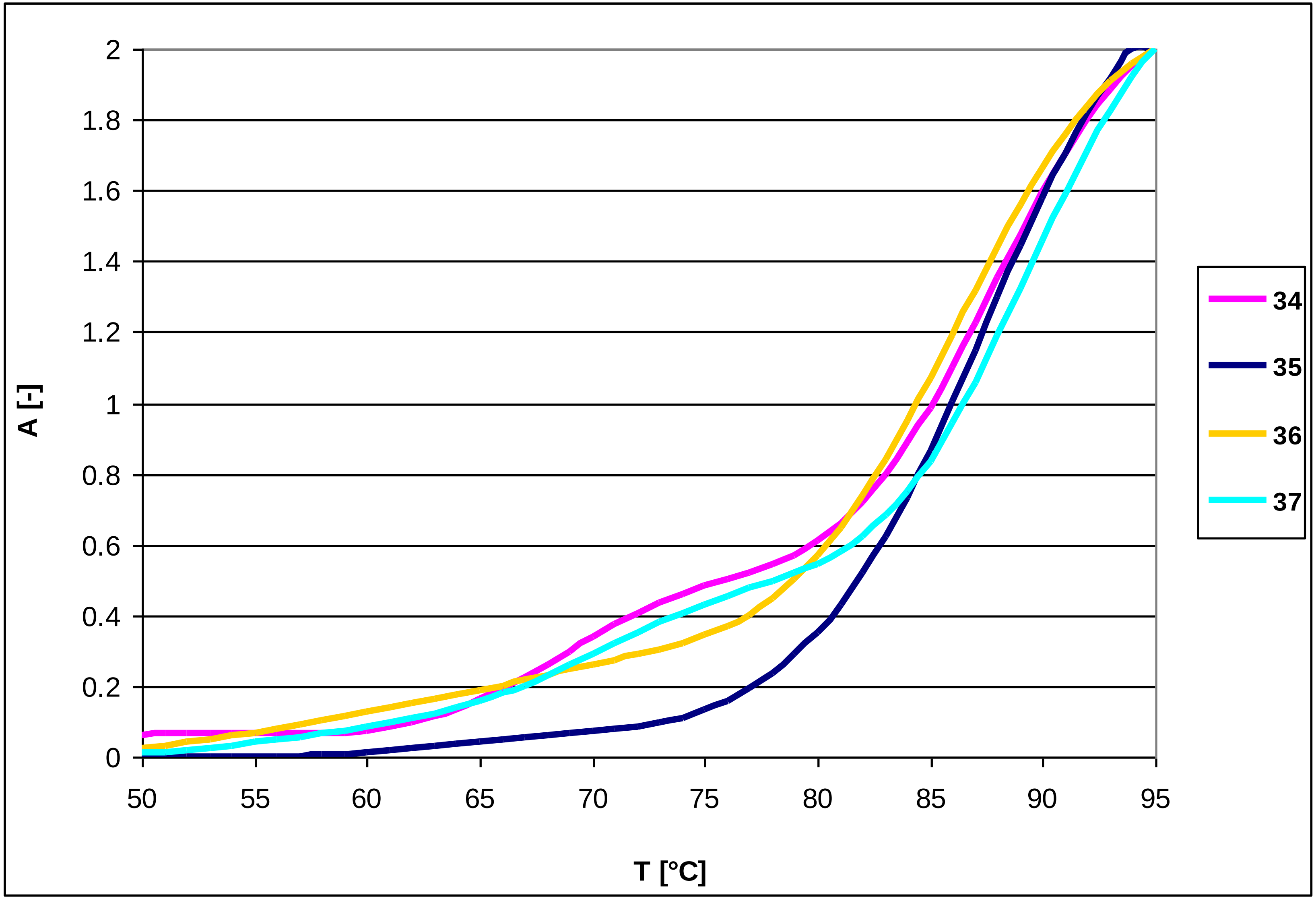

Figure S125. Melting curves of ct-DNA upon addition of **34-37** (c (ct-DNA) = 2×10^{-5} mol dm^{-3}) at molar ratio r = 0.3 (r = [compound]/[ct-DNA]), sodium cacodylate buffer (pH 7.0, 20 mM).

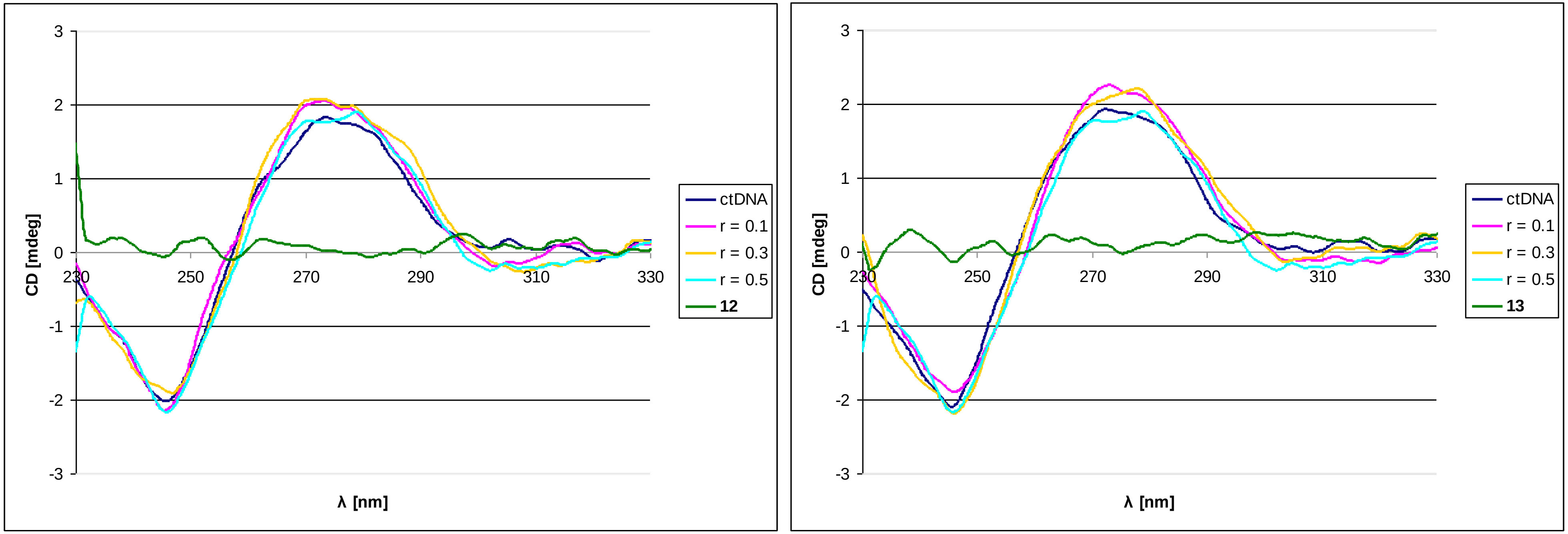

Figure S126. Changes in the CD spectrum of ct-DNA upon addition of **12** (c (ct-DNA) = 4 × 10^{-5} mol dm^{-3}) (left) and **13** (c (ct-DNA) = 4 × 10^{-5} mol dm^{-3}) (right) at different molar ratios r = [compound]/[ct-DNA], sodium cacodylate buffer (pH 7.0, 20 mM).

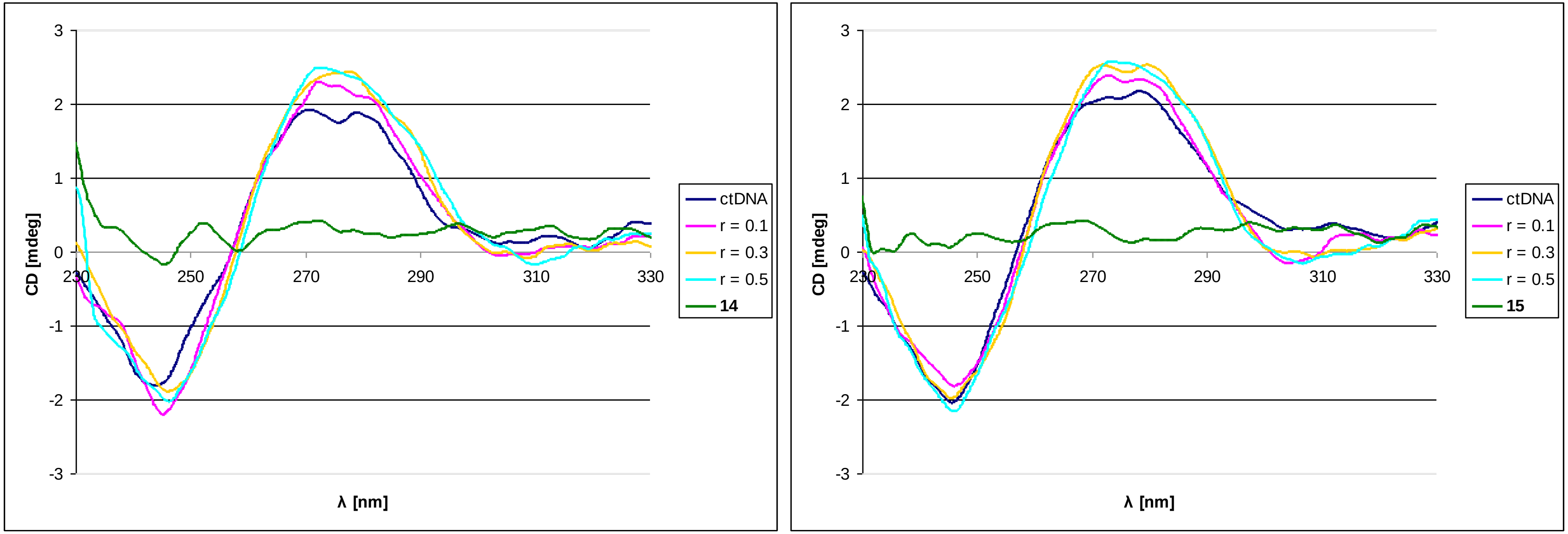

Figure S127. Changes in the CD spectrum of ct-DNA upon addition of **14** (c (ct-DNA) = 4 × 10^{-5} mol dm^{-3}) (left) and **15** (c (ct-DNA) = 4 × 10^{-5} mol dm^{-3}) (right) at different molar ratios r = [compound]/[ct-DNA], sodium cacodylate buffer (pH 7.0, 20 mM).

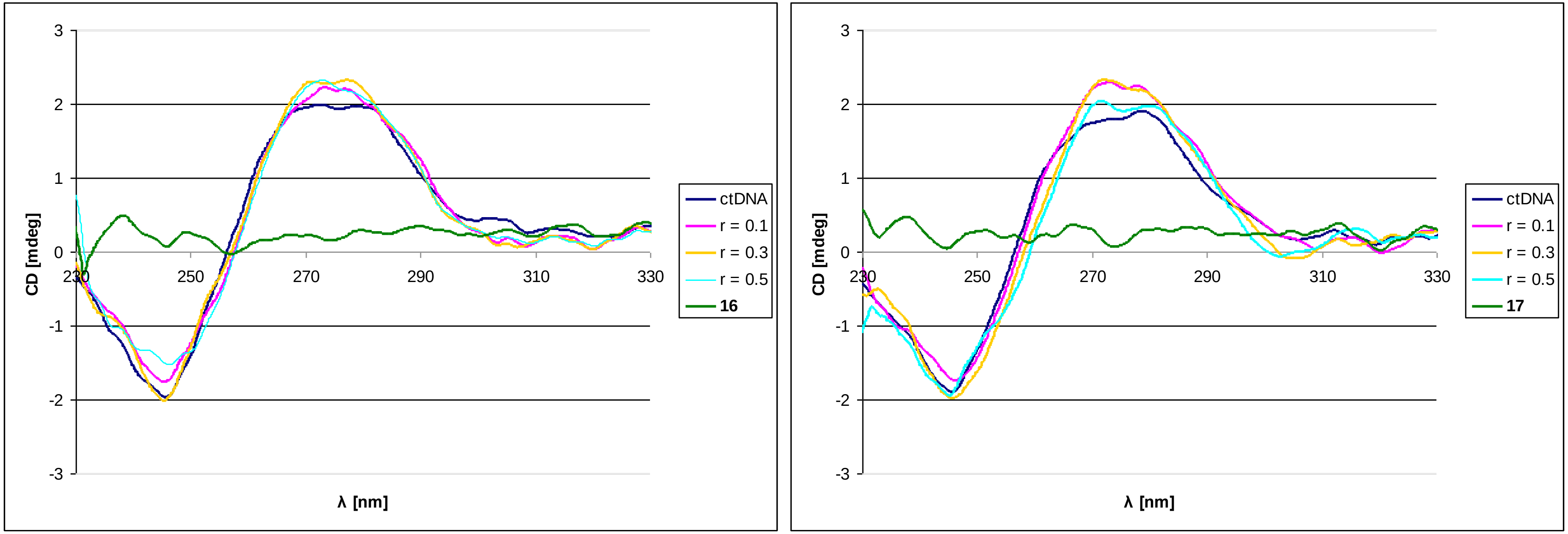

Figure S128. Changes in the CD spectrum of ct-DNA upon addition of **16** (c (ct-DNA) = 4 × 10^{-5} mol dm^{-3}) (left) and **17** (c (ct-DNA) = 4 × 10^{-5} mol dm^{-3}) (right) at different molar ratios r = [compound]/[ct-DNA], sodium cacodylate buffer (pH 7.0, 20 mM).

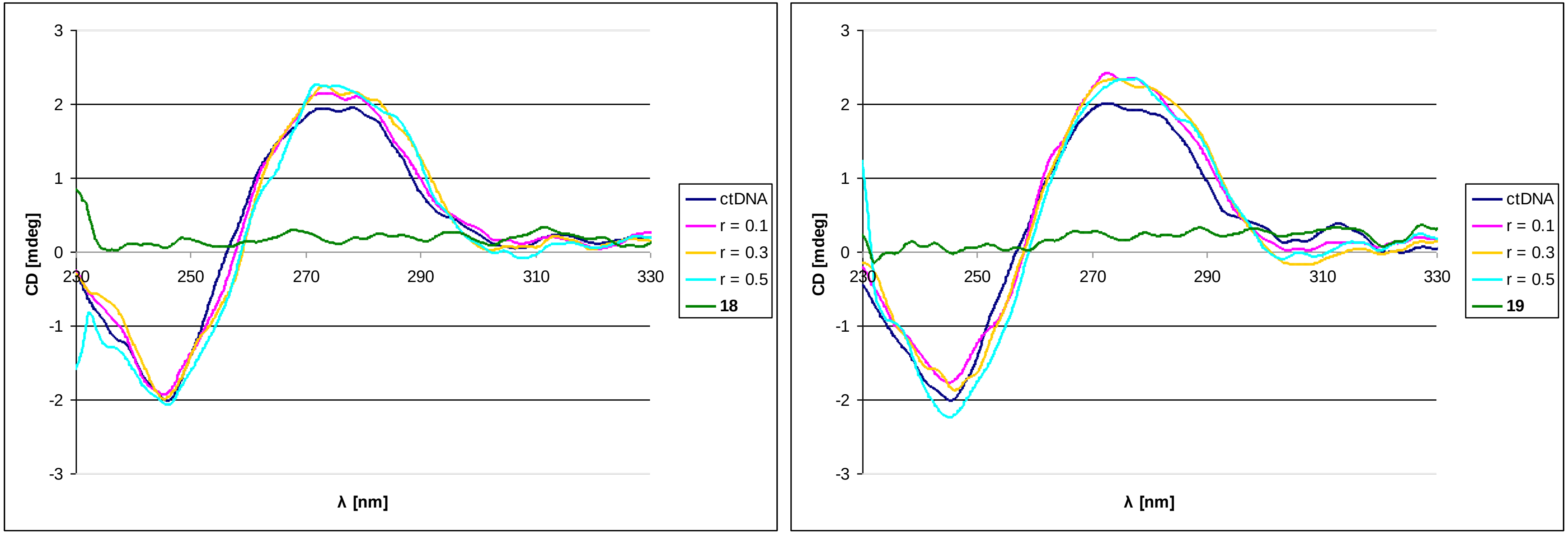

Figure S129. Changes in the CD spectrum of ct-DNA upon addition of **18** (c (ct-DNA) = 4 × 10^{-5} mol dm^{-3}) (left) and **19** (c (ct-DNA) = 4 × 10^{-5} mol dm^{-3}) (right) at different molar ratios r = [compound]/[ct-DNA], sodium cacodylate buffer (pH 7.0, 20 mM).

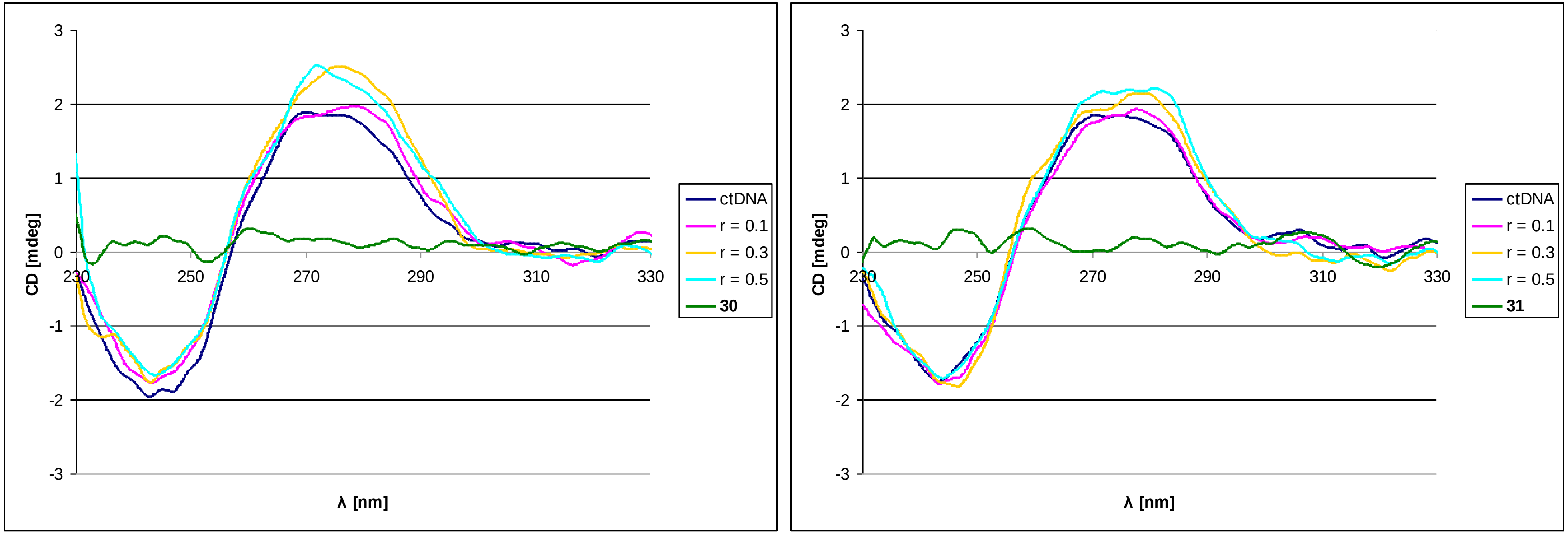

Figure S130. Changes in the CD spectrum of ct-DNA upon addition of **30** (c (ct-DNA) = 4 × 10^{-5} mol dm^{-3}) (left) and **31** (c (ct-DNA) = 4 × 10^{-5} mol dm^{-3}) (right) at different molar ratios r = [compound]/[ct-DNA], sodium cacodylate buffer (pH 7.0, 20 mM).

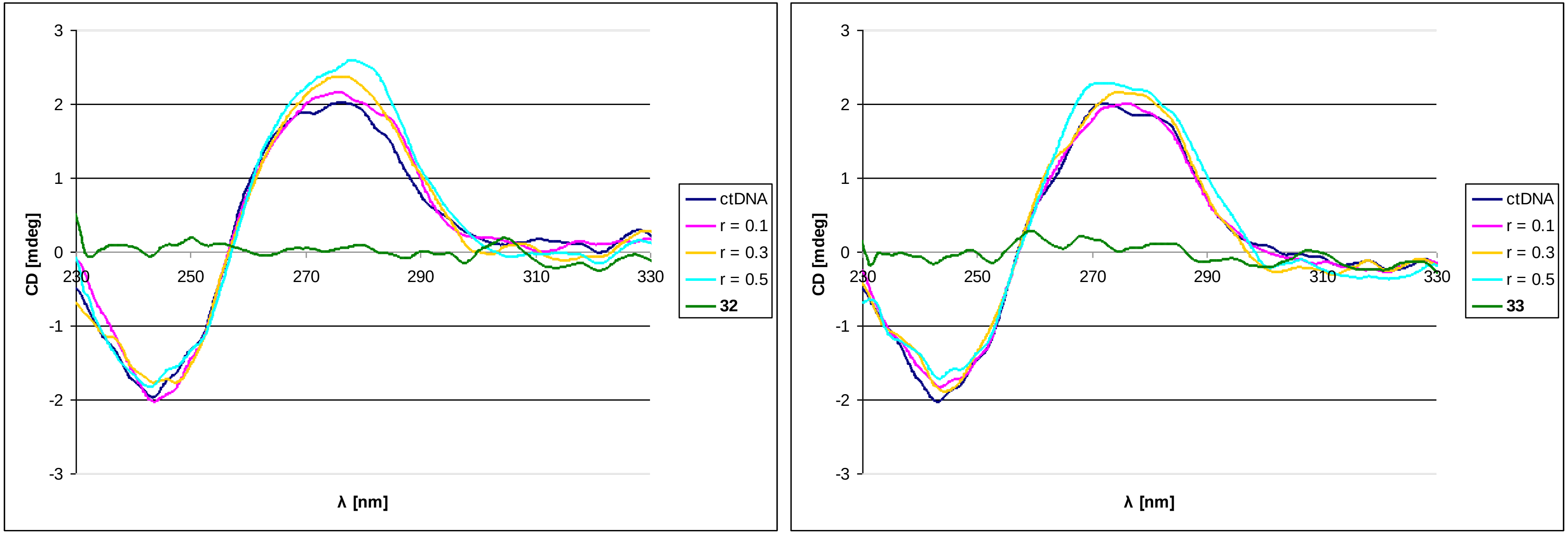

Figure S131. Changes in the CD spectrum of ct-DNA upon addition of **32** (c (ct-DNA) = 4 × 10^{-5} mol dm^{-3}) (left) and **33** (c (ct-DNA) = 4 × 10^{-5} mol dm^{-3}) (right) at different molar ratios r = [compound]/[ct-DNA], sodium cacodylate buffer (pH 7.0, 20 mM).

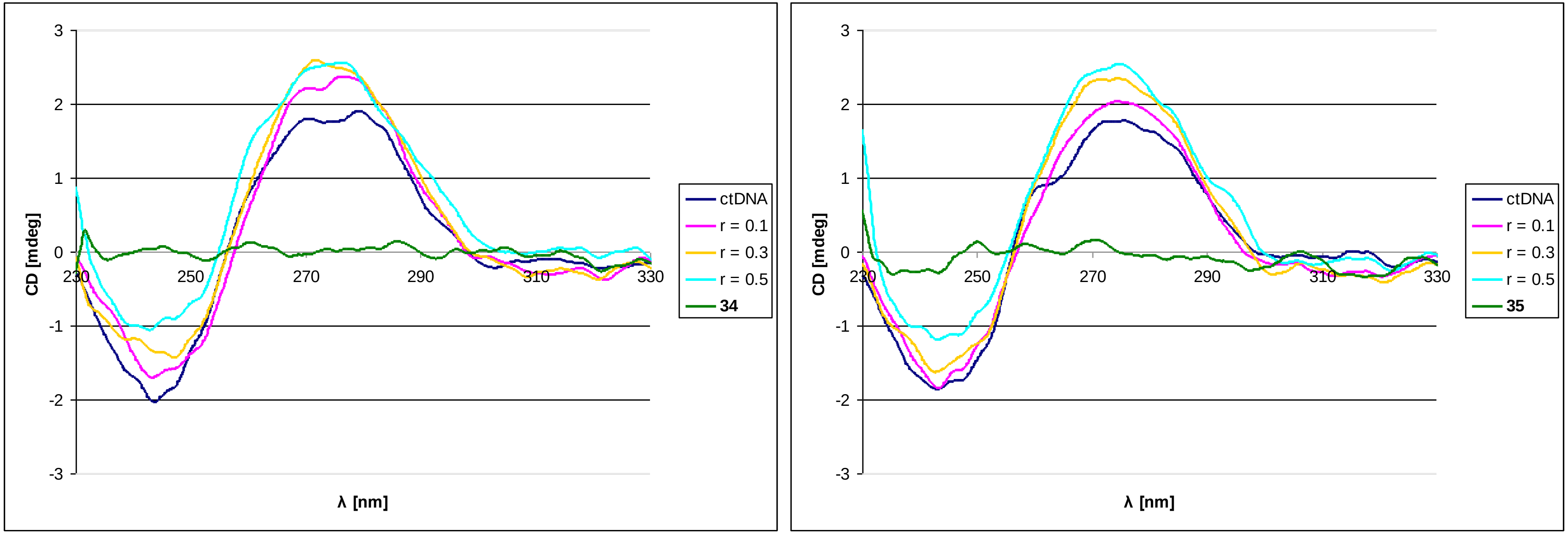

Figure S132. Changes in the CD spectrum of ct-DNA upon addition of **34** (c (ct-DNA) = 4 × 10^{-5} mol dm^{-3}) (left) and **35** (c (ct-DNA) = 4 × 10^{-5} mol dm^{-3}) (right) at different molar ratios r = [compound]/[ct-DNA], sodium cacodylate buffer (pH 7.0, 20 mM).

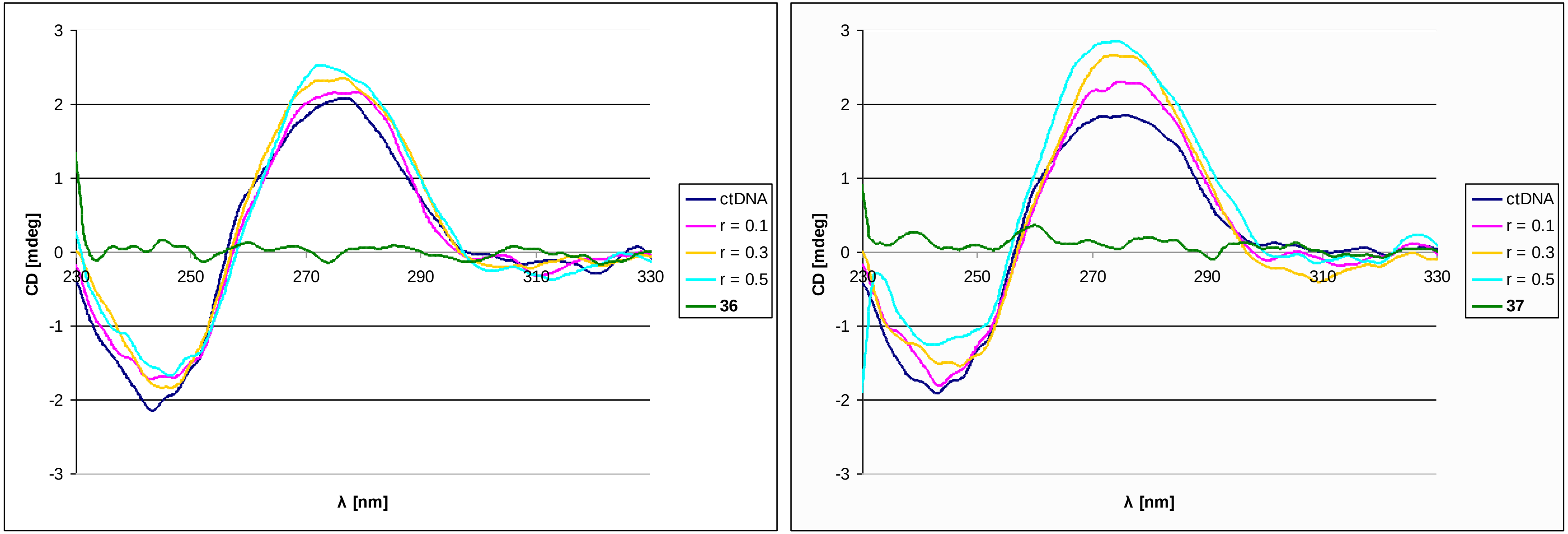

Figure S133. Changes in the CD spectrum of ct-DNA upon addition of **36** (c (ct-DNA) = 4×10^{-5} mol dm^{-3}) (left) and **37** (c (ct-DNA) = 4×10^{-5} mol dm^{-3}) (right) at different molar ratios r = [compound]/[ct-DNA], sodium cacodylate buffer (pH 7.0, 20 mM).

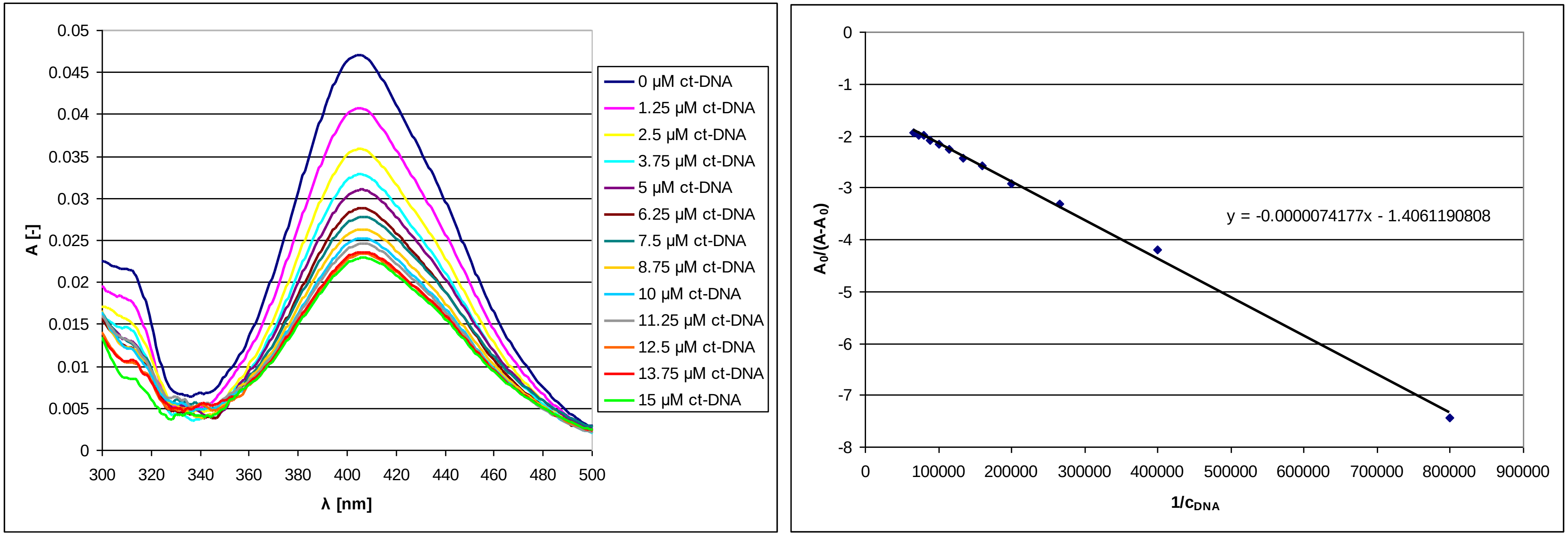

Figure S134. UV-vis absorption spectra of compound **12** (10 µM) in the presence of increasing amount of cf-DNA (0-15 µM) (left). The plot of A_0/A-A_0 versus 1/[DNA] yielded the binding constant (right).

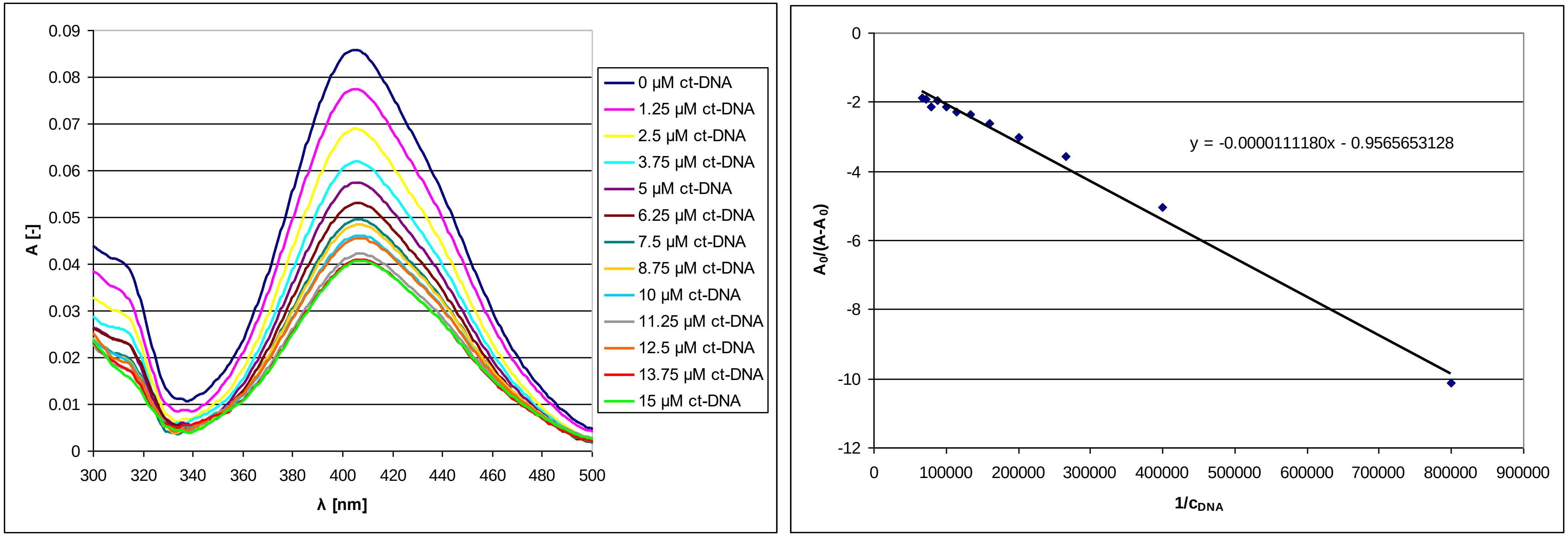

Figure S135. UV-vis absorption spectra of compound **13** (10 µM) in the presence of increasing amount of ct-DNA (0-15 µM) (left). The plot of $A_0/A-A_0$ versus 1/[DNA] yielded the binding constant (right).

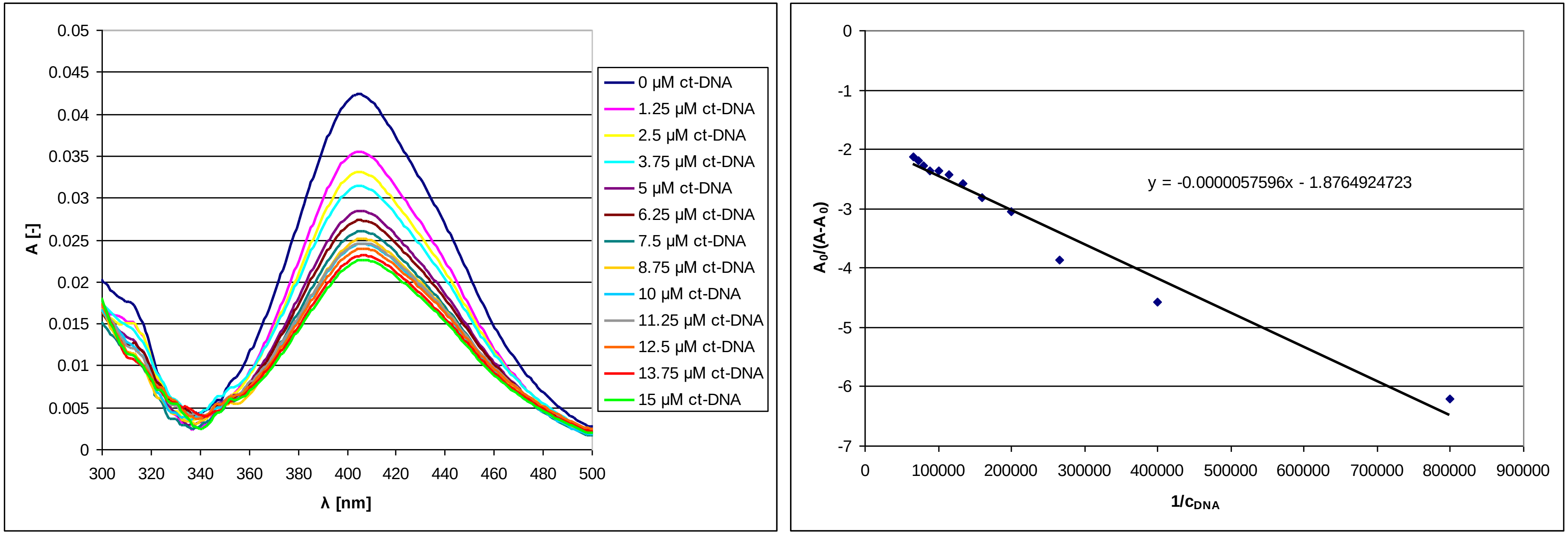

Figure S136. UV-vis absorption spectra of compound **14** (10 µM) in the presence of increasing amount of ct-DNA (0-15 µM) (left). The plot of A_0/A-A_0 versus 1/[DNA] yielded the binding constant (right).

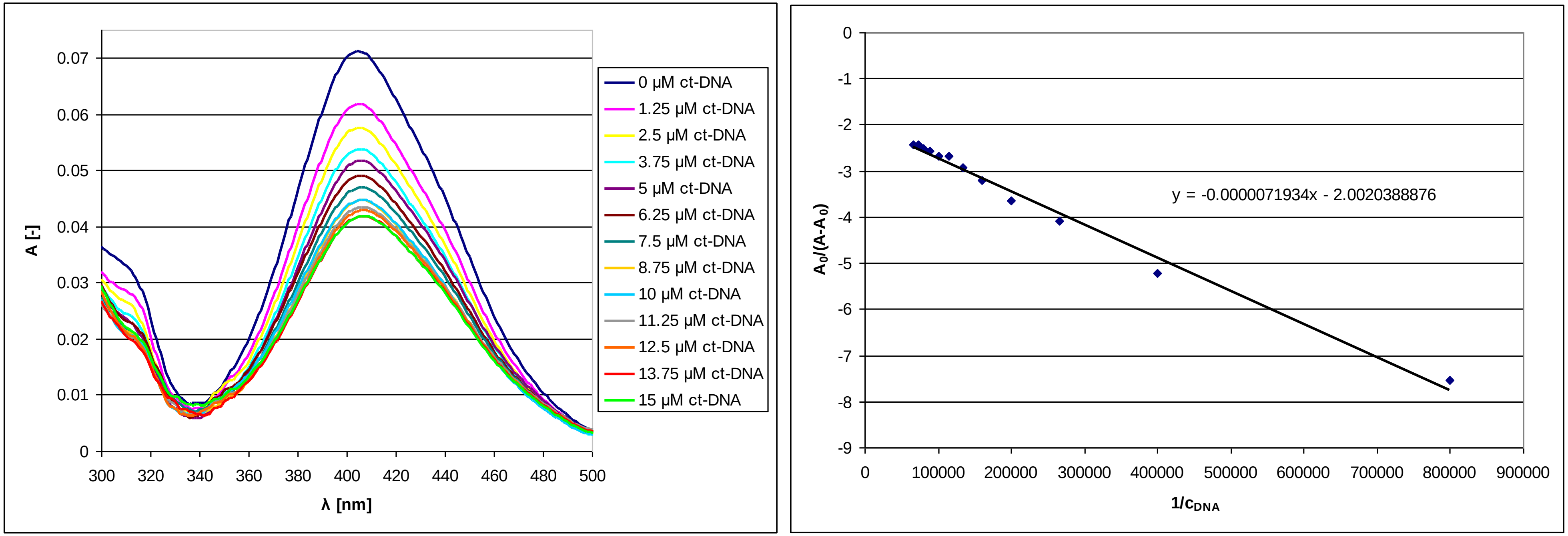

Figure S137. UV-vis absorption spectra of compound **15** (10 μM) in the presence of increasing amount of ct-DNA (0-15 μM) (left). The plot of $A_0/A-A_0$ versus 1/[DNA] yielded the binding constant (right).

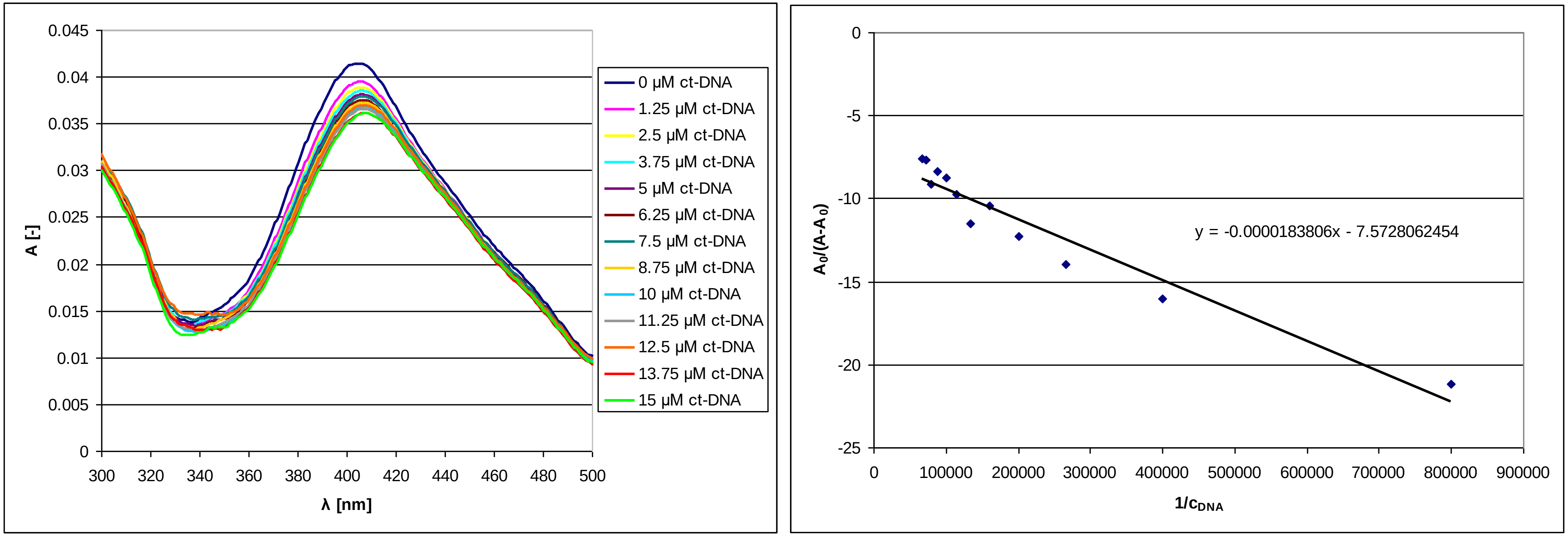

Figure S138. UV-vis absorption spectra of compound **16** (10 μM) in the presence of increasing amount of ct-DNA (0-15 μM) (left). The plot of $A_0/A-A_0$ versus 1/[DNA] yielded the binding constant (right).

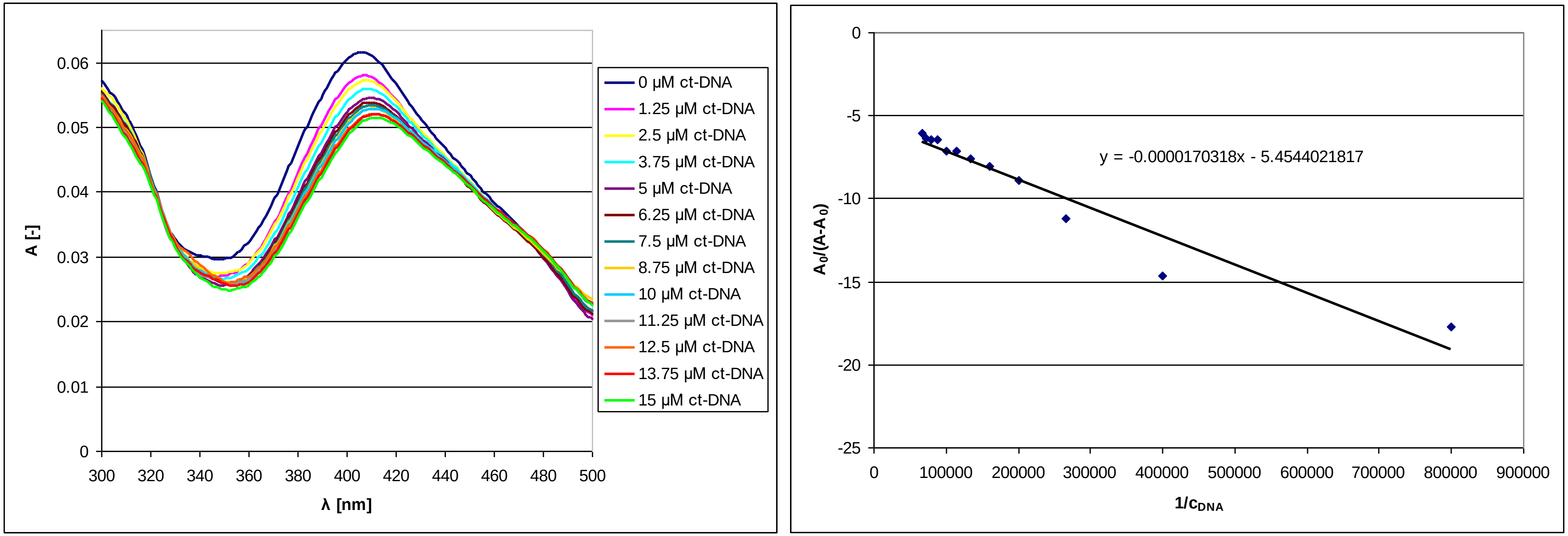

Figure S139. UV-vis absorption spectra of compound **17** (10 µM) in the presence of increasing amount of ct-DNA (0-15 µM) (left). The plot of $A_0/A-A_0$ versus 1/[DNA] yielded the binding constant (right).

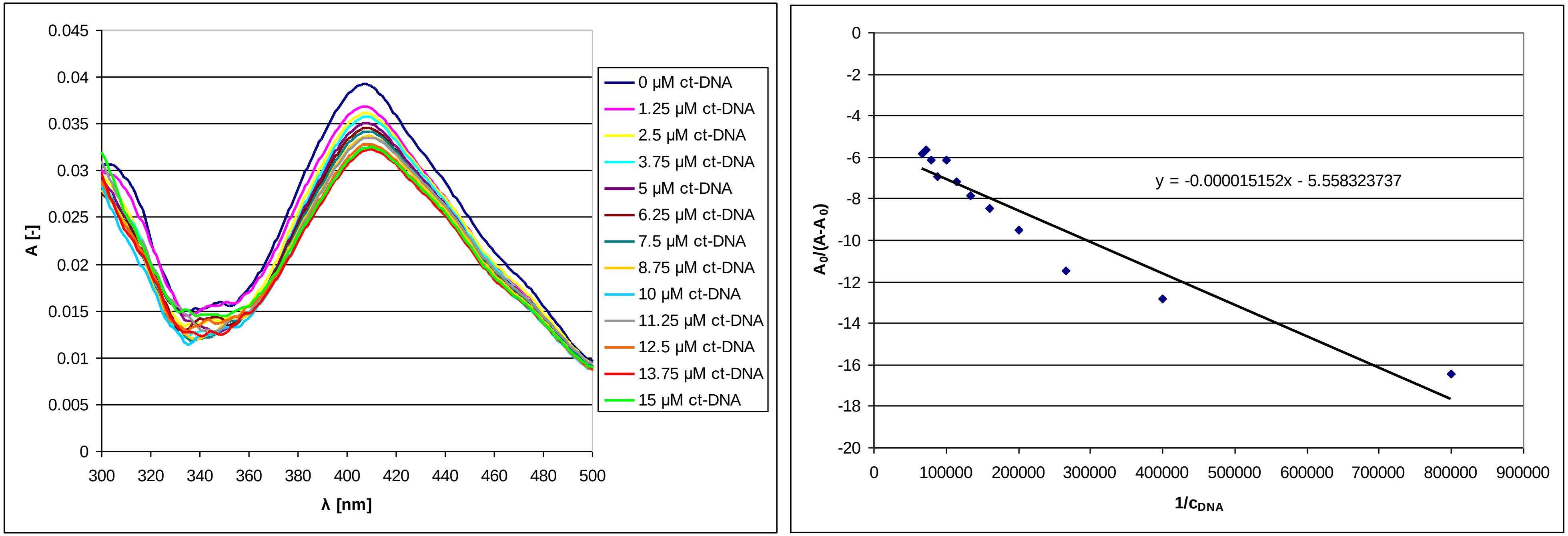

Figure S140. UV-vis absorption spectra of compound **18** (10 μM) in the presence of increasing amount of ct-DNA (0-15 μM) (left). The plot of $A_0/A-A_0$ versus 1/[DNA] yielded the binding constant (right).

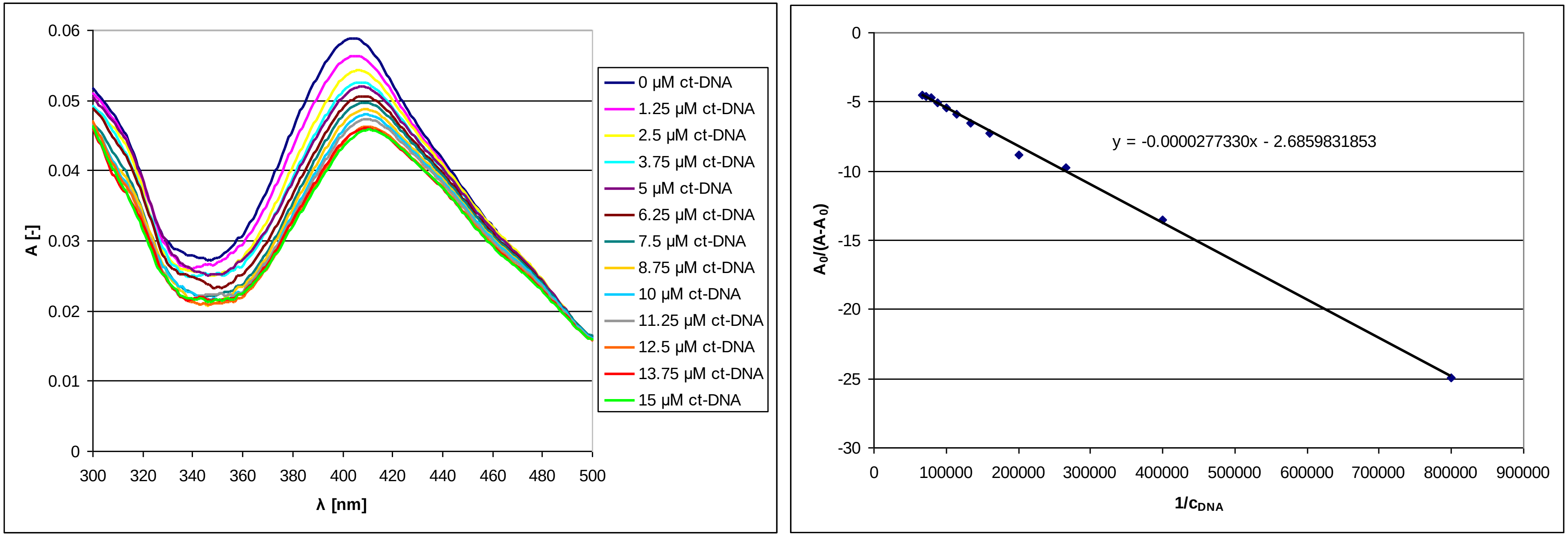

Figure S141. UV-vis absorption spectra of compound **19** (10 µM) in the presence of increasing amount of ct-DNA (0-15 µM) (left). The plot of $A_0/A-A_0$ versus 1/[DNA] yielded the binding constant (right).

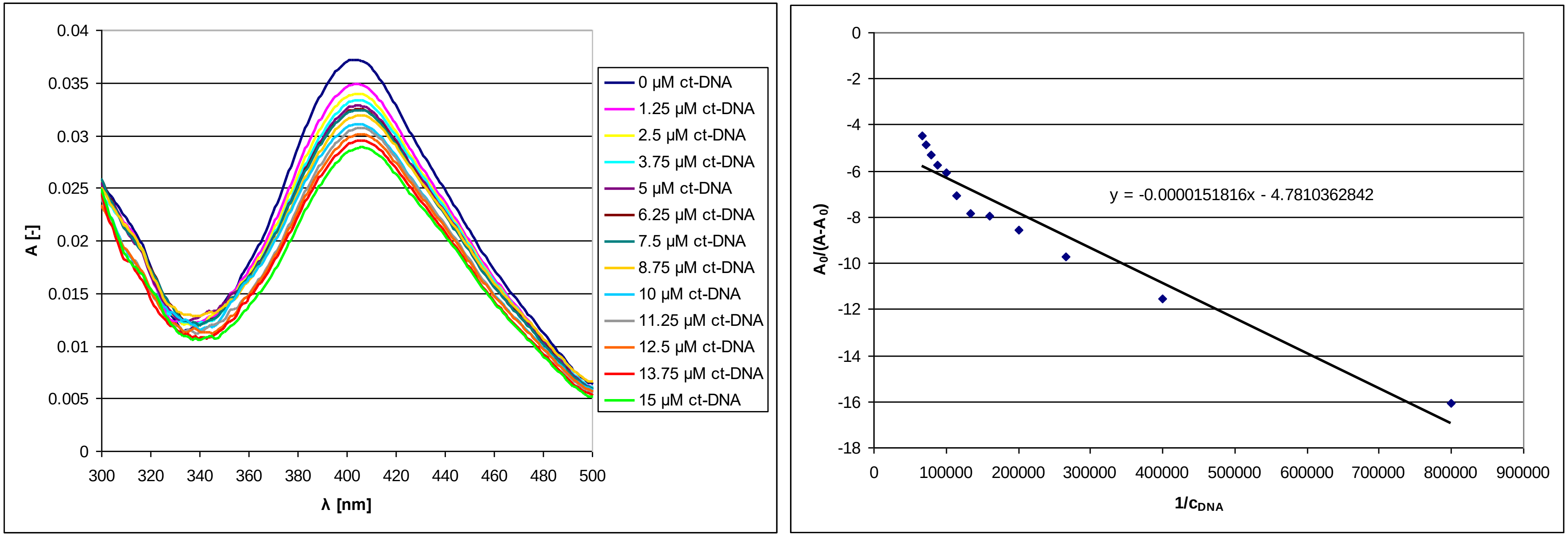

Figure S142. UV-vis absorption spectra of compound **30** (10 µM) in the presence of increasing amount of ct-DNA (0-15 µM) (left). The plot of $A_0/A-A_0$ versus 1/[DNA] yielded the binding constant (right).

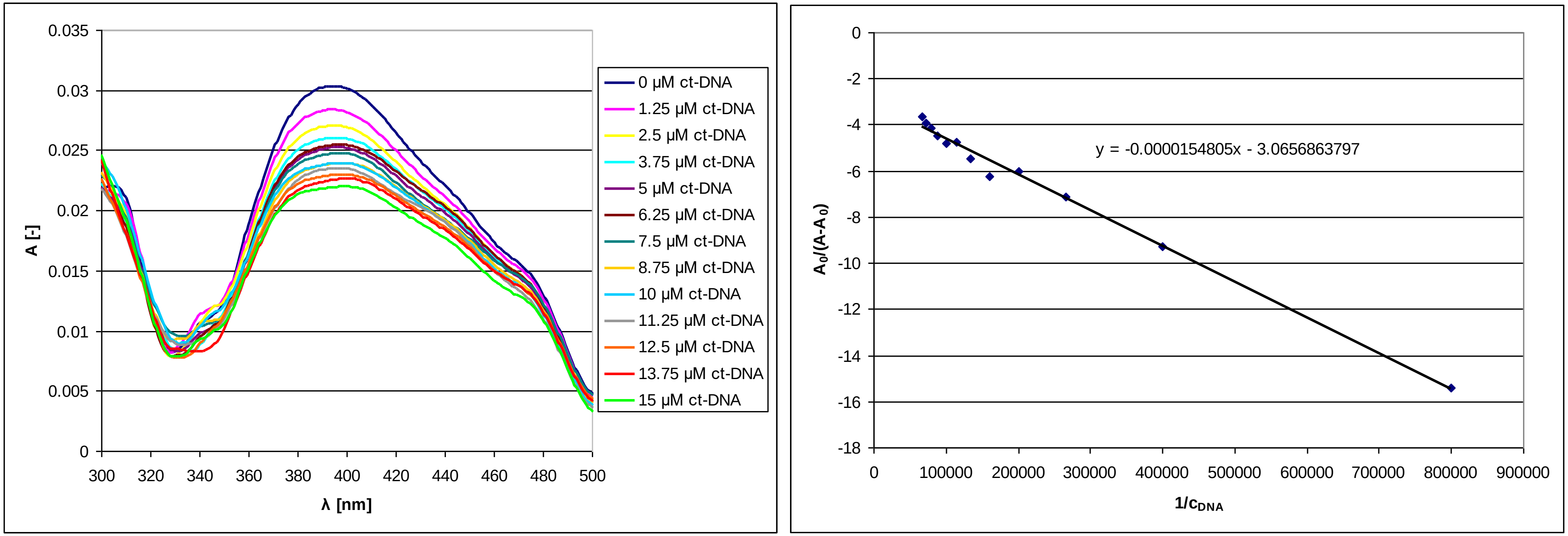

Figure S143. UV-vis absorption spectra of compound **31** (10 μM) in the presence of increasing amount of ct-DNA (0-15 μM) (left). The plot of A_0/A-A_0 versus 1/[DNA] yielded the binding constant (right).

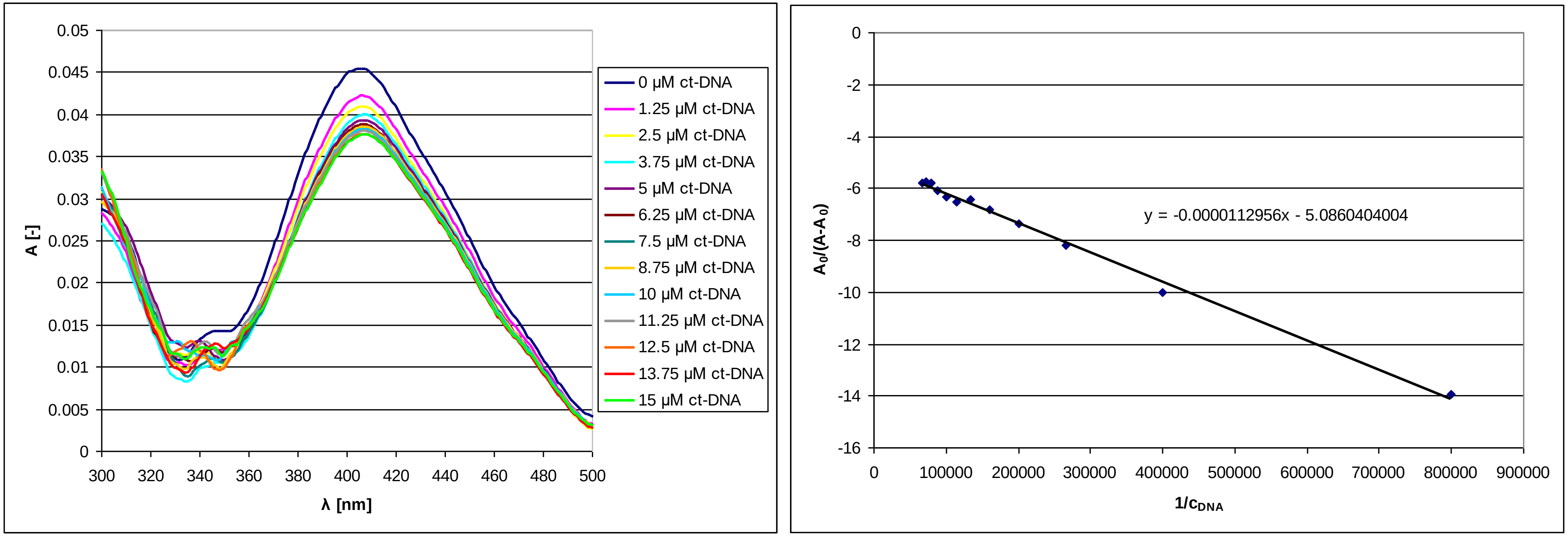

Figure S144. UV-vis absorption spectra of compound **32** (10 μM) in the presence of increasing amount of ct-DNA (0-15 μM) (left). The plot of $A_0/A-A_0$ versus 1/[DNA] yielded the binding constant (right).

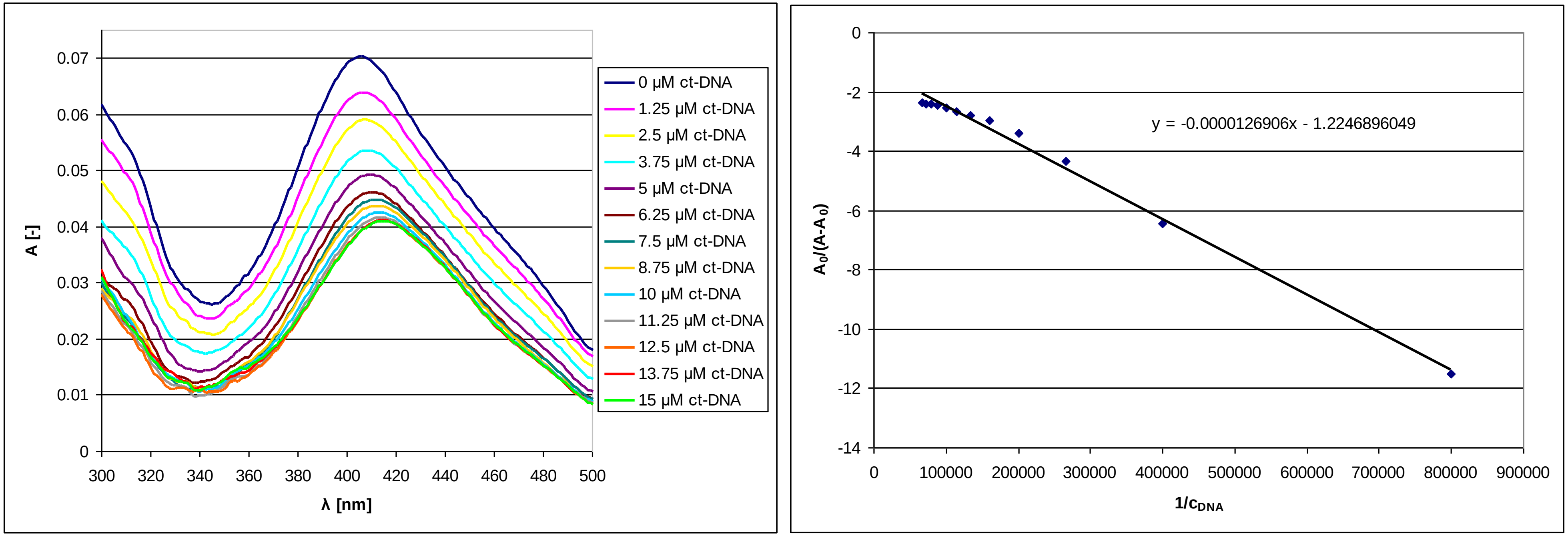

Figure S145. UV-vis absorption spectra of compound **34** (10 µM) in the presence of increasing amount of ct-DNA (0-15 µM) (left). The plot of $A_0/A-A_0$ versus 1/[DNA] yielded the binding constant (right).

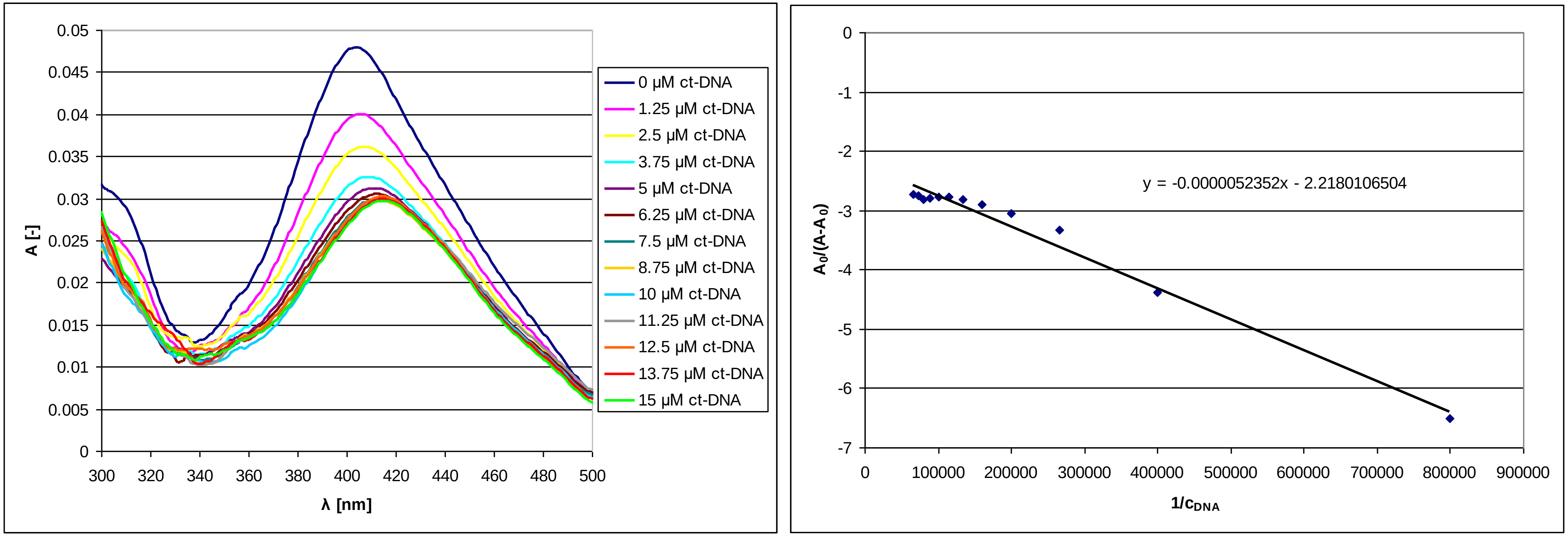

Figure S146. UV-vis absorption spectra of compound **35** (10 µM) in the presence of increasing amount of ct-DNA (0-15 µM) (left). The plot of $A_0/A\text{-}A_0$ versus 1/[DNA] yielded the binding constant (right).

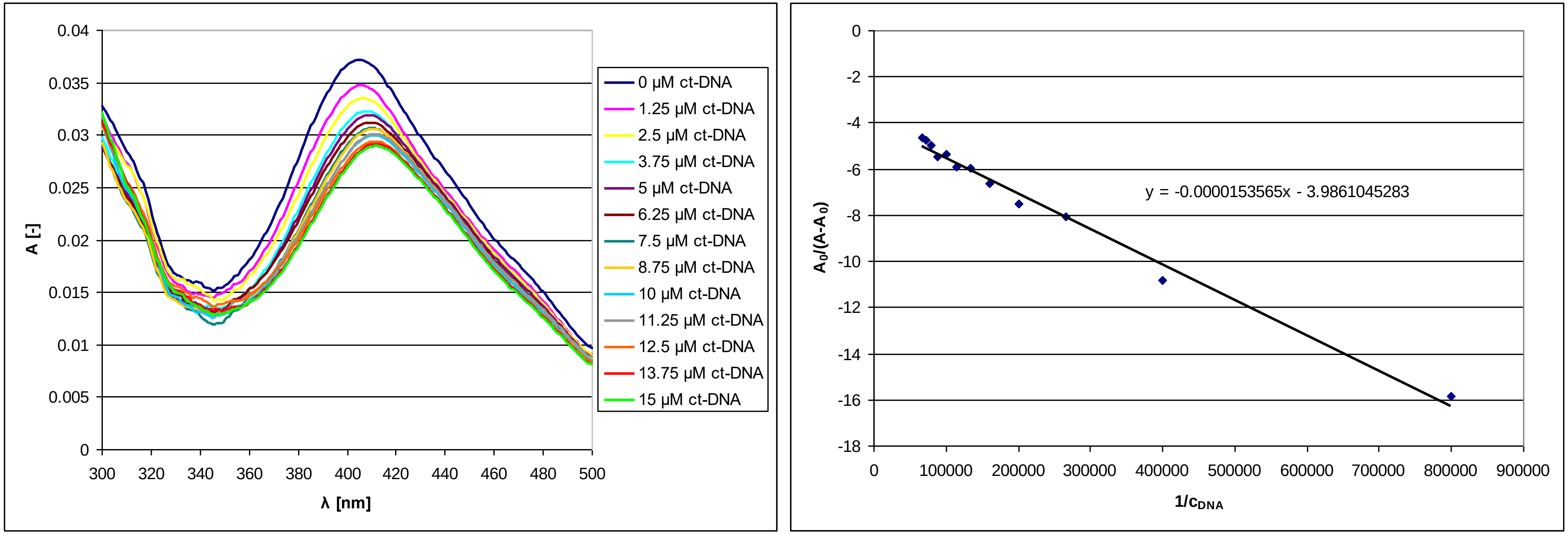

Figure S147. UV-vis absorption spectra of compound **36** (10 μM) in the presence of increasing amount of ct-DNA (0-15 μM) (left). The plot of $A_0/A-A_0$ versus 1/[DNA] yielded the binding constant (right).

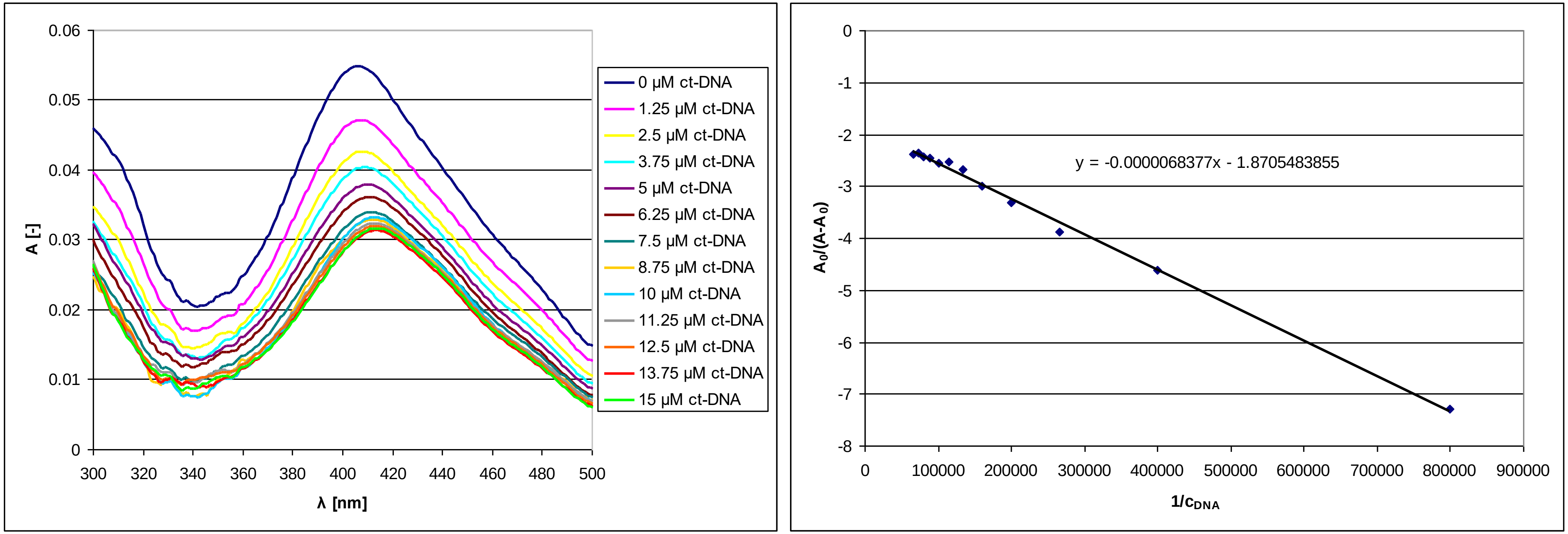

Figure S148. UV-vis absorption spectra of compound **37** (10 µM) in the presence of increasing amount of ct-DNA (0-15 µM) (left). The plot of $A_0/A-A_0$ versus 1/[DNA] yielded the binding constant (right).

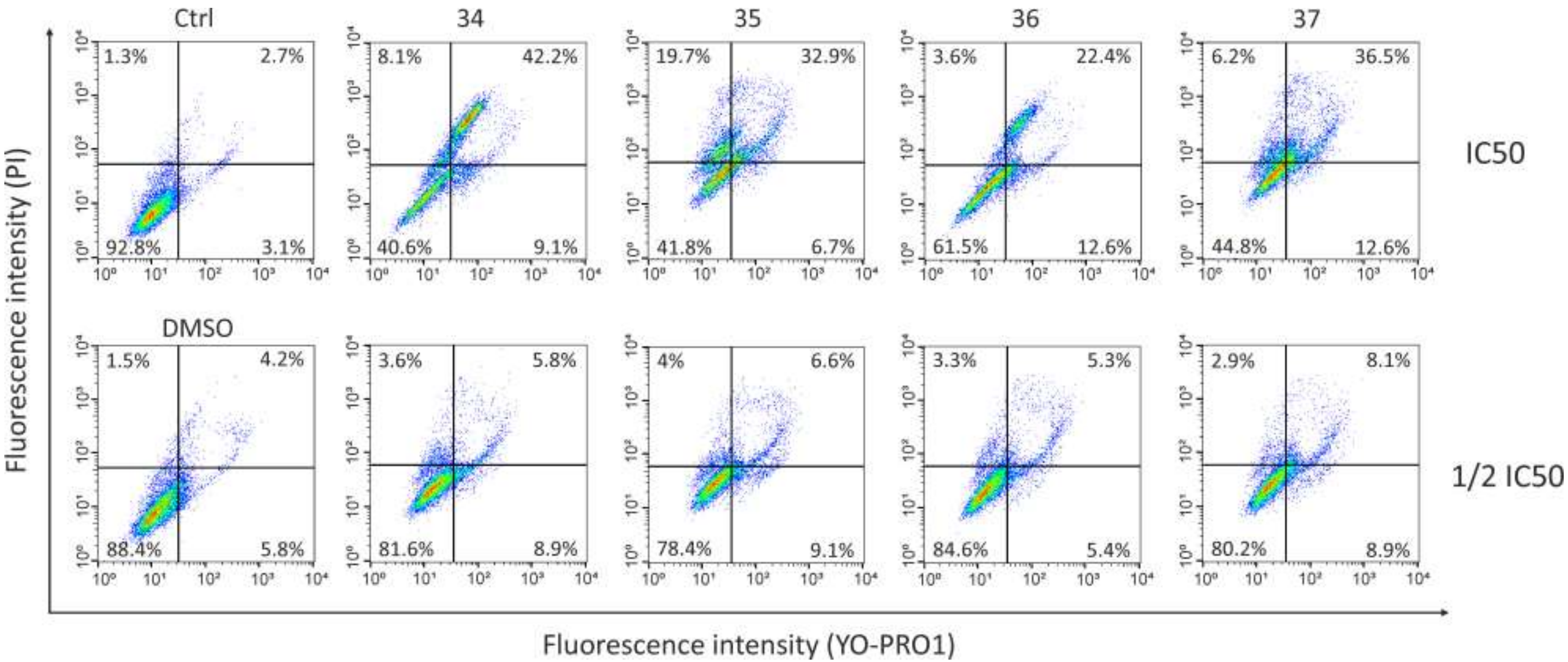

Figure S149. Apoptosis/necrosis analysis in HepG2 cells after 24 h treatment with compounds **34**-**37**. The cells were incubated with the compounds for 24 h, and apoptosis induction was estimated by flow cytometry using YO-PRO1/PI double staining. Live (lower left quadrant), apoptotic (lower right and upper right quadrant) and necrotic cells (upper right quadrant) were counted and the mean number of cells in each quadrant from three replicates is presented on the graph.

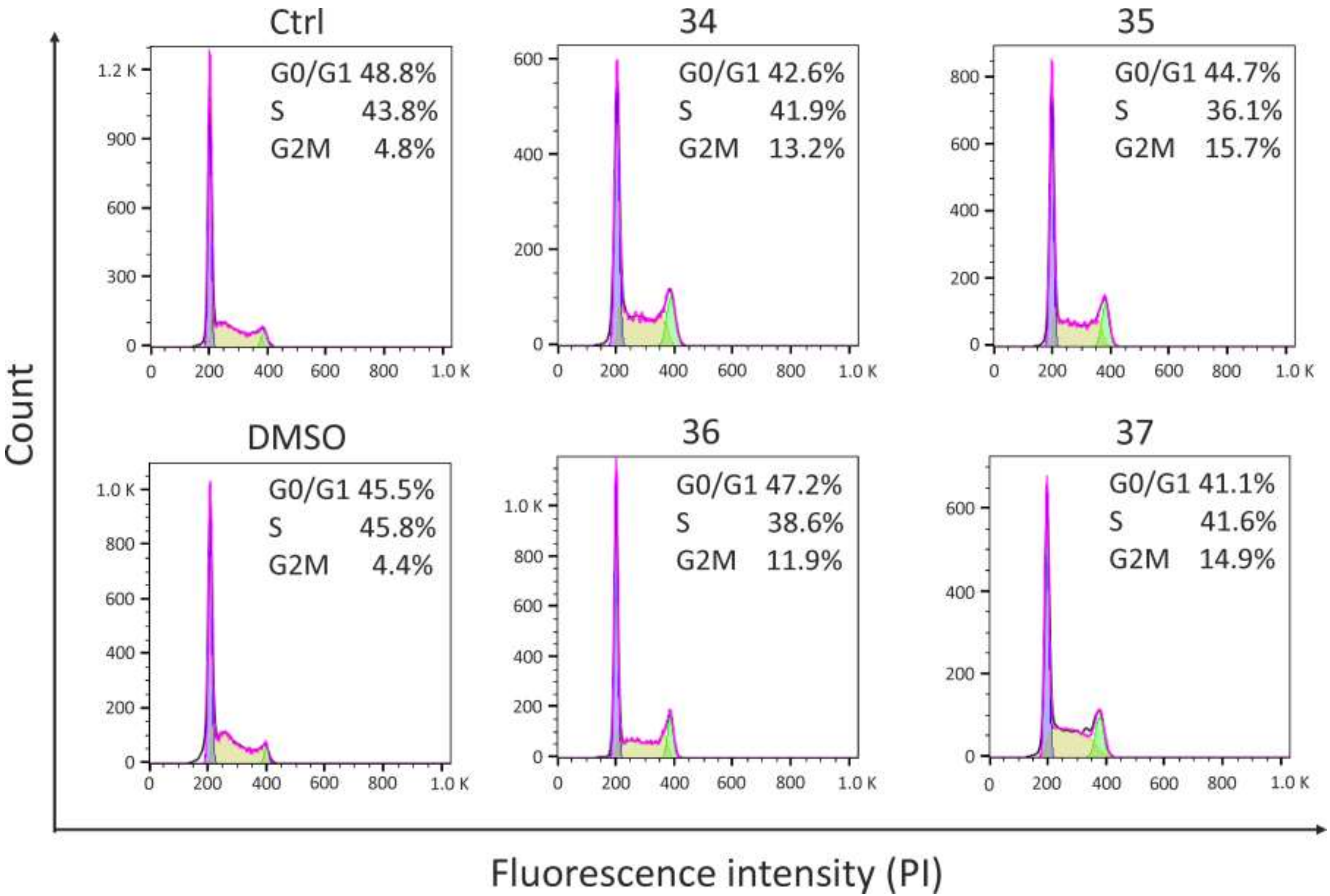

Figure S150. Cell cycle analysis using flow cytometry in HepG2 cells. Graphical representation of flow cytometry analysis of cell cycle using PI staining after 24 h of compounds administration. The number of cells in each phase of cell cycle was estimated, and presented as a mean form three replicates.

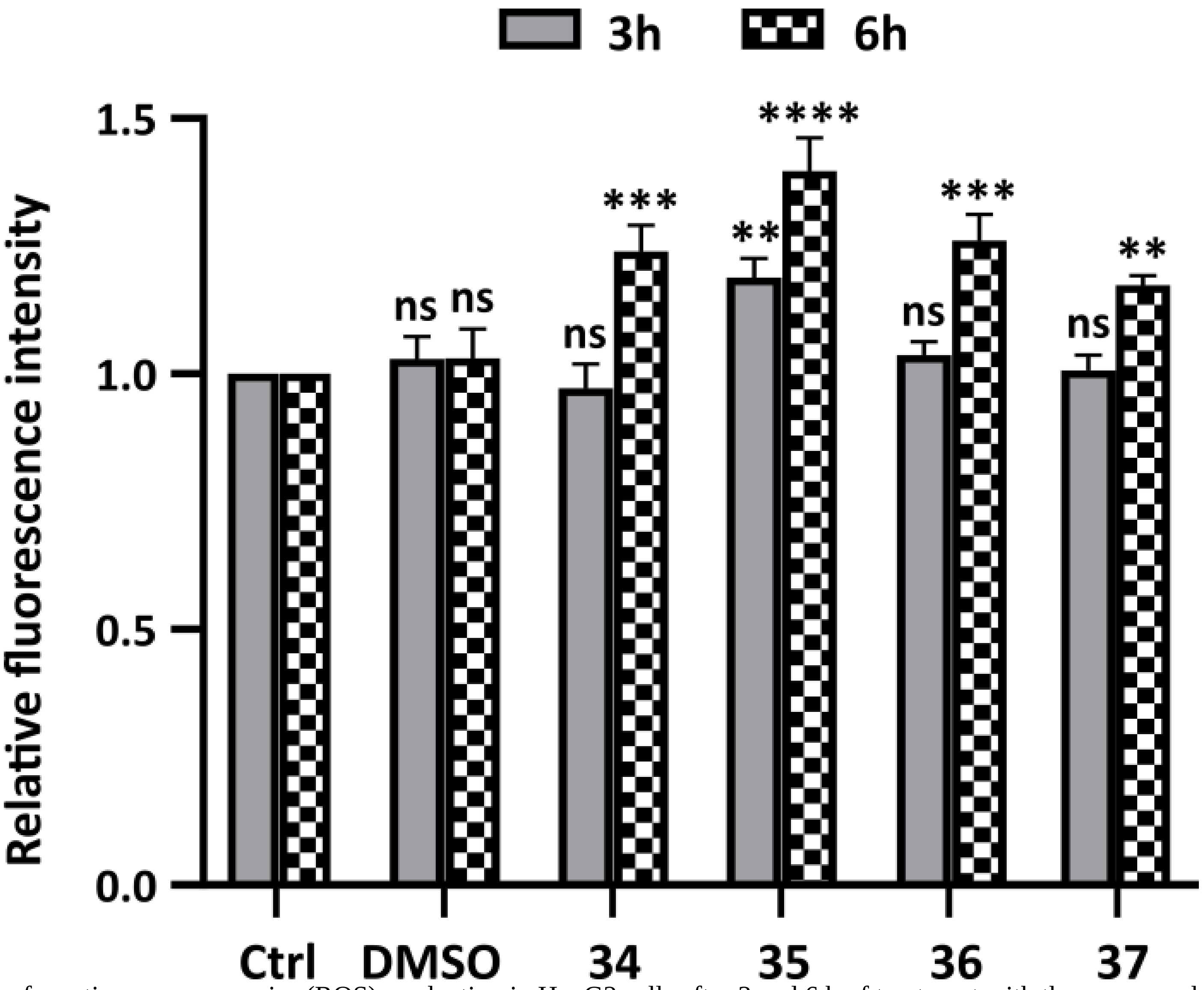

Figure S151. Analysis of reactive oxygen species (ROS) production in HepG2 cells after 3 and 6 h of treatment with the compounds **34-37**.
The cells were treated with the compounds in a concentration corresponding to IC_{50} value, and the ROS production was analyzed using H_2DCFDA/PI double staining using flow cytometer with excitation at 488 nm. Statistical significance is indicated with asterisks: (ns) $p < 0.05$, ** $p < 0.01$, *** $p < 0.001$, **** $p < 0.0001$.

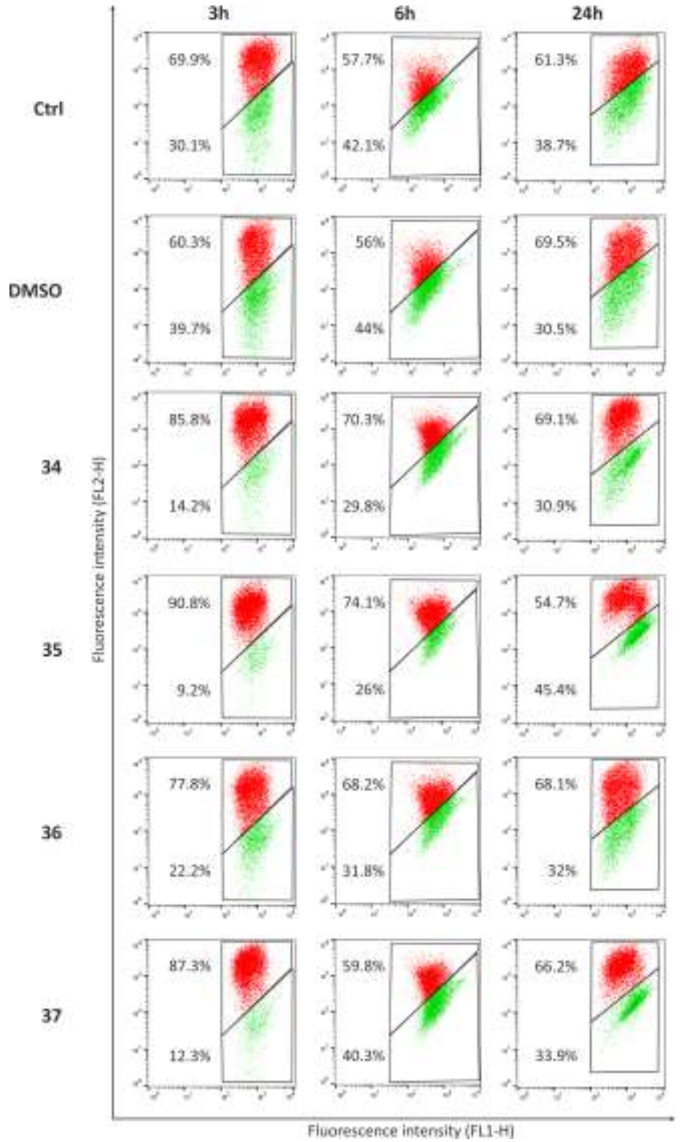

Figure S152. Mitochondrial membrane potential (MMP) analysis in HepG2 cells treated with compounds **34-37**.The cells were treated with the analyzed compounds in a final concentration corresponding to IC_{50} value, and MMP was analyzed after 3, 6 and 24 h of incubation via flow cytometry using JC-1 fluorescent probe. 3 µM FCCP was used as a negative control emitting strong green fluorescent signal, emitted by the dye monomers. Fluorescence signal from aggregates (red) and monomers (green) was gated and showed as a representative dot plots.

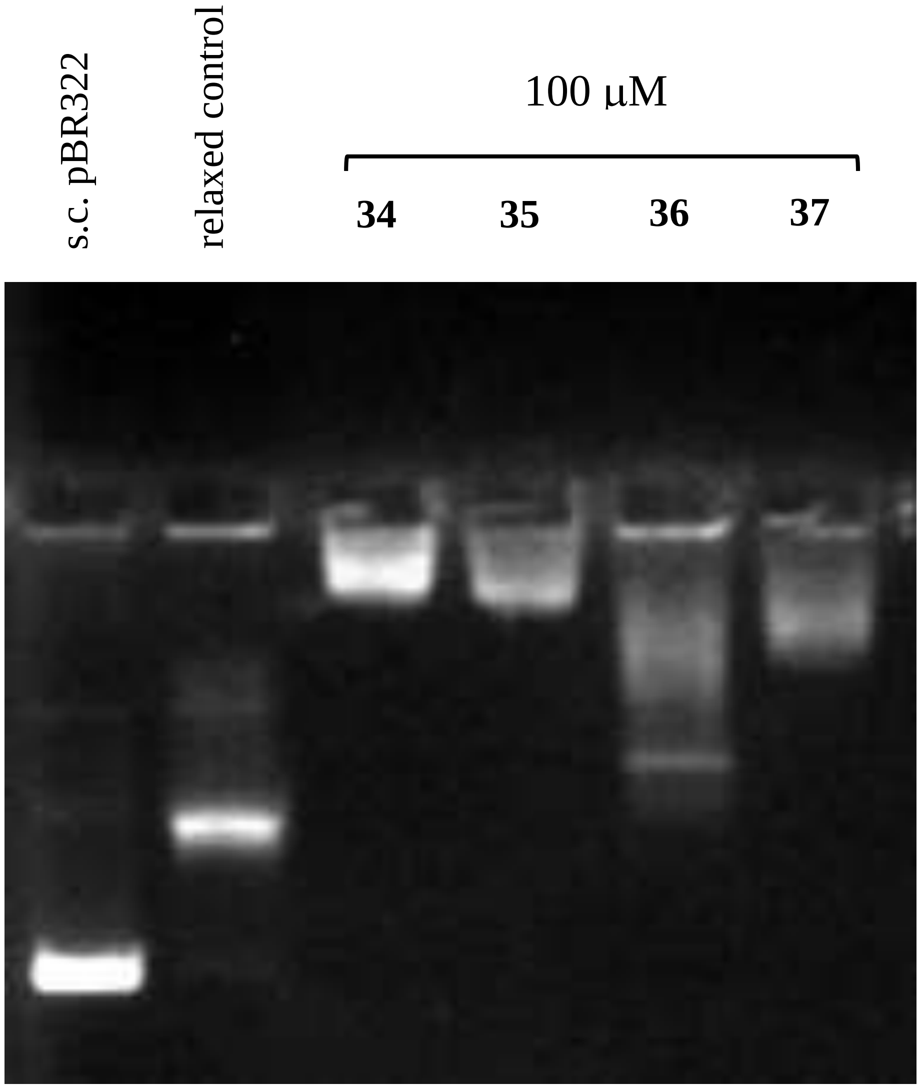

Figure S153. Human topoisomerase IIα relaxation assay in the presence of conjugates modified with carborane-1,8-naphthalimides **34-37** at the concentration of 100 µM. s.c. pBR322 – supercoiled plasmid DNA.

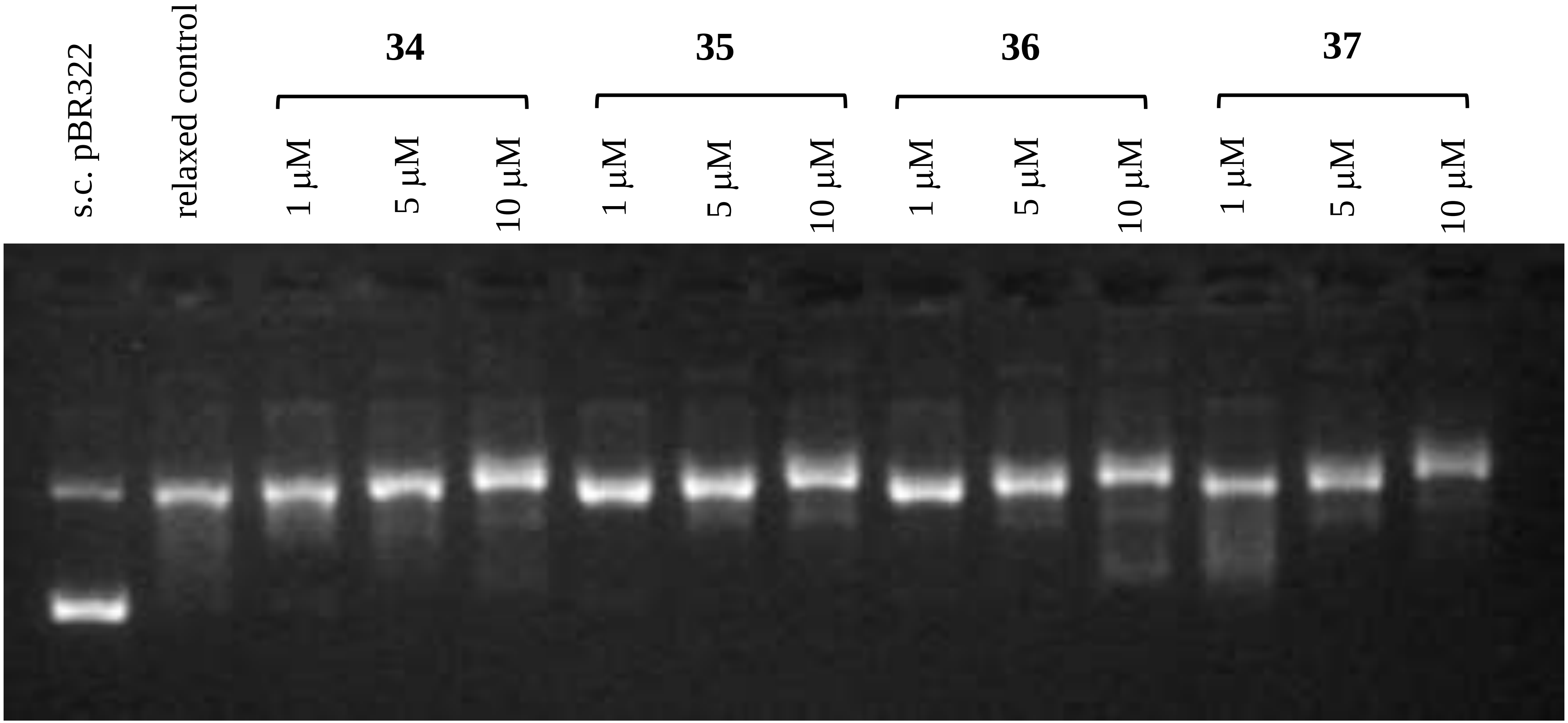

Figure S154. Inhibition of the relaxation activity of human topoisomerase IIα in the presence of compounds **34-37** at the concentrations of 1, 5, and 10 µM. s.c. pBR322 – supercoiled plasmid DNA.

Table S1. Crystallographic data.

Compound	**15**
Wavelength [Å]	1.54184
Temperature [K]	100
Space group	Cc
Z	12
a [Å]	16.8780(14)
b [Å]	52.734(3)
c [Å]	11.1940(6)
α [°]	90
β [°]	108.441(7)
γ [°]	90
R_{sym}/R_{pim}	0.0790/0.0442
Resolution [Å]	0.870
% completeness	0.85
Parameters in refinement	1261
Unique reflections (F^+/F^- unmerged)	12835
R/R(for $F_o>4\sigma$)	0.1678/0.0793
CSD code	CCDC 2205389
